# A New Hadrosaurine (Dinosauria: Hadrosauridae) from the Marine Deposits of the Late Cretaceous Hakobuchi Formation, Yezo Group, Japan

**DOI:** 10.1038/s41598-019-48607-1

**Published:** 2019-09-05

**Authors:** Yoshitsugu Kobayashi, Tomohiro Nishimura, Ryuji Takasaki, Kentaro Chiba, Anthony R. Fiorillo, Kohei Tanaka, Tsogtbaatar Chinzorig, Tamaki Sato, Kazuhiko Sakurai

**Affiliations:** 1 Hokkaido University Museum, Hokkaido University, Sapporo, Hokkaido 060-0810 Japan; 2 Hobetsu Museum, Mukawa, Hokkaido 054-0211 Japan; 3 Department of Natural History and Planetary Sciences, Hokkaido University, Sapporo, Hokkaido 060-0810 Japan; 4 Faculty of Biosphere-Geosphere Science, Okayama University of Science, Okayama, 700-0005 Japan; 5 Perot Museum of Nature and Science, Dallas, Texas 75201 United States; 6 Graduate School of Life and Environmental Sciences, University of Tsukuba, Tsukuba, Ibaraki 305-8572 Japan; 7 Division of Vertebrate Paleontology, Institute of Paleontology and Geology, Mongolian Academy of Sciences, Ulaanbaatar, 15160 Mongolia; 8 Department of Astronomy and Earth Sciences, Tokyo Gakugei University, Koganei, Tokyo 184-8501 Japan

**Keywords:** Palaeontology, Environmental impact

## Abstract

A nearly complete skeleton of a new hadrosaurid, *Kamuysaurus japonicus* gen. et sp. nov., was discovered from the outer shelf deposits of the Upper Cretaceous Hakobuchi Formation of the Yezo Group in Hobetsu area of Mukawa town in Hokkaido, Japan. *Kamuysaurus* belongs to the sub-clade of Hadrosaurinae, Edmontosaurini, and forms a monophyly with *Laiyangosaurus* and *Kerberosaurus* from the northern Far East. *Kamuysaurus* has a long anterior platform for the nasofrontal sutural surface, which may indicate the presence of a small supracranial crest, similar to a sub-adult form of *Brachylophosaurus* based on the extension of the nasofrontal sutural surface. The Dispersal Extinction Cladogenesis analysis with the 50% Majority Rule consensus tree suggests that the clade of *Kamuysaurus*, *Laiyangosaurus*, and *Kerberosaurus* may have dispersed into Asia prior to the late Campanian and the potential endemism of this clade during the late Campanian and early Maastrichtian in the northern Far East. The results of both Dispersal Extinction Cladogenesis and Ancestral State Reconstruction analyses imply that the marine-influenced environment in North America during the Campanian may have played an important role for the hadrosaurid diversification in its early evolutionary history.

## Introduction

Hadrosaurid dinosaurs are one of the most successful herbivorous dinosaurs in the Late Cretaceous, and these fossil remains are common in the uppermost Cretaceous (Campanian and Maastrichtian) deposits in Laurasia (North America, Asia, and Europe) and some areas of Gondwana (South America and Antarctica)^1,2^. Asian countries have yielded remains of both subclades of Hadrosauridae, Hadrosaurinae^3^ (or Saurolophinae^4^) and Lambeosaurinae, from Maastrichtian deposits. Most of Maastrichtian hadrosaurid taxa have been recovered from eastern Asia, where the richest area is the set of exposures along the Heilong Jiang (River) of China (*Wulagasaurus*^5^ for a hadrosaurine and *Sahaliyania*^5^ and *Charonosaurus*^6^ for lambeosaurines) or the Amur River of Russia (*Kerberosaurus*^7^ for a hadrosaurine and *Amurosaurus*^8^ and *Olorotitan*^9^ for lambeosaurines). The Nemegt Formation of Mongolia is the only inland area with discoveries of Maastrichtian hadrosaurids in eastern Asia and has yielded the hadrosaurine *Saurolophus*^10^ and *Barsboldia*^11^. A lambeosaurine specimen has been reported from the Maastrichtian deposits in Awaji Island of Hyogo, Japan, but its taxonomic assignment is not resolved yet^12^.

Here, we report a new taxon of a hadrosaurid dinosaur, the first nearly complete mid-to-large sized dinosaur skeleton from Japan. This specimen was recovered from the marine deposits of the Late Cretaceous Hakobuchi Formation (early Maastrichtian) of the Yezo Group in Hokkaido, Japan, during the joint excavation of the Hobetsu Museum and Hokkaido University Museum, that largely occurred in 2013 and 2014. This new hadrosaurid contributes to our understanding of the diversity of hadrosaurids in marine-influenced environments because hadrosaurid materials from marine deposits are rarely reported: with notable exceptions such as the lambeosaurine *Nipponosaurus* from Russia^13^, the hadrosaurine *Hadrosaurus*^14^, *Lophorhothon*^15^, and *Augustynolophus*^16^ from USA, and other fragmentary materials^1,17^. This new specimen also sheds light on our understanding of the diversity of hadrosaurids in the Far East, and the spatial and environmental significance of hadrosaurid evolution during the Late Cretaceous.

## Results

### Geology

**Figure 1:**
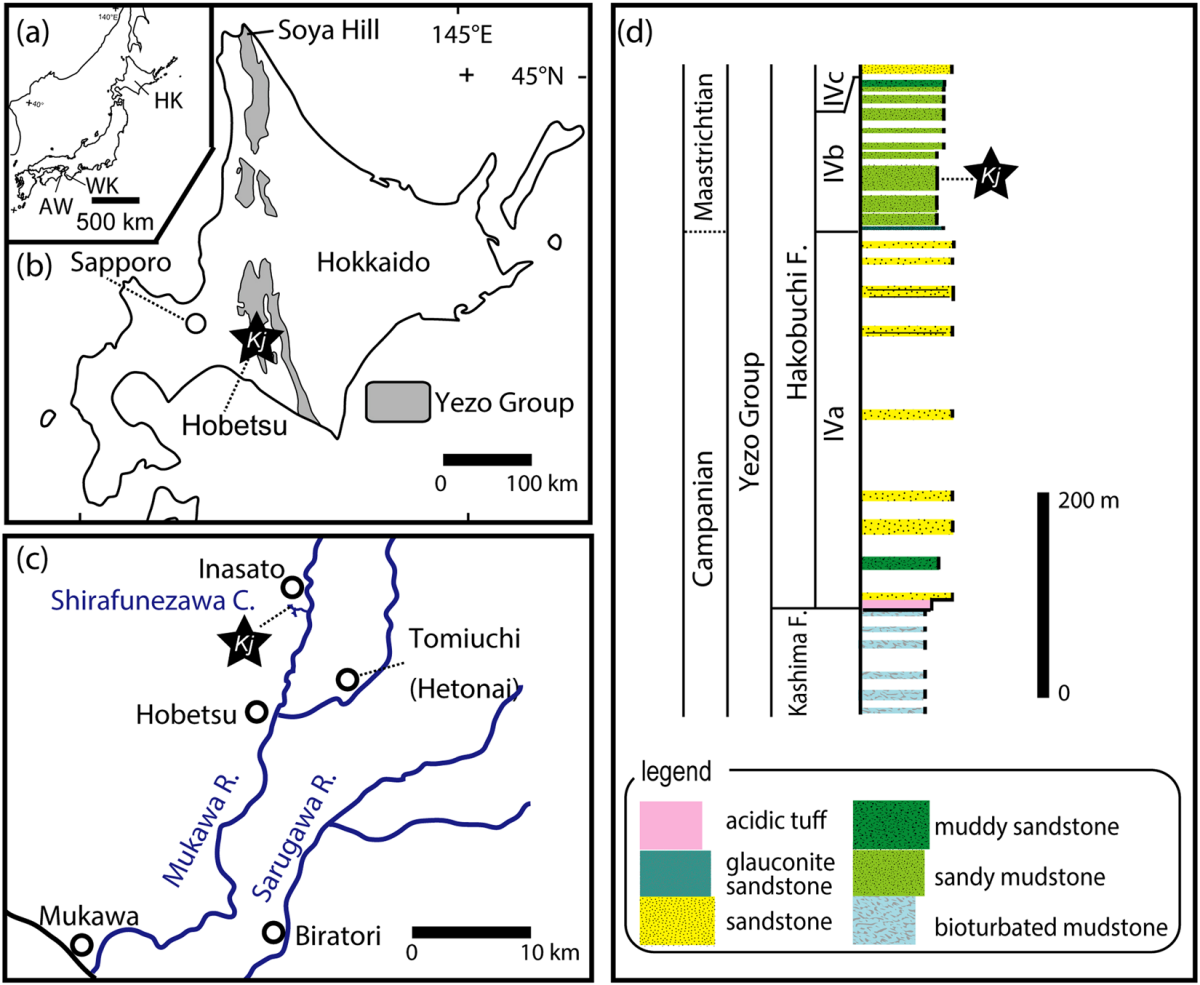
Geology and stratigraphy of the *Kamuysaurus japonicus* gen. et sp. nov. (**a**) Map of Japan, showing the location of Hokkaido (HK) and Wakayama (WK) prefectures and Awaji Island of Hyogo Prefecture (AW) (Supplementary Text S1). (**b**) Map of Hokkaido, showing the distribution of the Cretaceous Yezo Group and the location of *Kamuysaurus* locality (*Kj* in a black star). (**c**) Map of the northern Hobetsu area (Inasato) of Mukawa town, showing the location of *Kamuysaurus* locality (*Kj* in a black star) along the Shirafunezawa Creek, which is a branch of the Hobetsu River. (**d**) Stratigraphic column along the Shirafunezawa Creek, showing the horizon of *Kamuysaurus japonicus* (*Kj* in a black star).

The Hakobuchi Formation is the uppermost unit of the Yezo Group, which is a part of Cretaceous to Paleocene forearc basin deposits that crop out in the southern Hokkaido, Japan (Fig. 1a,b; Supplementary Text S1 and Figs S1 and S2)^18,19^. The specimen described in this study was discovered from an outcrop of the middle part of IVb rock unit consisting of mainly sandy mudstone, along the Shirafunezawa Creek in the northern Hobetsu area of Mukawa town in Hokkaido (Fig. 1c,d). This unit is correlated to the lowest Maastrichtian in age and has yielded ammonoids, mosasaurs and a sea turtle^20–24^. Its depositional environment is considered outer shelf because of the presence of glauconite sandstone and the absence of hummocky cross-stratification. A semi-articulated, nearly complete skeleton was excavated in an area of 4 m × 7 m. Despite its completeness, some bones are heavily damaged or eroded by a bio-erosion prior to burial.

### Systematic paleontology

Dinosauria Owen^25^

Ornithischia Seeley^26^

Ornithopoda Marsh^27^

Hadrosauridae Cope^28^

Hadrosaurinae Lambe^29^

*Kamuysaurus japonicus* gen. et sp. nov.

### Etymology

“*Kamuy*” refers deity to the Ainu, which is indigenous people in Hokkaido Island of Japan, and “*sauros*” means reptile in Latin. Specific name, “*japon*”, refers to Japan.

### Holotype

A nearly complete skeleton with the skull and mandible (HMG-1219), housed at the Hobetsu Museum, Hobetsu area of Mukawa town, Hokkaido, Japan.

### Locality and horizon

An outcrop (42° 50′ 48″N, 142° 7′ 20″E) along the Shirafunezawa Creek in the northern Hobetsu area of Mukawa town in Hokkaido; the middle of IVb unit (early Maastrichtian) of the Hakobuchi Formation of the Yezo Group.

### Diagnosis

This specimen is assigned as a hadrosaurine hadrosaurid with the following unique characters: the midpoint of the quadratojugal notch positioned at roughly three-quarters of the total length of the quadrate from the dorsal end, short ascending process of the surangular, and anterior inclination of neural spines of sixth to thirteenth dorsal vertebrae. Diagnosed also by the unique combination of characters: slightly curved primary ridge of the maxillary teeth, high average height/width ratio of dentary tooth over 3.30, moderate medial extension of the symphyseal process of the dentary, anterior margin of the coronoid process of the dentary more developed than the posterior margin, triangular ventral margin of the anterior process of the jugal as wide as its height, moderately inclined palatine articular facet of the jugal, nearly straight caudal margin of the quadratojugal flange of the jugal, smoothly curved anterodorsal margin of the prefrontal along the orbital rim, squamosal process of the postorbital terminates anterior to the quadrate cotylus, long nasofrontal sutural surface of the frontal, subrectangular infratemporal fenestra, weak expansion of deltopectoral crest of the humerus, and slender humerus with humeral shaft less than 20% as wide as the length.

### Description

The skeleton with skull and mandible is nearly complete (Supplementary Tables S1) except for the rostrum of the skull and sacral vertebrae although many of the elements are damaged by bio-erosion.

**Figure 2:**
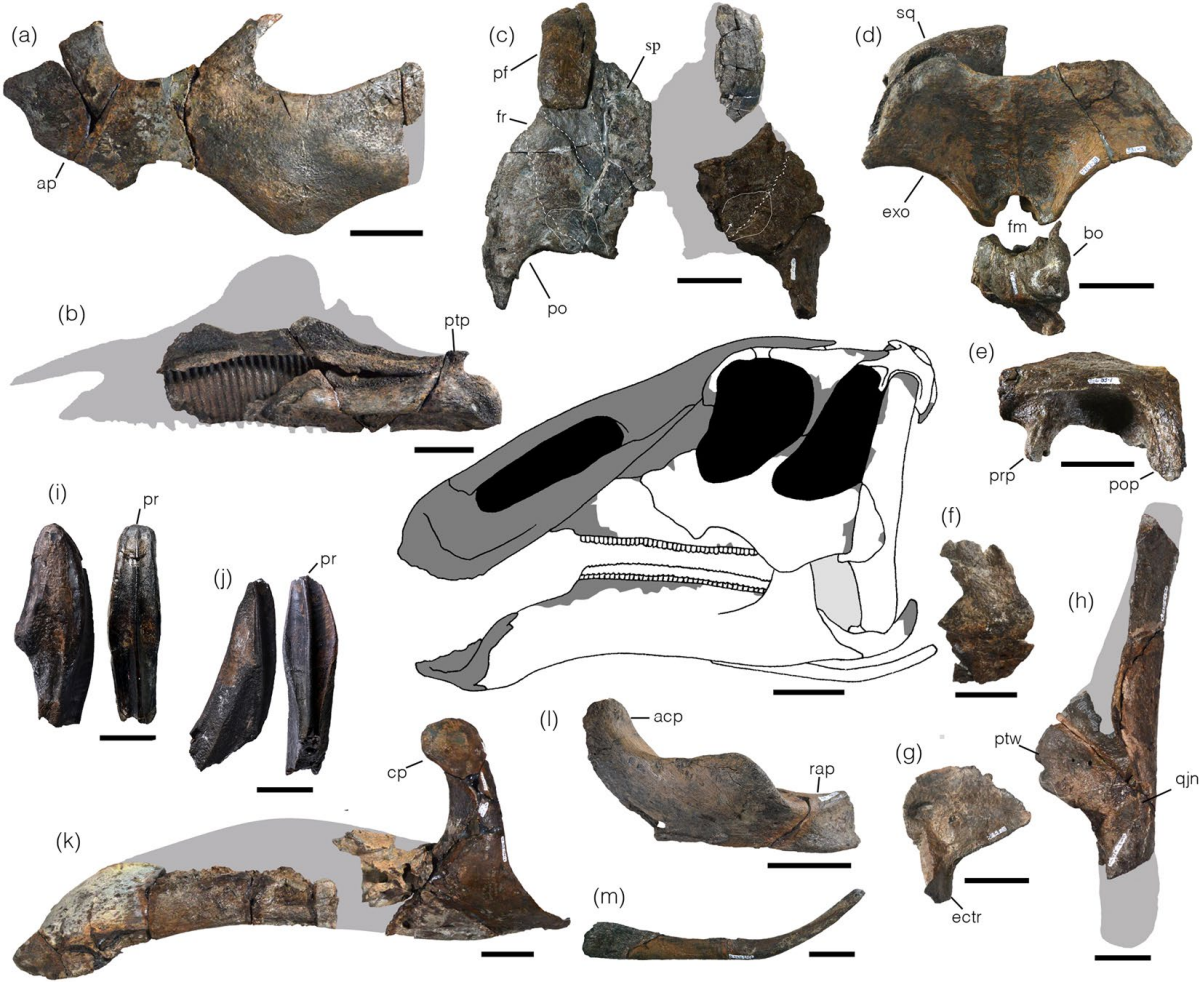
Selected skull elements of *Kamuysaurus japonicus* gen. et sp. nov. (**a**) Right jugal in lateral view (reversed); (**b**) right maxilla in lateral view (reversed); (**c**) prefrontals, frontals, and postorbitals in dorsal view; (**d**) squamosal, exoccipital, basioccipital, and basisphenoid in posterior view; (**e**) left squamosal in lateral view; (**f**), right quadratojugal in lateral view (reversed); (**g)** left pterygoid in lateral view; (**h**) left quadrate in lateral view; (**i**) maxillary tooth in labial and distal views; (**j**) dentary tooth in lingual and distal views; (**k**) right dentary in lateral view (reversed); (**l**) right surangular in lateral view (reserved); (**m**) right ceratobranchial in lateral view (reversed). All scales are 5 cm except 1 cm scale bar for (**i**,**j**). Abbreviations: acp, ascending process; ap, anterior process; bo, basioccipital; cp, coronoid process; ectr, ectopterygoid ramus; exo, exoccipital; fm, foramen magnum; fr, frontal; pf, prefrontal; po, postorbital; pop, postcotyloid process; pr, primary ridge; prp, precotyloid process; ptp, pterygoid process; ptw, pterygoid wing; qjn, quadratojugal notch; rap, retroarticular process; sp, sutural platform; sq, squamosal. Grey areas are missing parts.

The finger-like pterygoid process of the maxilla projects posterodorsally (Fig. 2b; Supplementary Fig. S3). The ectopterygoid shelf is thick and horizontally oriented. The right maxilla preserves 39 alveoli along a length of 234 mm, suggesting that the average alveolus width is 6 to 7 mm. The line of the nutrient foramina forms a dorsally convex arc, and its highest point is positioned on the dorsal half of the maxilla body, which is typical for hadrosaurids.

The dorsal surface of the prefrontal is smooth (Fig. 2c; Supplementary Fig. S4), lacking a ridge seen in lambeosaurines^5^. The prefrontal forms a smoothly curved anterodorsal corner of the orbital rim unlike some hadrosaurines (*Edmontosaurus*, *Gryposaurus*, *Kerberosaurus*, *Kritosaurus*, and *Secernosaurus*)^7,30,31^ with an angled corner. The anterodorsal corner of the orbital rim lacks an eversion.

The maximum depth of the anterior process of the jugal is almost twice as deep as the depth of the jugal below the orbit (Fig. 2a; Supplementary Fig. S5). The lacrimal articulation facet is slightly recurved posteriorly. The anterior process is wedged-shaped and pointed as in most hadrosaurines. The ventral side of the anterior process has a triangular-shaped ventral process as in *Gryposaurus* (e.g., RTMP 1980.022.0001) and *Brachylophosaurus* (e.g., FMNH PR 862) but unlike the bowed ventral process of *Edmontosaurus* (e.g., DMNH EPV 130653)^32^. The ridge for the palatine articular facet on the medial surface of the anterior process is inclined anteriorly with an angle of 114° from the line between the lower ends of orbit and infratemporal fenestra, which is also a feature present in *Gryposaurus*^32^. The apex of the ventral process is positioned posteroventral to the dorsal process as in non-hadrosaurid hadrosauroids and most hadrosaurines. The width of the orbital margin is roughly the same as that of the infratemporal fenestra. The concavity of the ventral border of the jugal between the ventral process of the anterior process and the posteroventral flange is deep and narrow unlike the shallow and wide concavity of *Saurolophus* (e.g., MPC-D 100/708) and *Prosaurolophus* (e.g., USNM 12712). The posteroventral flange of the jugal is not strongly developed. The maximum depth of the jugal at the ventral flange is 26% deeper than the minimum depth at the contractive portion of the jugal. The posterior process is massive and lacks a deep posteroventral concavity as in *Edmontosaurus* (e.g., DMNH EPV 130653, CMN 2288) but unlike *Maiasaura* (e.g., ROM 44770), *Brachylophosaurus* (e.g., FMNH PR 862), or *Gryposaurus* (e.g., RTMP 1980.022.0001). The ventral border of the posterior process is nearly parallel to the dorsal border. The posterior border of the posterior process is nearly straight.

The quadrate (Fig. 2h; Supplementary Fig. S6) is nearly straight in lateral view as in hadrosaurines except *Shantungosaurus*^3^. The squamosal buttress is absent as in some hadrosaurines (*Brachylophosaurus*, *Gryposaurus*, *Kritosaurus*, and *Maiasaura*)^31,33–35^. The midpoint of the quadratojugal notch is positioned at roughly three-quarters, or larger, of the total length of the quadrate from the dorsal end. The ventral position of the notch is common in hadrosaurines, but that of *Kamuysaurus* is the most ventrally positioned among hadrosaurids. The notch is widely arcuate and shallow as in other hadrosaurines except *Saurolophus*^36^. The dorsal margin of the notch is angled by 35° from the main axis of the quadrate. The ventral end of the quadrate is nearly as long as wide. The medial condyle is reduced and placed more dorsally than the lateral condyle. Medial to the pterygoid wing is a posterolateral spur.

The ventral edge of the lamina of the pterygoid between the ectopterygoid ramus and the ventral quadrate process is concave (Fig. 2g; Supplementary Fig. S6).

The squamosal (Fig. 2e; Supplementary Fig. S7) is low dorsoventrally above the quadrate cotylus unlike lambeosaurines. The dorsal surface is smooth and does not have a recessed surface for the squamosal process of the postorbital, indicating that the position of the posterior tip of the squamosal process does not reach to the quadrate cotylus as in some hadrosaurines (*Gryposaurus* and *Kritosaurus*)^31,34^. The precotyloid process is offset medially from the main body of the squamosal with a horizontal shelf above the process as in *Maiasaura* (e.g., ROM 44770). The dorsoventral length of the process is shorter than the horizontal width of the quadrate cotylus and is shorter than the postcotyloid process.

The dorsal surface of the postorbital (Fig. 2c; Supplementary Fig. S4) is flat, and a dorsal promontorium is absent as in most of hadrosaurines. The lateral border forms a horizontal dorsal rim and a strongly curved posterodorsal corner of the orbit in lateral view. The base of the postorbital bar lacks a deep inner cavity unlike in *Edmontosaurus*^3,30^. On the ventral surface of the postorbital and frontal there is a deep pit, where the lateral process of the laterosphenoid articulates.

The frontal (Fig. 2c; Supplementary Fig. S4) participates in the orbital rim. The articular surface with the prefrontal is deeply excavated, and its posterior end is pointed. Medial to the prefrontal contact surface is an anteroposteriorly long and gently sloped sutural surface, extending posterior to the prefrontal contact surface. Although this surface is slightly damaged by a bio-erosion, it probably retains the original shape of a sutural surface with the nasal. The frontal is as long as wide as in non-hadrosaurid hadrosauroids and hadrosaurines. The presphenoid and orbitosphenoid are firmly fused to the frontal. The frontal-presphenoid-orbitosphenoid complex has a cup-shaped depression for the cerebral fossa, and anterior to the cerebral fossa has a shallow depression for the olfactory tract.

The exoccipital (Fig. 2d; Supplementary Fig. S7) is fan-shaped in posterior view and is wider than high (w/h ratio = 1.58). The exoccipital shelf is well-extended posteriorly relative to the foramen magnum in ventral view as in hadrosaurines. The supraoccipital is fused to the exoccipital and is high dorsoventrally. The basioccipital shows that the occipital condyle projects nearly horizontally.

The anterior ramus of the dentary (Fig. 2k; Supplementary Fig. S8) is deflected ventrally by 39° as in some hadrosaurines (e.g., *Prosaurolophus*, *Saurolophus*, and *Edmontosaurus*)^35–39^. The medial projection of the symphyseal process is slightly less than twice as wide as the minimum breath of the anterior ramus of the dentary posterior to the symphyseal process in dorsal view. In anterior view, the lingual curvature of the symphyseal process is gentle. In dorsal view, the angle of symphysis from the lateral surface of the anterior ramus is only 7°. An edentulous dorsal margin is slightly more than one-fifth of the length from the anterior end of the dental battery to the posterior end of the coronoid process. The preserved alveoli of the dentary are counted as 36 in a length of 28 cm, indicating the average alveolus width of 7.8 mm. The coronoid process is slightly inclined anteriorly in lateral view, and its dorsal end is expanded anteroposteriorly and semi-circular in outline. The ventral border of the dentary is bowed below the coronoid process.

The triangular-shaped splenial is as long as the surangular (Supplementary Fig. S9).

The ascending process of the surangular (Fig. 2l; Supplementary Fig. S9) projects anterodorsally and does not reach to the coronoid process of the dentary. The retroarticular process lacks an upward curvature and its lateral profile is rectangular-shaped. In dorsal view, the process is thin transversely and projects posteromedially with an angle of 144° from the medial edge of the main body of the surangular.

Both maxillary and dentary teeth are lanceolate (Fig. 2i, j; Supplementary Fig. S10), but the maxillary teeth have a blunt coronal apex, whereas the dentary teeth have a pointy apex. Tooth crown height/width ratios are 3.7 in large maxillary teeth and tend to be smaller in small teeth. The primary ridge at the middle of the crown is slightly sinuous in most teeth. The secondary ridge is absent. Marginal denticles are mostly absent, but they are small and limited to the coronal tip if present. The root is slender and is angled 150° from the enameled surface. The largest dentary tooth crown height/width ratio is 3.6 as in some lambeosaurines. The primary ridge at the middle or slightly distal to the midline of tooth crown is slightly sinuous in most teeth. Marginal denticles are absent or extremely small as in hadrosaurines other than *Gryposaurus notabilis* and are composed of one faint knob as seen only in *Edmontosaurus*. The root of the dentary teeth is angled by 130–140° from the enameled surface.

**Figure 3:**
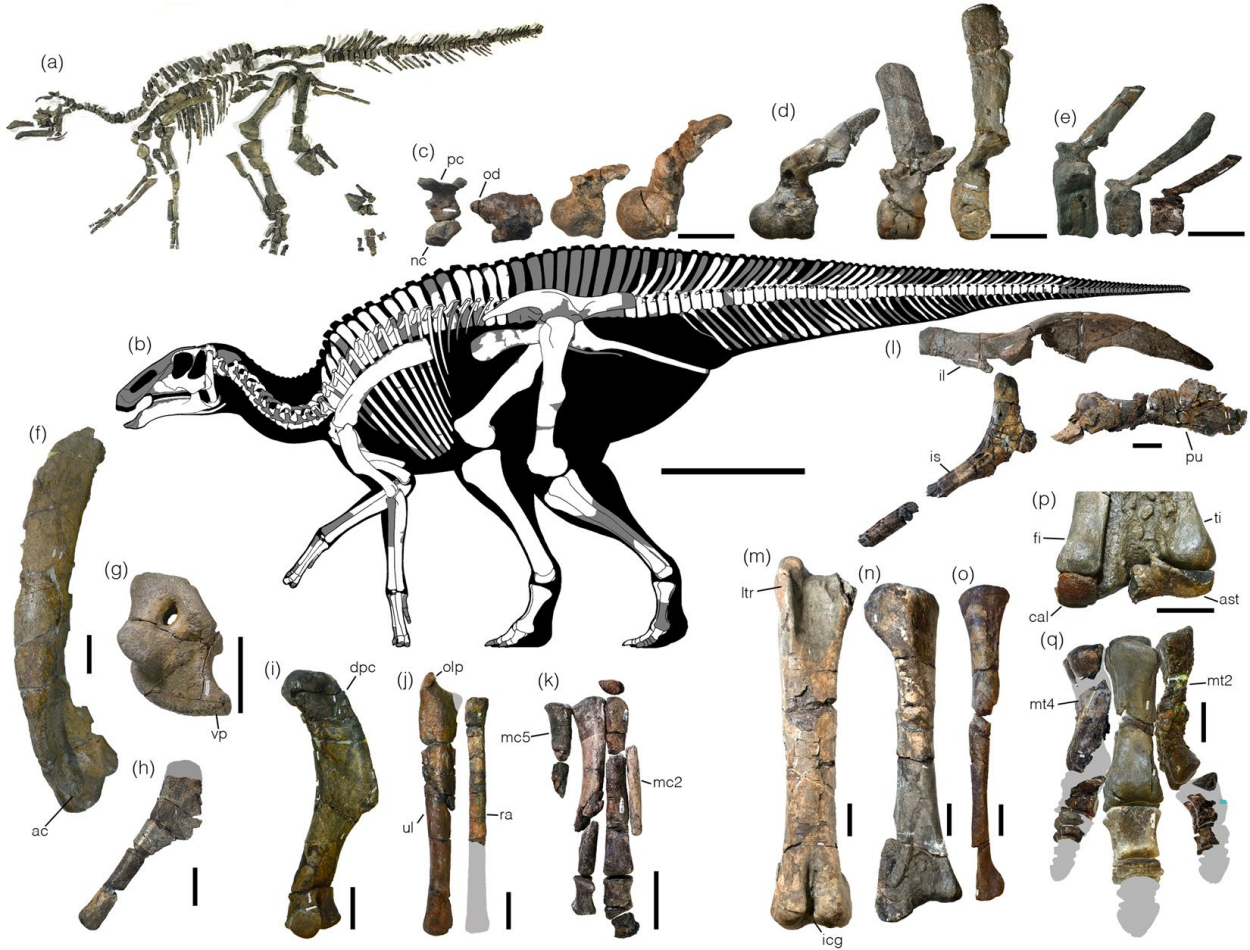
(**a**) The holotype skeleton of *Kamuysaurus japonicus* gen. et sp. nov. (**b**) Reconstructed skeleton, showing recovered elements. Selected postcranial elements: cervical vertebrae (atlas, axis, and fourth and twelfth cervicals) in left lateral view (**c**), dorsal vertebrae (first, seventh, and sixteenth dorsals) in left lateral view (**d**), caudal vertebrae (anterior, middle, and posterior caudals) in left lateral view (**e**), left scapula (**f**) and coracoid (**g**) in lateral view, right sternum in ventral view (**h**), left humerus in anterior view (i), right ulna and radius in medial view (**j**), right manus in dorsal view (**k**), right pelvis in lateral view (**l**), right femur in anterior view (**m**), right tibia in anterior view (**n**), right fibula in lateral view (**o**), right astragalus and calcaneum, articulated positioned with tibia (**p**), and right pes in dorsal view (**q**). All scales are 10 cm except 1 m scale for (**b**). Abbreviations; ac, acromion process; ast, astragalus; cal, calcaneum; dpc, deltopectoral crest; fi, fibula; icg, intercondylar groove; il, ilium; is, ischium; ltr, lesser trochanter; mc2, metacarpal II; mc5, metacarpal V; mt2, metatarsal II; mt4, metatarsal IV; nc, neurocentrum; od, odontoid; olp, olecranon process; pc, pleurocentrum; pu, pubis; ra, radius; ti, tibia; ul, ulna; vp, ventral process.

The unfused pleurocentrum of the atlas (Fig. 3c; Supplementary Fig. S11 and Table S2) have a semicircular- shaped postzygapophyseal articular surface, differing from an oval-shaped surface in *Brachylophosaurus*^33,40^. The anterior surface of the neurocentrum lacks tubercles unlike *Brachylophosaurus*^33,40^. Anteriorly projecting odontoid is dorsoventrally compressed. The intercentrum is fused to the axis, ventral to the odontoid. The axis has large and laterally projecting parapophyses and a concave posterior intervertebral surface. Eleven post-axial cervical vertebrae are opisthocoelous (Fig. 3c; Supplementary Fig. S12). The third cervical lacks the neural spine, whereas it is low in the other cervicals. Finger-like postzygapophyses form V-shaped in dorsal view with a divergent angle of roughly 90°.

Dorsal vertebrae (Fig. 3d; Supplementary Fig. S13 and Table S2) are morphologically distinguished into three segments; anterior, middle and posterior dorsal vertebrae^41^. In the anterior vertebrae, three dorsals have posteriorly tilted neural spines with a pointed dorsal tip as in *Tsintaosaurus*, *Olorotitan*, and *Magnapaulia*^42–44^ but unlike *Edmontosaurus*^41^. The neural spine is nearly vertical in the fifth dorsal and clearly inclined anteriorly in the sixth to thirteenth dorsals, which is a unique feature to *Kamuysaurus*. The neural spines of the sixteenth and seventeenth dorsals are also inclined anteriorly but the inclination is small. The first three dorsal vertebrae are opisthocoelous as in the cervical vertebrae. The outline of the middle dorsal vertebrae is heart-shaped with a weak ventral keel, whereas that of the anterior and posterior dorsal vertebrae is circular.

All caudal centra (Fig. 3e; Supplementary Fig. S14 and Table S2) are amphiplatyan. The first four caudal vertebrae lack the chevron facet as in non-lambeosaurine hadrosaurids^45^. The posterior inclination of the neural spines becomes larger posteriorly. The cone-shaped transverse processes progressively shorten posteriorly and become a horizontal low ridge from the sixteenth to forty-fifth caudals. The outline of the centrum is circular in the first four vertebrae, trapezoidal from the fifth to twenty third vertebrae, and hexagonal from the twenty-fourth to forty-fourth vertebrae. One left cervical rib, fifteen dorsal ribs from the left side, eleven ribs from the right side, and thirty chevrons are preserved (Supplementary Fig. S15).

Scapulae and coracoids from both sides and the right sternum are preserved (Fig. 3f–h; Supplementary Fig. S16 and Table S3). The scapular neck is positioned at two-fifths of the scapula length from the proximal end. As in hadrosaurines, the anterior end of the weak acromion process is straight, and the deltoid ridge is distinct. The scapular blade with a convex dorsal margin and concave ventral margin gradually expands distally as in hadrosaurines. The convex scapular facet of the coracoid is slightly larger than the concave glenoid facet as in *Secernosaurus*^46^, where most of hadrosaurids have an opposite condition. The coracoid foramen is large and oval-shaped. The biceps tubercle is well developed as a lobe-shaped process, which is separated from the ventral process by a groove, and projects lateroventrally. The ventral process is long and recurved ventrally, and the ratio of its length to the width of the base of the ventral process is 0.67.

Forelimbs are nearly complete except some elements in the manus (Fig. 3i–k; Supplementary Figs S17–S19 and Table S3). The humerus is slender, where its shaft is less than 20% of the total length. The total length is slightly shorter than the scapula. The deltopectoral crest is slightly more than half of the humerus length and is moderately expanded lateroventrally. The radial condyle is larger than the ulnar condyle. The ulna is slightly longer than humerus. The olecranon process is short and is less than 10% of the ulnar length. Both ends of the radius are semi-circular in outlines. A tetrahedral-shaped manual carpal has two large concave surfaces. All metacarpals are slender, but metacarpals III, IV, and V are thicker than metacarpal II and are subequal in length. Phalanges II-2 and III-2 of the manus are wedge-shaped. Phalanx III-1 is square-shaped in dorsal view. Phalanx III-3 is a hoof-shaped ungual phalanx. Phalanx IV-1 is about twice as long as wide. The proximal surface of phalanx V-1 is concave.

The pelvic elements are heavily damaged (Fig. 3l; Supplementary Fig. S20 and Table S3). The long preacetabular process of the ilium is gently curved and deflected ventrally. The supracetabular process is asymmetrically U-shaped and projects lateroventrally to approximately half of the iliac central plate height. The dorsal border of the central plate is strongly concave. The sacral ridge on the medial surface is positioned close to the dorsal edge of the ilium. The postacetabular process is twisted dorsomedially and moderately thickens posteriorly. The iliac peduncle of the ischium diverges proximally. The ischial shaft is straight and bears laterally convex and medially flat surfaces.

The hind limbs are nearly complete except for some of the elements in the left pes (Fig. 3m–q; Supplementary Figs S2l–24 and Table S3). The fourth trochanter at mid-length of the femur is rounded-shape. The lateral and medial distal condyles on the cranial side are fully open. The tibia is shorter than the femur. The cnemial crest projects anterolaterally. The fibula is twisted by 45° at two-thirds from the proximal end. The astragalus covers the medial malleolus of the tibia completely. The proximal surface of the calcaneum has two contact surfaces for the tibia and fibula. Metatarsal III has a length/width ratio of 4.12. Phalanx II-1 of pes is the longest and the slenderest among the phalanges. Phalanx II-2 is nearly twice as wide as long. Phalanx III-1 is the largest among phalanges and is slightly longer than wide. Phalanges III-2 and III-3 are more than three times as wide as long. Phalanx III-4 is a hoof-shaped ungual phalanx with a smooth plantar surface. Phalanx IV-1 is nearly as long as wide. Phalanges IV-2 and IV-3 are compressed proximodistally with width/length ratios of less than three.

Two large (3 cm in diameter) rounded pebbles and two small (1 cm in diameter) sub-angular pebbles were associated with the specimens, which are potentially gastroliths (Supplementary Fig. S25).

**Figure 4:**
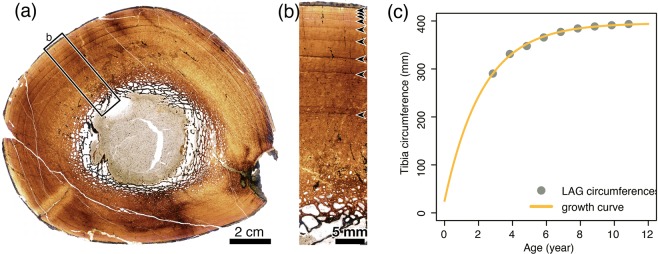
(**a**) Whole thin section image under plane polarized light. (**b**) Zoom of area indicated in (**a**), and arrows demarcate visible LAG. (**c**) Reconstructed growth curve of tibial circumference using monomolecular model.

A histological section of the mid-diaphysis of the tibia exhibits a semi-circular outline with minimal cracks suggesting the section area experienced only minimal postmortem distortion (Fig. 4a; Supplementary Fig. S26). The medullary cavity, occupying less than half of the diameter of the whole cross-section, is filled with sandy mudstone and some plant remains and is completely surrounded by sparse trabeculae composed of lamellar bone matrix. The primary bone tissue is heavily remodeled by secondary osteons and erosional cavities at the inner one-quarter of the cortex but is composed of the combination of woven and parallel-fibred bone tissues with reticular to plexiform vascular patterns where the primary bone tissue is left. There is a sharp decrease in vascular density and spaces in this area, similar to *Maiasaura*^47,48^. The outer three-fourths of the cortex are generally composed of woven and parallel-fibred bone tissues with plexiform to laminar vascular patterns, but the vascularity tends to be more laminar outwardly. In this area of the cortex, there are at least nine lines of arrested growth (LAGs; Fig. 4b). The outermost layer of the cortex becomes opaque under plane polarized light and isotropic under cross polarized light presumably due to diagenesis, and therefore the external fundamental system (EFS) cannot be observed.

## Discussion

Ontogenetic assessment of the holotype tibia based on bone microstructure and growth model fitting indicates that this individual had reached its somatic maturity. Among fitted growth models, the monomolecular model has the lowest value of Akaike information criterion (AIC), but the Von Bertalanffy and Gompertz models have **Δ**AIC values lower than 2, suggesting these three models have substantial support to be plausible growth models (Supplementary Table S5). The ages at the first LAG are estimated as 2.85, 3.8, and 4.4 years old for the aforementioned models, which means up to the second, third, and fourth growth marks are missing, respectively. Nonetheless, the last few LAGs of the outermost circumference are closely apposed and the growth curve reconstruction indicates that this individual had reached its asymptotic body size (estimated body masses of 5,296 ± 1,357 kg^49^ as a quadruped and of 4,087 ± 1,047 as a biped; estimated body length of 8 m) and no further significant growth could have been expected if the animal had continued living (Fig. 4c). The typical osteohistological features seen on adult hadrosaurs^47,48,50,51^ and the reconstructed tibial growth curve based on the LAG incremental pattern strongly indicate that this individual is an adult that reached somatic maturity even though an EFS cannot be observed.

**Figure 5:**
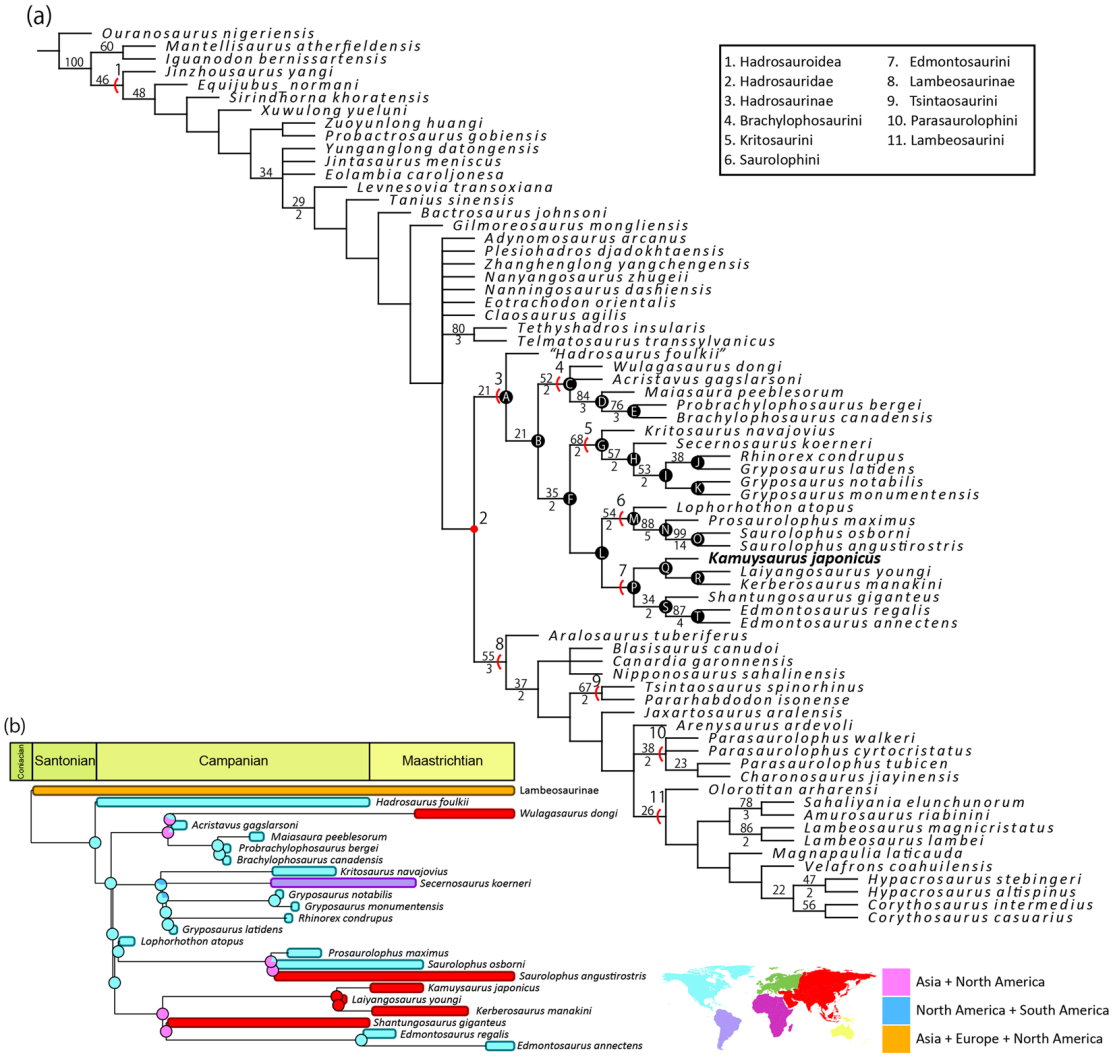
(**a**) A strict consensus tree of the most parsimonious trees obtained in this analysis. Numbers above branch lines represent bootstrap proportions, whereas those below the lines represent Bremer decay values. Bootstrap proportions lower than 20 and Bremer decay values less than 1 are not shown. Synapomorphies at each node is shown in Supplementary Text S2. (**b**) Time-calibrated cladogram with the 50% Majority Rule consensus tree of Hadrosaurinae, calculated based on additive method, showing ancestral ranges of all nodes inferred by the Dispersal Extinction Cladogenesis analysis in this study. Results using other time-calibration methods are provided in Supplementary Fig. S28.

A phylogenetic analysis produced 192 most parsimonious trees of 1139 steps (C. I. of 0.424 and R. I. of 0.830). The topology of the strict consensus tree, nearly identical to that of Xing and others^30^, shows that Hadrosaurinae consists of four major clades (Brachylophosaurini^52^, Kritosaurini^53^, Saurolophini^16^, and Edmontosaurini^54^) and *Kamuysaurus* is placed within Edmontosaurini (Fig. 5a). The clade of Saurolophini and Edmontosaurini is well supported by twelve unambiguous synapomorphies, and *Kamuysaurus* possesses four of these characters: bowed ventral margin of the coronoid process of the dentary, ventrally faced surangular, dorsal margin of the infratemporal fenestra narrower than its ventral margin, and twisted humerus. One of four unambiguous synapomorphies for the clade of Edmontosaurini (one rounded knob of marginal denticles of dentary teeth) is preserved in *Kamuysaurus*. Within the clade of Edmontosaurini, *Kamuysaurus* forms a monophyly with *Laiyangosaurus*^55^ and *Kerberosaurus*, sharing four unambiguous synapomorphies. Two of these synapomorphies (moderate ventral deflection of the rostral dentary and strong medial deflection of the caudal process of the surangular) are commonly seen in lambeosaurines, whereas a similar width of orbit and infratemporal fenestra is seen in the members of Kritosaurini, and slight ventral deflection of the preacetabular process of the ilium is a primitive feature for hadrosauroids. *Kamuysaurus* is basal to the clade of *Laiyangosaurus* and *Kerberosaurus* because these two taxa share an unambiguous synapomorphy (slightly bowed ventral margin, ventral to the coronoid process of the dentary).

*Kamuysaurus* shares some features with some sub-clades within Hadrosaurinae: slightly curved primary ridge of the maxillary teeth, anteroposteriorly expanded apex of the coronoid process of the dentary with well-developed anterior and posterior margins, nearly straight caudal margin of the quadratojugal flange of the jugal, and less expanded deltopectoral crest of the humerus with Brachylophosaurini; moderately inclined palatine articular facet of the jugal and squamosal process of the postorbital located anterior to the quadrate cotylus with Kritosaurini; and triangular-shaped ventral process with a similar height and width of the anterior process of the jugal with Brachylophosaurini and Kritosaurini. Other characters in *Kamuysaurus* appear in other hadrosaurines such as moderate medial extension of the symphyseal process of the dentary in derived Brachylophosaurini (*Brachylophosaurus* and *Probrachylophosaurus*) and *Secernosaurus* and a smoothly curved anterodorsal margin of the prefrontal in Brachylophosaurini and *Saurolophus*. Subtriangular-shaped infratemporal fenestra is an unambiguous synapomorphy for the clade of Saurolophini and Edmontosaurini, but *Kamuysaurus* reversely acquired a primitive condition (sub-rectangular-shaped infratemporal fenestra). The slender humerus is seen only in *Kamuysaurus* and *Hadrosaurus* in hadrosaurids and is a primitive condition as in non-hadrosaurid hadrosauroids.

Crested hadrosaurids such as two derived Brachylophosaurini (*Brachylophosaurus* and *Probrachylophosaurus*) and the derived Saurolophini *Saurolophus*, and lambeosaurines higher than *Aralosaurus* have the combination of two phylogenetic characters in frontal, related to the supracranial crest: presence of sutural platform of the frontal and long sutural platform. The nasofrontal sutural surface consists of the prefrontal and frontal in lambeosaurines but is formed only by the frontal in these crested hadrosaurines. Because the nasal is not preserved and the contact surface for the nasal is slightly damaged by a bio-erosion in *Kamuysaurus*, the presence of these characters in *Kamuysaurus* needs to be treated with caution; however, based on the morphology of the preserved element, the frontal of *Kamuysaurus* probably has these two characters and resembles that of crested hadrosaurines. The extension of the sutural surface (133.17 mm long) in *Kamuysaurus* is 84% of the frontal length (158.59 mm), much longer than those of *Edmontosaurus* (e.g., AMNH 427, ROM 64623) and instead similar to a sub-adult form of *Brachylophosaurus*^50^, indicating possible presence of supracranial crest in *Kamuysaurus*. Within Edmontosaurini, a trace of soft-tissue cranial crest is preserved in *Edmontosaurus*^56^, but the “bony” supracranial crest has not been reported yet, which may suggest that *Kamuysaurus* is the first Edmontosaurini with the crest and that the crest evolved convergently at least three times in the Hadrosaurinae. In *Brachylophosaurus*, the nasofrontal sutural surface enlarges as the posterior margin of the suture migrates posteriorly through ontogeny and eventually covers whole area of the frontal surface, which is rugose to stabilize the supracranial crest, in an adult form^50^. The smooth sutural surface of the frontal in *Kamuysaurus* similar to the feature in a sub-adult *Brachylophosaurus*^50^ suggests that the supracranial crest of *Kamuysaurus* is small, if present at all.

*Kamuysaurus* is the first hadrosaurine dinosaur from Japan, and all subclades contain Asian taxa: *Wulagasaurus* in Brachylophosaurini, a possible material in Kritosaurini^57^, *Saurolophus* in Saurolophini, and *Shantungosaurus*, *Laiyangosaurus*, and *Kerberosaurus* in Edmontosaurini. The Dispersal Extinction Cladogenesis (DEC) analyses with five time-calibrated 50% Majority Rule consensus trees show generally consistent results that the most probable ancestral areas for Hadrosaurinae and Saurolophini are North America, that of Brachylophosaurini is either North America or Asia + North America, and that of Kritosaurini is either North America or North and South Americas (Fig. 5b; Supplementary Fig. S28). The most recent common ancestor of Edmontosaurini was reconstructed as living in a widespread range in Asia and North America with faunal communications between these two continental areas through Beringia represented by present-day Alaska^58,59^, where Xing and others^30^ documented a high probability of the group having originated in Asia. Vicariance event of *Shantungosaurus* and *Edmontosaurus* is suggested to have probably occurred during the early Campanian. Likewise, the vicariance of the clade of *Kamuysaurus*, *Laiyangosaurus*^60^, and *Kerberosaurus* may have taken place in Asia during the late Campanian. Paleogeographically, the type localities of *Kamuysaurus*, *Laiyangosaurus*, and *Kerberosaurus* were located along the eastern edge of the Asian continent, extending approximately 15° or 1,600 km longitudinally, during the Late Cretaceous, which may suggest the endemism or provinciality of the clade in this area from the late Campanian to the early Maastrichtian.

**Figure 6:**
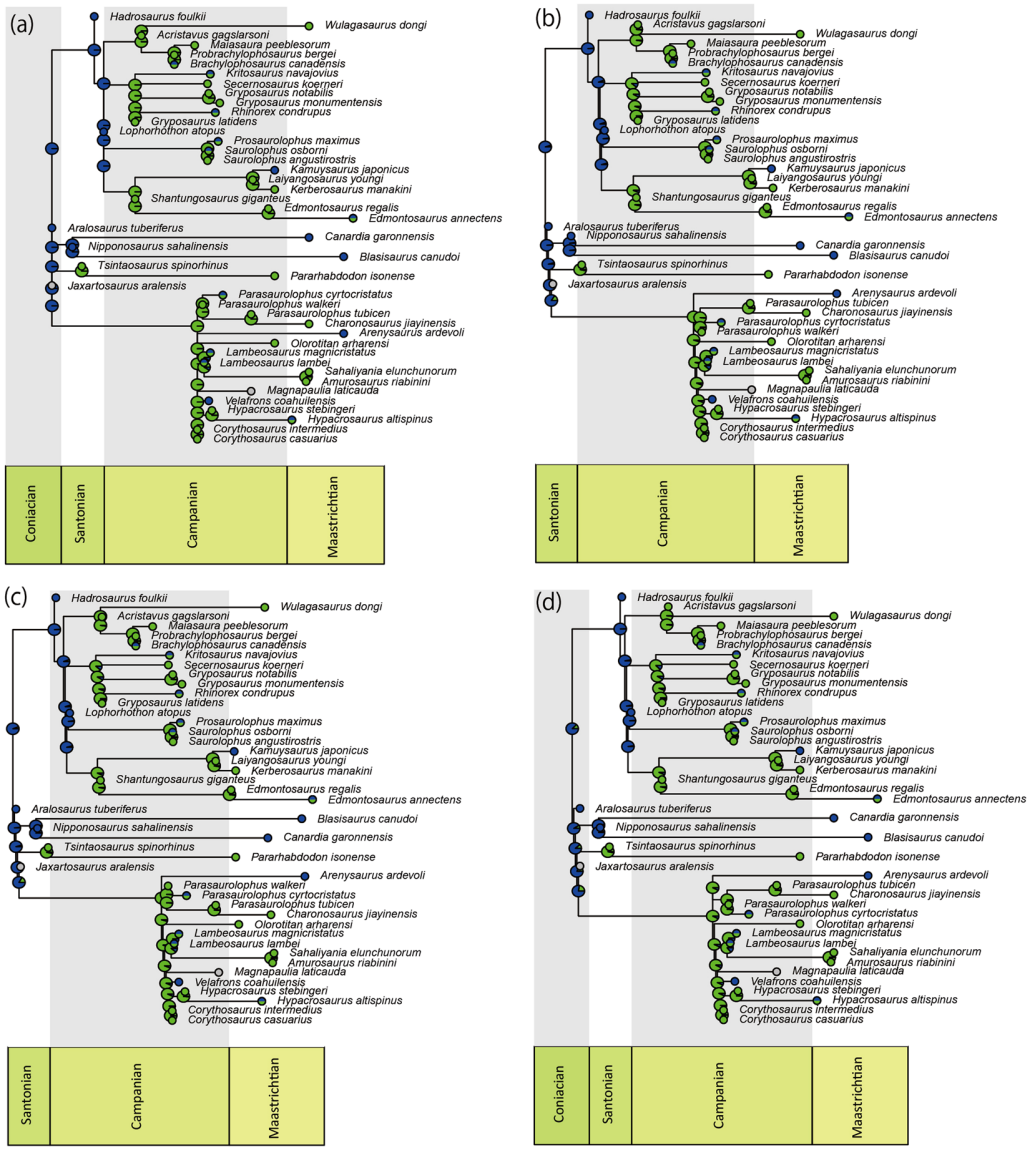
Ancestral habitat environments of all nodes inferred by the Ancestral State Reconstruction analyses based on the time-calibrated strict consensus tree using basic (**a**), additive (**b**), zero-branch lengths additive (**c**), and minimum branch lengths (**d**) methods. Colors in pie charts represent ancestral habitat environments; green as inland, blue as marginal, and grey as unknown.

**Figure 7:**
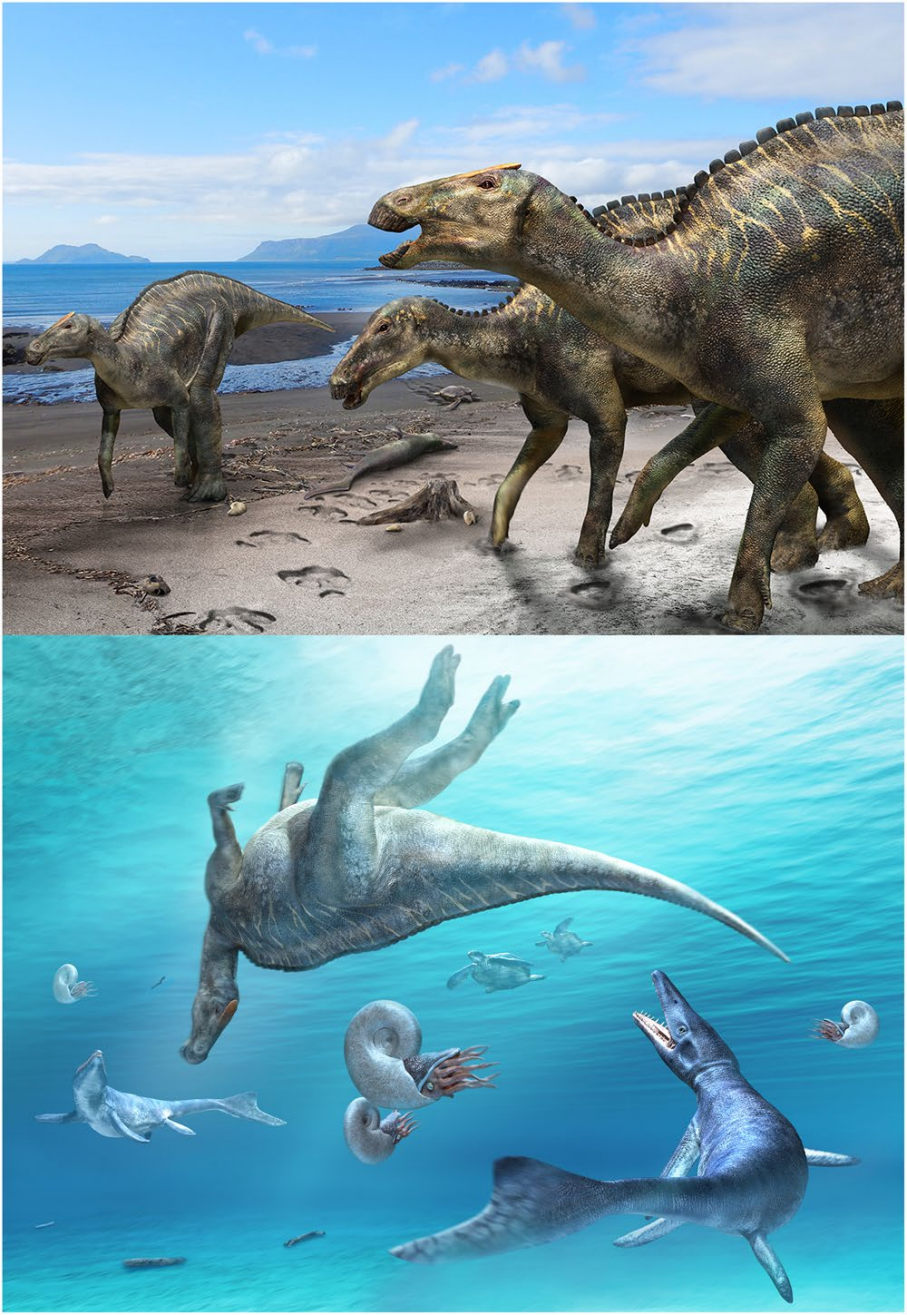
Above: Life reconstruction of *Kamuysaurus japonicus* gen. et sp. nov. with a carcass of a mosasaur (*Phosphorosaurus ponpetelegans*), a sea turtle (*Mesodermochelys undulates*), and shells of ammonoids (*Patagiosites compressu*s and *Gaudryceras hobetsense*) and bivalves (*Nannonavis elongatus*) on the beach (above). The individual of *Kamuysaurus* in the foreground is reconstructed based on the assumption of the presence of a supracranial crest, similar to a sub-adult form of *Brachylophosaurus*. The individual behind it is reconstructed without the crest. Below: Carcass of *Kamuysaurus*, floating in the sea, with two mosasaurs (*Mosasaurus hobetsuensis*), two sea turtles (*Mesodermochelys undulates*), and four ammonoids (*Pachydiscus japonicus*).

Horner^17^ reviewed dinosaur remains from marine sediments in North America and documented the richness of hadrosaurids, especially hadrosaurines (1:17 for the ratio of lambeosaurines to hadrosaurines), indicating the marginal habitat preference of hadrosaurines compared to lambeosaurines. Among the taxa used in our phylogenetic analysis includes three hadrosaurines (*Kamuysaurus*, *Hadrosaurus* and *Lophorhothon*) from marine sediments, and *Kamuysaurus* is the only taxon in the Edmontosaurini. The Ancestral State Reconstruction (ASR) analysis using the strict consensus trees by four methods for branch lengths calculations (basic, additive, zero-branch length additive, and minimum branch length) under ARD model (lowest AIC value among the three models tested; Supplementary Table S7) shows that the most probable ancestral habitat environments for the clades of Hadrosauridae, Hadrosaurinae, and Lambeosaurinae as well as the successive clades of basal hadrosaurids are marginal (Fig. 6), although ASR by the branch lengths calculations with equal method shows ancestral environments as inland for these clades. This line of evidence implies that the habitat preference of primitive hadrosaurids as well as *Kamuysaurus* (Fig. 7) may have been marine-influenced environment. Combining with the result of the DCE analysis with the 50% Majority Rule consensus tree, resulting in North America as the ancestral area for the successive clades of basal Hadrosauridae, the marine-influenced environment in North America, together with the Laramide orogeny^61^, may have played an important role for the hadrosaurid diversification in its early evolutionary history.

## Materials and Methods

### Materials

The holotype of *Kamuysaurus japonicus* (HMG-1219) used in this study includes the following elements: right maxilla, missing the anterior one-third of the medial side and anterior half of the lateral side; left maxilla, preserving middle portion of the main body; both prefrontals, missing both anterior and posterior ends; right jugal nearly complete; left jugal, preserving only anterior process; right quadrate, missing the dorsal end; left quadrate, missing its dorsal end and mandibular condyles; left pterygoid, missing the pterygoid ramus and dorsal quadrate process; left squamosal, missing the postorbital process; both postorbitals, missing tips of the squamosal and jugal processes; right frontal, missing medial portion; left frontal; portions of presphenoid-orbitosphenoid of both sides; exoccipital, missing paroccipital process; supraoccipital with a heavily eroded dorsal surface by bio-erosion; basioccipital; basisphenoid, preserving only left basipterygoid process; right dentary, missing its middle portion; left dentary, missing the posterior process; left splenial; right surangular; isolated sixty-eight maxillary and seventy-two dentary teeth; complete right and partial left ceratobranchials; atlas, missing the left pleurocentrum; axis, missing neural arch; eleven post-axial cervical vertebrae with damaged neural arches and centra; seventeen dorsal vertebrae with damaged neural arches and centra; three partial sacral vertebrae; forty-four caudal vertebrae with damaged neural arches and centra, missing more posterior caudal vertebrae; one left cervical rib; fifteen dorsal ribs from the left side and eleven ribs from right side; thirty chevrons; both scapulae; both coracoids; right sternum, missing its proximal end; both humeri; both ulnae; both radii; two distal carpals; left manus, missing metacarpal V, phalanges II-2, III-2, III-3, IV-2, IV-3, V-1, V-2, and V-3; right manus, missing phalanges II-1, II-3, IV-2, IV-3, V-1, V-2, and V-3; both ilia; both pubes; both ischia; both femora; both tibiae; both fibulae; both astragali; both calcanea; left pes, missing metatarsal II, phalanges II-3, IV-2, IV-3, IV-4, and IV-5; right pes, missing phalanges II-3, III-3, III-4, IV-4, and IV-5.

### Histology

In order to access the somatic maturity of this individual, the right tibia was thin-sectioned. Tibiae have been often used for osteohistological examination of hadrosaurs^47,48,50,51^ because they have a thick cortex and therefore preserve a more complete record of the growth. Prior to sectioning, molds and casts of the sampled tibia were created at Gobi Support Japan Co., Ltd. A thin section was made in the thin-section lab of the Faculty of Science, Hokkaido University. The transverse cross-section of the tibia was cut at the minimum circumference of the diaphysis (Supplementary Fig. S25). The mid-shaft was embedded in resin prior to mounting. After the segment was mounted on a slide, the sample was ground down to approximately 150 μm until optical clearance was obtained and growth marks were visible. We coated the slide with oil to increase the refraction index and observed the slide under both plane and cross-polarized light. The slide was imaged under plane polarized light using a Keyence digital microscope VHX-5000 in Hokkaido University Museum and an Olympus BX60 polarization microscope with a Basler Microscopy camera in Okayama University of Science. A permanent coverslip was mounted to extend the slide longevity. We generally follow terminology proposed by Francillon-Vieillot^62^ to describe the microstructure of the thin-sectioned tibia sample. General osteohistological features of the tibia resemble those previously described for adult hadrosaur tibia microstructures^47,48,50,51^, which are composed of fibrolamellar bone tissues (or woven-parallel complex^63^) with plexiform to laminar vascularity in the outer cortex and numerous secondary osteons in the inner cortex. Following Cooper and others^64^, we implemented age retrocalculation on the observed LAG incremental pattern (Supplementary Table S4) in this specimen and the growth curve reconstruction in the tibia circumference (Fig. 4c and Supplementary Fig. S27) using R ver. 3.5.2^65^ (Supplementary Data S1).

### Phylogenetic analysis

Based on the data matrix of Xing and others^30^, seven taxa (*Adynomosaurus*^66^, *Blasisaurus*^67^, *Canardia*^68^, *Laiyangosaurus*^55^, *Nipponosaurus*^13,69–71^, *Plesiohadros*^72^, and *Sahaliyania*^5^) and four characters were added (Supplementary Text S3). *Bonapartesaurus*^73^ is excluded from this analysis because of its poor preservation. Using the modified data matrix, phylogenetic analyses were conducted using TNT ver. 1.5^74^. The modified data matrix consists of 350 characters and 70 taxa (Supplementary Data S2). *Ouranosaurus nigeriensis* was selected as the outgroup. The maximum number of trees was set to 99,999 in memory. A traditional search was performed with 10,000 replicates of Wagner trees using random addition sequences, followed by the TBR branch swapping that held 10 trees per replicate. To calculate the support for the resultant trees, bootstrap resampling was performed using standard absolute frequencies with five thousand replicates, and Bremer decay indices were calculated.

### DEC analysis

In order to reconstruct ancestral ranges of hadrosaurines, Dispersal Extinction Cladogenesis (DEC)^75^ analysis is conducted using software RASP 4.1b^76^. Since a fully bifurcated tree is necessary for DEC analysis, 50% Majority Rule consensus tree was obtained from our phylogenetic result. Prior to the analysis, branch lengths were assigned using five methods (basic, equal, additive, zero-branch length additive, and minimum branch length) of timePaleoPhy function of R package paleotree 3.3.0^77^ (Supplementary Data 3–8). Maximum of two areas are allowed in ancestral distributions. Ancestral ranges of Asia + South America and Europe + South America are excluded from the analysis (Supplementary Data S9 and 10).

### Ancestral state reconstruction

Ancestral state reconstruction on habitat environments is conducted using rayDISC function of R package corHMM 1.2.2^78^ under three models (ER, SYM, and ARD). Prior to the analysis, branch lengths of the strict consensus tree were assigned using five methods (basic, equal, additive, zero-branch length additive, and minimum branch length) of timePaleoPhy function of R package paleotree 3.3.0^77^ (Supplementary Data S3 and S11–15). Habitat environments of hadrosauroids are inferred from the depositional environments compiled from published literatures (Supplementary Table S6). Habitat environments are categorized into two categories herein: inland and marginal (Supplementary Data S16). Habitat environment is assigned as inland if no sign of marine influence is recognized in the depositional environment. Marginal habitat environment is inferred by the deposits that show evidences of occasional marine influences (e.g., tidal influence, occurrences of marine organisms) and the marine deposits.

### Nomenclatural Acts

The electronic edition of this article conforms to the requirements of the amended International Code of Zoological Nomenclature, and hence the new names contained herein are available under that Code from the electronic edition of this article. This published work and the nomenclatural acts it contains have been registered in ZooBank, the online registration system for the ICZN. The ZooBank LSIDs (Life Science Identifiers) can be resolved and the associated information viewed through any standard web browser by appending the LSID to the prefix “http://zoobank.org/”. The LSID for this publication is: urn:lsid:zoobank.org:act:FC9D5C4F-AF5E-4FB5-9D45-EA4E91229BDC.

## Supplementary information

Supplementary Information

## References

[1] Weishampel, D. B. *et al*. Dinosaur distribution in *The**Dinosauria*: *Second Edition* (eds Weishampel, D. B., Dodson, P., & Osmólska, H.) 515–606 (University of California Press, 2004).

[2] Lund EK, Gates TA (2006). A historical and biogeographical examination of hadrosaurian dinosaurs. New Mexico Museum of Natural History and Science Bulletin.

[3] Xing H (2014). Comparative osteology and phylogenetic relationship of *Edmontosaurus* and *Shantungosaurus* (Dinosauria: Hadrosauridae) from the Upper Cretaceous of North America and East Asia. Acta Geologica Sinica-English Edition.

[4] Prieto-Márquez A (2010). Global phylogeny of Hadrosauridae (Dinosauria: Ornithopoda) using parsimony and Bayesian methods. Zool. J. Linn. Soc..

[5] Godefroit P, Shulin H, Tingxiang Y, Lauters P (2008). New hadrosaurid dinosaurs from the uppermost Cretaceous of northeastern China. Acta Palaeontol. Pol..

[6] Godefroit P, Zan S, Jin L (2000). *Charonosaurus jiayinensis* ng, n. sp., a lambeosaurine dinosaur from the Late Maastrichtian of northeastern China. Comptes Rendus de l’Académie des Sciences-Series IIA-Earth and Planetary Science.

[7] Bolotsky YL, Godefroit P (2004). A new hadrosaurine dinosaur from the Late Cretaceous of Far Eastern Russia. J. Vert. Paleontol..

[8] Godefroit, P., Bolotsky, Y. L. & Van Itterbeeck, J. The lambeosaurine dinosaur *Amurosaurus riabinini*, from the Maastrichtian of Far Eastern Russia. *Acta Palaeontol*. *Pol*. **49** (2004).

[9] Godefroit P, Bolotsky Y, Alifanov V (2003). A remarkable hollow-crested hadrosaur from Russia: an Asian origin for lambeosaurines. C.R. Palevol.

[10] Rozhdestvensky A (1952). A new representative of the duck-billed dinosaurs from the Upper Cretaceous deposits of Mongolia. Doklay Akademii Naukk SSSR.

[11] Maryańska T, Osmólska H (1981). First lambeosaurine dinosaur from the Nemegt Formation, Upper Cretaceous, Mongolia. Acta Palaeontol. Pol..

[12] Suzuki D, Saegusa H, Furutani H (2005). Newly found hadrosaurid fossil co-producing broadleaf fossils from Sumoto, west central Japan. J. Vert. Paleontol..

[13] Nagao T (1936). *Nipponosaurus sachalinensis*: a new genus and species of trachodont dinosaur from Japanese Saghalien. Journal of the Faculty of Science, Hokkaido Imperial University. Series 4, Geology and mineralogy.

[14] Leidy J (1858). *Hadrosaurus foulkii*, *a new saurian from the Cretaceous of New Jersey*, related to * Iguanodon*. Proc. Acad. Nat. Sci. Phila..

[15] Langston W (1960). The vertebrate fauna of the Selma Formation of Alabama: Part VI. The Dinosaurs. Fieldiana: Geology Memoirs.

[16] Prieto-Márquez A, Wagner JR, Bell PR, Chiappe LM (2014). The late-surviving ‘duck-billed’ dinosaur *Augustynolophus* from the upper Maastrichtian of western North America and crest evolution in Saurolophini. Geol. Mag..

[17] Horner, J. R. Upper Cretaceous dinosaurs from the Bearpaw Shale (marine) of south-central Montana with a checklist of Upper Cretaceous dinosaur remains from marine sediments in North America. *J*. *Paleontol*., 566-577 (1979).

[18] Takashima R (2004). *Geology* and stratigraphy of forearc basin sediments in Hokkaido, Japan: Cretaceous environmental events on the north-west Pacific margin. Cretaceous Res..

[19] Shigeta Y, Maeda H (2005). Yezo Group research in Sakhalin—a historical review. Natl. Sci. Mus. Monogr..

[20] Suzuki S (1985). A new species of *Mosasaurus* (Reptilia, Squamata) from the Upper Cretaceous Hakobuchi Group in central Hokkaido, Japan. *Evolution and Adaptaion of Marine Vertebrates*. The Monograph of the Association for the Geological Collaboration in Japan.

[21] Hirayama R, Chitoku T (1996). Family Dermochelyidae (Superfamily Chelonioidea) from the Upper Cretaceous of north Japan. Transactions and Proceedings of the Paleontological Society of Japan. New series.

[22] Konishi T, Caldwell MW, Nishimura T, Sakurai K, Tanoue K (2015). A new halisaurine mosasaur (Squamata: Halisaurinae) from Japan: the first record in the western Pacific realm and the first documented insights into binocular vision in mosasaurs. J. Syst. Palaeontol..

[23] Matsumoto, T. Selected Cretaceous leading ammonites in Hokkaido and Saghalien. *The Cretaceous System in the Japanese Islands*, 243–324 (1954).

[24] Shigeta Y, Nishimura T (2013). A new species of *Gaudryceras* (Ammonoidea, Gaudryceratidae) from the lowest Maastrichtian of Hokkaido, Japan and its biostratigraphic implications. Paleontol. Res..

[25] Owen R (1842). Report on British fossil reptiles, Part 2. Report of the British Association for the Advancement of Science.

[26] Seeley HG (1887). On the classification of the fossil animals commonly named Dinosauria. Proceedings of the Royal Society of London.

[27] Marsh OC (1881). Principal characters of American Jurassic dinosaurs, part IV. Am. J. Sci..

[28] Cope ED (1870). Synopsis of the extinct Batrachia, Reptilia and Aves of North America. Trans. Am. Philos. Soc..

[29] Lambe LM (1918). On the genus *Trachodon* of Leidy. Ottawa Naturalist.

[30] Xing H, Mallon JC, Currie ML (2017). Supplementary cranial description of the types of *Edmontosaurus regalis* (Ornithischia: Hadrosauridae), with comments on the phylogenetics and biogeography of Hadrosaurinae. PLoS ONE.

[31] Lambe LMO (1914). *Gryposaurus notabilis*, a new genus and species of trachodont dinosaur from the Belly River Formation of Alberta, with a description of the skull of *Chasmosaurus belli*. Ottawa Naturalist.

[32] Gates TA, Farke AA (2009). Biostratigraphic and biogeographic implications of a hadrosaurid (Ornithopoda: Dinosauria) from the Upper Cretaceous Almond Formation of Wyoming, USA. Cretaceous Res..

[33] Cuthbertson RS, Holmes RB (2010). The first complete description of the holotype of *Brachylophosaurus canadensis* Sternberg, 1953 (Dinosauria: Hadrosauridae) with comments on intraspecific variation. Zool. J. Linn. Soc..

[34] Brown B, Pepper GH (1910). The Cretaceous Ojo Alamo Beds of New Mexico: with description of the new dinosaur genus. Kritosaurus. Bull. Am. Mus. Nat. Hist..

[35] Horner JR (1992). Cranial morphology of *Prosaurolophus* (Ornithischia: Hadrosauridae) with descriptions of two new hadrosaurid species and an evaluation of hadrosaurid phylogenetic relationships. Museum of the Rockies Occasional Paper.

[36] Bell PR (2011). Cranial osteology and ontogeny of *Saurolophus angustirostris* from the late Cretaceous of Mongolia with comments on *Saurolophus osborni* from Canada. Acta Palaeontol. Pol..

[37] McGarrity CT, Campione NE, Evans DC (2013). Cranial anatomy and variation in *Prosaurolophus maximus* (Dinosauria: Hadrosauridae). Zool. J. Linn. Soc..

[38] Bell PR (2011). Redescription of the skull of *Saurolophus osborni* Brown 1912 (Ornithischia: Hadrosauridae). Cretaceous Res..

[39] Maryańska T, Osmólska H (1981). Cranial anatomy of *Saurolophus angustirostris* with comments on the Asian Hadrosauridae (Dinosauria). Palaeontol. Pol..

[40] Prieto-Márquez A (2005). New information on the cranium of Brachylophosaurus canadensis(Dinosauria, Hadrosauridae), with a revision of its phylogenetic position. J. Vert. Paleontol..

[41] Campione, N. E. Postcranial anatomy of *Edmontosaurus regalis* (Hadrosauridae) from the Horseshoe Canyon Formation, Alberta, Canada in *Hadrosaurs* (eds Eberth, D. A. & Evans, D. C.) 208–244 (Indiana University Press, 2015).

[42] Young C-C (1958). The dinosaurian remains of Laiyang, Shantung. Palaeontologica Sinica.

[43] Godefroit P, Bolotsky YL, Bolotsky IY (2012). Osteology and relationships of *Olorotitan arharensis*, a hollow-crested hadrosaurid dinosaur from the latest Cretaceous of Far Eastern Russia. Acta Palaeontol. Pol..

[44] Prieto-Márquez A, Chiappe LM, Joshi SH (2012). The lambeosaurine dinosaur *Magnapaulia laticaudus* from the late cretaceous of Baja California, Northwestern Mexico. PLoS ONE.

[45] Horner, J. R., Weishampel, D. B. & Forster, C. A. Hadrosauridae in *The**Dinosauria**: Second Edition* (eds Weishampel, D. B., Dodson, P., & Osmólska, H.) 438–463 (University of California Press, 2004).

[46] Prieto-Márquez A, Salinas GC (2010). A re-evaluation of *Secernosaurus koerneri* and *Kritosaurus australis* (Dinosauria, Hadrosauridae) from the Late Cretaceous of Argentina. J. Vert. Paleontol..

[47] Horner JR, De Ricqlès A, Padian K (2000). Long bone histology of the hadrosaurid dinosaur *Maiasaura peeblesorum*: growth dynamics and physiology based on an ontogenetic series of skeletal elements. J. Vert. Paleontol..

[48] Woodward HN, Freedman Fowler EA, Farlow JO, Horner JR (2015). *Maiasaura*, a model organism for extinct vertebrate population biology: a large sample statistical assessment of growth dynamics and survivorship. Paleobiology.

[49] Campione NE, Evans DC (2012). A universal scaling relationship between body mass and proximal limb bone dimensions in quadrupedal terrestrial tetrapods. BMC Biol..

[50] Freedman Fowler EA, Horner JR (2015). A new brachylophosaurin hadrosaur (Dinosauria: Ornithischia) with an intermediate nasal crest from the Campanian Judith River Formation of northcentral Montana. PLoS ONE.

[51] Fondevilla V (2018). Ontogeny and taxonomy of the hadrosaur (Dinosauria, Ornithopoda) remains from Basturs Poble bonebed (late early Maastrichtian, Tremp Syncline, Spain). PLoS ONE.

[52] Gates TA, Horner JR, Hanna RR, Nelson CR (2011). New unadorned hadrosaurine hadrosaurid (Dinosauria, Ornithopoda) from the Campanian of North America. J. Vert. Paleontol..

[53] Lapparent AF, Lavocat R (1955). Dinosauriens. Traité de paléontologie.

[54] Brett-Surman, M. K. *A Revision of the Hadrosauridae (Reptilia: Ornithischia) and Their Evolution During the Campanian and Maastrichtian* PhD thesis, George Washington University (1989).

[55] Zhang J (2017). A new saurolophine hadrosaurid (Dinosauria: Ornithopoda) from the Upper Cretaceous of Shandong, China. An. Acad. Bras. Cienc..

[56] Bell PR, Fanti F, Currie PJ, Arbour VM (2014). A mummified duck-billed dinosaur with a soft-tissue cock’s comb. Curr. Biol..

[57] Zhang, Y. G., Wang, K. B., Chen, S. Q., Liu, D. & Xing, H. Osteological re-assessment and taxonomic revision of “*Tanius laiyangensis*” (Ornithischia: Hadrosauroidea) from the Upper Cretaceous of Shandong, China. *Anat Rec (Hoboken)*, 10.1002/ar.24097 (2019). 10.1002/ar.24097 30773831

[58] Fiorillo AR (2008). Dinosaurs of Alaska: Implications for the Cretaceous origin of Beringia. Geological Society of America Special Papers.

[59] Fiorillo AR (2018). An unusual association of hadrosaur and therizinosaur tracks within Late Cretaceous rocks of Denali National Park, Alaska. Sci. Rep..

[60] Liu Y (2010). Sedimentary facies and taphonomy of Late Cretaceous deaths of dinosaur, Zhucheng, eastern Shandong. Geological Review.

[61] Gates TA, Prieto-Marquez A, Zanno LE (2012). Mountain building triggered late Cretaceous North American megaherbivore dinosaur radiation. PLoS ONE.

[62] Francillon-Vieillot, H. *et al*. Microstructure and mineralization of vertebrate skeletal tissues in *Skeletal Biomineralization: Patterns*, *Processes and Evolutionary**Trends* (ed. Carter, J. G.) 471–530 (Springer US, 1991).

[63] Prondvai E, Stein KHW, de Ricqlès A, Cubo J (2014). Development-based revision of bone tissue classification: the importance of semantics for science. Biol. J. Linn. Soc..

[64] Cooper LN, Lee AH, Taper ML, Horner JR (2008). Relative growth rates of predator and prey dinosaurs reflect effects of predation. Proc. R. Soc. Biol. Sci. Ser. B.

[65] R Core Team. R: A Language and environment for statistical computing, https://www.R-project.org/ (R Foundation for Statistical Computing, Vienna, Austria, 2018).

[66] Prieto-Márquez A, Fondevilla V, Sellés AG, Wagner JR, Galobart À (2019). *Adynomosaurus arcanus*, a new lambeosaurine dinosaur from the Late Cretaceous Ibero-Armorican Island of the European archipelago. Cretaceous Res..

[67] Cruzado-Caballero P, Pereda-Suberbiola X, Ruiz-Omeñaca JI (2010). *Blasisaurus canudoi* gen. et sp. nov., a new lambeosaurine dinosaur (Hadrosauridae) from the latest Cretaceous of Arén (Huesca, Spain). Can. J. Earth Sci..

[68] Prieto-Márquez A, Dalla Vecchia FM, Gaete R, Galobart A (2013). Diversity, relationships, and biogeography of the lambeosaurine dinosaurs from the European Archipelago, with description of the new aralosaurin *Canardia garonnensis*. PLoS ONE.

[69] Takasaki R, Chiba K, Kobayashi Y, Currie PJ, Fiorillo AR (2017). Reanalysis of the phylogenetic status of *Nipponosaurus sachalinensis* (Ornithopoda: Dinosauria) from the Late Cretaceous of Southern Sakhalin. Hist. Biol..

[70] Suzuki D, Weishampel DB, Minoura N (2004). *Nipponosaurus sachalinensis* (Dinosauria; Ornithopoda): anatomy and systematic position within Hadrosauridae. J. Vert. Paleontol..

[71] Nagao T (1938). On the limb-bones of *Nipponosaurus sachalinensis* Nagao, a Japanese hadrosaurian dinosaur. Annotationes zoologicae Japonenses.

[72] Tsogtbaatar, K., Weishampel, D. B., Evans, D. C. & Watabe, M. A new hadrosauroid (*Plesiohadros djadokhtaensis*) from the Late Cretaceous djadokhtan fauna of southern Mongolia in *Hadrosaurs* (eds Eberth, D. A. & Evans, D. C.) 108–135 (Indiana University Press, 2015).

[73] Cruzado-Caballero P, Powell J (2017). *Bonapartesaurus rionegrensis*, a new hadrosaurine dinosaur from South America: implications for phylogenetic and biogeographic relations with North America. J. Vert. Paleontol..

[74] Goloboff PA, Catalano SA (2016). TNT version 1.5, including a full implementation of phylogenetic morphometrics. Cladistics.

[75] Ree RH, Smith SA (2008). Maximum likelihood inference of geographic range evolution by dispersal, local extinction, and cladogenesis. Syst. Biol..

[76] Yu Y, Harris AJ, Blair C, He X (2015). RASP (Reconstruct Ancestral State in Phylogenies): a tool for historical biogeography. Mol. Phylogen. Evol..

[77] Bapst D (2012). W. paleotree: an R package for paleontological and phylogenetic analyses of evolution. Methods in Ecology and Evolution.

[78] Beaulieu, J. M., Oliver, J. C. & O’Meara, B. C. corHMM: Analysis of Binary Character Evolution, https://CRAN.R-project.org/package=corHMM (2017).

