# Author Correction: A New Hadrosaurine (Dinosauria: Hadrosauridae) from the Marine Deposits of the Late Cretaceous Hakobuchi Formation, Yezo Group, Japan

**DOI:** 10.1038/s41598-019-53861-4

**Published:** 2019-11-20

**Authors:** Yoshitsugu Kobayashi, Tomohiro Nishimura, Ryuji Takasaki, Kentaro Chiba, Anthony R. Fiorillo, Kohei Tanaka, Tsogtbaatar Chinzorig, Tamaki Sato, Kazuhiko Sakurai

**Affiliations:** 1 Hokkaido University Museum, Hokkaido University, Sapporo, Hokkaido, 060-0810 Japan; 2 Hobetsu Museum, Mukawa, Hokkaido, 054-0211 Japan; 3 Department of Natural History and Planetary Sciences, Hokkaido University, Sapporo, Hokkaido, 060-0810 Japan; 4 Faculty of Biosphere-Geosphere Science, Okayama University of Science, Okayama, 700-0005 Japan; 5 Perot Museum of Nature and Science, Dallas, Texas 75201 United States; 6 Graduate School of Life and Environmental Sciences, University of Tsukuba, Tsukuba, Ibaraki, 305-8572 Japan; 7 Division of Vertebrate Paleontology, Institute of Paleontology and Geology, Mongolian Academy of Sciences, Ulaanbaatar, 15160 Mongolia; 8 Department of Astronomy and Earth Sciences, Tokyo Gakugei University, Koganei, Tokyo, 184-8501 Japan

Correction to: *Scientific Reports* 10.1038/s41598-019-48607-1, published online 05 September 2019

The original version of this Article did not meet the requirement of the amendment of Article 8.5.3 of the International Code of Zoological Nomenclature^1^. To fully meet the amended provisions of the Code, the following Nomenclatural Acts have been added to the Materials and Methods section:

## Nomenclatural Acts.

The electronic edition of this article conforms to the requirements of the amended International Code of Zoological Nomenclature, and hence the new names contained herein are available under that Code from the electronic edition of this article. This published work and the nomenclatural acts it contains have been registered in ZooBank, the online registration system for the ICZN. The ZooBank LSIDs (Life Science Identifiers) can be resolved and the associated information viewed through any standard web browser by appending the LSID to the prefix “http://zoobank.org/”. The LSID for this publication is: urn:lsid:zoobank.org:act:FC9D5C4F-AF5E-4FB5-9D45-EA4E91229BDC.
