# Supplementary material for: A New Hadrosaurine (Dinosauria: Hadrosauridae) from the Marine Deposits of the Late Cretaceous Hakobuchi Formation, Yezo Group, Japan
Source: Sci Rep. 2019 Sep 5;9:12389. doi: 10.1038/s41598-019-48607-1 (PMC6728324; doi:10.1038/s41598-019-48607-1)
Supplement: Supplementary file 1 — Supplementary Information [file 41598_2019_48607_MOESM1_ESM.docx]

Supplementary information for “A NEW HADROSAURINE (DINOSAURIA: HADROSAURIDAE) FROM THE MARINE DEPOSITS OF THE LATE CRETACEOUS HAKOBUCHI FORMATION, YEZO GROUP, JAPAN” by Yoshitsugu Kobayashi, Tomohiro Nishimura, Ryuji Takasaki, Kentaro Chiba, Anthony R. Fiorillo, Kohei Tanaka, Tsogtbaatar Chinzorig, Tamaki Sato, and Kazuhiko Sakurai

# ­­Supplementary Text

## Supplementary Text S1: Additional information of the geology of the Hakobuchi Formation.

The Hakobuchi Formation is uppermost part of the Yezo Group, which consists of the Cretaceous to Paleocene forearc basin deposits^1,2^, is distributed in the meridional region of Hokkaido, northern Japan (Fig. 1 and Supplementary Figure S1)^3,4^. It mainly consists of sandstone and conglomerate and is rich in molluscan and marine vertebrate fossils^3,5-7^. The formation crops out in Hobetsu area of Mukawa town and its adjacent areas. The lithostratigraphy and biostratigraphy of the Hakobuchi Formation are well studied in southern Hobetsu area (Tomiuchi or Hetonai)^8,9^. On the other hand, those of the northern Hobetsu area (Inasato), where the hadrosaurid dinosaur was excavated, are poorly understood; however, this study reveals that the lithostratigraphic subdivision of the formation of the southern Hobetsu area can be applied to the northern Hobetsu area. In addition, common index fossils of ammonoids and inoceramid bivalves occur from both areas.

The Hakobuchi Formation is sub-divided into five rock units (IVa-IVe), and *Kamuysaurus* occurs from a large outcrop of the middle part of IVb unit, consisting of mainly sandy mudstone, of the Hakobuchi Formation at upstream of Shirafunezawa Creek, northern Hobetsu area (Fig 1C, Supplementary Figure S1; 42^o^50’48”N, 142^o^7’20”E). IVb unit is correlated to the *Nostoceras hetonaiense* ammonoid zone (Fig. 1, Supplementary Figure S2), which contains multiple ammonoid taxa including *Pachydiscus* (*Neodesmocereas*) *japonicus*^10^, *Nostoceras hetonaiense*^11^, and *Gaudryceras hobetsense*^12^. At the *Kamuysaurus* locality, *P*. (*N*.) *japonicus* was recovered from 5 meters below and 3 meters above the *Kamuysaurus*-bearing horizon (Supplementary Figure S1). In addition to *P*. (*N*.) *japonicus,* some marine bivalves (e.g., *Nannonavis*, and *Nucula* and *Inoceramus*), gastropods, shark teeth, and fish scales were found.

The marine deposits from the upper Campanian to lower Maastrichtian in Japan are subdivided into six biostratigraphic zones based on ammonoids, and the *Nostoceras hetonaiense* zone is the second youngest zone. The *Nostoceras hetonaiense* zone of the Kita-ama Formation in Awaji Island, southwest Japan, is magnetostratigraphically correlated to between chron 32.2n to 32.1n, which is the uppermost Campanian to lowest Maastrichtian^13^. In previous studies, the base of *Nostoceras hetonaiense* zone was defined as the Campanian—Maastrichtian boundary^14^, suggesting that the hadrosaurid bearing horizon is dated as the Maastrichtian in age.

The lower part of the second oldest zone, the *Didymoceras awajiense* zone, was radiometrically (U–Pb) dated as 72.4±0.8 Ma, whereas the upper part of the youngest zone, *Garudyceras izumiense* zone, or more above horizon of *G*. *izumiese* zone was dated as 70.6±1.2 Ma^15,16^. These radiometric dates confirm that the age of the *Nostoceras hetonaiense* zone is between 72.4±0.8 Ma and 70.6±1.2 Ma but still leaves a possibility that this zone is older than 72.1 Ma because there is no study on radiometric dating for either the *Nostoceras hetonaiense* or *Pachydiscus awajiensis* zone. Therefore, further study is needed to refine the age of the hadrosaurid bearing horizon, but this study considers the lowest Maastrichtian for the age of the *Nostoceras hetonaiense* zone until then.

A semi-articulated, nearly complete skeleton of *Kamuysaurus* was excavated in an area of 4 m x 7 m (Supplementary Figure S1). Despite of its completeness, some bones such as centra of dorsal vertebrae are heavily damaged and surfaces of some bones were eroded by a bio-erosion before it was buried.

**References for Supplementary Text S1**

1 Okada, H. Collision orogenesis and sedimentation in Hokkaido, Japan in *Accretion techtonics in the circum-pacific regions* (eds. Hashimoto, M. & Ueda, S.) 91-105 (Terra Scientific Publishing Co., 1983).

2 Takashima, R. *et al.* Geology and stratigraphy of forearc basin sediments in Hokkaido, Japan: Cretaceous environmental events on the north-west Pacific margin. *Cretaceous Res.* **25**, 365-390, doi:10.1016/j.cretres.2004.02.004 (2004).

3 Matsumoto, T. Selected Cretaceous leading ammonites in Hokkaido and Saghalien. *The Cretaceous System in the Japanese Islands*, 243-324 (1954).

4 Ando, H. & Tomosugi, T. Unconformity between the Upper Maastrichtian and Upper Paleocene in the Hakobuchi Formation, north Hokkaido, Japan: a major time gap within the Yezo forearc basin sediments. *Cretaceous Res.* **26**, 85-95, doi:10.1016/j.cretres.2004.11.001 (2005).

5 Suzuki, S. A new species of *Mosasaurus* (Reptilia, Squamata) from the Upper Cretaceous Hakobuchi Group in central Hokkaido, Japan. *Evolution and Adaptaion of Marine Vertebrates. The Monograph of the Association for the Geological Collaboration in Japan* **30**, 45-66 (1985).

6 Hirayama, R. & Chitoku, T. Family Dermochelyidae (Superfamily Chelonioidea) from the Upper Cretaceous of north Japan. *Transactions and Proceedings of the Paleontological Society of Japan. New series* **184**, 597-622, doi:10.14825/prpsj1951.1996.184_597 (1996).

7 Konishi, T., Caldwell, M. W., Nishimura, T., Sakurai, K. & Tanoue, K. A new halisaurine mosasaur (Squamata: Halisaurinae) from Japan: the first record in the western Pacific realm and the first documented insights into binocular vision in mosasaurs. *J. Syst. Palaeontol.* **14**, 809-839, doi:10.1080/14772019.2015.1113447 (2015).

8 Matsumoto, T. Fundamentals in the Cretaceous stratigraphy of Japan. Part I. *Mem. Fac. Sci., Kyushu Imp. Univ. Ser. D* **1**, 129-280 (1942).

9 Tanaka, K. Cretaceous deposits in the Tomiuchi district, southern Central Hokkaido. *Bull. Geol. Surv. Jpn.* **11**, 543-554 (1960).

10 Matsumoto, T. A note on the Japanese Pachydiscidae. *Sci. Rep. Fac. Sci. Kyushu. Univ. Geol.* **2**, 34-46 (1947).

11 Matsumoto, T. Some heteromorph ammonites from the Cretaceous of Hokkaido. *Mem. Fac. Sci. Kyushu Univ. Ser. D Geol.* **23**, 303-366 (1977).

12 Shigeta, Y. & Nishimura, T. A new species of *Gaudryceras* (Ammonoidea, Gaudryceratidae) from the lowest Maastrichtian of Hokkaido, Japan and its biostratigraphic implications. *Paleontol. Res.* **17**, 47-57, doi:10.2517/1342-8144-17.1.47 (2013).

13 Kodama, K. Magnetostratigraphy of the Izumi Group along the Median Tectonic Line in Shikoku and Awaji Islands, Southwest Japan. *J. Geol. Soc. Jpn.* **96**, 265-278 (1990).

14 Morozumi, Y. Late Cretaceous (Campanian and Maastrichtian) ammonites from Awaji Island, Southwest Japan. *Bull. Osaka Mus. Nat. Hist.* **39**, 1-58 (1985).

15 Shigeta, Y., Tsutsumi, Y. & Misaki, A. U–Pb age of the *Didymoceras awajiense* Zone (upper Campanian, Cretaceous) in the Aridagawa area, Wakayama, southwestern Japan. *Bull. Natl. Mus. Nat. Sci. Ser. C, Geol. & Paleontol.* **43**, 11-18 (2017).

16 Shigeta, Y., Izukura, M. & Tsutsumi, Y. An early Maastrichtian (latest Cretaceous) ammonoid fauna from the Soya Hill area, Hokkaido, northern Japan. *Bull. Hobetsu Mus.* **32**, 7-41 (2017).

## Supplementary Text S2: Synapomorphies of nodes and autapomorphies of terminal taxa within Hadrosaurinae, shown in Figure 5 in the main text.

**Clade A (Hadrosaurinae)**

6 (3): Enameled surface of the dentary teeth bears only the primary ridge

11 (2): Small marginal denticles of dentary teeth

296 (1): Absence of the ridge connecting the caudoventral margin of the supraacetabular process and the dorsal margin of the postacetabular process of ilium

325 (1): Craniodorsal corner of the ischial pubic peduncle located dorsal to the dorsal margin of the ischial shaft.

**Clade B**

275 (1): Ulna/humerus ratio in between 1.0 and 1.2.

**Clade C (Brachylophosaurini)**

22 (1): Slightly curved primary ridge of maxillary tooth

75 (1): Triangular rostrolateral corner of the premaxillary oral margin.

109 (1): The maxilla-lacrimal contact is covered by the jugal and not exposed laterally.

144 (1): Anterior process of the nasal is thick, subrectangular, and abruptly decreases the depth in the anterior end.

148 (0): Absence of the paired process at the frontonasal suture on the nasal.

150 (1): Convex and arcuate dorsal margin of rostrum dorsal to the external naris.

157 (0): Elliptic and dorsoventrally broad narial foramen.

216 (1): Well-developed alar process of the basisphenoid.

219 (2): Maximum width across the sphenoccipital tubercles/minimum width of the rostral constriction greater than 1.9.

232 (0): Maximum mediolateral width of the braincase across the postorbitals/the mediolateral width of the occiput across the quadrate cotyli of the squamosals more than 1.25.

270 (0): Width of the deltopectral crest relative to the humeral shaft less than 1.65

295 (0): Dorsoventral height/anteroposterior length of iliac central plate greater than 0.8.

**Clade D**

80 (1): Presence of at least one oval foramen on the anterodorsal surface of the premaxillary lip.

112 (2): Slightly inclined ectopterygoid shelf of maxilla (5º- 10º)

133 (0): Presence of the squamosal buttress of the quadrate.

138 (1): Presence of solid supracranial crest without lateral excavation of circumnarial fossa.

147 (2): Anterior end of the nasal reaches the anterior margin of the external naris.

175 (1): Dorsolaterally flaring anterodorsal margin of the prefrontal.

193 (1): The nasal contact surface of the frontal/total length of the frontal over 0.4.

288 (1): Anteroposterior length of the preacetabular process/anteroposterior length of the central plate of the ilium greater than 1.7.

**Clade E**

43 (1): Mediolateral width of dentary symphyseal region/minimum breadth of the dentary caudal to the symphyseal process in between 1.65 and 2.60.

191 (1): Presence of the frontal platform for supporting the supracranial crest.

195 (1): Presence of the median cleft on the nasal articular surface of the frontal.

198 (1): Anteroposterior length of the interfrontal suture/width of the frontal in between 0.4 and 0.8.

200 (2): Absence of the nasal-frontal fontanelle.

**Clade F**

4 (3): Three or more functional teeth on the dentary.

49 (2): Well-developed coronoid process of the dentary, posterior margin more pronounced than the anterior margin.

74 (3): Thin and strongly deflected premaxillary oral margin slightly recurved anterior to the premaxillary accessory fossa.

97 (1): Ventrally curved anteroventral process of the maxilla.

112 (3): Nearly horizontal ectopterygoid shelf of maxilla (less than 5º)

123 (2): Convex posterior margin of the jugal with pointed dorsal apex.

170 (2): Triangular, anteroposteriorly elongated lacrimal, with straight and dorsoventrally thick rostral process.

173 (1): Nearly right-angled anterodorsal margin of the prefrontal along the orbital rim.

271 (2): Angle between the anterolateral and ventral margins of the humeral deltopectoral crest less than 110º

341 (1): Length/width of the metatarsal III up to 4.5

**Clade G (Kritosaurini)**

39 (0): Edentulous slope of dentary is angled less than 150º

98 (2): Dorsal and ventral margins of the anteroventral process of the maxilla greater than 42 º

119 (1): Palatine articular surface of the jugal inclined in between 110 º and 130 º.

125 (1): Width of jugal at the posterior contraction/distance between the ventral extremities of infratemporal fenestra and the orbital rim: in between 0.6 and 0.8.

133 (0): Presence of the squamosal buttress of the quadrate.

142 (2): Nasal forming transversely narrow, arched promontory.

186 (0): Posterior end of the squamosal process of the postorbital located anterior to the quadrate cotylus.

226 (1): Dorsal margin of the orbit located lower than that of the infratemporal fenestra.

**Clade H**

100 (3): Regular diamond-shaped jugal articular surface located at the midline of the maxilla

148 (2): Posterior end of the nasal forms a pair of conjunctive processes inserted in between the frontals

**Clade I**

17 (0): Less than 1.25 maxillary teeth/cm.

38 (2): Length of the dentary edentulous slope/length from the anterior alveolus posterior margin of the coronoid process: in between 0.20 and 0.31.

300 (2): Postacetabular process length/central plate length: 1.1 or greater.

**Clade J**

2 (0): Less than 0.7 dentary tooth/cm.

8 (1): Slightly curved primary ridge of dentary tooth crown.

**Clade K**

62 (2): Surangular facing more ventrally than laterally.

72 (1): Maximum premaxillary width/width of the posterior contractive region: in between 1.70 and 2.00.

**Clade L**

16 (2): Presence of more than 45 maxillary dental alveoli.

47 (1): Well-developed ventral margin of the dentary ventral to the coronoid process.

62 (2): Surangular facing more ventrally than laterally.

117 (0): Bowed ventral margin of the jugal anterior process.

144 (2): Long, finger-shaped anterior end of the nasal without prominent change in depth.

145 (2): Greatly shortened hook-like anteroventral process of the nasal.

147 (2): Anterior end of the nasal reaches the anterior margin of the external naris.

161 (2): Circumnarial fossa deeply incised into nasal and premaxilla.

227 (1): Subtriangular infratemporal fenestra.

228 (1): Dorsal margin of the infratemporal fenestra narrower than the ventral margin.

273 (1): Strongly twisted humerus.

320 (2): Dorsoventral height of the iliac peduncle of the ischium more than twice as long as its length at the anterodorsal margin.

**Clade M (Saurolophini)**

121 (0): Shallow and wide concave ventral margin of the jugal.

132 (2): Wide arcuate and anteroposteriorly shallow quadratojugal notch of the quadrate which is slightly recurved and oriented rostrally.

142 (6): Nasal forming posterodorsally elevated, laterodorsally depressed, and transversely broad nasal crest.

**Clade N**

138 (2): Presence of solid supracranial crest which is laterally excavated by posterior expansion of the circumnarial fossa.

139 (1): Presence of supracranial crest composed of nasals.

183 (1): Short and robust squamosal process of the postorbital.

197 (1): Exclusion of frontal from the orbital rim

198 (2): Anteroposterior length of the interfrontal suture/width of the frontal smaller than 0.4.

343 (1): Proximodistally compressed pedal phalanges III-2 and III-3.

344 (1): Proximodistally compressed pedal phalanges IV-2, IV-3, and IV-4.

**Clade O**

72 (1): Maximum premaxillary width/width of the posterior contractive region: in between 1.70 and 2.00.

84 (1): Posterior end of the premaxillary posteroventral process located anterodorsal to the prefrontal.

88 (1): Nasal located posterior to the posteroventral process of the premaxilla.

142 (4): Nasal forming elongate, stick-shaped solid crest triangular in cross-section.

173 (0): Arcuate or smoothly curved anterodorsal margin of the prefrontal along the orbital rim.

178 (1): Presence of the dorsal promontrium of the postorbital.

190 (2): Medial rami of the squamosals contacting each other.

191 (1): Presence of the frontal platform for supporting the supracranial crest.

193 (1): The nasal contact surface of the frontal/total length of the frontal over 0.4.

194 (2): Anterior part of the frontal angled in between 25 º and 35 º relative to the level.

199 (2): Absence of the fontanelle of the skull roof in juveniles.

200 (2): Absence of the nasal-frontal fontanelle.

241 (1): Slightly elongated neural spines of the anterior dorsal vertebrae.

255 (1): Coracoid ventral process, dorsoventral height/anteroposterior width: 0.6 to 0.8.

270 (2): Width of the deltopectoral crest relative to the humeral shaft greater than 1.90.

274 (0): Ulna length/width: less than 10.

336 (1): Club-shaped distal end of the fibula.

**Clade P (Edmontosaurini)**

12 (1): Dentary marginal denticles each consisting one rounded knob.

74 (4): Thick and lip-shaped premaxillary oral margin.

151 (0): Angle between the external naris and the maxillary dental battery less than 30 º

163 (0): Absence of nasal elevation.

**Clade Q**

41 (1): Ventral deflection of at the predentary articulation region angled 17 º – 25 º.

66 (1): Ventromedial margin of the anterior part of surangular angled less than 150 º relative to the posterior process.

225 (0): Nearly equal anteroposterior widths of the orbit and the infratemporal fenestra.

287 (0): Preacetabular process of the ilium angled greater than 150 degrees relative to the central plate.

**Clade R**

47 (0): Slightly bowed ventral margin of the dentary ventral to the coronoid process.

**Clade S**

1 (2): More than 45 dentary dental alveoli.

181 (2): Anteroposteriorly broad ventral process of the postorbital, strongly expanded both anterior and posterior margins.

183 (1): Short and robust squamosal process of the postorbital.

204 (1): Finger-shaped, anterocaudally long median anterior process of the parietal.

234 (0): Strong ventral deflection of the occipital condyle.

309 (2): Circular prepubic process well developed ventrally.

**Clade T**

27 (1): Anteroventral and dorsal margins of the predentary angled 56 º – 74 º.

38 (3): Edentulous region of the dentary longer than 0.45 times the length between the anteriormost alveolus and the posterior margin of the coronoid process.

48 (2): Coronoid process of the dentary anteriorly inclined more than 70 º.

182 (1): Jugal process of the postorbital bearing deep inner cavity.

221 (1): Short postorbital process of the laterosphenoid.

324 (1): Obturator foramen completely enclosed by the obturator process and the pubic peduncle of the ischium.

327 (0): Dorsoventral depth of the ischium/length of the ischial shaft: up to 0.05.

**Autapomorphies**

*Kamuysaurus japonicus*

22 (1): Slightly curved primary ridge of maxillary tooth

43 (1): Mediolateral width of dentary symphyseal region/minimum breadth of the dentary caudal to the symphyseal process in between 1.65 and 2.60.

49 (1): Well-developed coronoid process of the dentary, anterior margin more pronounced than the posterior margin.

117 (1): Triangular shaped ventral margin of the jugal anterior process which is as high as its width.

119 (1): Palatine articular surface of the jugal inclined in between 110 º and 130 º.

123 (3): Straight posterior margin of the jugal with pointed dorsal apex.

173 (0): Arcuate or smoothly curved anterodorsal margin of the prefrontal along the orbital rim.

186 (0): Posterior end of the squamosal process of the postorbital located anterior to the quadrate cotylus.

191 (1): Presence of the frontal platform for supporting the supracranial crest.

193 (1): The nasal contact surface of the frontal/total length of the frontal over 0.4.

227 (0): Subrectangular infratemporal fenestra.

270 (0): Width of the deltopectral crest relative to the humeral shaft less than 1.65

272 (0): Slender humerus

“*Hadrosaurus foulkii*”

-

*Wulagasaurus dongi*

-

*Acristavus gagslarsoni*

42 (0): Anteriorly located posteriormost end of the deflected ventral margin of the dentary.

130 (0): Quadratojugal notch of the quadrate located nearly at the midpoint of the quadrate.

163 (0): Absence of nasal elevation.

181 (0): Anteroposteriorly broad ventral process of the postorbital, strongly expanded anterior margin.

315 (0): Length/width ratio of the ischial peduncle of the pubis less than 2.

*Maiasaura peeblesorum*

130 (0): Quadratojugal notch of the quadrate located nearly at the midpoint of the quadrate.

348 (1): Angular ventral flange of jugal

*Probrachylophosaurus bergei*

16 (2): Presence of more than 45 maxillary dental alveoli.

41 (2): Ventral deflection of at the predentary articulation region angled at larger than 25 º.

135 (0): Medial condyle of the quadrate only slightly elevated.

*Brachylophosaurus canadensis*

38 (3): Edentulous region of the dentary longer than 0.45 times the length between the anteriormost alveolus and the posterior margin of the coronoid process.

*Kritosaurus navajovius*

6 (2): Enameled surface of the dentary teeth bears two or three ridges

29 (1): Presence of six denticles.

121 (0): Shallow and wide concave ventral margin of the jugal.

138 (2): Presence of solid supracranial crest which is laterally excavated by posterior expansion of the circumnarial fossa.

139 (1): Presence of supracranial crest composed of nasals.

*Secernosaurus koerneri*

43 (1): Mediolateral width of dentary symphyseal region/minimum breadth of the dentary caudal to the symphyseal process in between 1.65 and 2.60.

112 (1): Moderately inclined ectopterygoid shelf of maxilla (10 º -20º)

181 (2): Anteroposteriorly broad ventral process of the postorbital, strongly expanded both anterior and posterior margins.

219 (0): Maximum width across the sphenoccipital tubercles/minimum width of the rostral constriction less than 1.5.

252 (1): Length ratio of scapular articular surface/glenoid of the coracoid in between 1.0 and 1.3.

253 (0): Angle between the scapular articular surface and the glenoid of the coracoid greater than 115 º.

295 (0): Dorsoventral height/anteroposterior length of iliac central plate greater than 0.8.

303 (1): Extremely thick postacetabular process of the ilium.

310 (1): Dorsoventrally short prepubic process of the pubis.

*Rhinorex condrupus*

7 (0): posteriorly positioned primary ridge of dentary teeth.

*Gryposaurus latidens*

5 (0): Height/width ratio of dentary tooth crown less than 1.95.

11 (1): Slightly big marginal denticles of dentary teeth.

121 (0): Shallow and wide concave ventral margin of the jugal.

149 (1): Nasal promontory located dorsal to the posterior margin of the external naris.

*Gryposaurus notabilis*

26 (0): Triangular-shaped predentary denticles.

*Gryposaurus monumentensis*

16 (2): Presence of more than 45 maxillary dental alveoli.

125 (2): Width of jugal at the posterior contraction/distance between the ventral extremities of infratemporal fenestra and the orbital rim larger than 0.8.

231 (2): External naris longer than 0.4 times the skull length.

*Lophorhothon atopus*

6 (2): Enameled surface of the dentary teeth bears two or three ridges.

*Prosaurolophus maximus*

296 (0): Presence of the ridge connecting the caudoventral margin of the supraacetabular process and the dorsal margin of the postacetabular process of ilium

310 (1): Dorsoventrally short prepubic process of the pubis.

317 (2): Markedly elongated prepubic process of the pubis.

341 (0): Length/width of the metatarsal III greater than 4.5.

*Saurolophus osborni*

1 (2): More than 45 dentary dental alveoli

27 (3): Anteroventral and dorsal margins of the predentary angled less than 40º

*Saurolophus angustirostris*

5 (1): Height/width ratio of the dentary teeth crown in between 1.95 and 2.70.

237 (1): concave dorsal margin of the axial neural spine.

*Laiyangosaurus youngi*

21 (0): Primary ridge of the maxillary tooth crown located distal to the midline.

*Kerberosaurus manakini*

-

*Shantungosaurus giganteus*

42 (2): Posteriorly located posteriormost end of the deflected ventral margin of the dentary.

129 (1): Quadrate strongly curved posteriorly.

203 (2): Slightly downward dorsal surface of the parietal.

*Edmontosaurus regalis*

255 (1): Coracoid ventral process, dorsoventral height/anteroposterior width: 0.6 to 0.8.

323 (1): Anteroposterior length of the ischial pubic peduncle nearly equivalent with its dorsoventral depth.

*Edmontosaurus annectens*

99 (1): Triangular lateral exposure of the maxilla anterior to the jugal articulation.

230 (1): Skull is more than twice as long as the height at the quadrate

231 (0): External naris shorter than a quarter of the skull length.

271 (1): Angle between the anterolateral and ventral margins of the humeral deltopectoral crest less than 110º

341 (0): Length/width of the metatarsal III greater than 4.5.

## Supplementary Text S3: Modified and added characters for the phylogenetic analysis in this study.

**Modified characters:**

99. Morphology of the laterally exposed surface of the rostrodorsal region of the maxilla, adjacent and rostral to the jugal articular surface (Prieto-Márquez^1^, character 88; modified based on Prieto-Márquez and others^2^): (0) subarcuate profile; (1) tall and sharply triangular to finger-shaped; (2) subtrapezoid to subrectangular, with horizontal dorsal margin under lacrimal.

236. Number of the cervical vertebrae (Horner and others^3^, character 66; Xing and others^4^, character 236; modified based on Evans and Reisz^5^): (0) 11 or fewer; (1) 12 or 13 (2) 14 or more.

**Additional characters:**

347. Maxilla. Rostrodorsal margin bearing a prominent subrectangular flange that rises vertically above the rostroventral process (Prieto-Marquez and others^2^, character 80): (0) absent; (1) present.

348. Jugal. Ventral flange shape (Suzuki and others^6^, character 21; Evans and Reisz^5^, character 31): (0) rounded or lobate; angular (1).

349. Frontal. Bifurcation of the rostromedial margin of the frontals at the sagittal plane of the skull roof, leaving a V-shaped space in between (Prieto-Márquez^1^, character 138; Prieto-Marquez and others ^2^, character 126): (0) absent; (1) present.

350. Ratio between the proximodistal length of the ulna and that of the femur (Takasaki and others^7^): (0) less than 0.53; (1) 0.53 or greater.

**References for Supplementary Text S3**

1 Prieto-Márquez, A. Global phylogeny of Hadrosauridae (Dinosauria: Ornithopoda) using parsimony and Bayesian methods. *Zool. J. Linn. Soc.* **159**, 435-502, doi:10.1111/j.1096-3642.2009.00617.x (2010).

2 Prieto-Márquez, A., Dalla Vecchia, F. M., Gaete, R. & Galobart, A. Diversity, relationships, and biogeography of the lambeosaurine dinosaurs from the European Archipelago, with description of the new Aralosaurin *Canardia garonnensis*. *PLoS ONE* **8**, e69835, doi:10.1371/journal.pone.0069835 (2013).

3 Horner, J. R., Weishampel, D. B. & Forster, C. A. Hadrosauridae in *The Dinosauria: Second Edition* (eds. Weishampel, D. B., Dodson, P., & Osmólska, H.) 438-463 (University of California Press, 2004).

4 Xing, H. *et al.* A new basal hadrosauroid dinosaur (Dinosauria: Ornithopoda) with transitional features from the late cretaceous of Henan Province, China. *PLoS ONE* **9**, e98821, doi:10.1371/journal.pone.0098821 (2014).

5 Evans, D. C. & Reisz, R. R. Anatomy and relationships of *Lambeosaurus magnicristatus*, a crested hadrosaurid dinosaur (Ornithischia) from the Dinosaur Park Formation, Alberta. *J. Vert. Paleontol.* **27**, 373-393 (2007).

6 Suzuki, D., Weishampel, D. B. & Minoura, N. *Nipponosaurus sachalinensis* (Dinosauria; Ornithopoda): anatomy and systematic position within Hadrosauridae. *J. Vert. Paleontol.* **24**, 145-164 (2004).

7 Takasaki, R., Chiba, K., Kobayashi, Y., Currie, P. J. & Fiorillo, A. R. Reanalysis of the phylogenetic status of *Nipponosaurus sachalinensis* (Ornithopoda: Dinosauria) from the Late Cretaceous of Southern Sakhalin. *Hist. Biol.*, 1-18, doi:10.1080/08912963.2017.1317766 (2017).

# Supplementary Tables

## Supplementary Table S1. Measurements of the skull elements of *Kamuysaurus japonicus* gen et sp. nov. in millimeters. Asterisk indicates the incompleteness of the element and the maximum value of preserved element. “est” indicates its estimated value.

| Element | | Measurement | Left | Right |
| --- | --- | --- | --- | --- |
| Skull | Maxilla | Length | 200.4* | 280.1* |
|  |  | Height | 86.6* | 86.3* |
|  | Prefrontal | Length | 100.8* | 97.2* |
|  |  | Width | 42.4 | 41.2 |
|  | Jugal | Length | 144.4* | 289.8 |
|  |  | Height | 111.1* | 153.7 |
|  | Quadratojugal | Length | 65.9* | 73.9* |
|  |  | Height | 60.9* | 112.5* |
|  | Quadrate | Height | 308.1* | 291.3 |
|  |  | Width, mandibular condyle | -- | 60.1 |
|  | Pterygoid | Length | 97.8* | -- |
|  |  | Height | 70.1* | -- |
|  | Squamosal | Length | 104.0* | -- |
|  |  | Width | 137.6 | -- |
|  | Postorbital | Length | 121.3* | 124.8* |
|  |  | Width | 61.4* | 86.6* |
|  | Frontal | Length | 158.6* | 93.8* |
|  |  | Width | 92.1* | 103.4* |
|  | Exoccipital | Length | 172.6 | |
|  |  | Width | 209.6* | |
| Mandible | Dentary | Length | 526.0* | 589.0est |
|  |  | Height | 220.8 | 229.3 |
|  | Surangular | Length | 87.8* | 185.2* |
|  |  | Width | 55.3* | 61.1 |
|  | Splenial | Length | 158.6* | -- |
|  |  | Height | 69.4* | -- |
|  | Ceratobranchial | Length | 144.1* | 294.9 |
|  |  | Height | 23.1* | 37.9 |
| Dentition | Maxillary teeth | Crown height, average | 27.0 | |
|  |  | Crown height, maximum | 30.8 | |
|  |  | Crown height, minimum | 18.8 | |
|  | Dentary teeth | Crown height, average | 31.3 | |
|  |  | Crown height, maximum | 37.3 | |
|  |  | Crown height, minimum | 21.7 | |

## Supplementary Table S2. Measurements of the axial elements of *Kamuysaurus japonicus* gen et sp. nov. in millimeters. Asterisk indicates the incompleteness of the element and the maximum value of preserved element. “est” indicates its estimated value.

| Element | # | Total height | Centrum length | Centrum height along the cranial aspect |
| --- | --- | --- | --- | --- |
| Cervical vertebrae | 1 | 118.4 | 35.4 | 31.3 |
|  | 2 | 83.9* | 102.0 | 55.6 |
|  | 3 | 110.1* | 85.4 | 56.8 |
|  | 4 | 119.9 | 93.6 | 67.3 |
|  | 5 | 123.6 | 84.4 | 61.8 |
|  | 6 | 111.2* | 91.9 | 70.6 |
|  | 7 | 86.6* | 96.7 | 68.9 |
|  | 8 | 133.5* | 96.0 | 69.4 |
|  | 9 | 134.1* | 103.9 | 77.0est |
|  | 10 | 176.9 | 87.5* | 68.6 |
|  | 11 | 140.3 | 86.5 | 70.2 |
|  | 12 | 120.1* | 91.5 | 57.9* |
|  | 13 | 146.0est | 72.8* | 56.4* |
| Dorsal vertebrae | 1 | 255.4 | 108.1 | 60.8 |
|  | 2 | 305.3* | 100.0* | 61.0 |
|  | 3 | 281.3* | 100.0 | 62.7 |
|  | 4 | 55.6* | -- | -- |
|  | 5 | 312.2* | -- | -- |
|  | 6 | 326.0 | 89.2 | 61.1 |
|  | 7 | 225.3* | -- | -- |
|  | 8 | 320.2 | 81.9* | 63.1 |
|  | 9 | 160.0* | -- | -- |
|  | 10 | 256.8* | -- | -- |
|  | 11 | 319.1 | 79.4* | 88.4* |
|  | 12 | 267.0* | 84.7 | 83.0 |
|  | 13 | 313.6* | 81.0* | 73.3* |
|  | 14 | 274.9* | -- | -- |
|  | 15 | 262.9* | -- | -- |
|  | 16 | 434.5 | 68.5 | 105.0 |
|  | 17 | 405.4 | 73.6 | 101.6 |
| Caudal vertebrae | 1 | 380.0est | 64.0 | 114.5 |
|  | 2 | 370.0est | 63.4 | 121.0 |
|  | 3 | 163.7* | 65.0 | 111.3* |
|  | 4 | 381.0 | 63.5 | 141.5 |
|  | 5 | 370.8 | 63.0 | 122.0 |
|  | 6 | 153.1* | 71.0 | 116.3 |
|  | 7 | 437.1 | 65.5 | 118.1* |
|  | 8 | 350.0* | 48.9 | 114.5 |
|  | 9 | 385.0est | 67.3 | 101.4 |
|  | 10 | 89.4* | -- | -- |
|  | 11 | 140.3* | 66.0 | 97.9 |
|  | 12 | 194.5* | 67.8 | 90.9est |
|  | 13 | 308.9* | 64.7 | 89.2 |
|  | 14 | 246.9* | 66.2 | 89.0 |
|  | 15 | 235.3* | 65.4 | 83.8 |
|  | 16 | 124.2* | 66.2 | 82.9 |
|  | 17 | 186.0* | 65.5 | 83.2 |
|  | 18 | 375.0est | 63.3 | 80.3 |
|  | 19 | 249.4* | 62.6 | 84.3 |
|  | 20 | 305.0* | 67.5 | 70.2* |
|  | 21 | 325.0est | 72.7 | 66.2 |
|  | 22 | 302.4 | 63.0 | 75.8 |
|  | 23 | 230.9* | 63.4 | 77.1 |
|  | 24 | 226.0* | 69.1 | 70.4 |
|  | 25 | 225.0* | 70.3 | 71.4 |
|  | 26 | 147.9* | 67.0 | 69.3 |
|  | 27 | 207.0* | 66.0 | 74.5 |
|  | 28 | 99.3* | 63.9* | 67.4 |
|  | 29 | 104.8* | 65.7 | 65.1 |
|  | 30 | 240.1* | 64.8 | 65.3 |
|  | 31 | 155.2* | 64.3 | 66.2 |
|  | 32 | 218.2* | 64.5 | 63.2 |
|  | 33 | 183.0* | 53.0* | 62.6* |
|  | 34 | 231.7* | 60.8 | 63.8 |
|  | 35 | 194.4* | 61.3 | 61.4 |
|  | 36 | 194.5 | 61.6 | 58.7est |
|  | 37 | 206.4* | 60.5 | 60.1 |
|  | 38 | 198.7 | 59.7 | 58.5 |
|  | 39 | 197.4* | 59.6 | 54.3 |
|  | 40 | 192.6est | 55.3 | 54.7 |
|  | 41 | 175.6 | 53.4* | 58.9* |
|  | 42 | 156.6 | 63.0* | 47.8* |
|  | 43 | 155.2* | 52.8 | 50.8* |
|  | 44 | 79.8* | 48.4* | 54.1 |

## Supplementary Table S3. Measurements of the appendicular elements of *Kamuysaurus japonicus* gen et sp. nov. in millimeters. Asterisk indicates the incompleteness of the element and the maximum value of preserved element. “est” indicates its estimated value.

| Element | | Measurements | Left | Right |
| --- | --- | --- | --- | --- |
| Pectoral girdle | Scapula | Length | 780.0 | 755.0 |
|  |  | Height at proximal end | 143.8 | 144.6 |
|  | Coracoid | Maximum length | 211.3 | 185.4 |
|  |  | Maximum width | 135.6 | 114.8 |
|  | Sternum | Maximum length | -- | 400.0* |
|  |  | Maximum width | -- | 109.1* |
| Forelimb | Humerus | Length | 620.0 | 610.0est |
|  |  | Circumference | 251.0 | 240.0* |
|  |  | Width at proximal end | 140.2 | 142.5* |
|  | Ulna | Length | 673* | 696.0 |
|  |  | Circumference | 175.0 | 180.0 |
|  |  | Width, proximal end | 132.4 | 114.2* |
|  | Radius | Length | 298.0* | 385.0* |
|  |  | Circumference | -- | 144.0 |
|  |  | Width, proximal end | -- | 82.5 |
|  | Distal carpal | Width, maximum | 43.8 | 43.5 |
|  |  | Length | 23.7 | 23.2 |
|  | Metacarpal II | Length | 145.4* | 147.3* |
|  |  | Width, shaft | 22.1 | 21.1 |
|  | Metacarpal III | Length | 285.8 | 281.5 |
|  |  | Width, proximal end | 37.1 | 34.9 |
|  |  | Width, shaft | 25.3 | 27.8 |
|  | Metacarpal IV | Length | 294.1 | 282.5est |
|  |  | Width, proximal end | 63.9 | 61.8 |
|  |  | Width, shaft | 35.1 | 38.5 |
|  | Metacarpal V | Length | -- | 97.5 |
|  |  | Width, proximal end | -- | 45.4 |
|  |  | Width, shaft | -- | 34.7 |
|  | Manual phalanx II-2 | Length | 25.1 | -- |
|  |  | Width, shaft | 38.7 | -- |
|  | Manual phalanx III-1 | Length | 44.9 | 44.4 |
|  |  | Width, proximal end | 56.5 | 50.1 |
|  |  | Width, shaft | 45.3 | 44.3 |
|  | Manual phalanx III-2 | Length | -- | 23.7 |
|  |  | Width, proximal end | -- | 45.5 |
|  |  | Width, shaft | -- | 42.7 |
|  | Manual phalanx III-3 | Length | -- | 39.7* |
|  |  | Width, proximal end | -- | 38.2* |
|  | Manual phalanx IV-1 | Length | 57.1 | 62.1 |
|  |  | Width, proximal end | 32.4 | 33.2 |
|  |  | Width, shaft | 28.4 | 25.1 |
|  | Manual phalanx V-1 | Length | -- | 61.6* |
|  |  | Width, proximal end | -- | 26.2* |
|  |  | Width, shaft | -- | 25.2* |
| Pelvis | Ilium | Length | 1005.0* | 993.0* |
|  |  | Height | 196.6* | 180.0* |
|  | Pubis | Length, preserved | 628* | 509* |
|  |  | Height, preserved | 190.7* | 149.3* |
|  | Ischium | Length | 822.0* | 620.0* |
|  |  | Height | 152.2* | 255.9* |
| Hind limb | Femur | Length | 1060.0 | 1095.0 |
|  |  | Width at proximal end | 292.7 | 232.0* |
|  |  | Shaft, circumference | -- | 453.0 |
|  | Tibia | Length | -- | 1035.0 |
|  |  | Anteroposterior length, proximal end | 315.0* | 364.0 |
|  |  | Shaft, circumference | -- | 364.0 |
|  |  | Transverse width, distal end | 252.0 | 296.0 |
|  | Fibula | Length | 915.0 | -- |
|  |  | Anteroposterior length, proximal end | 164.4 | -- |
|  | Metatarsal II | Length | -- | 360.0 |
|  |  | Width, shaft | -- | 73.71* |
|  | Metatarsal III | Length | -- | 366.0 |
|  |  | Width, shaft | -- | 84.05* |
|  | Metatarsal IV | Length | 158.4* | 312.5* |
|  |  | Width, shaft | 98.1 | 69.6* |
|  | Pedal phalanx II-1 | Length | 128.2 | 112.3est |
|  |  | Width, shaft | 85.8* | 68.0* |
|  | Pedal phalanx II-2 | Length | 41.5 | 41.9 |
|  |  | Width, maximum | 78.1 | 54.1* |
|  | Pedal phalanx III-1 | Length | 112.2 | 114.5 |
|  |  | Width, maximum | 124.7 | 127.7 |
|  | Pedal phalanx III-2 | Length | 27.5 | 34.2 |
|  |  | Width, maximum | 94.3* | 114.5 |
|  | Pedal phalanx III-3 | Length | 30.1 | -- |
|  |  | Width, maximum | 84.3* | -- |
|  | Pedal phalanx III-4 | Length | 89.3* | -- |
|  |  | Width | 78.8* | -- |
|  | Pedal phalanx IV-1 | Length | 92.1 | 99.3* |
|  |  | Width, maximum | 95.5 | 81.6* |
|  | Pedal phalanx IV-2 | Length | 24.3 | -- |
|  |  | Width, maximum | 61.0* | -- |
|  | Pedal phalanx IV-3 | Length | 23.1 | -- |
|  |  | Width, maximum | 63.9 | -- |

## Supplementary Table S4. LAG circumferences in millimeters

| LAG No. | Circumference |
| --- | --- |
| 1 | 290.4 |
| 2 | 331.2 |
| 3 | 347.8 |
| 4 | 365.6 |
| 5 | 377.2 |
| 6 | 384.4 |
| 7 | 388.6 |
| 8 | 391.2 |
| 9 | 393.5 |

## Supplementary Table S5. Estimated parameters of fitted growth models in this study, AIC values, and estimated age at the 1st growth marks when different growth models were fitted to the dataset.

| Models | A | K | m | AIC | AICc | delta AICc | Age at 1st LAG |
| --- | --- | --- | --- | --- | --- | --- | --- |
| Monomolecular | 395.6 | 0.4 | 0.0 | 43.9 | 49.9 | 0.0 | 2.9 |
| Von Bertalanffy | 394.8 | 0.5 | 0.7 | 44.8 | 50.8 | 0.9 | 3.8 |
| Gompertz | 394.4 | 0.5 | 1.0 | 45.2 | 51.2 | 1.3 | 4.4 |
| Logistic | 393.4 | 0.6 | 2.0 | 46.6 | 52.6 | 2.7 | 6.7 |
| Extreme | 391.8 | 0.7 | 4.0 | 49.2 | 55.2 | 5.3 | 11.7 |
| Innominate | 389.8 | 0.9 | 8.4 | 53.8 | 59.8 | 9.9 | 19.4 |

## Supplementary Table S6. Depositional environments

| Taxa | Depositional environment | Formation | subunit | Reference |
| --- | --- | --- | --- | --- |
| *Adelolphus hutchisoni* | Terrestrial + Marginal | Wahweap Formation | Upper Member | Lawton and others^1^, Jinnah and Roberts^2^ |
| *Bactrosaurus johnsoni* | Terrestrial | Iren Dabasu Formation |  | Currie and Eberth^3^; Xing and others^4^ |
| *Claosaurus agilis* | Marine | Niobrara Chalk Formation | Smoky Hill Chalk Member | Hattin^5^ |
| *Eolambia caroljonesa* | Terrestrial | Cedar Mountain Formation | Mussentuchit Member | Garrison and others^6^ |
| *Eotrachodon orientalis* | Marine | Mooreville Chalk | Unnamed | Wylie and King^7^ |
| *Equijubus normani* | Terrestrial |  | Middle Grey Unit | Tang et al. (2001) |
| *Gilmoreosaurus mongliensis* | Terrestrial | Iren Dabasu Formation |  | Currie and Eberth^3^; Xing and others^4^ |
| *Iguanodon bernissartensis* | Terrestrial | Sainte-Barbe Clays Formation |  | Dejax and others^8^; Yans and others^9^; Spagna and others^10^ |
| *Jintasaurus meniscus* | Terrestrial |  |  | Tang and others^11^ |
| *Jinzhousaurus yangi* | Terrestrial | Yixian Formation | Dakangpu Member | Wang and Xu^12^; Barret and others^13^; Wang and others^14^ |
| *Levnesovia transoxiana* | Terrestrial | Bissekty Formation |  | Archibald and others^15^; Redman and others^16^ |
| *Mantellisaurus atherfieldensis* | Terrestrial | Wessex Formation |  | Insole and Hutt^17^; Radley^18^ |
| *Nanningosaurus dashiensis* | ? |  |  |  |
| *Nanyangosaurus zhugeii* | Terrestrial | Xiaguan Formation (Equivalent of Gagou Fm.) |  | Liang and others^19^; Wang and others^20^ |
| *Ouranosaurus nigeriensis* | Terrestrial | upper Elrhaz Formation |  | Sereno and others^21,22^ |
| *Plesiohadros djadokhtaensis* | Terrestrial | Djadokhta Formation |  | Tsogtbaatar and others^23^ |
| *Probactrosaurus gobiensis* | Terrestrial | Dashuigou Formation |  | Van Itterbeeck and others^24^ |
| *Sirindhorna khoratensis* | Terrestrial + Marginal | Khok Krut Formation |  | Racey^25^; Racey and others^26^ |
| *Tanius sinensis* | Terrestrial | Jiangjunding Formation |  | Liu and others^27^, Wang and others^28^ |
| *Telmatosaurus transsylvanicus* | Terrestrial | Sampetru Formation+Densus-Ciula Formation + "Pui beds" |  | Grigorescu and Csiki^29^; Vecchia^30^ |
| *Tethyshadros insularis* | Marginal | Liburnian Formation |  | Arbulla and others^31^; Vecchia^32^ |
| *Xuwulong yueluni* | Terrestrial |  |  | Tang and others^11^ |
| *Yunganglong datongensis* | ? | lower Zhumapu Formation |  |  |
| *Zhanghenglong yangchengensis* | Terrestrial | Majiacun Formation | middle member (Unit 2) | Xing and others^33^ |
| *Zuoyunlong huangi* | ? | lower Zhumapu Formation |  |  |
| *Acristavus gagslarsoni* | Terrestrial | lower Two Medicine Formation; Wahweap Formation | upper Middle Mudstone Member | Gates and others^34^; Jinnah and others^35^ |
| *Brachylophosaurus canadensis* | Terrestrial + Marginal | Oldman Formation; Judith River Formation | Unit 2 (OF), McClelland Ferry Member (JRF) | Cuthbertson and Holmes^36^; Rogers and others^37^ |
| *Edmontosaurus annectens* | Terrestrial + Marginal | Lance Formation, Hell Creek Formation, Frenchman Formation |  | Connor^38^, Mclver^39^, Murphy and others^40^, Colson and others^41^, Bamforth and others^42^, Ullman and others^43^ |
| *Edmontosaurus regalis* | Terrestrial | Horseshoe Canyon Formation, Wapiti Formation | Horsethief Member (HCF), Unit 4 (WF) | Eberth and Braman^44^, Bell and Campione^45^, Bell and others^46^, Evans and others^47^ |
| *Gryposaurus latidens* | Terrestrial | Two Medicine Formation | Lithofacies 3 | Rogers^48^ |
| *Gryposaurus monumentensis* | Terrestrial | Kaiparowits Formation | upper half of the middle unit | Roberts and others^49^ |
| *Gryposaurus notabilis* | Terrestrial | Dinosaur Park Formation | sandy zone | Eberth^50^ |
| *Hadrosaurus foulkii* | Marine | Woodbury Formation |  | Prieto-Márquez and others^51^; Gallagher^52^ |
| ***Kamuysaurus japonicus*** | Marine | Hakobuchi Formation | IVb | This study |
| *Kerberosaurus manakini* | Terrestrial | Udurchukan Formation | Unit 2 (Blagoveschensk) | Lauters and others^53^, Van Itterbeeck and others^54^, Godefroit and others^55^ |
| *Kritosaurus navajovius* | Terrestrial + Marginal | upper Kirtland Formation, Cerro del Pueblo Formation | De-Na-Zin Mbr | Hunt and Lucas^56^, Williamson^57^, Kirkland and others^58^, Eberth and others^59^ |
| *Laiyangosaurus youngi* | Terrestrial | Jingangkou Formation |  | Liu and others^27^, Wang and others^28^ |
| *Lophorhothon atopus* | Marine | Mooreville Chalk |  | Wylie and King^7^ |
| *Maiasaura peeblesorum* | Terrestrial | Two Medicine Formation | Lithofacies 4 | Schmitt and others^60^ |
| *Probrachylophosaurus bergei* | Terrestrial | Judith River Formation | upper portion of Kennedy Coulee | Eberth^50^, Freedman Fowler and Horner^61^ |
| *Prosaurolophus maximus* | Terrestrial + Marginal | Dinosaur Park Formation, Two Medicine Formation | Sandy zone + muddy zone (DPF), Unit 5 (TMF) | Eberth^50^, Rogers^48^ |
| *Rhinorex condrupus* | Terrestrial + Marginal | Neslen Formation |  | Kirschbaum and Hettinger^62^ |
| *Saurolophus angustirostris* | Terrestrial | Nemegt Formation |  | Bell and others^63^, Eberth^64^ |
| *Saurolophus osborni* | Terrestrial + Marginal | Horseshoe Canyon Formation | uppermost Morrison Member - Tolman Member | Eberth and Braman^44^, Eberth and others^65^ |
| *Secernosaurus koerneri* | Terrestrial | Bajo Barreal Formation, Los Alamitos Formation | Upper Member (BBF) | Bonaparte and others^66^, Paredes and others^67^ |
| *Shantungosaurus giganteus* | Terrestrial | Uppermost Xingezhuang Formation - lower Hongtuya Formation |  | Kuang and others^68^, He and others^69^ |
| *Wulagasaurus dongi* | Terrestrial | Yuliangze Formation |  | Godefroit and others^70^ |
| *Amurosaurus riabinini* | Terrestrial | Udurchukan Formation | Unit 2 | Lauters and others^53^ |
| *Aralosaurus tuberiferus* | Marginal | Bostobe Formation |  | Kordikova and others^71^ |
| *Arenysaurus ardevoli* | Marginal | Tremp Formation | gray unit' | Pujalte and Schmitz^72^, Riera and others^73^, Cruzado-Caballero and others^74^ |
| *Blasisaurus canudoi* | Marginal | Aren Formation |  | Oms and Canudo^75^, Cruzado-Caballero and others^76^, Vecchia and others^77^ |
| *Canardia garonnensis* | Marginal | Marnes d'Auzas Formation |  | Laurent and others^78^, Prieto-Márquez and others^79^ |
| *Charonosaurus jiayinensis* | Terrestrial | Yuliangze Formation |  | Godefroit and others^80^ |
| *Corythosaurus casuarius* | Terrestrial | Dinosaur Park Formation | sandy zone | Eberth^50^ |
| *Corythosaurus intermedius* | Terrestrial | Dinosaur Park Formation | sandy zone | Eberth^50^ |
| *Hypacrosaurus altispinus* | Terrestrial + Marginal | Horseshoe Canyon Formation | Morrison Member + Tolman Member | Eberth and Braman^44^, Eberth and others^65^ |
| *Hypacrosaurus stebingeri* | Terrestrial | Two Medicine Formation, Oldman Formation | Lithofacies 3 (TMF) | Rogers^48^, Varricchio and others^81^ |
| *Jaxartosaurus aralensis* | ? | Dabrazinskaya Svita |  |  |
| *Lambeosaurus lambei* | Terrestrial + Marginal | Dinosaur Park Formation | sandy zone + muddy zone | Eberth^50^ |
| *Lambeosaurus magnicristatus* | Terrestrial + Marginal | Dinosaur Park Formation | muddy zone | Eberth^50^ |
| *Magnapaulia laticauda* | Terrestrial + Marginal | El Gallo Formation | El Disecado Member | Renne and others^82^ |
| *Nipponosaurus sahalinensis* | Marine |  |  | Nagao^83^ |
| *Olorotitan arharensi* | Terrestrial | Udurchukan Formation |  | Van Itterbeeck and others^54^, Godefroit and others^84^ |
| *Pararhabdodon isonense* | Terrestrial | Tremp Formation | lower red unit | Vecchia and others^77^ |
| *Parasaurolophus cyrtocristatus* | Terrestrial + Marginal | Fruitland Formation, Kaiparowits Formation | ?Fossil Forest Mbr (FF), lower 1/3 (KF) | Fassett and Hinds ^85^, Roberts and others^49^, Tapanila and Roberts^86^ |
| *Parasaurolophus tubicen* | Terrestrial | Kirtland Formation | De-Na-Zin Member | Hunt and Lucas^56^ |
| *Parasaurolophus walkeri* | Terrestrial | Dinosaur Park Formation | sandy zone | Eberth^50^ |
| *Sahaliyania elunchunorum* | Terrestrial | Yuliangze Formation |  | Godefroit and others^70^ |
| *Tsintaosaurus spinorhinus* | Terrestrial | Jingangkou Formation |  | Liu and others^27^, Wang and others^28^ |
| *Velafrons coahuilensis* | Marginal | Cerro del Pueblo Formation | 335m above the base of the formation | Eberth and others^59^ |

**References for Supplementary Table S6**

1 Lawton, T. F., Pollock, S. L. & Robinson, R. A. J. Integrating sandstone petrology and nonmarine sequence stratigraphy: application to the late Cretaceous fluvial systems of southwestern Utah, U.S.A. *J. Sediment. Res.* **73**, 389-406, doi:10.1306/100702730389 (2003).

2 Jinnah, Z. A. & Roberts, E. M. Facies associations, paleoenvironment, and base-level changes in the Upper Cretaceous Wahweap Formation, Utah, U.S.A. *J. Sediment. Res.* **81**, 266-283, doi:10.2110/jsr.2011.22 (2011).

3 Currie, P. J. & Eberth, D. A. Palaeontology, sedimentology and palaeoecology of the Iren Dabasu Formation (Upper Cretaceous), Inner Mongolia, People's Republic of China. *Cretaceous Res.* **14**, 127-144, doi:10.1006/cres.1993.1011 (1993).

4 Xing, H., He, Y., Li, L. & Xi, D. A review on the study of the stratigraphy, sedimentology, and paleontology of the Iren Dabasu Formation, Inner Mongolia in *Proceedings of the Thirteenth Anual Meeting of the Chinese Society of Vertebrate Paleontology* (ed. Dong, W.) 1-44 (China Ocean Press, 2012).

5 Hattin, D. E. Stratigraphy and depositional environment of Smoky Hill Chalk Member, Niobrara Chalk (Upper Cretaceous) of the type area, western Kansas. *Kans. Geol. Surv. Bull.* **225**, 108 (1982).

6 Garrison, J. R. *et al.* A multidisciplinary study of the Lower Cretaceous Cedar Mountain Formation, Mussentuchit Wash, Utah: a determination of the paleoenvironment and paleoecology of the *Eolambia caroljonesa* dinosaur quarry. *Cretaceous Res.* **28**, 461-494, doi:10.1016/j.cretres.2006.07.007 (2007).

7 Wylie, J. A. & King, D. T. Mooreville Chalk (Upper Cretaceous), sedimentary facies and sea-level cycles, west-central Alabama. *J. Ala. Acad. Sci.* **57**, 145 (1986).

8 Dejax, J., Pons, D. & Yans, J. Palynology of the dinosaur-bearing Wealden facies in the natural pit of Bernissart (Belgium). *Rev. Palaeobot. Palynol.* **144**, 25-38, doi:10.1016/j.revpalbo.2005.10.004 (2007).

9 Yans, J., Robaszynski, F. & Masure, E. Biostratigraphy of the Cretaceous sediments overlying the wealden facies in the Iguanodon Sinkhole at Bernissart in *Bernissart Dinosaurs and Early Cretaceous Terrestrial Ecosystems* (ed. Godefroit, P.) 69-78 (Indiana University Press, 2012).

10 Spagna, P., Yans, J., Schnyder, J. & Dupuis, C. The paleoenvironment of the Bernissart iguanodons: Sedimentological analysis of the Lower Cretaceous Wealden facies in the Bernissart area in *Bernissart Dinosaurs and Early Cretaceous Terrestrial Ecosystems* (ed. Godefroit, P.) 87-96 (Indiana University Press, 2012).

11 Tang, F. *et al.* Biostratigraphy and palaeoenvironment of the dinosaur-bearing sediments in Lower Cretaceous of Mazongshan area, Gansu Province, China. *Cretaceous Res.* **22**, 115-129, doi:10.1006/cres.2000.0242 (2001).

12 Wang, X. & Xu, X. A new iguanodontid (*Jinzhousaurus yangi* gen. et sp. nov.) from the Yixian Formation of western Liaoning, China. *Chin. Sci. Bull.* **46**, 1669-1672, doi:10.1007/bf02900633 (2001).

13 Barrett, P. M., Butler, R. J., Xiao-Lin, W. & Xing, X. Cranial anatomy of the iguanodontoid ornithopod *Jinzhousaurus yangi* from the Lower Cretaceous Yixian Formation of China. *Acta Palaeontol. Pol.* **54**, 35-48, doi:10.4202/app.2009.0105 (2009).

14 Wang, X., Pan, R., Butler, R. J. & Barrett, P. M. The postcranial skeleton of the iguanodontian ornithopod *Jinzhousaurus yangi* from the Lower Cretaceous Yixian Formation of western Liaoning, China. *Earth and Environmental Science Transactions of the Royal Society of Edinburgh* **101**, 135-159, doi:10.1017/s1755691010009266 (2011).

15 Archibald, J. D. *et al.* Précis of the Cretaceous paleontology, biostratigraphy and sedimentology at Dzharakuduk (Turonian?-Santonian), Kyzylkum Desert, Uzbekistan. *Bull. N. M. Mus. Nat. Hist. Sci.* **14**, 21-28 (1998).

16 Redman, C. M. & Leighton, L. R. Multivariate faunal analyses of the Turonian Bissekty Formation: variation in the degree of marine influence in temporally and spatially averaged fossil assemblages. *Palaios* **24**, 18-26, doi:10.2110/palo.2007.p07-072r (2009).

17 Insole, A. N. & Hutt, S. The palaeoecology of the dinosaurs of the Wessex Formation (Wealden Group, Early Cretaceous), Isle of Wight, Southern England. *Zool. J. Linn. Soc.* **112**, 197-215, doi:10.1111/j.1096-3642.1994.tb00318.x (1994).

18 Radley, J. D. Stratigraphy, palaeontology and palaeoenvironment of the Wessex Formation (Wealden Group, Lower Cretaceous) at Yaverland, Isle of Wight, southern England. *Proc. Geol. Assoc.* **105**, 199-208, doi:10.1016/s0016-7878(08)80119-8 (1994).

19 Liang, X., Wen, S., Yang, D., Zhou, S. & Wu, S. Dinosaur eggs and dinosaur egg-bearing deposits (Upper Cretaceous) of Henan Province, China: Occurrences, palaeoenvironments, taphonomy and preservation. *Progr. Nat. Sci.* **19**, 1587-1601, doi:10.1016/j.pnsc.2009.06.012 (2009).

20 Wang, D. *et al.* Discovery of invertebrate zoolite in the Xiaguan Formation of Xiaguan-Gaoqiu Basin, Henan, China, and its importance for stratigraphic subdivision comparison. *Acta Geologica Sinica* **87**, 1049-1058 (2013).

21 Sereno, P. C., Sidor, C. A., Larsson, H. C. E. & Gado, B. A new notosuchian from the Early Cretaceous of Niger. *J. Vert. Paleontol.* **23**, 477-482, doi:10.1671/0272-4634(2003)023[0477:annfte]2.0.co;2 (2003).

22 Sereno, P. C. *et al.* Evidence for avian intrathoracic air sacs in a new predatory dinosaur from Argentina. *PLoS ONE* **3**, e3303, doi:10.1371/journal.pone.0003303 (2008).

23 Tsogtbaatar, K., Weishampel, D. B., Evans, D. C. & Watabe, M. A new hadrosauroid (*Plesiohadros djadokhtaensis*) from the late Cretaceous Djadokhtan fauna of southern Mongolia in *Hadrosaurs* (eds. Eberth, D. A. & Evans, D. C.) 108-135 (Indiana University Press, 2015).

24 Van Itterbeeck, J., Bultynck, P., Li, G. W. & Vandenberghe, N. Stratigraphy, sedimentology and palaeoecology of the dinosaur-bearing Cretaceous strata at Dashuiguo (Inner Mongolia, People's Republic China). *Bulletin van het Koninklijk Belgisch Instituut voor Natuurwetenschappen, Aardwetenschappen* **71**, 51-70 (2001).

25 Racey, A. Mesozoic red bed sequences from SE Asia and the significance of the Khorat Group of NE Thailand. *Geological Society, London, Special Publications* **315**, 41-67, doi:10.1144/sp315.5 (2009).

26 Racey, A. & Goodall, J. G. S. Palynology and stratigraphy of the Mesozoic Khorat Group red bed sequences from Thailand. *Geological Society, London, Special Publications* **315**, 69-83, doi:10.1144/sp315.6 (2009).

27 Liu, Y., Kuang, H., Peng, N., xu, H. & Liu, Y. Sedimentary facies of dinosaur trackways and bonebeds in the Cretaceous Jiaolai Basin, eastern Shandong, China, and their paleogeographical implications. *Earth Sci. Front.* **18**, 9-24 (2011).

28 Wang, Q., Wang, X., Zhao, Z., Zhang, J. & Jiang, S. New turtle egg fossil from the Upper Cretaceous of the Laiyang Basin, Shandong Province, China. *An. Acad. Bras. Cienc.* **85**, 103-111, doi:doi.org/10.1590/S0001-37652013000100008 (2013).

29 Grigorescu, D. & Csiki, Z. Ontogenetic development of *Telmatosaurus transsylvanicus* (Ornitischia: Hadrosauria) from the Maastrichtian of the Haţeg Basin, Romania-evidence from the limb bones. *Hantkeniana* **5**, 20-26 (2006).

30 Vecchia, F. M. D. *Telmatosaurus* and the other hadrosaurids of the Cretaceous European Archipelago. An overview. *Nat. Nascosta* **32**, 1-55 (2006).

31 Arbulla, D. *et al.* Escursione nel Carso Triestino, in Slovenia e Croazia. 8 giugno. Stop 1. La successione Santoniano–Campaniana del Villaggio del Pescatore (Carso Triestino) nel quale sono stati rinvenuti i resti di dinosauro in *Guida alle escursioni/excursions guide* (eds. Melis, R., Romano, R., & Fonda, G.) (Societa Paleontologica Italiana - Giornate di Paleontologia, 2006).

32 Vecchia, F. M. D. I dinosauri del Villaggio del Pescatore (Trieste): qualche aggiornamento. *Atti Mus. Civ. Stor. Nat. Trieste* **53**, 111-130 (2008).

33 Xing, H. *et al.* A new basal hadrosauroid dinosaur (Dinosauria: Ornithopoda) with transitional features from the late cretaceous of Henan Province, China. *PLoS ONE* **9**, e98821, doi:10.1371/journal.pone.0098821 (2014).

34 Gates, T. A., Horner, J. R., Hanna, R. R. & Nelson, C. R. New unadorned hadrosaurine hadrosaurid (Dinosauria, Ornithopoda) from the Campanian of North America. *J. Vert. Paleontol.* **31**, 798-811, doi:10.1080/02724634.2011.577854 (2011).

35 Jinnah, Z. A. *et al.* New 40Ar-39Ar and detrital zircon U-Pb ages for the Upper Cretaceous Wahweap and Kaiparowits formations on the Kaiparowits Plateau, Utah: implications for regional correlation, provenance, and biostratigraphy. *Cretaceous Res.* **30**, 287-299, doi:10.1016/j.cretres.2008.07.012 (2009).

36 Cuthbertson, R. S. & Holmes, R. B. The first complete description of the holotype of *Brachylophosaurus canadensis* Sternberg, 1953 (Dinosauria: Hadrosauridae) with comments on intraspecific variation. *Zool. J. Linn. Soc.* **159**, 373-397, doi:10.1111/j.1096-3642.2009.00612.x (2010).

37 Rogers, R. R. *et al.* Age, correlation, and lithostratigraphic revision of the Upper Cretaceous (Campanian) Judith River Formation in its type area (North-Central Montana), with a comparison of low- and high-accommodation alluvial records. *The Journal of Geology* **124**, 99-135, doi:10.1086/684289 (2016).

38 Connor, C. W. The Lance Formation - petrography and stratigraphy, Powder River basin and nearby basins, Wyoming and Montana. *U.S. Geological Survey Bulletin* **1917**, 11-17, doi:10.3133/b1917I (1992).

39 McIver, E. E. The paleoenvironment of *Tyrannosaurus rex* from southwestern Saskatchewan, Canada. *Can. J. Earth Sci.* **39**, 207-221, doi:10.1139/e01-073 (2002).

40 Murphy, E. C., Hoganson, J. W. & Johnson, K. R. Lithostratigraphy of the Hell Creek Formation in North Dakota in *The Hell Creek Formation and the Cretaceous-Tertiary boundary in the northern Great Plains: An Integrated continental record of the end of the Cretaceous* (eds. Hartman, J. H., Johnson, K. R., & Nichols, D. J.) Vol. 361 9-34 (Geological society of America special paper, 2002).

41 Colson, M. C. Stratigraphy and depositional environments of the upper Fox Hills and lower Hell Creek Formations at the Concordia Hadrosaur Site in northwestern South Dakota. *Rocky. Mt. Geol* **39**, 93-111, doi:10.2113/39.2.93 (2004).

42 Bamforth, E. L., Button, C. L. & Larsson, H. C. E. Paleoclimate estimates and fire ecology immediately prior to the end-Cretaceous mass extinction in the Frenchman Formation (66Ma), Saskatchewan, Canada. *Palaeogeogr., Palaeoclimatol., Palaeoecol.* **401**, 96-110, doi:10.1016/j.palaeo.2014.02.020 (2014).

43 Ullmann, P. V., Shaw, A., Nellermoe, R. O. N. & Lacovara, K. J. Taphonomy of the Standing Rock Hadrosaur Site, Corson County, South Dakota. *Palaios* **32**, 779-796, doi:10.2110/palo.2017.060 (2017).

44 Eberth, D. A. & Braman, D. R. A revised stratigraphy and depositional history for the Horseshoe Canyon Formation (Upper Cretaceous), southern Alberta plains. *Can. J. Earth Sci.* **49**, 1053-1086, doi:<https://doi.org/10.1139/e2012-035> (2012).

45 Bell, P. R. & Campione, N. E. Taphonomy of the Danek Bonebed: a monodominant *Edmontosaurus* (Hadrosauridae) bonebed from the Horseshoe Canyon Formation, Alberta. *Can. J. Earth Sci.* **51**, 992-1006, doi:10.1139/cjes-2014-0062 (2014).

46 Bell, P. R., Fanti, F., Currie, P. J. & Arbour, V. M. A mummified duck-billed dinosaur with a soft-tissue cock's comb. *Curr. Biol.* **24**, 70-75, doi:10.1016/j.cub.2013.11.008 (2014).

47 Evans, D. C., Eberth, D. A., Ryan, M. J. & Therrien, F. Hadrosaurid (*Edmontosaurus*) bonebeds from the Horseshoe Canyon Formation (Horsethief Member) at Drumheller, Alberta, Canada: geology, preliminary taphonomy, and significance. *Can. J. Earth Sci.* **52**, 642-654, doi:10.1139/cjes-2014-0184 (2015).

48 Rogers, R. R. Sequence analysis of the Upper Cretaceous Two Medicine and Judith River formations, Montana; nonmarine response to the Claggett and Bearpaw marine cycles. *J. Sediment. Res.* **68**, 615-631, doi:10.2110/jsr.68.604 (1998).

49 Roberts, E. M., Sampson, S. D., Deino, A. L., Bowring, S. A. & Buchwaldt, R. The Kaiparowits Formation: a remarkable record of Late Cretaceous terrestrial environments, ecosystems, and evolution in western North America in *At the Top of the Grand Staircase: the late Cretaceous of Southern Utah* (eds. Titus, A. L. & Loewen, M. A.) 85-106 (Indiana University Press, 2013).

50 Eberth, D. A. The geology in *Dinosaur Provincial Park: A Spectacular Ancient Ecosystem Revealed* (eds. Currie, P. J. & Koppelhus, E. B.) 367-397 (Indiana University Press, 2005).

51 Prieto-Márquez, A., Weishampel, D. B. & Horner, J. R. The dinosaur *Hadrosaurus foulkii*, from the Campanian of the east coast of North America, with a reevaluation of the genus. *Acta Palaeontol. Pol.* **51** (2006).

52 Gallagher, W. B. Recent mosasaur discoveries from New Jersey and Delaware, USA: stratigraphy, taphonomy and implications for mosasaur extinction. *Netherlands Journal of Geosciences* **84**, 241-245, doi:10.1017/s0016774600021028 (2016).

53 Lauters, P., Bolotsky, Y. L., Van Itterbeeck, J. & Godefroit, P. Taphonomy and age profile of a latest Cretaceous dinosaur bone bed in Far Eastern Russia. *Palaios* **23**, 153-162, doi:10.2110/palo.2006.p06-031r (2008).

54 Van Itterbeeck, J., Bolotsky, Y., Bultynck, P. & Godefroit, P. Stratigraphy, sedimentology and palaeoecology of the dinosaur-bearing Kundur section (Zeya-Bureya Basin, Amur Region, Far Eastern Russia). *Geol. Mag.* **142**, 735, doi:10.1017/s0016756805001226 (2005).

55 Godefroit, P., Bolotsky, Y. L. & Lauters, P. A new saurolophine dinosaur from the latest cretaceous of Far Eastern Russia. *PLoS ONE* **7**, e36849, doi:10.1371/journal.pone.0036849 (2012).

56 Hunt, A. P. & Lucas, S. G. Stratigraphy, paleontology and age of the Fruitland Kirtland formations (Upper Cretaceous), San Juan Basin, New Mexico. *New Mexico Geological Society Guidebook* **43**, 217-239 (1992).

57 Williamson, T. Review of Hadrosauridae (Dinosauria, Ornithischia) from the San Juan Basin, New Mexico. *New Mexico Museum of Natural History and Science* **17**, 191-213 (2000).

58 Kirkland, J. I. *et al.* The Late Cretaceous Difunta Group of the Parras Basin, Coahuila, Mexico. *Universidad Aut´onoma del Estado de Hidalgo, Avances en Investigaci´on* **3**, 133-172 (2000).

59 Eberth, D. A. *et al.* Cerro del Pueblo Fm (Difunta Group, Upper Cretaceous), Parras Basin, southern Coahuila, Mexico: reference sections, age, and correlation. *Rev. Mex. Cienc. Geol* **21**, 335-352 (2004).

60 Schmitt, J. G., Jackson, F. D. & Hanna, R. R. Debris flow origin of an unusual late Cretaceous hadrosaur bonebed in the Two Medicine Formation of western Montana in *Hadrosaurs* (eds. Eberth, D. A. & Evans, D. C.) 486-501 (Indiana University Press, 2015).

61 Freedman Fowler, E. A. & Horner, J. R. A new brachylophosaurin hadrosaur (Dinosauria: Ornithischia) with an intermediate nasal crest from the Campanian Judith River Formation of northcentral Montana. *PLoS ONE* **10**, e0141304, doi:10.1371/journal.pone.0141304 (2015).

62 Kirschbaum, M. A. & Hettinger, R. D. Facies analysis and sequence stratigraphic framework of Upper Campanian strata (Neslen and Mount Garfield formations, Bluecastle Tongue of the Castlegate Sandstone, and Mancos Shale), eastern Book cliffs, Colorado and Utah. 46 (69-G, 2004).

63 Bell, P. R. *et al.* Sedimentological and taphonomic observations on the “Dragon's Tomb” *Saurolophus* (Hadrosauridae) bonebed, Nemegt Formation (Upper Cretaceous), Mongolia. *Palaeogeogr., Palaeoclimatol., Palaeoecol.*, doi:10.1016/j.palaeo.2017.11.034 (2017).

64 Eberth, D. A. Stratigraphy and paleoenvironmental evolution of the dinosaur-rich Baruungoyot-Nemegt succession (Upper Cretaceous), Nemegt Basin, southern Mongolia. *Palaeogeogr., Palaeoclimatol., Palaeoecol.* **494**, 29-50, doi:10.1016/j.palaeo.2017.11.018 (2018).

65 Eberth, D. A. *et al.* Dinosaur biostratigraphy of the Edmonton Group (Upper Cretaceous), Alberta, Canada: evidence for climate influence. *Can. J. Earth Sci.* **50**, 701-726, doi:10.1139/cjes-2012-0185 (2013).

66 Bonaparte, J., Franchi, M. R., Powell, J. E. & Sepulveda, E. La Formación Los Alamitos (Campaniano-Maastrichtiano) del sudeste de Rio Negro, con descripcion de *Kritosaurus australis* n. sp. (Hadrosauridae). Significado paleogeografico de los vertebrados. *Rev. Asoc. Geol. Argent.* **39**, 284-299 (1984).

67 Paredes, J. M., Foix, N. & Allard, J. O. Sedimentology and alluvial architecture of the Bajo Barreal Formation (Upper Cretaceous) in the Golfo San Jorge Basin: Outcrop analogues of the richest oil-bearing fluvial succession in Argentina. *Mar. Pet. Geol.* **72**, 317-335, doi:10.1016/j.marpetgeo.2016.02.013 (2016).

68 Kuang, H.-W. *et al.* Research on taphonomy of late Cretaceous dinosaurs in Zhucheng, eastern Shandong, China. *Acta Geologica sinica* **88**, 1353-1371 (2014).

69 He, B. *et al.* Soft-sediment deformation structures in the Cretaceous Zhucheng depression, Shandong Province, East China; their character, deformation timing and tectonic implications. *J. Asian Earth Sci.* **110**, 101-122, doi:10.1016/j.jseaes.2014.12.005 (2015).

70 Godefroit, P., Shulin, H., Tingxiang, Y. & Lauters, P. New hadrosaurid dinosaurs from the uppermost Cretaceous of northeastern China. *Acta Palaeontol. Pol.* **53**, 47-74, doi:10.4202/app.2008.0103 (2008).

71 Kordikova, E. G. *et al.* Small vertebrates from the late Cretaceous and early Tertiary of the northeastern Aral Sea region, Kazakhstan. *J. Paleontol.* **75**, 390-400, doi:10.1666/0022-3360(2001)075<0390:svftlc>2.0.co;2 (2001).

72 Pujalte, V. & Schmitz, B. Revisión de la estratigrafía del Grupo Tremp («Garumniense», Cuenca de Tremp-Graus, Pirineos meridionales). *Geogaceta* **38**, 79-82 (2005).

73 Riera, V., Oms, O., Gaete, R. & Galobart, À. The end-Cretaceous dinosaur succession in Europe: The Tremp Basin record (Spain). *Palaeogeogr., Palaeoclimatol., Palaeoecol.* **283**, 160-171, doi:10.1016/j.palaeo.2009.09.018 (2009).

74 Cruzado-Caballero, P., Canudo, J. I., Moreno-Azanza, M. & Ruiz-Omeñaca, J. I. New material and phylogenetic position of *Arenysaurus ardevoli*, a lambeosaurine dinosaur from the late Maastrichtian of Arén (northern Spain). *J. Vert. Paleontol.* **33**, 1367-1384, doi:10.1080/02724634.2013.772061 (2013).

75 Oms, O. & Canudo, J. I. Datación magnetoestratigráfica de los dinosaurios del Cretácico terminal (Maastrichtiense superior) de Arén (Huesca, Unidad Surpirenaica Central). *Geo-Temas* **6**, 51-54 (2004).

76 Cruzado-Caballero, P., Pereda-Suberbiola, X. & Ruiz-Omeñaca, J. I. *Blasisaurus canudoi* gen. et sp. nov., a new lambeosaurine dinosaur (Hadrosauridae) from the latest Cretaceous of Arén (Huesca, Spain). *Can. J. Earth Sci.* **47**, 1507-1517, doi:10.1139/e10-081 (2010).

77 Vecchia, F. M. D. *et al.* The hadrosauroid record in the Maastrichtian of the eastern Tremp Syncline (northern Spain) in *Hadrosaurs* (eds. Eberth, D. A. & Evans, D. C.) 298-314 (Indiana University Press, 2015).

78 Laurent, Y., Bilotte, M. & Le Loeuff, J. Late Maastrichtian continental vertebrates from southwestern France: correlation with marine fauna. *Palaeogeogr., Palaeoclimatol., Palaeoecol.* **187**, 121-135, doi:10.1016/s0031-0182(02)00512-6 (2002).

79 Prieto-Márquez, A., Dalla Vecchia, F. M., Gaete, R. & Galobart, A. Diversity, relationships, and biogeography of the lambeosaurine dinosaurs from the European Archipelago, with description of the new Aralosaurin *Canardia garonnensis*. *PLoS ONE* **8**, e69835, doi:10.1371/journal.pone.0069835 (2013).

80 Godefroit, P. *et al.* Recent advances on the study of hadrosaurid dinosaurs in Heilongjiang (Amur) River area between China and Russia. *Global Geology* **14**, 160-191 (2011).

81 Varricchio, D. J. *et al.* Tracing the Manson impact event across the Western Interior Cretaceous Seaway. *Geological Society of America Special Papers* **465**, 269-299, doi:10.1130/2010.2465(17) (2010).

82 Renne, P. R., Fulford, M. M. & Busby-Spera, C. High resolution 40AR/39AR chronostratigraphy of the Late Cretaceous El Gallo Formation, Baja California del Norte, Mexico. *Geophys. Res. Lett.* **18**, 459-462, doi:10.1029/91gl00464 (1991).

83 Nagao, T. *Nipponosaurus sachalinensis*: a new genus and species of trachodont dinosaur from Japanese Saghalien. *Journal of the Faculty of Science, Hokkaido Imperial University. Series 4, Geology and mineralogy* **3**, 185-220 (1936).

84 Godefroit, P., Bolotsky, Y. L. & Bolotsky, I. Y. Osteology and relationships of *Olorotitan arharensis*, a hollow-crested hadrosaurid dinosaur from the latest Cretaceous of Far Eastern Russia. *Acta Palaeontol. Pol.* **57**, 527-560, doi:10.4202/app.2011.0051 (2012).

85 Fassett, J. E. & Hinds, J. S. Geology and fuel resources of the Fruitland Formation and Kirtland Shale of the San Juan Basin, New Mexico and Colorado. *U.S. Geological Survey Professional Paper* **676**, 1-76 (1971).

86 Tapanila, L. & Roberts, E. M. Continental invertibrates and trace fossils from the Campanian Kaiparowits Formation, Utah in *At the Top of the Grand Staircase: the late Cretaceous of Southern Utah* (eds. Titus, A. L. & Loewen, M. A.) 132-152 (Indiana University Press, 2014).

## Supplementary Table S7. AIC values of ASR analyses

| Branch length calculation | Model | AIC values |
| --- | --- | --- |
| Basic | ER | 67.37412 |
| Basic | SYM | 67.37412 |
| Basic | ARD | 57.36662 |
| Equal | ER | 63.98659 |
| Equal | SYM | 63.98659 |
| Equal | ARD | 60.73556 |
| Additive | ER | 68.29923 |
| Additive | SYM | 68.29923 |
| Additive | ARD | 58.47383 |
| Zero-branch length additive | ER | 68.40706 |
| Zero-branch length additive | SYM | 68.40706 |
| Zero-branch length additive | ARD | 58.47383 |
| Minimum branch length | ER | 68.67199 |
| Minimum branch length | SYM | 68.67199 |
| Minimum branch length | ARD | 58.47938 |

# Supplementary Data

## Supplementary Data S1. R script of age retrocalculation and tibia growth curve reconstruction.

gm <- read.csv("mukawa.csv")

mn <- min(grep("gm.c", colnames(gm))) # column number of the frist growth mark

mx <- max(grep("gm.c", colnames(gm))) # column number of the last growth mark

# age estimation ---------------------------------------------------------------

# par(mfrow = c(1, 1), mar = c(2.5, 2.5, 1.5, 0.3), mgp = c(1.5, 0.5, 0))

for (i in 1:1) {

# data manipulation for Ford-Walford plot

gm.rc <- data.frame(NULL)

gm.temp <- na.omit(t(gm[i, mn:mx]))

gm.rc <- rbind(gm.rc, cbind(transform(gm$taxa[i]),

gm.temp[1:nrow(gm.temp)-1], gm.temp[2:nrow(gm.temp)]))

colnames(gm.rc) <- c("taxa", "t", "t1")

# Ford-Walford plot

plot(1, 1, xlab = "tibia cirucmference at time t (mm)",

ylab = "tibia cirucmference at time t + 1 (mm)", type = "n",

xlim = c(min(gm.rc$t), max(gm.rc$t)), ylim = c(min(gm.rc$t1), max(gm.rc$t1)))

gm.temp <- na.omit(t(gm[i, mn:mx]))

gm.temp <- cbind(transform(gm$taxa[i]),

gm.temp[1:nrow(gm.temp)-1], gm.temp[2:nrow(gm.temp)])

points(gm.temp[,2], gm.temp[,3], col = c("#201F1E90"), type = "o",

lwd = 2, pch = 16)

# model fitting on Ford-Walford plot

sha.par <- c(0, 2/3, .99, 2, 4, 8.4) # shape parameters

sha.par.name <- c("mono", "vonb", "gomp", "logi", "extr", "inno") # model names

gm.rc <- cbind(gm.rc, m = NA)

aic.bic <- data.frame(NULL)

coef <- data.frame(NULL)

A.ini <- max(gm$outermost[i])

K.ini.temp <- -log((gm.rc[, 3] - A.ini)/(gm.rc[,2] - A.ini))

K.ini <- mean(K.ini.temp[!is.nan(K.ini.temp)][!is.infinite(K.ini.temp)])

# K = - ln((y(t+1)-A)/(y(t)-A)) (according to process error model)

for (j in 1:length(sha.par)) {

gm.rc$m <- sha.par[j]

fit <- NULL

fit <- try(nls(t1 ~ A * (1 + exp(- K) * ((t / A)^(1 - m) - 1))^(1 / (1 - m)),

data = gm.rc, start = list(A = A.ini, K = K.ini), trace = TRUE))

if (class(fit)[1] != "try-error") {

aic.bic.temp <- data.frame(AIC = AIC(fit), AICc = AICc(fit),

BIC = BIC(fit))

aic.bic <- rbind(aic.bic, aic.bic.temp)

coef.temp <- t(data.frame(coef(fit)))

coef <- rbind(coef, coef.temp)

} else {

aic.bic.temp <- data.frame(AIC = NA, AICc = NA, BIC = NA)

aic.bic <- rbind(aic.bic, aic.bic.temp)

coef.temp <- data.frame(A = NA, K = NA)

coef <- rbind(coef, coef.temp)

}

}

aic.bic <- cbind(aic.bic, dAICc = aic.bic$AICc - min(na.omit(aic.bic$AICc)))

aic.bic <- cbind(aic.bic, weight = exp(- 1 / 2 * aic.bic$dAICc) /

sum(exp(- 1 / 2 * na.omit(aic.bic$dAICc))))

coef <- cbind(coef, m = sha.par)

row.names(coef) <- c(sha.par.name)

row.names(aic.bic) <- c(sha.par.name)

# averaging model using AIC weights

coef <- rbind(coef, aver = c(sum(as.vector(na.omit(coef$A)) *

as.vector(na.omit(aic.bic$weight))), sum(na.omit(coef$K) *

na.omit(aic.bic$weight)), sum(coef$m[is.na(coef[, 1]) != 1] *

na.omit(aic.bic$weight))))

aic.bic <- rbind(aic.bic, aver = c(NA, NA, NA, NA, NA))

assign(paste0("aic.bic", ".", gm$taxa[i]), aic.bic)

# write.csv(aic.bic, "Table2.csv", quote = FALSE, row.names = F)

colour <- rbind("#FFB74C", "#B9C42F", "#008000", "#0090A8", "#233B6C",

"#F58F98", "#ED1A3D")

row.names(colour) <- c(sha.par.name, "aver")

for (j in 1:nrow(coef)) {

A <- coef[j, 1]

K <- coef[j, 2]

m <- coef[j, 3]

curve(A * (1 + exp(- K)*((x / A)^(1 - m) - 1))^(1 / (1 - m)), add = T,

col = colour[j], bty = "n", lwd = 2)

}

legend("topleft", legend = c(sha.par.name, "aver"), pch = - 1, lty = 1, lwd = 1,

col = colour, bty = "n")

legend("bottomright", legend = c("growth marks"), bty = "n",

col = "#201F1E90", pch = 16, lty = 1)

# age retrocalculation

age.est <- data.frame(NULL)

rtc <- data.frame(x = 1:(mx - mn + 1), y = as.vector(t(gm[i, mn:mx])))

rtc <- na.omit(rtc)

A0 <- 25 # neonatal size

# pick a shape parameter of the lowest AIC

# coef.temp <- coef[which(aic.bic$dAIC == 0), ]

plot(rtc, xlab = "growth mark count", ylab = "tibia cirucumference (mm)",

col = c("#201F1E90"), pch = 16, type = "o", lwd = 3,

xlim = c(- log(((1/A)^(1 - m)-1)/(m-1)) / K - (1/K) *

log(-((m-1)*A^(1-m))/(A^(1-m) - A0^(1-m))) - 7, nrow(rtc) + 1),

ylim = c(0, max(rtc$y)), main = gm$taxa[i])

abline(h = A0, col = "grey20", lty = 3, lwd = 2)

legend("topleft", legend = c(sha.par.name, "aver", "growth marks",

"hatchling size"), pch = c(- 1, - 1, - 1, - 1, - 1, - 1, - 1),

lty = c(1, 1, 1, 1 ,1, 1, 1, 1, 3), lwd = 1, bty = "n",

col = c(colour, "#201F1E90", "grey20"))

for (j in 1:nrow(coef)) {

A <- coef$A[j]

K <- coef$K[j]

m <- coef$m[j]

# par(mfrow = c(7, 5), mar = c(2.5, 2.5, 1.5, 0.3), mgp = c(1.5, 0.5, 0))

for (k in 0:30) {

retro <- try(nls(y ~ A * (1 + (m - 1)* exp(- K *((x + missing.age) +

(1 / K) * log( - ((m - 1) * A^(1 - m))/(A^(1 - m) -

A0^(1 - m))))))^(1 / (1 - m)), # I is replaced with the eqaution

#with A0 (I <- -(1/K) * log(-((m-1)*A^(1-m))/(A^(1-m) - A0^(1-m)))

# simple parameter calculation (this can be derived from f(0) = A0)

data = rtc, start = list(missing.age = k), trace = TRUE))

if (class(retro) != "try-error") break

if (class(retro) == "try-error") next

}

if (class(retro) != "try-error") {

missing.age <- as.numeric(coef(retro))

# double optimization

do.eq <- function(params, age) {

A <- params[1]

m <- params[2]

K <- params[3]

A0 <- params[4]

missing.age <- params[5]

size <- A * (1 + (m - 1) * exp(- K *((age + missing.age) +

(1 / K) * log(-((m - 1) * A^(1 - m))/(A^(1 - m) -

A0^(1 - m))))))^(1 / (1 - m))

# I is replaced with the eqaution with A0 (I <- -(1/K) *

# log(-((m-1)*A^(1-m))/(A^(1-m) - A0^(1-m)))

# simple parameter calculation (this can be derived from f(0) = A0)

return(size)

}

sRSS <- function(params, model, age, size){

sum((size - model(params, age))^2)

}

do.par <- optim(c(A, m, K, A0, missing.age),

sRSS, model = do.eq, age = rtc[,1], size = rtc[,2], method = "L-BFGS-B",

lower = c(A - 0.00001, m - 0.00001, K - 0.00001, 0, 0),

upper = c(A + 0.00001, m + 0.00001, K + 0.00001, Inf, missing.age * 2),

control = list(maxit = 2000))

A0 <- do.par$par[4]

missing.age <- do.par$par[5]

age.est <- rbind(age.est, missing.age)

curve(A * (1+(m - 1)* exp(- K *((x + as.numeric(missing.age)) + (1 / K) *

log(-((m - 1) * A^(1 - m))/(A^(1 - m) - A0^(1 - m))))))^(1 / (1 - m)),

col = colour[j], lwd = 2, add = T)

} else {

missing.age <- NA

age.est <- rbind(age.est, missing.age)

}

}

A <- coef$A

K <- coef$K

m <- coef$m

coef <- cbind(coef, I = -(1/K) * log(-((m-1)*A^(1-m))/(A^(1-m) - A0^(1-m))))

assign(paste0("coef", ".", gm$taxa[i]), coef)

rownames(age.est) <- rownames(coef)

colnames(age.est) <- "age.est"

assign(paste0("age.est", ".", gm$taxa[i]), age.est)

# write.csv(age.est, "age.est.mono.csv", quote = FALSE, row.names = F)

}

# tibia growth curve comparison ------------------------------------------------

colour.taxa <- rbind("#9be15d", "#00e3ae", "#01baef", "#00e3ae", "#ffb400")

# age estimation from averaged model

age <- c(1:(mx - mn + 1)) + age.est[rownames(age.est) == "mono", ]

grw.tib <- data.frame(age, gm = na.omit(as.vector(t(gm[i, mn:mx]))))

assign(paste0("grw.tib", ".", gm$taxa[i]), grw.tib)

plot(1, type = "n", xlim = c(0, 20), ylim = c(0, 400),

xlab = "age (year)", ylab = "tibia growth mark circum (mm)",

main = "tibia growth")

grw.tib <- eval(parse(text = paste0("grw.tib", ".", gm$taxa[i])))

points(grw.tib, col = c("#201F1E90"), pch = 16, type = "o", lwd = 3)

coef <- eval(parse(text = paste0("coef", ".", gm$taxa[i])))

A <- coef[rownames(coef) == "mono", ]$A

K <- coef[rownames(coef) == "mono", ]$K

I <- coef[rownames(coef) == "mono", ]$I

m <- coef[rownames(coef) == "mono", ]$m

curve(A * (1 + (m - 1) * exp(- K * (x - I)))^(1 / (1 - m)), add = TRUE,

col = colour.taxa[i], bty = "n", lwd = 2)

# export data ------------------------------------------------------------------

age.est <- eval(parse(text = paste0("age.est", ".", gm$taxa[i])))

aic.bic <- eval(parse(text = paste0("aic.bic", ".", gm$taxa[i])))

coef <- eval(parse(text = paste0("coef", ".", gm$taxa[i])))

tib.grw <- cbind(age.est + 1, aic.bic, coef)

write.csv(tib.grw, paste0("tib.grw", ".", gm$taxa[i], ".csv"),

quote = FALSE, row.names = F)

## Supplementary Data S2. Data matrix used for the phylogenetic analysis in this study.

#NEXUS

[written Thu Feb 28 18:09:10 JST 2019 by Mesquite version 3.2 (build 801) at DESKTOP-V0460LR/192.168.11.15]

BEGIN TAXA;

TITLE Taxa;

DIMENSIONS NTAX=70;

TAXLABELS

Ouranosaurus_nigeriensis Bactrosaurus_johnsoni Claosaurus_agilis Eolambia_caroljonesa Eotrachodon_orientalis Equijubus_normani Gilmoreosaurus_mongliensis Iguanodon_bernissartensis Jintasaurus_meniscus Jinzhousaurus_yangi Levnesovia_transoxiana Mantellisaurus_atherfieldensis Nanningosaurus_dashiensis Nanyangosaurus_zhugeii Probactrosaurus_gobiensis Sirindhorna_khoratensis Tanius_sinensis Telmatosaurus_transsylvanicus Tethyshadros_insularis Xuwulong_yueluni Yunganglong_datongensis Zhanghenglong_yangchengensis Zuoyunlong_huangi Acristavus_gagslarsoni Brachylophosaurus_canadensis Edmontosaurus_annectens Edmontosaurus_regalis Gryposaurus_latidens Gryposaurus_monumentensis Gryposaurus_notabilis Hadrosaurus_foulkii Kerberosaurus_manakini Kritosaurus_navajovius Lophorhothon_atopus Maiasaura_peeblesorum Probrachylophosaurus_bergei Prosaurolophus_maximus Rhinorex_condrupus Saurolophus_angustirostris Saurolophus_osborni Secernosaurus_koerneri Shantungosaurus_giganteus Wulagasaurus_dongi Amurosaurus_riabinini Aralosaurus_tuberiferus Arenysaurus_ardevoli Charonosaurus_jiayinensis Corythosaurus_casuarius Corythosaurus_intermedius Hypacrosaurus_altispinus Hypacrosaurus_stebingeri Jaxartosaurus_aralensis Lambeosaurus_lambei Lambeosaurus_magnicristatus Magnapaulia_laticauda Olorotitan_arharensi Pararhabdodon_isonense Parasaurolophus_cyrtocristatus Parasaurolophus_tubicen Parasaurolophus_walkeri Tsintaosaurus_spinorhinus Velafrons_coahuilensis Nipponosaurus_sahalinensis Canardia_garonnensis Blasisaurus_canudoi Sahaliyania_elunchunorum Laiyangosaurus_youngi Plesiohadros_djadokhtaensis Adynomosaurus_arcanus Kamuysaurus_japonicus

;

END;

BEGIN CHARACTERS;

TITLE Character_Matrix;

DIMENSIONS NCHAR=350;

FORMAT DATATYPE = STANDARD GAP = - MISSING = ? SYMBOLS = " 0 1 2 3 4 5 6";

MATRIX

Ouranosaurus_nigeriensis 0000000000000000000000?200100000000??01002010000000000000000000000000000?00000000000000000??0?00?000000010000003200?00000000000000000000000001000000020000001000001??00??000000000011000000?0000000000???01000000010000000100000100011001000100?11102000010000010000000000000000000000000000000120000000000100000000000000000000000001100000000000?100000000??

Bactrosaurus_johnsoni 0111{1 2}100212?1110{0 1}111{0 1}010001000011010001211101{0 1}00000000011011001000111110000000110000000000??00?0{0 1}101001000010010110000000200011?011100000000000000?100?00000?0?0000??00??1000000000000010101000000000000000001000011111000100??000000??0100?001??00011?10002000000010011000000{0 1}0010??101???1??1020011100011000001000001001110011111001201011111000?1000010001?

Claosaurus_agilis ????1300??1????????11?1????????????????????????????????????????????????????????????????????????????????????????????????????????????????????????????????????????????????????????????????????????????????????????????????????????????????????????????0?1?????????00000002000000000000???????????0?111112?00?1111?0???????????????????????????????????1110??????0

Eolambia_caroljonesa 01110{2 3}001110111001010011000000111?10?00102101{0 1}000000000110000010000?1111?00000000000000000??00?001010?00?00?0010100?00000100020?0000000?0000000?000100?0000010?0000??00????????00000000?000000000000000?001000000111010000100????000????110??01?0000?01000020000010000000000000001???110?0?1??0000000000011000000000000000010011101001100000001000?1000?0000??

Eotrachodon_orientalis ?1??12001110111?01?1101101110001101000010110100001010101101101100011??10030000110000000000??000101?2001001{0 1}101112100001012?0021?011111?10000000??121001000001011210??00??1000000?0011001?10010000000000??0100010011??11000100??000000001??1?001?000?????????????????????????????????????????1?????????????????????11?0?01101?210112?01?000????1???????????001?

Equijubus_normani 00110000??000??00??00000001000000000000002?010000?0?0???000000100?010000000000010000000000??000000010??0000100000?000000010001?000000000000000000021000000001000000??00??1000000?00000000001??00000000???00?0?0?0001???0???0???00000?001?00000100??0?0????????????????????????????????????????000000000000000??0??????????????????????????????????????????00??

Gilmoreosaurus_mongliensis 01112???????11?001111010??0?0001101??0??010010???0?0000110???????????????????????????????????0???10200101001?011110??0000??00???00110001??00000000???????000?0????????0??100???0?00????????????????00????????????????????????????????????????01??00??????002000000??001100000000010??1????????1021011100{0 1}120100010?????001?1?011111001100011111000?10000000??0

Iguanodon_bernissartensis 000000000000000001000000{0 1}00000000000000110000000000000000000000000000000000000000000000000??00000000000000000000000000000100000000000000000000000000000000001000000??00??00000000000000100010000000000???010000000000000??10000000000000000000000001100000000000000000000000000000000000000000100{0 1}0000000001000000100000000000000000001000000000000011000000??

Jintasaurus_meniscus ????????????????????????????????????????????????????????????????????????????????????????????????????????????????????????????????????????0?0?0??????1?????????????????????????????0011001?0010000000000000110000101110?000?100????00?1??1100?????????????????????????????????????????????????????????????????????????????????????????????????????????????????1?

Jinzhousaurus_yangi 00000000??00?0000??0000??0?00????0?0000102??0?00??0??0?0000000000?0???0?100000?00000000000??0?0?0?000?0000010000??00000002000000000?0?00000000000001001000001010000??00??00000000000000000010000000000???00000??????????????000000001011?0?000000000100000000000000000000000000000000000000000000000000000010000000000000000000000000110000000000001000000?0?0

Levnesovia_transoxiana 00??110?111011100?11101??010000???1??001011010???????00?10110010000??????????????????????????0??0?0??010100?0010110?00000???001??111?00???????????????????????????????????00000000011001?100000000000000001001000011110000100????00?0??1100????????0?1????0100??????????00000??0???????????????????101000??????????00??011?1??????????????1??1??????????100?1?

Mantellisaurus_atherfieldensis 00000000000000000{0 1}000000000000000000001101000000000000000000000000000000000000000000000000??00000000000000000000000000000100000010000000000000000000001000001000000??00??00000000000000100010000000000???000000000100000??100000100010000000000000001000000000000000000000000000000000000000000{0 1}0{0 1}0000000000000000000000000000000000001000000000000000000000??

Nanningosaurus_dashiensis 01??1101111?11100?1110???????????????????2????011101100110?????????1?????????????????????????0?0000101100001?11111???????????????????11????????????????????????????????????????????????1?101??????????????????????1?1????????????????????????11?????????????????????0010?????????????????????????????????????????????????????100001001?00011111???????????0???

Nanyangosaurus_zhugeii ?????????????????????????????????????????????????????????????????????????????????????????????????????????????????????????????????????????????????????????????????????????????????????????????????????????????????????????????????????????????????001110?????????????????00100011010?11000111?????????????????????????????????????????1?0001111100011000010???0

Probactrosaurus_gobiensis 00111100210011100??1?0?000?000011?1??01101101?0000000001100??0????0???00000000?00000000000??000???????0??0??0??00?00000000??0?0?1000?00???000000???????00000?0???????00????????0?00???00?00?0000000000???0{0 1}000??0??????????0???0000000?1?00??????000?0?000010000000000000000000001??01???001??0?0000000000000000100000?00???0001001001100000000?????00000000??

Sirindhorna_khoratensis 001?01001100011001?1001??010??0???0000000200000000000000100??0100001??0000000011000?????0????00000?100000001000000?0000?01?0010??????00?0?000?????0??0?00?00?0???????0???????????000?000?0010000?00000???00000000?110?0000100??000001??1?00???????????????????????????????????????????????????????????????????????????????????????????????????????????????000?

Tanius_sinensis ??????????????????????????????????????????????????????????????????????????????????????????????????????????????????0?00000000011?01??0???0000?00??????????????0???????????????0?0?0011001010?0?0000000000?0100100001111000?10000?00000??0100?101???0?1??????????100000000000000100?????????????10200111000111100000????????????????????????10???1???????????01?

Telmatosaurus_transsylvanicus 011?1200??0?11101?11100??????????????00000011000110100111011011010111111000000010000000000??00??010200101101002111?0?0000???001?01??01110000000???010?1000001000000??00????????0?00???????0??0000000000??00?0??1??11111???100????0000001?1??011????????????????????????????????????????????????????????????????????????????????????????????????0??????????0?1?

Tethyshadros_insularis 01111101??0011101111100?001000011?1000?00???1?001?0???111?1101101?11111?000000010000000000??00??01021?1011?10?2???000??0000002?001111?11000000000001001000001000000??00??1000000000110010100?000000000????0000??0111???????????000000101???00110000111110001000100010???00000000?10111??0011111?2111320001?10120??10010011110??111?0?101??11??1???11001?1000?1

Xuwulong_yueluni 00110100??00111001?100100?100000000010010200100000000001100?001000010000000000110000000000??000000010??00001000?0100000002?001001000000000000000000?0000000010100?0??00??10000000000000100010000000000???000000000110??0???000?000000001100000100000??1???????????????????????????????????????000000000000000000??000000000100000010012000????????????????00??

Yunganglong_datongensis ?????????????????????????????????????????????????????????????????????????????????????????????????????????????????????????????????????????????????????????????????????????????????????????????????????????0??010?01110?????100????????????00??01??????????????????????????????????????????????????????????????????????????????????????1?000???01?????????????0?

Zhanghenglong_yangchengensis 01211210?11?11100111111??????????????01202011000???0011110???????????1???????????????????????0?01101001011010121210?012?12?0021???????????????????????????????????????????????????????????????????????????????????????????????????????????????1?1??????????????101000010?????????1????????????????????????????????????????????????????????????????????????0???

Zuoyunlong_huangi ????????????????????????????????????????????????????????????????????????????????????????????????????????????????????????????????????????????????????????????????????????????????????????????????????????????????????????????????????????????????????????????????????????????????????????????????000000000000000010?????????????????????000????????????????????

Acristavus_gagslarsoni 1122131??12?11111121111??????????????21{1 2}00011101110111121011110010111112?11101?00000000000??00?1010210211111112121001121113120110011111100000001?10001?0000000?11?0??00??1000000100{0 1}0001110?1200000000?1010020111011110100200???100?0??0111??????????????????????????????????????1????????????????????????????????1????11101??????????????1111????????????00??

Brachylophosaurus_canadensis 11222311212?111111211{0 1}120121112120211312{0 1}{1 2}1111011111111210111100101111121111011{0 1}0000000000??00110102102111111122211011211231{1 2}0110111011101100301112001{1 2}000000011101??00??1000010100{0 1}{0 1}00111001110111001?2010020111011110100200101100000101111111100011111111211210111102100101011012111101111111120122201112111211111300111111{0 2}11112{0 1}1111??11111{0 1}0011000011000?

Edmontosaurus_annectens 213313102121111211211?21{0 1}111?111302113100{0 1}0101122101111211111200101111121401111{0 1}1000000000??00111012102111111123211101201020211101111111000000022121020000001011210??00??20010001001211111021100000000010101201120111100001010111011010110110111000101111112112101111021001011111{0 1}1111111011111{0 1}2113221111211121111120011110{0 1}21211211101??1111100011000010001{0 1}

Edmontosaurus_regalis 2133231021211112112110210111?11130211{2 3}100{0 1}0101122101111211111{1 2}0010111112140111101000000000??00111102102111111123211101201020211101111111000000022121020000001011210??00??2001000100121111102110000000001010120112011110000101011101100111011011?0001011111121111011110210010112111111111101111102113221111211121111120011110021211111101??1111100011100?100011

Gryposaurus_latidens 10230311111011110?211????????????????202120111012101111211111100101111121301??100000000000???001120310211111012321???11001??121????????100000200110?12200000?0?1101??00???00???01?????0???02??0000?????????????????????????????10?00?????11?011????10111111211210100112100101{0 1}{1 2}1001??1????????101?112210112211211111301111?02??????????1???????1????????1001??

Gryposaurus_monumentensis 11231310??2?111201211020011101013121020221011101?10111121111120010111111130101100000000000??10011203102111110123211111101221221101110111000002001102222000001031101??00??20010001001100110021?0000000001?10?2011?011?10???10000111000021111???????????????????????????????????????????????????????????????????????????????????????????????????????????????011?

Gryposaurus_notabilis 11231310112?111101211??00021010131210{1 2}02{1 2}{0 1}011101210111121111120010111111130101100000000000??10011203102111110123211111101121121101110111000002001102222000001031101??00??200100010011001100211000000000101002011201111000010000101000011111101110001011111121121010011210010112101111111101111111111221111221??11111301?11?0221111201101??1111110011100?100111

Hadrosaurus_foulkii ?1??131?112???1????1101?????????????????????????????????????????????????????????????????????????????????????????2????????????????????????????????????????????????????????????????????????????????????????????????????????????????????????????????????????????1??????????00000110010???????????1?21132211112??12111?????11????21111??1?????111?1???????????0??1

Kerberosaurus_manakini 1????????????1?211211?????????????????????????01?101111211?????????1?1???????????00?00???????0??110210211111?12321110120{0 1}020211?0111111?0000000?21?202000000?0?1210??00???00100010011001?1021?00000000?1010020??2?111100?0100???0011?????11??????????1?1???????10?10112100101121111???????????0?2113221111211121111130011111121211201111??????????????????001?

Kritosaurus_navajovius 11232210??2?1111112110?00?1?1101212??1012001110121011112111111001?1111???30??????000000000??0001120210211111?12321111110022112110111011102100200110102?0000010?1101??00??20010001001100110021?00000000???{0 1}0020112011110000100?01010000?1111??11???????????????????????????????????????????????????????????2???????????????????????????????????????????????00?1

Lophorhothon_atopus ????22102120??1????1101??????????????????????????????????????????????????????????00000000????0????02???111??0??????10??00????????112?11?0000060????10??00000?0?1221??00???00?0001001100??1021?000000000?0??0???120111???????0?0110??0??1111??????00???????????????????????????????????????11?????????????????????????????????2121??????1??11?11000?1100010????

Maiasaura_peeblesorum 11222311212?111111211??2012111212021120211011101111111121011110010111112111101110000000000??00110102102111111122211011211231{1 2}01100110111012005011120011000000001101??00??1000010100110011100120013000001?1002011101111010020010110000000111?11110001111?111211?10111102100101011011111?01111111120122201112111211111300111111{0 2}1111201111??11111000110?0011010?

Probrachylophosaurus_bergei 11222311212?111211?11112??2???212????202211111011101111210111100?011?11??11???11?0000??0?????01101?210211111?1222110?1211131201?011101010110030?11?00??00000?0?1101??00???0000?010011001?1001110111001?2?10020111011110100?00????0000????11??11?000???1???????????????????????????????????????1?201222?11??11121111????111?1?011112?1?????????1100????????000?

Prosaurolophus_maximus {1 2}1232310112?1112112110?01121011131211110{0 1}001011{1 2}210111121111120010111112130101100000000000??00011{0 1}02102111110123211101200{0 1}20211101121111021006022121021000002021221??00??20010{0 1}010011011110211000000110101002011201111000000000110110{0 1}111111?11?0001011111121121011111210010112211111111101111102112{2 3}21011221121111131011120221211211111??111110001100111000?1

Rhinorex_condrupus 10231301??2?11110?21??????????????????????????0121?1111211?????????1?11?13?10110?000000000??1???12?31??111110??321111??01???121?0???????000002001102222000001021101??00??200100010011001100?1100000000?1?10020?????????????00??111000?11??1???????????????????????????????????????????????????????????????????????????????????????????????????????????????0?0?

Saurolophus_angustirostris 11231310??2?111211211??01121011131211{1 2}1000010111210111121111120010111111130101100001000100??00011102102111110123211101200020211101121111021004022121021000002021221??00??2000010110110111102121012001122?1002011201111?0??00000110110111111111111001011111121111011110210010{1 2}222101111??1011111021123211112211211?11300111?0021211211111??1111110011101110000{0 1}

Saurolophus_osborni 21232310112?1112112110?0113101113121?21000010111210111121111120010111111130101100001000100??00011102102111110123211101200020211101121111021004022121021000002021221??00??2000010110110111102121012001122?1002011201111?0??00000110110111111101111001011?111211110111102100101222101111??1011111021123211112211211?11300111??021211211111??1111110011101110000?

Secernosaurus_koerneri 1?2?2310??2?111111?110???1?1?????????1021111110?2101?112?11???????1111???????????????0???????0??12?3102??1????2121?????????????????????????????????2??????00?0???????0????0010001001200???0?1?000000?0??01002011201111000?000???????0??1?1???11??00?1??11111012101001121?????????1???1????????1111112200112111111111311111202211112011?1??11111????11?????0?1?

Shantungosaurus_giganteus 21331311??2?11121?211???0?2??????????110020111112101111211????????1111??140??????00000000????0?110?210211111??2321??????????????1111111?00000002?12102000000?0??210??00???00?00010012011?102110000000001012120112?111100??100???101100?1101??11?00011111111211210110102100101{1 2}21111??1????????102113221111211121111120011111021211201111??11111000?1100?100?1?

Wulagasaurus_dongi 11??????????11???????????????????????11101011?01?1?1111210???????????1?????????????????????????????????????1??????00?12???3??01?01111?1????????????????????????????????????????????????????????????????????????????????????????????????????????????????111121121011?1021001010110?????????????10??1222011?2?112111???????????21111201111???????0??????????????

Amurosaurus_riabinini 11232211212?11111121111??????????????1022211110111011112111111111111111112100010012201?1?111?1??101201311121?22321132121101020111001111110332?14?211?2?11111????00132?????12010?111110010111121111111112102010110111111000100?01????1?2??11??11??101?1?01112111112?1001011112222112??1????????1020132210112111211110100111110111112?01100?11111101?10???10001?

Aralosaurus_tuberiferus 11222211?11011111??110????????????????1?11????011101111211?????????1?????????????000000000???1??002201201101?12121111???11?020??11121??110110?0????0???00?00?0????10000???00000010011001?10?000000000000?0?010110?1????????????110001??1?1??????????????????????????????101?11?10????????????????????????????????????????????????????????????1????????????10??

Arenysaurus_ardevoli 112?2211211?111?1????????????????????100121101011101111211111111111111????????????????????????????????????2?????2????????????0??????????10?????????1??????????????????????12110?1101100101111211131111?2?0201011011111100?100???1?0?1??1111??11??10???0?1?1??1?100?1??2???????????????????????????????????????????101??1??????????????????1??1??????????????1?

Charonosaurus_jiayinensis 11233311212011111??111???????????????{0 1}02121110011101111211111111111111???????????1???????????1??11?10?311121??23211321211010201111111111103?2?????11???111?1????001???????12010?1101100112121212130112121020?0110011111001100??112??1??11111?111110111?011121111121110111111222201?1?1011??11110201322101120112111101001111?0111112001101111111111?10?1?100?0?

Corythosaurus_casuarius 112322112110111111211??01132111140210111{1 2}{0 1}111{0 1}0111011112111112111111111112100010011201110111?1??{0 1}012013111211223211321211{0 1}1021111000111110322?15?211?2{0 1}11111?1??0013211113120101111110010111121111111112102010110011111011000001120010201112011111011111111211111211101011112222112111111011111120132210112111211110100111110111112101200111111101110111100011

Corythosaurus_intermedius 112322112110111111211??01132111140210111{1 2}1111{0 1}0111011112111112111111111112100010011201110111?1??1012013111211223211321211{0 1}1021111000111110322?15?211?2{0 1}11111?1??00132111131201011111100101111211111111121020101100111110110000011200102011120111110111?111121111121110101111222211211111101111112013221011211121111010011111011111210120011111110111011110001?

Hypacrosaurus_altispinus 11233211212?11111121112?113221114021?1111{1 2}11110111011112111112111011111112100010011201210201?1??1012013111211223211321211{0 1}1021111000111110322?15?211?2111111?2??00132111131201011111100111111211111111121020101100111110100{0 1}0001120010101112111?11112101111211111211001011112222112111111011111120132210112111111110100111110111112101200111111101111111100111

Hypacrosaurus_stebingeri 112332{0 1}02110111111211??111321111402101011201110111011112111112111011111111100010011201110111?1??101201311121122321132121111021111000111110322?15?211?2111111?2??0013211113120101111110011111121111111112102010110011111011100??1120010201112?111?11121?1111211111211001011112222112???????????112013221011211121111010011111011111?101200111111?01?11111100011

Jaxartosaurus_aralensis ????????????????????????????????????????????????????????????????????????????????????????????????????????????????????????????????????????10????????11????11?1????001???????11????10011001?1021011111110??100010110?111??0???00???10??1??1?11???????????????????????????????????????????????????????????????????????????????????????????????????????????????????

Lambeosaurus_lambei 1123{2 3}21121101111112111101?2101114021011111011{0 1}0111011112111112111111111112100010012201211112?1??0012013111211223211321211{0 1}1020111001111110332?14?211?2111111?1??001321121312010111111001011112111111111210201011001111100011000112001020111201111101110111121111121100101111??22112111111011111{0 1}201322101121112111101?0111111111112101100111111101110111100011

Lambeosaurus_magnicristatus 11?3?211????11111121111?112?01114021011111??11011?0?1112111111111?11111112100010012201211112?1??00120131112112232?132121101021111001111110332?14?211?2111111?1??0013211213120101111110010111121111111112?02?10110011???????1???1120010201112?11?110????11??????11211001011112222112111??1011111020132210112111211?101001111?1111112?012001111111??110???1000?1

Magnapaulia_laticauda 11?3221121??11111?211????????????????????????????????112?1??????????11111200001001?????1?10??1??001201311121??232113212111?02???????????103??????21??2?111?1????001?????????????1?????????????1??????????????????????????????????????????????11?1?1?2???1112111??????01?111121221?????????????1020132210112?1??111101?01111101111121001011?????????1??????00??

Olorotitan_arharensi 11?3221121???11111211??1113111114?1?02111111000111011112111112111111111112100010011201110200?1??0011013111211223211421211010211?1??11??110322?1??211?2111111????0013??1??31201011???????????12111???1112??2???????11?????????0011200?021?1120111110111?11112111112111010111122221?????????????1120132210112111211??????1111??111112001100?11111???????????00?1

Pararhabdodon_isonense 11??????????11111????????????????0???100222101??????111211??????????11???????????????????????????0?10?4111?1?12321??????????????????????????????????????????????????????????????1????????????????????????????????????????????????????????????11????111?????????1?????010111122220????????????????????????????????????????????1????????????????????????????????

Parasaurolophus_cyrtocristatus 112222112110111???????????????????1???????????0??1?1?1121?1111????1111???????????13010000??0?1??1????1???????????????1?????????????????110332?04?211?2211121????001220100312010111011001?2121212110112?2?02010110011111001100??112??10?1?111?11?1101110????????10011101111112122011111??1?1???1021133210112011211110100111110100011001201111111111110???100?01

Parasaurolophus_tubicen 11233{2 3}11??2011111121111???????????1??112121110011101111211111111111111???????????13010000??0?1??1111013111210223211{2 3}21211010{2 3}0111111111110332?04?211?2211121????0012201003120101110110011{1 2}121212130112?2102010110011111001100??112??1??1111????????????????????????????????????????????????????????????0???????????01???111???????????????????????????????000?

Parasaurolophus_walkeri 11233???????111?1??111111121?1113011010202111?01?1?111121?11111111111111111000?0013010000200?1??1111013111?10?232?1221?11010301110?1111110332?04?211?2211121????00122010031201011101100111121212120112?2102010110011???????0???11200102111110111110111??11121111121110111111222201111101121111102013221011201121?1101001111?0111112001101111111???11?????00001

Tsintaosaurus_spinorhinus 1122221111101111112110?10121011140110{1 2}002221010{1 2}110111121111111{0 1}10111110110001100111000100?0?1??001101411121?123211?212?1110301?101{1 2}111110321?03?212?2?11111????0011001???11?00010011001?1021010001110??10201011011111100?100??110??10?11111?11?110111001112010100110010111{0 1}22220121?1011??1?11021132200112011{1 2}11110100111110111111001100111111101?1011110000?

Velafrons_coahuilensis 11??????????11?11??????11122?1114?1??012111111011101111211????????11111112100010011201010101?1??1012013111211122211321211110201?1100111?10322?15?211?2111111?1??001311111?12????111??001?1??1211111?1112?02010110??????????????11?001020?????????????????????????????01?1111?2221????1????????112013121011211111111????11111?1111121011001?????????10???1000??

Nipponosaurus_sahalinensis ?{0 1}{1 2 3}{2 3}{2 3}2101?????1?{0 1}?{0 1 2}110????????????????????????0{1 2}?1?1???11?111????????????????????????0???????1???{0 1}?????????????{1 2 3}?1?????????1?????????????????????????????1???????????1?????????????????????????????????????????????????????????????????????2?11?????????????{0 1}??0??1?001{0 1}?????????{0 1}??1{0 1}???01111??????{0 1 2 3}2101???111111???????????1?{0 1}?????1{0 1}00{0 1}11??{0 1}101?1{0 1}?111001?0

Canardia_garonnensis ?1?23200??????1?1?{1 2}1102???????????????????????????????????11?11??????????????????????????????1??012?1?311121??2321??????????????11121?1???????????????????????????????????12010????????????????????????????????????????????????????????????????????????1???????11??1???01011111???????????????????????????????????????????????????????????????????????????1???

Blasisaurus_canudoi 11233310?????11?1?211?1??????????????10012101?01110111111111?1111????????????????????????????????????????????????11?21111031?01??????????????????????????????????????????3???????????????????????????????????????????????????????????????????????????????????????????????????????1?????????????????????????????????????????????????????????????????????????1??

Sahaliyania_elunchunorum 11232211212?111?1?21111??????????????10222111101110111121111?11111111111??10001001?201?1?111?1??101?0?311121??2321132121101?20111101111110342?????11???111?1????0013??????12010?1??????1?111121111111112102011110111111000100?01????1?2??11??11??101?1?01112111112?1001011112222112??1?????1??0020132210112111211110100111110111112?01100?11111101?10???10001?

Laiyangosaurus_youngi {1 2}1?????????????{1 2}1??1002??????????????1101{0 1}010?01210111121111121011????1??40??110?????????????0??1{0 1}0210211111?123211101{1 2}000?0211?????????000000???1??02?00??0???12?0???????????????????????02??????????????????????????????????????????????????????????????????????????????????????????????????????????????????????????????????????????????????????????????00??

Plesiohadros_djadokhtaensis 11??1200??1011100?1110?0012100013111?11001111000010111111?????100??1??1??40001110????????????0?0100??0???????????1??????1000??100112?001??????????????????????????????????00000??0011001?1001?00000000??????0?0?011111?0??20000???00?????10?????????????????????????????????????????1101?01?????????????????????????????????????????????????????00?1?0111?00??

Adynomosaurus_arcanus ?1??2?????????1???????????????????????????????01???111121??????????????????????????????????????????????????????????????????????????????????????????????????????????????????????????????????????????????????????????????????????????????????????????????0???????1??000010?????????????????????????01?320?1?2?????1????????????200000000000011???0????1?????????

Kamuysaurus_japonicus ?1??33112121111?1??1112??????????????11010111111110111121?11120011?1?1?????????????????????????????????????????3211111101030211101111111??????????????????????????????????000?0??001?0???00?11101?0000?????0?01?211?1???????0?0100010??111?1?11?100???1?111111110110112100101020111??111?0??1?0?????22111???112111??????????????1????1????111010?0??111010?011

;

END;

BEGIN TREES;

Title 'Trees from "results.nex"';

ID 0168df071ab91;

LINK Taxa = Taxa;

TRANSLATE

[0] 1 Ouranosaurus_nigeriensis,

[1] 2 Bactrosaurus_johnsoni,

[2] 3 Claosaurus_agilis,

[3] 4 Eolambia_caroljonesa,

[4] 5 Eotrachodon_orientalis,

[5] 6 Equijubus_normani,

[6] 7 Gilmoreosaurus_mongliensis,

[7] 8 Iguanodon_bernissartensis,

[8] 9 Jintasaurus_meniscus,

[9] 10 Jinzhousaurus_yangi,

[10] 11 Levnesovia_transoxiana,

[11] 12 Mantellisaurus_atherfieldensis,

[12] 13 Nanningosaurus_dashiensis,

[13] 14 Nanyangosaurus_zhugeii,

[14] 15 Probactrosaurus_gobiensis,

[15] 16 Sirindhorna_khoratensis,

[16] 17 Tanius_sinensis,

[17] 18 Telmatosaurus_transsylvanicus,

[18] 19 Tethyshadros_insularis,

[19] 20 Xuwulong_yueluni,

[20] 21 Yunganglong_datongensis,

[21] 22 Zhanghenglong_yangchengensis,

[22] 23 Zuoyunlong_huangi,

[23] 24 Acristavus_gagslarsoni,

[24] 25 Brachylophosaurus_canadensis,

[25] 26 Edmontosaurus_annectens,

[26] 27 Edmontosaurus_regalis,

[27] 28 Gryposaurus_latidens,

[28] 29 Gryposaurus_monumentensis,

[29] 30 Gryposaurus_notabilis,

[30] 31 Hadrosaurus_foulkii,

[31] 32 Kerberosaurus_manakini,

[32] 33 Kritosaurus_navajovius,

[33] 34 Lophorhothon_atopus,

[34] 35 Maiasaura_peeblesorum,

[35] 36 Probrachylophosaurus_bergei,

[36] 37 Prosaurolophus_maximus,

[37] 38 Rhinorex_condrupus,

[38] 39 Saurolophus_angustirostris,

[39] 40 Saurolophus_osborni,

[40] 41 Secernosaurus_koerneri,

[41] 42 Shantungosaurus_giganteus,

[42] 43 Wulagasaurus_dongi,

[43] 44 Amurosaurus_riabinini,

[44] 45 Aralosaurus_tuberiferus,

[45] 46 Arenysaurus_ardevoli,

[46] 47 Charonosaurus_jiayinensis,

[47] 48 Corythosaurus_casuarius,

[48] 49 Corythosaurus_intermedius,

[49] 50 Hypacrosaurus_altispinus,

[50] 51 Hypacrosaurus_stebingeri,

[51] 52 Jaxartosaurus_aralensis,

[52] 53 Lambeosaurus_lambei,

[53] 54 Lambeosaurus_magnicristatus,

[54] 55 Magnapaulia_laticauda,

[55] 56 Olorotitan_arharensi,

[56] 57 Pararhabdodon_isonense,

[57] 58 Parasaurolophus_cyrtocristatus,

[58] 59 Parasaurolophus_tubicen,

[59] 60 Parasaurolophus_walkeri,

[60] 61 Tsintaosaurus_spinorhinus,

[61] 62 Velafrons_coahuilensis,

[62] 63 Nipponosaurus_sahalinensis,

[63] 64 Canardia_garonnensis,

[64] 65 Blasisaurus_canudoi,

[65] 66 Sahaliyania_elunchunorum,

[66] 67 Laiyangosaurus_youngi,

[67] 68 Plesiohadros_djadokhtaensis,

[68] 69 Adynomosaurus_arcanus,

[69] 70 Kamuysaurus_japonicus;

TREE tnt_1 = (1,((10,(6,(16,(20,((21,(4,(9,(11,(17,(2,(7,(3,(13,((5,(22,(68,(69,(14,((31,(((24,43),(35,(25,36))),((((42,(26,27)),(70,(32,67))),(34,(37,(39,40)))),(33,(41,((28,38),(29,30))))))),(45,(((52,(46,((56,(((44,66),(53,54)),(55,(62,((48,49),(50,51)))))),(58,(60,(47,59)))))),(57,61)),(64,(63,65)))))))))),(18,19))))))))))),(15,23)))))),(8,12)));

TREE tnt_2 = (1,((10,(6,(16,(20,((21,(4,(9,(11,(17,(2,(7,(3,(13,((5,(22,(68,(69,(14,((31,((43,(24,(35,(25,36)))),((((42,(26,27)),(70,(32,67))),(34,(37,(39,40)))),(33,(41,((28,38),(29,30))))))),(45,(((52,(46,((56,(((44,66),(53,54)),(55,(62,((48,49),(50,51)))))),(58,(60,(47,59)))))),(57,61)),(64,(63,65)))))))))),(18,19))))))))))),(15,23)))))),(8,12)));

TREE tnt_3 = (1,((10,(6,(16,(20,((21,(4,(9,(11,(17,(2,(7,(3,(13,((5,(22,(68,(69,(14,((31,(((24,43),(35,(25,36))),((((42,(26,27)),(70,(32,67))),(34,(37,(39,40)))),(33,(41,((28,38),(29,30))))))),(45,(((52,((46,(56,(((44,66),(53,54)),(55,(62,((48,49),(50,51))))))),(58,(60,(47,59))))),(57,61)),(64,(63,65)))))))))),(18,19))))))))))),(15,23)))))),(8,12)));

TREE tnt_4 = (1,((10,(6,(16,(20,((21,(4,(9,(11,(17,(2,(7,(3,((13,(5,(22,(68,(69,(14,((31,(((24,43),(35,(25,36))),((((42,(26,27)),(70,(32,67))),(34,(37,(39,40)))),(33,(41,((28,38),(29,30))))))),(45,(((52,(46,((56,(((44,66),(53,54)),(55,(62,((48,49),(50,51)))))),(58,(60,(47,59)))))),(57,61)),(64,(63,65))))))))))),(18,19)))))))))),(15,23)))))),(8,12)));

TREE tnt_5 = (1,((10,(6,(16,(20,((21,(4,(9,(11,(17,(2,(7,(3,(13,((14,(5,(22,(68,(69,((31,(((24,43),(35,(25,36))),((((42,(26,27)),(70,(32,67))),(34,(37,(39,40)))),(33,(41,((28,38),(29,30))))))),(45,(((52,(46,((56,(((44,66),(53,54)),(55,(62,((48,49),(50,51)))))),(58,(60,(47,59)))))),(57,61)),(64,(63,65)))))))))),(18,19))))))))))),(15,23)))))),(8,12)));

TREE tnt_6 = (1,((10,(6,(16,(20,((21,(4,(9,(11,(17,(2,(7,(3,(13,((5,(14,(22,(68,(69,((31,(((24,43),(35,(25,36))),((((42,(26,27)),(70,(32,67))),(34,(37,(39,40)))),(33,(41,((28,38),(29,30))))))),(45,(((52,(46,((56,(((44,66),(53,54)),(55,(62,((48,49),(50,51)))))),(58,(60,(47,59)))))),(57,61)),(64,(63,65)))))))))),(18,19))))))))))),(15,23)))))),(8,12)));

TREE tnt_7 = (1,((10,(6,(16,(20,((21,(4,(9,(11,(17,(2,(7,(3,(13,((5,((14,22),(68,(69,((31,(((24,43),(35,(25,36))),((((42,(26,27)),(70,(32,67))),(34,(37,(39,40)))),(33,(41,((28,38),(29,30))))))),(45,(((52,(46,((56,(((44,66),(53,54)),(55,(62,((48,49),(50,51)))))),(58,(60,(47,59)))))),(57,61)),(64,(63,65))))))))),(18,19))))))))))),(15,23)))))),(8,12)));

TREE tnt_8 = (1,((10,(6,(16,(20,((21,(4,(9,(11,(17,(2,(7,(3,(13,((5,(22,(14,(68,(69,((31,(((24,43),(35,(25,36))),((((42,(26,27)),(70,(32,67))),(34,(37,(39,40)))),(33,(41,((28,38),(29,30))))))),(45,(((52,(46,((56,(((44,66),(53,54)),(55,(62,((48,49),(50,51)))))),(58,(60,(47,59)))))),(57,61)),(64,(63,65)))))))))),(18,19))))))))))),(15,23)))))),(8,12)));

TREE tnt_9 = (1,((10,(6,(16,(20,((21,(4,(9,(11,(17,(2,(7,(3,(13,((5,(22,((14,68),(69,((31,(((24,43),(35,(25,36))),((((42,(26,27)),(70,(32,67))),(34,(37,(39,40)))),(33,(41,((28,38),(29,30))))))),(45,(((52,(46,((56,(((44,66),(53,54)),(55,(62,((48,49),(50,51)))))),(58,(60,(47,59)))))),(57,61)),(64,(63,65))))))))),(18,19))))))))))),(15,23)))))),(8,12)));

TREE tnt_10 = (1,((10,(6,(16,(20,((21,(4,(9,(11,(17,(2,(7,(3,(13,((5,(22,(68,(14,(69,((31,(((24,43),(35,(25,36))),((((42,(26,27)),(70,(32,67))),(34,(37,(39,40)))),(33,(41,((28,38),(29,30))))))),(45,(((52,(46,((56,(((44,66),(53,54)),(55,(62,((48,49),(50,51)))))),(58,(60,(47,59)))))),(57,61)),(64,(63,65)))))))))),(18,19))))))))))),(15,23)))))),(8,12)));

TREE tnt_11 = (1,((10,(6,(16,(20,((21,(4,(9,(11,(17,(2,(7,(13,(3,((5,(22,(68,(14,(69,((31,(((24,43),(35,(25,36))),((((42,(26,27)),(70,(32,67))),(34,(37,(39,40)))),(33,(41,((28,38),(29,30))))))),(45,(((52,(46,((56,(((44,66),(53,54)),(55,(62,((48,49),(50,51)))))),(60,(58,(47,59)))))),(57,61)),(64,(63,65)))))))))),(18,19))))))))))),(15,23)))))),(8,12)));

TREE tnt_12 = (1,((10,(6,(16,(20,((21,(4,(9,(11,(17,(2,(7,(13,(3,((5,(22,(68,(14,(69,((31,(((24,43),(35,(25,36))),((((42,(26,27)),(70,(32,67))),(34,(37,(39,40)))),(33,(41,((28,38),(29,30))))))),(45,(((52,((46,(56,(((44,66),(53,54)),(55,(62,((48,49),(50,51))))))),(58,(60,(47,59))))),(57,61)),(64,(63,65)))))))))),(18,19))))))))))),(15,23)))))),(8,12)));

TREE tnt_13 = (1,((10,(6,(16,(20,((21,(4,(9,(11,(17,(2,(7,(13,(3,((5,(22,((14,68),(69,((31,(((24,43),(35,(25,36))),((((42,(26,27)),(70,(32,67))),(34,(37,(39,40)))),(33,(41,((28,38),(29,30))))))),(45,(((52,(46,((56,(((44,66),(53,54)),(55,(62,((48,49),(50,51)))))),(60,(58,(47,59)))))),(57,61)),(64,(63,65))))))))),(18,19))))))))))),(15,23)))))),(8,12)));

TREE tnt_14 = (1,((10,(6,(16,(20,((21,(4,(9,(11,(17,(2,(7,(3,((13,(5,(22,(68,(14,(69,((31,(((24,43),(35,(25,36))),((((42,(26,27)),(70,(32,67))),(34,(37,(39,40)))),(33,(41,((28,38),(29,30))))))),(45,(((52,(46,((56,(((44,66),(53,54)),(55,(62,((48,49),(50,51)))))),(60,(58,(47,59)))))),(57,61)),(64,(63,65))))))))))),(18,19)))))))))),(15,23)))))),(8,12)));

TREE tnt_15 = (1,((10,(6,(16,(20,((21,(4,(9,(11,(17,(2,(7,(13,(3,((14,(5,(22,(68,(69,((31,(((24,43),(35,(25,36))),((((42,(26,27)),(70,(32,67))),(34,(37,(39,40)))),(33,(41,((28,38),(29,30))))))),(45,(((52,(46,((56,(((44,66),(53,54)),(55,(62,((48,49),(50,51)))))),(60,(58,(47,59)))))),(57,61)),(64,(63,65)))))))))),(18,19))))))))))),(15,23)))))),(8,12)));

TREE tnt_16 = (1,((10,(6,(16,(20,((21,(4,(9,(11,(17,(2,(7,(13,(3,((5,(14,(22,(68,(69,((31,(((24,43),(35,(25,36))),((((42,(26,27)),(70,(32,67))),(34,(37,(39,40)))),(33,(41,((28,38),(29,30))))))),(45,(((52,(46,((56,(((44,66),(53,54)),(55,(62,((48,49),(50,51)))))),(60,(58,(47,59)))))),(57,61)),(64,(63,65)))))))))),(18,19))))))))))),(15,23)))))),(8,12)));

TREE tnt_17 = (1,((10,(6,(16,(20,((21,(4,(9,(11,(17,(2,(7,(13,(3,((5,((14,22),(68,(69,((31,(((24,43),(35,(25,36))),((((42,(26,27)),(70,(32,67))),(34,(37,(39,40)))),(33,(41,((28,38),(29,30))))))),(45,(((52,(46,((56,(((44,66),(53,54)),(55,(62,((48,49),(50,51)))))),(60,(58,(47,59)))))),(57,61)),(64,(63,65))))))))),(18,19))))))))))),(15,23)))))),(8,12)));

TREE tnt_18 = (1,((10,(6,(16,(20,((21,(4,(9,(11,(17,(2,(7,(13,(3,((5,(22,(14,(68,(69,((31,(((24,43),(35,(25,36))),((((42,(26,27)),(70,(32,67))),(34,(37,(39,40)))),(33,(41,((28,38),(29,30))))))),(45,(((52,(46,((56,(((44,66),(53,54)),(55,(62,((48,49),(50,51)))))),(60,(58,(47,59)))))),(57,61)),(64,(63,65)))))))))),(18,19))))))))))),(15,23)))))),(8,12)));

TREE tnt_19 = (1,((10,(6,(16,(20,((4,(9,(21,(11,(17,(2,(7,(13,(3,((5,(22,(68,(14,(69,((31,(((24,43),(35,(25,36))),((((42,(26,27)),(70,(32,67))),(34,(37,(39,40)))),(33,(41,((28,38),(29,30))))))),(45,(((52,(46,((56,(((44,66),(53,54)),(55,(62,((48,49),(50,51)))))),(60,(58,(47,59)))))),(57,61)),(64,(63,65)))))))))),(18,19))))))))))),(15,23)))))),(8,12)));

TREE tnt_20 = (1,((10,(6,(16,(20,((21,(4,(9,(11,(17,(2,(7,(13,(3,((5,(22,(68,(14,(69,((31,((43,(24,(35,(25,36)))),((((42,(26,27)),(70,(32,67))),(34,(37,(39,40)))),(33,(41,((28,38),(29,30))))))),(45,(((52,(46,((56,(((44,66),(53,54)),(55,(62,((48,49),(50,51)))))),(60,(58,(47,59)))))),(57,61)),(64,(63,65)))))))))),(18,19))))))))))),(15,23)))))),(8,12)));

TREE tnt_21 = (1,((10,(6,(16,(20,((4,(9,(21,(11,(17,(2,(7,(13,(3,((14,(5,(22,(68,(69,((31,(((24,43),(35,(25,36))),((((42,(26,27)),(70,(32,67))),(34,(37,(39,40)))),(33,(41,((28,38),(29,30))))))),(45,(((52,(46,((56,(((44,66),(53,54)),(55,(62,((48,49),(50,51)))))),(58,(60,(47,59)))))),(57,61)),(64,(63,65)))))))))),(18,19))))))))))),(15,23)))))),(8,12)));

TREE tnt_22 = (1,((10,(6,(16,(20,((4,(9,(21,(11,(17,(2,(7,(13,(3,((14,(5,(22,(68,(69,((31,((43,(24,(35,(25,36)))),((((42,(26,27)),(70,(32,67))),(34,(37,(39,40)))),(33,(41,((28,38),(29,30))))))),(45,(((52,(46,((56,(((44,66),(53,54)),(55,(62,((48,49),(50,51)))))),(58,(60,(47,59)))))),(57,61)),(64,(63,65)))))))))),(18,19))))))))))),(15,23)))))),(8,12)));

TREE tnt_23 = (1,((10,(6,(16,(20,((4,(9,(21,(11,(17,(2,(7,(13,(3,((14,(5,(22,(68,(69,((31,(((24,43),(35,(25,36))),((((42,(26,27)),(70,(32,67))),(34,(37,(39,40)))),(33,(41,((28,38),(29,30))))))),(45,(((52,((46,(56,(((44,66),(53,54)),(55,(62,((48,49),(50,51))))))),(58,(60,(47,59))))),(57,61)),(64,(63,65)))))))))),(18,19))))))))))),(15,23)))))),(8,12)));

TREE tnt_24 = (1,((10,(6,(16,(20,((4,(9,(21,(11,(17,(2,(7,(13,(3,((5,(14,(22,(68,(69,((31,(((24,43),(35,(25,36))),((((42,(26,27)),(70,(32,67))),(34,(37,(39,40)))),(33,(41,((28,38),(29,30))))))),(45,(((52,(46,((56,(((44,66),(53,54)),(55,(62,((48,49),(50,51)))))),(58,(60,(47,59)))))),(57,61)),(64,(63,65)))))))))),(18,19))))))))))),(15,23)))))),(8,12)));

TREE tnt_25 = (1,((10,(6,(16,(20,((4,(9,(21,(11,(17,(2,(7,(3,((13,(14,(5,(22,(68,(69,((31,(((24,43),(35,(25,36))),((((42,(26,27)),(70,(32,67))),(34,(37,(39,40)))),(33,(41,((28,38),(29,30))))))),(45,(((52,(46,((56,(((44,66),(53,54)),(55,(62,((48,49),(50,51)))))),(58,(60,(47,59)))))),(57,61)),(64,(63,65))))))))))),(18,19)))))))))),(15,23)))))),(8,12)));

TREE tnt_26 = (1,((10,(6,(16,(20,((4,(9,(21,(11,(17,(2,(7,(3,(((5,(22,(68,(69,((31,(((24,43),(35,(25,36))),((((42,(26,27)),(70,(32,67))),(34,(37,(39,40)))),(33,(41,((28,38),(29,30))))))),(45,(((52,(46,((56,(((44,66),(53,54)),(55,(62,((48,49),(50,51)))))),(58,(60,(47,59)))))),(57,61)),(64,(63,65))))))))),(13,14)),(18,19)))))))))),(15,23)))))),(8,12)));

TREE tnt_27 = (1,((10,(6,(16,(20,((4,(9,(21,(11,(17,(2,(7,(13,(3,((5,((14,22),(68,(69,((31,(((24,43),(35,(25,36))),((((42,(26,27)),(70,(32,67))),(34,(37,(39,40)))),(33,(41,((28,38),(29,30))))))),(45,(((52,(46,((56,(((44,66),(53,54)),(55,(62,((48,49),(50,51)))))),(58,(60,(47,59)))))),(57,61)),(64,(63,65))))))))),(18,19))))))))))),(15,23)))))),(8,12)));

TREE tnt_28 = (1,((10,(6,(16,(20,((4,(9,(21,(11,(17,(2,(7,(13,(3,((5,(22,(14,(68,(69,((31,(((24,43),(35,(25,36))),((((42,(26,27)),(70,(32,67))),(34,(37,(39,40)))),(33,(41,((28,38),(29,30))))))),(45,(((52,(46,((56,(((44,66),(53,54)),(55,(62,((48,49),(50,51)))))),(58,(60,(47,59)))))),(57,61)),(64,(63,65)))))))))),(18,19))))))))))),(15,23)))))),(8,12)));

TREE tnt_29 = (1,((10,(6,(16,(20,((4,(9,(21,(11,(17,(2,(7,(13,(3,((5,(22,((14,68),(69,((31,(((24,43),(35,(25,36))),((((42,(26,27)),(70,(32,67))),(34,(37,(39,40)))),(33,(41,((28,38),(29,30))))))),(45,(((52,(46,((56,(((44,66),(53,54)),(55,(62,((48,49),(50,51)))))),(58,(60,(47,59)))))),(57,61)),(64,(63,65))))))))),(18,19))))))))))),(15,23)))))),(8,12)));

TREE tnt_30 = (1,((10,(6,(16,(20,((4,(9,(21,(11,(17,(2,(7,(13,(3,((5,(22,(68,(14,(69,((31,(((24,43),(35,(25,36))),((((42,(26,27)),(70,(32,67))),(34,(37,(39,40)))),(33,(41,((28,38),(29,30))))))),(45,(((52,(46,((56,(((44,66),(53,54)),(55,(62,((48,49),(50,51)))))),(58,(60,(47,59)))))),(57,61)),(64,(63,65)))))))))),(18,19))))))))))),(15,23)))))),(8,12)));

TREE tnt_31 = (1,((10,(6,(16,(20,((21,(4,(9,(11,(17,(2,(7,(3,((14,(13,(5,(22,(68,(69,((31,(((24,43),(35,(25,36))),((((42,(26,27)),(70,(32,67))),(34,(37,(39,40)))),(33,(41,((28,38),(29,30))))))),(45,(((52,((46,(56,(((44,66),(53,54)),(55,(62,((48,49),(50,51))))))),(58,(60,(47,59))))),(57,61)),(64,(63,65))))))))))),(18,19)))))))))),(15,23)))))),(8,12)));

TREE tnt_32 = (1,((10,(6,(16,(20,((21,(4,(9,(11,(17,(2,(7,(3,((14,(13,(5,(22,(68,(69,((31,((43,(24,(35,(25,36)))),((((42,(26,27)),(70,(32,67))),(34,(37,(39,40)))),(33,(41,((28,38),(29,30))))))),(45,(((52,((46,(56,(((44,66),(53,54)),(55,(62,((48,49),(50,51))))))),(58,(60,(47,59))))),(57,61)),(64,(63,65))))))))))),(18,19)))))))))),(15,23)))))),(8,12)));

TREE tnt_33 = (1,((10,(6,(16,(20,((21,(4,(9,(11,(17,(2,(7,(3,((14,(13,(5,(22,(68,(69,((31,(((24,43),(35,(25,36))),((((42,(26,27)),(70,(32,67))),(34,(37,(39,40)))),(33,(41,((28,38),(29,30))))))),(45,(((52,(46,((56,(((44,66),(53,54)),(55,(62,((48,49),(50,51)))))),(58,(60,(47,59)))))),(57,61)),(64,(63,65))))))))))),(18,19)))))))))),(15,23)))))),(8,12)));

TREE tnt_34 = (1,((10,(6,(16,(20,((21,(4,(9,(11,(17,(2,(7,(3,((14,(13,(5,(22,(68,(69,((31,(((24,43),(35,(25,36))),((((42,(26,27)),(70,(32,67))),(34,(37,(39,40)))),(33,(41,((28,38),(29,30))))))),(45,(((52,(46,((56,(((44,66),(53,54)),(55,(62,((48,49),(50,51)))))),(60,(58,(47,59)))))),(57,61)),(64,(63,65))))))))))),(18,19)))))))))),(15,23)))))),(8,12)));

TREE tnt_35 = (1,((10,(6,(16,(20,((21,(4,(9,(11,(17,(2,(7,(13,(3,((14,(5,(22,(68,(69,((31,(((24,43),(35,(25,36))),((((42,(26,27)),(70,(32,67))),(34,(37,(39,40)))),(33,(41,((28,38),(29,30))))))),(45,(((52,((46,(56,(((44,66),(53,54)),(55,(62,((48,49),(50,51))))))),(58,(60,(47,59))))),(57,61)),(64,(63,65)))))))))),(18,19))))))))))),(15,23)))))),(8,12)));

TREE tnt_36 = (1,((10,(6,(16,(20,((21,(4,(9,(11,(17,(2,(7,(3,((13,(14,(5,(22,(68,(69,((31,(((24,43),(35,(25,36))),((((42,(26,27)),(70,(32,67))),(34,(37,(39,40)))),(33,(41,((28,38),(29,30))))))),(45,(((52,((46,(56,(((44,66),(53,54)),(55,(62,((48,49),(50,51))))))),(58,(60,(47,59))))),(57,61)),(64,(63,65))))))))))),(18,19)))))))))),(15,23)))))),(8,12)));

TREE tnt_37 = (1,((10,(6,(16,(20,((21,(4,(9,(11,(17,(2,(7,(3,((13,(5,(14,(22,(68,(69,((31,(((24,43),(35,(25,36))),((((42,(26,27)),(70,(32,67))),(34,(37,(39,40)))),(33,(41,((28,38),(29,30))))))),(45,(((52,((46,(56,(((44,66),(53,54)),(55,(62,((48,49),(50,51))))))),(58,(60,(47,59))))),(57,61)),(64,(63,65))))))))))),(18,19)))))))))),(15,23)))))),(8,12)));

TREE tnt_38 = (1,((10,(6,(16,(20,((21,(4,(9,(11,(17,(2,(7,(3,((13,((5,14),(22,(68,(69,((31,(((24,43),(35,(25,36))),((((42,(26,27)),(70,(32,67))),(34,(37,(39,40)))),(33,(41,((28,38),(29,30))))))),(45,(((52,((46,(56,(((44,66),(53,54)),(55,(62,((48,49),(50,51))))))),(58,(60,(47,59))))),(57,61)),(64,(63,65)))))))))),(18,19)))))))))),(15,23)))))),(8,12)));

TREE tnt_39 = (1,((10,(6,(16,(20,((21,(4,(9,(11,(17,(2,(7,(3,((13,(5,((14,22),(68,(69,((31,(((24,43),(35,(25,36))),((((42,(26,27)),(70,(32,67))),(34,(37,(39,40)))),(33,(41,((28,38),(29,30))))))),(45,(((52,((46,(56,(((44,66),(53,54)),(55,(62,((48,49),(50,51))))))),(58,(60,(47,59))))),(57,61)),(64,(63,65)))))))))),(18,19)))))))))),(15,23)))))),(8,12)));

TREE tnt_40 = (1,((10,(6,(16,(20,((21,(4,(9,(11,(17,(2,(7,(3,((13,(5,(22,(14,(68,(69,((31,(((24,43),(35,(25,36))),((((42,(26,27)),(70,(32,67))),(34,(37,(39,40)))),(33,(41,((28,38),(29,30))))))),(45,(((52,((46,(56,(((44,66),(53,54)),(55,(62,((48,49),(50,51))))))),(58,(60,(47,59))))),(57,61)),(64,(63,65))))))))))),(18,19)))))))))),(15,23)))))),(8,12)));

TREE tnt_41 = (1,((10,(6,(16,(20,((21,(4,(9,(11,(17,(2,(7,(3,(13,((14,(5,(22,(68,(69,((31,((43,(24,(35,(25,36)))),((((42,(26,27)),(70,(32,67))),(34,(37,(39,40)))),(33,(41,((28,38),(29,30))))))),(45,(((52,(46,((56,(((44,66),(53,54)),(55,(62,((48,49),(50,51)))))),(58,(60,(47,59)))))),(57,61)),(64,(63,65)))))))))),(18,19))))))))))),(15,23)))))),(8,12)));

TREE tnt_42 = (1,((10,(6,(16,(20,((21,(4,(9,(11,(17,(2,(7,(3,(13,((5,(14,(22,(68,(69,((31,((43,(24,(35,(25,36)))),((((42,(26,27)),(70,(32,67))),(34,(37,(39,40)))),(33,(41,((28,38),(29,30))))))),(45,(((52,(46,((56,(((44,66),(53,54)),(55,(62,((48,49),(50,51)))))),(58,(60,(47,59)))))),(57,61)),(64,(63,65)))))))))),(18,19))))))))))),(15,23)))))),(8,12)));

TREE tnt_43 = (1,((10,(6,(16,(20,((21,(4,(9,(11,(17,(2,(7,(3,((13,(14,(5,(22,(68,(69,((31,((43,(24,(35,(25,36)))),((((42,(26,27)),(70,(32,67))),(34,(37,(39,40)))),(33,(41,((28,38),(29,30))))))),(45,(((52,(46,((56,(((44,66),(53,54)),(55,(62,((48,49),(50,51)))))),(58,(60,(47,59)))))),(57,61)),(64,(63,65))))))))))),(18,19)))))))))),(15,23)))))),(8,12)));

TREE tnt_44 = (1,((10,(6,(16,(20,((21,(4,(9,(11,(17,(2,(7,(3,(((5,(22,(68,(69,((31,((43,(24,(35,(25,36)))),((((42,(26,27)),(70,(32,67))),(34,(37,(39,40)))),(33,(41,((28,38),(29,30))))))),(45,(((52,(46,((56,(((44,66),(53,54)),(55,(62,((48,49),(50,51)))))),(58,(60,(47,59)))))),(57,61)),(64,(63,65))))))))),(13,14)),(18,19)))))))))),(15,23)))))),(8,12)));

TREE tnt_45 = (1,((10,(6,(16,(20,((21,(4,(9,(11,(17,(2,(7,(3,(13,((5,(22,(14,(68,(69,((31,((43,(24,(35,(25,36)))),((((42,(26,27)),(70,(32,67))),(34,(37,(39,40)))),(33,(41,((28,38),(29,30))))))),(45,(((52,(46,((56,(((44,66),(53,54)),(55,(62,((48,49),(50,51)))))),(58,(60,(47,59)))))),(57,61)),(64,(63,65)))))))))),(18,19))))))))))),(15,23)))))),(8,12)));

TREE tnt_46 = (1,((10,(6,(16,(20,((21,(4,(9,(11,(17,(2,(7,(3,(13,((5,((14,22),(68,(69,((31,((43,(24,(35,(25,36)))),((((42,(26,27)),(70,(32,67))),(34,(37,(39,40)))),(33,(41,((28,38),(29,30))))))),(45,(((52,(46,((56,(((44,66),(53,54)),(55,(62,((48,49),(50,51)))))),(58,(60,(47,59)))))),(57,61)),(64,(63,65))))))))),(18,19))))))))))),(15,23)))))),(8,12)));

TREE tnt_47 = (1,((10,(6,(16,(20,((21,(4,(9,(11,(17,(2,(7,(3,(13,((5,(22,(68,(14,(69,((31,((43,(24,(35,(25,36)))),((((42,(26,27)),(70,(32,67))),(34,(37,(39,40)))),(33,(41,((28,38),(29,30))))))),(45,(((52,(46,((56,(((44,66),(53,54)),(55,(62,((48,49),(50,51)))))),(58,(60,(47,59)))))),(57,61)),(64,(63,65)))))))))),(18,19))))))))))),(15,23)))))),(8,12)));

TREE tnt_48 = (1,((10,(6,(16,(20,((21,(4,(9,(11,(17,(2,(7,(3,(13,((5,(22,((14,68),(69,((31,((43,(24,(35,(25,36)))),((((42,(26,27)),(70,(32,67))),(34,(37,(39,40)))),(33,(41,((28,38),(29,30))))))),(45,(((52,(46,((56,(((44,66),(53,54)),(55,(62,((48,49),(50,51)))))),(58,(60,(47,59)))))),(57,61)),(64,(63,65))))))))),(18,19))))))))))),(15,23)))))),(8,12)));

TREE tnt_49 = (1,((10,(6,(16,(20,((21,(4,(9,(11,(17,(2,(7,(3,(13,((14,(5,(22,(68,(69,((31,((43,(24,(35,(25,36)))),((((42,(26,27)),(70,(32,67))),(34,(37,(39,40)))),(33,(41,((28,38),(29,30))))))),(45,(((52,((46,(56,(((44,66),(53,54)),(55,(62,((48,49),(50,51))))))),(58,(60,(47,59))))),(57,61)),(64,(63,65)))))))))),(18,19))))))))))),(15,23)))))),(8,12)));

TREE tnt_50 = (1,((10,(6,(16,(20,((21,(4,(9,(11,(17,(2,(7,(3,(13,((14,(5,(22,(68,(69,((31,((43,(24,(35,(25,36)))),((((42,(26,27)),(70,(32,67))),(34,(37,(39,40)))),(33,(41,((28,38),(29,30))))))),(45,(((52,(46,((56,(((44,66),(53,54)),(55,(62,((48,49),(50,51)))))),(60,(58,(47,59)))))),(57,61)),(64,(63,65)))))))))),(18,19))))))))))),(15,23)))))),(8,12)));

TREE tnt_51 = (1,((10,(6,(16,(20,((21,(4,(9,(11,(17,(2,(7,(13,(3,((5,(14,(22,(68,(69,((31,(((24,43),(35,(25,36))),((((42,(26,27)),(70,(32,67))),(34,(37,(39,40)))),(33,(41,((28,38),(29,30))))))),(45,(((52,((46,(56,(((44,66),(53,54)),(55,(62,((48,49),(50,51))))))),(58,(60,(47,59))))),(57,61)),(63,(64,65)))))))))),(18,19))))))))))),(15,23)))))),(8,12)));

TREE tnt_52 = (1,((10,(6,(16,(20,((21,(4,(9,(11,(17,(2,(7,(13,(3,((5,(22,(14,(68,(69,((31,(((24,43),(35,(25,36))),((((42,(26,27)),(70,(32,67))),(34,(37,(39,40)))),(33,(41,((28,38),(29,30))))))),(45,(((52,((46,(56,(((44,66),(53,54)),(55,(62,((48,49),(50,51))))))),(58,(60,(47,59))))),(57,61)),(63,(64,65)))))))))),(18,19))))))))))),(15,23)))))),(8,12)));

TREE tnt_53 = (1,((10,(6,(16,(20,((21,(4,(9,(11,(17,(2,(7,(13,(3,((5,((14,22),(68,(69,((31,(((24,43),(35,(25,36))),((((42,(26,27)),(70,(32,67))),(34,(37,(39,40)))),(33,(41,((28,38),(29,30))))))),(45,(((52,((46,(56,(((44,66),(53,54)),(55,(62,((48,49),(50,51))))))),(58,(60,(47,59))))),(57,61)),(63,(64,65))))))))),(18,19))))))))))),(15,23)))))),(8,12)));

TREE tnt_54 = (1,((10,(6,(16,(20,((21,(4,(9,(11,(17,(2,(7,(13,(3,((5,(22,((14,68),(69,((31,(((24,43),(35,(25,36))),((((42,(26,27)),(70,(32,67))),(34,(37,(39,40)))),(33,(41,((28,38),(29,30))))))),(45,(((52,((46,(56,(((44,66),(53,54)),(55,(62,((48,49),(50,51))))))),(58,(60,(47,59))))),(57,61)),(63,(64,65))))))))),(18,19))))))))))),(15,23)))))),(8,12)));

TREE tnt_55 = (1,((10,(6,(16,(20,((4,(9,(21,(11,(17,(2,(7,(13,(3,((5,(14,(22,(68,(69,((31,(((24,43),(35,(25,36))),((((42,(26,27)),(70,(32,67))),(34,(37,(39,40)))),(33,(41,((28,38),(29,30))))))),(45,(((52,((46,(56,(((44,66),(53,54)),(55,(62,((48,49),(50,51))))))),(58,(60,(47,59))))),(57,61)),(63,(64,65)))))))))),(18,19))))))))))),(15,23)))))),(8,12)));

TREE tnt_56 = (1,((10,(6,(16,(20,((21,(4,(9,(11,(17,(2,(7,(13,(3,((5,(14,(22,(68,(69,((31,((43,(24,(35,(25,36)))),((((42,(26,27)),(70,(32,67))),(34,(37,(39,40)))),(33,(41,((28,38),(29,30))))))),(45,(((52,((46,(56,(((44,66),(53,54)),(55,(62,((48,49),(50,51))))))),(58,(60,(47,59))))),(57,61)),(63,(64,65)))))))))),(18,19))))))))))),(15,23)))))),(8,12)));

TREE tnt_57 = (1,((10,(6,(16,(20,((4,(9,(21,(11,(17,(2,(7,(13,(3,((5,(22,(14,(68,(69,((31,(((24,43),(35,(25,36))),((((42,(26,27)),(70,(32,67))),(34,(37,(39,40)))),(33,(41,((28,38),(29,30))))))),(45,(((52,((46,(56,(((44,66),(53,54)),(55,(62,((48,49),(50,51))))))),(58,(60,(47,59))))),(57,61)),(63,(64,65)))))))))),(18,19))))))))))),(15,23)))))),(8,12)));

TREE tnt_58 = (1,((10,(6,(16,(20,((21,(4,(9,(11,(17,(2,(7,(13,(3,((5,(22,(14,(68,(69,((31,((43,(24,(35,(25,36)))),((((42,(26,27)),(70,(32,67))),(34,(37,(39,40)))),(33,(41,((28,38),(29,30))))))),(45,(((52,((46,(56,(((44,66),(53,54)),(55,(62,((48,49),(50,51))))))),(58,(60,(47,59))))),(57,61)),(63,(64,65)))))))))),(18,19))))))))))),(15,23)))))),(8,12)));

TREE tnt_59 = (1,((10,(6,(16,(20,((4,(9,(21,(11,(17,(2,(7,(13,(3,((5,((14,22),(68,(69,((31,(((24,43),(35,(25,36))),((((42,(26,27)),(70,(32,67))),(34,(37,(39,40)))),(33,(41,((28,38),(29,30))))))),(45,(((52,((46,(56,(((44,66),(53,54)),(55,(62,((48,49),(50,51))))))),(58,(60,(47,59))))),(57,61)),(63,(64,65))))))))),(18,19))))))))))),(15,23)))))),(8,12)));

TREE tnt_60 = (1,((10,(6,(16,(20,((21,(4,(9,(11,(17,(2,(7,(13,(3,((5,((14,22),(68,(69,((31,((43,(24,(35,(25,36)))),((((42,(26,27)),(70,(32,67))),(34,(37,(39,40)))),(33,(41,((28,38),(29,30))))))),(45,(((52,((46,(56,(((44,66),(53,54)),(55,(62,((48,49),(50,51))))))),(58,(60,(47,59))))),(57,61)),(63,(64,65))))))))),(18,19))))))))))),(15,23)))))),(8,12)));

TREE tnt_61 = (1,((10,(6,(16,(20,((21,(4,(9,(11,(17,(2,(7,(13,(3,((5,(22,(68,(69,(14,((31,((43,(24,(35,(25,36)))),((((42,(26,27)),(70,(32,67))),(34,(37,(39,40)))),(33,(41,((28,38),(29,30))))))),(45,(((52,(46,((56,(((44,66),(53,54)),(55,(62,((48,49),(50,51)))))),(60,(58,(47,59)))))),(57,61)),(64,(63,65)))))))))),(18,19))))))))))),(15,23)))))),(8,12)));

TREE tnt_62 = (1,((10,(6,(16,(20,((21,(4,(9,(11,(17,(2,(7,(13,(3,((5,(14,(22,(68,(69,((31,((43,(24,(35,(25,36)))),((((42,(26,27)),(70,(32,67))),(34,(37,(39,40)))),(33,(41,((28,38),(29,30))))))),(45,(((52,(46,((56,(((44,66),(53,54)),(55,(62,((48,49),(50,51)))))),(60,(58,(47,59)))))),(57,61)),(64,(63,65)))))))))),(18,19))))))))))),(15,23)))))),(8,12)));

TREE tnt_63 = (1,((10,(6,(16,(20,((21,(4,(9,(11,(17,(2,(7,(13,(3,((5,(22,(14,(68,(69,((31,((43,(24,(35,(25,36)))),((((42,(26,27)),(70,(32,67))),(34,(37,(39,40)))),(33,(41,((28,38),(29,30))))))),(45,(((52,(46,((56,(((44,66),(53,54)),(55,(62,((48,49),(50,51)))))),(60,(58,(47,59)))))),(57,61)),(64,(63,65)))))))))),(18,19))))))))))),(15,23)))))),(8,12)));

TREE tnt_64 = (1,((10,(6,(16,(20,((21,(4,(9,(11,(17,(2,(7,(13,(3,((5,((14,22),(68,(69,((31,((43,(24,(35,(25,36)))),((((42,(26,27)),(70,(32,67))),(34,(37,(39,40)))),(33,(41,((28,38),(29,30))))))),(45,(((52,(46,((56,(((44,66),(53,54)),(55,(62,((48,49),(50,51)))))),(60,(58,(47,59)))))),(57,61)),(64,(63,65))))))))),(18,19))))))))))),(15,23)))))),(8,12)));

TREE tnt_65 = (1,((10,(6,(16,(20,((21,(4,(9,(11,(17,(2,(7,(13,(3,((5,(22,((14,68),(69,((31,((43,(24,(35,(25,36)))),((((42,(26,27)),(70,(32,67))),(34,(37,(39,40)))),(33,(41,((28,38),(29,30))))))),(45,(((52,(46,((56,(((44,66),(53,54)),(55,(62,((48,49),(50,51)))))),(60,(58,(47,59)))))),(57,61)),(64,(63,65))))))))),(18,19))))))))))),(15,23)))))),(8,12)));

TREE tnt_66 = (1,((10,(6,(16,(20,((4,(9,(21,(11,(17,(2,(7,(13,(3,((5,(22,(68,(69,(14,((31,((43,(24,(35,(25,36)))),((((42,(26,27)),(70,(32,67))),(34,(37,(39,40)))),(33,(41,((28,38),(29,30))))))),(45,(((52,(46,((56,(((44,66),(53,54)),(55,(62,((48,49),(50,51)))))),(60,(58,(47,59)))))),(57,61)),(64,(63,65)))))))))),(18,19))))))))))),(15,23)))))),(8,12)));

TREE tnt_67 = (1,((10,(6,(16,(20,((21,(4,(9,(11,(17,(2,(7,(13,(3,((5,(22,(68,(69,(14,((31,(((24,43),(35,(25,36))),((((42,(26,27)),(70,(32,67))),(34,(37,(39,40)))),(33,(41,((28,38),(29,30))))))),(45,(((52,(46,((56,(((44,66),(53,54)),(55,(62,((48,49),(50,51)))))),(60,(58,(47,59)))))),(57,61)),(64,(63,65)))))))))),(18,19))))))))))),(15,23)))))),(8,12)));

TREE tnt_68 = (1,((10,(6,(16,(20,((21,(4,(9,(11,(17,(2,(7,(13,(3,((5,(22,(68,(69,(14,((31,((43,(24,(35,(25,36)))),((((42,(26,27)),(70,(32,67))),(34,(37,(39,40)))),(33,(41,((28,38),(29,30))))))),(45,(((52,((46,(56,(((44,66),(53,54)),(55,(62,((48,49),(50,51))))))),(58,(60,(47,59))))),(57,61)),(64,(63,65)))))))))),(18,19))))))))))),(15,23)))))),(8,12)));

TREE tnt_69 = (1,((10,(6,(16,(20,((21,(4,(9,(11,(17,(2,(7,(3,((13,(5,(22,(68,(69,(14,((31,((43,(24,(35,(25,36)))),((((42,(26,27)),(70,(32,67))),(34,(37,(39,40)))),(33,(41,((28,38),(29,30))))))),(45,(((52,(46,((56,(((44,66),(53,54)),(55,(62,((48,49),(50,51)))))),(60,(58,(47,59)))))),(57,61)),(64,(63,65))))))))))),(18,19)))))))))),(15,23)))))),(8,12)));

TREE tnt_70 = (1,((10,(6,(16,(20,((21,(4,(9,(11,(17,(2,(7,(3,((13,(5,(14,(22,(68,(69,((31,((43,(24,(35,(25,36)))),((((42,(26,27)),(70,(32,67))),(34,(37,(39,40)))),(33,(41,((28,38),(29,30))))))),(45,(((52,(46,((56,(((44,66),(53,54)),(55,(62,((48,49),(50,51)))))),(60,(58,(47,59)))))),(57,61)),(64,(63,65))))))))))),(18,19)))))))))),(15,23)))))),(8,12)));

TREE tnt_71 = (1,((10,(6,(16,(20,((4,(9,(21,(11,(17,(2,(7,(13,(3,((5,(22,(68,(69,(14,((31,(((24,43),(35,(25,36))),((((42,(26,27)),(70,(32,67))),(34,(37,(39,40)))),(33,(41,((28,38),(29,30))))))),(45,(((52,(46,((56,(((44,66),(53,54)),(55,(62,((48,49),(50,51)))))),(60,(58,(47,59)))))),(57,61)),(64,(63,65)))))))))),(18,19))))))))))),(15,23)))))),(8,12)));

TREE tnt_72 = (1,((10,(6,(16,(20,((4,(9,(21,(11,(17,(2,(7,(3,((13,(5,(22,(68,(69,(14,((31,(((24,43),(35,(25,36))),((((42,(26,27)),(70,(32,67))),(34,(37,(39,40)))),(33,(41,((28,38),(29,30))))))),(45,(((52,(46,((56,(((44,66),(53,54)),(55,(62,((48,49),(50,51)))))),(60,(58,(47,59)))))),(57,61)),(64,(63,65))))))))))),(18,19)))))))))),(15,23)))))),(8,12)));

TREE tnt_73 = (1,((10,(6,(16,(20,((4,(9,(21,(11,(17,(2,(7,(13,(3,((14,(5,(22,(68,(69,((31,(((24,43),(35,(25,36))),((((42,(26,27)),(70,(32,67))),(34,(37,(39,40)))),(33,(41,((28,38),(29,30))))))),(45,(((52,(46,((56,(((44,66),(53,54)),(55,(62,((48,49),(50,51)))))),(60,(58,(47,59)))))),(57,61)),(64,(63,65)))))))))),(18,19))))))))))),(15,23)))))),(8,12)));

TREE tnt_74 = (1,((10,(6,(16,(20,((4,(9,(21,(11,(17,(2,(7,(13,(3,((5,(14,(22,(68,(69,((31,(((24,43),(35,(25,36))),((((42,(26,27)),(70,(32,67))),(34,(37,(39,40)))),(33,(41,((28,38),(29,30))))))),(45,(((52,(46,((56,(((44,66),(53,54)),(55,(62,((48,49),(50,51)))))),(60,(58,(47,59)))))),(57,61)),(64,(63,65)))))))))),(18,19))))))))))),(15,23)))))),(8,12)));

TREE tnt_75 = (1,((10,(6,(16,(20,((4,(9,(21,(11,(17,(2,(7,(13,(3,((5,(22,(14,(68,(69,((31,(((24,43),(35,(25,36))),((((42,(26,27)),(70,(32,67))),(34,(37,(39,40)))),(33,(41,((28,38),(29,30))))))),(45,(((52,(46,((56,(((44,66),(53,54)),(55,(62,((48,49),(50,51)))))),(60,(58,(47,59)))))),(57,61)),(64,(63,65)))))))))),(18,19))))))))))),(15,23)))))),(8,12)));

TREE tnt_76 = (1,((10,(6,(16,(20,((4,(9,(21,(11,(17,(2,(7,(13,(3,((5,((14,22),(68,(69,((31,(((24,43),(35,(25,36))),((((42,(26,27)),(70,(32,67))),(34,(37,(39,40)))),(33,(41,((28,38),(29,30))))))),(45,(((52,(46,((56,(((44,66),(53,54)),(55,(62,((48,49),(50,51)))))),(60,(58,(47,59)))))),(57,61)),(64,(63,65))))))))),(18,19))))))))))),(15,23)))))),(8,12)));

TREE tnt_77 = (1,((10,(6,(16,(20,((4,(9,(21,(11,(17,(2,(7,(13,(3,((5,(22,((14,68),(69,((31,(((24,43),(35,(25,36))),((((42,(26,27)),(70,(32,67))),(34,(37,(39,40)))),(33,(41,((28,38),(29,30))))))),(45,(((52,(46,((56,(((44,66),(53,54)),(55,(62,((48,49),(50,51)))))),(60,(58,(47,59)))))),(57,61)),(64,(63,65))))))))),(18,19))))))))))),(15,23)))))),(8,12)));

TREE tnt_78 = (1,((10,(6,(16,(20,((4,(9,(21,(11,(17,(2,(7,(13,(3,((5,(22,(68,(69,(14,((31,(((24,43),(35,(25,36))),((((42,(26,27)),(70,(32,67))),(34,(37,(39,40)))),(33,(41,((28,38),(29,30))))))),(45,(((52,(46,((56,(((44,66),(53,54)),(55,(62,((48,49),(50,51)))))),(58,(60,(47,59)))))),(57,61)),(64,(63,65)))))))))),(18,19))))))))))),(15,23)))))),(8,12)));

TREE tnt_79 = (1,((10,(6,(16,(20,((4,(9,(21,(11,(17,(2,(7,(13,(3,((5,(22,(68,(69,(14,((31,(((24,43),(35,(25,36))),((((42,(26,27)),(70,(32,67))),(34,(37,(39,40)))),(33,(41,((28,38),(29,30))))))),(45,(((52,((46,(56,(((44,66),(53,54)),(55,(62,((48,49),(50,51))))))),(58,(60,(47,59))))),(57,61)),(64,(63,65)))))))))),(18,19))))))))))),(15,23)))))),(8,12)));

TREE tnt_80 = (1,((10,(6,(16,(20,((4,(21,(9,(11,(17,(2,(7,(3,((13,(5,(22,(68,(69,(14,((31,(((24,43),(35,(25,36))),((((42,(26,27)),(70,(32,67))),(34,(37,(39,40)))),(33,(41,((28,38),(29,30))))))),(45,(((52,(46,((56,(((44,66),(53,54)),(55,(62,((48,49),(50,51)))))),(60,(58,(47,59)))))),(57,61)),(64,(63,65))))))))))),(18,19)))))))))),(15,23)))))),(8,12)));

TREE tnt_81 = (1,((10,(6,(16,(20,((21,(4,(9,(11,(17,(2,(7,(3,((13,(5,(22,(68,(69,(14,((31,((43,(24,(35,(25,36)))),((((42,(26,27)),(70,(32,67))),(34,(37,(39,40)))),(33,(41,((28,38),(29,30))))))),(45,(((52,((46,(56,(((44,66),(53,54)),(55,(62,((48,49),(50,51))))))),(58,(60,(47,59))))),(57,61)),(65,(63,64))))))))))),(18,19)))))))))),(15,23)))))),(8,12)));

TREE tnt_82 = (1,((10,(6,(16,(20,((21,(4,(9,(11,(17,(2,(7,(3,((13,(5,(22,(68,(69,(14,((31,(((24,43),(35,(25,36))),((((42,(26,27)),(70,(32,67))),(34,(37,(39,40)))),(33,(41,((28,38),(29,30))))))),(45,(((52,((46,(56,(((44,66),(53,54)),(55,(62,((48,49),(50,51))))))),(58,(60,(47,59))))),(57,61)),(65,(63,64))))))))))),(18,19)))))))))),(15,23)))))),(8,12)));

TREE tnt_83 = (1,((10,(6,(16,(20,((21,(4,(9,(11,(17,(2,(7,(3,((13,(5,(22,(68,(69,(14,((31,((43,(24,(35,(25,36)))),((((42,(26,27)),(70,(32,67))),(34,(37,(39,40)))),(33,(41,((28,38),(29,30))))))),(45,(((52,(46,((56,(((44,66),(53,54)),(55,(62,((48,49),(50,51)))))),(58,(60,(47,59)))))),(57,61)),(65,(63,64))))))))))),(18,19)))))))))),(15,23)))))),(8,12)));

TREE tnt_84 = (1,((10,(6,(16,(20,((21,(4,(9,(11,(17,(2,(7,(3,((13,(14,(5,(22,(68,(69,((31,((43,(24,(35,(25,36)))),((((42,(26,27)),(70,(32,67))),(34,(37,(39,40)))),(33,(41,((28,38),(29,30))))))),(45,(((52,((46,(56,(((44,66),(53,54)),(55,(62,((48,49),(50,51))))))),(58,(60,(47,59))))),(57,61)),(65,(63,64))))))))))),(18,19)))))))))),(15,23)))))),(8,12)));

TREE tnt_85 = (1,((10,(6,(16,(20,((21,(4,(9,(11,(17,(2,(7,(3,((13,(5,(14,(22,(68,(69,((31,((43,(24,(35,(25,36)))),((((42,(26,27)),(70,(32,67))),(34,(37,(39,40)))),(33,(41,((28,38),(29,30))))))),(45,(((52,((46,(56,(((44,66),(53,54)),(55,(62,((48,49),(50,51))))))),(58,(60,(47,59))))),(57,61)),(65,(63,64))))))))))),(18,19)))))))))),(15,23)))))),(8,12)));

TREE tnt_86 = (1,((10,(6,(16,(20,((21,(4,(9,(11,(17,(2,(7,(3,((13,((5,14),(22,(68,(69,((31,((43,(24,(35,(25,36)))),((((42,(26,27)),(70,(32,67))),(34,(37,(39,40)))),(33,(41,((28,38),(29,30))))))),(45,(((52,((46,(56,(((44,66),(53,54)),(55,(62,((48,49),(50,51))))))),(58,(60,(47,59))))),(57,61)),(65,(63,64)))))))))),(18,19)))))))))),(15,23)))))),(8,12)));

TREE tnt_87 = (1,((10,(6,(16,(20,((21,(4,(9,(11,(17,(2,(7,(3,((13,(5,((14,22),(68,(69,((31,((43,(24,(35,(25,36)))),((((42,(26,27)),(70,(32,67))),(34,(37,(39,40)))),(33,(41,((28,38),(29,30))))))),(45,(((52,((46,(56,(((44,66),(53,54)),(55,(62,((48,49),(50,51))))))),(58,(60,(47,59))))),(57,61)),(65,(63,64)))))))))),(18,19)))))))))),(15,23)))))),(8,12)));

TREE tnt_88 = (1,((10,(6,(16,(20,((21,(4,(9,(11,(17,(2,(7,(3,((13,(5,(22,(14,(68,(69,((31,((43,(24,(35,(25,36)))),((((42,(26,27)),(70,(32,67))),(34,(37,(39,40)))),(33,(41,((28,38),(29,30))))))),(45,(((52,((46,(56,(((44,66),(53,54)),(55,(62,((48,49),(50,51))))))),(58,(60,(47,59))))),(57,61)),(65,(63,64))))))))))),(18,19)))))))))),(15,23)))))),(8,12)));

TREE tnt_89 = (1,((10,(6,(16,(20,((21,(4,(9,(11,(17,(2,(7,(3,((13,(5,(22,((14,68),(69,((31,((43,(24,(35,(25,36)))),((((42,(26,27)),(70,(32,67))),(34,(37,(39,40)))),(33,(41,((28,38),(29,30))))))),(45,(((52,((46,(56,(((44,66),(53,54)),(55,(62,((48,49),(50,51))))))),(58,(60,(47,59))))),(57,61)),(65,(63,64)))))))))),(18,19)))))))))),(15,23)))))),(8,12)));

TREE tnt_90 = (1,((10,(6,(16,(20,((21,(4,(9,(11,(17,(2,(7,(3,((13,(5,(22,(68,(14,(69,((31,((43,(24,(35,(25,36)))),((((42,(26,27)),(70,(32,67))),(34,(37,(39,40)))),(33,(41,((28,38),(29,30))))))),(45,(((52,((46,(56,(((44,66),(53,54)),(55,(62,((48,49),(50,51))))))),(58,(60,(47,59))))),(57,61)),(65,(63,64))))))))))),(18,19)))))))))),(15,23)))))),(8,12)));

TREE tnt_91 = (1,((10,(6,(16,(20,((4,(9,(21,(11,(17,(2,(7,(3,((13,(5,(22,(68,((14,69),((31,(((24,43),(35,(25,36))),((((42,(26,27)),(70,(32,67))),(34,(37,(39,40)))),(33,(41,((28,38),(29,30))))))),(45,(((52,(46,((56,(((44,66),(53,54)),(55,(62,((48,49),(50,51)))))),(58,(60,(47,59)))))),(57,61)),(64,(63,65)))))))))),(18,19)))))))))),(15,23)))))),(8,12)));

TREE tnt_92 = (1,((10,(6,(16,(20,((4,(9,(21,(11,(17,(2,(7,(3,((13,(5,(22,(68,((14,69),((31,(((24,43),(35,(25,36))),((((42,(26,27)),(70,(32,67))),(34,(37,(39,40)))),(33,(41,((28,38),(29,30))))))),(45,(((52,((46,(56,(((44,66),(53,54)),(55,(62,((48,49),(50,51))))))),(58,(60,(47,59))))),(57,61)),(64,(63,65)))))))))),(18,19)))))))))),(15,23)))))),(8,12)));

TREE tnt_93 = (1,((10,(6,(16,(20,((4,(9,(21,(11,(17,(2,(7,(3,((13,(5,(14,(22,(68,(69,((31,(((24,43),(35,(25,36))),((((42,(26,27)),(70,(32,67))),(34,(37,(39,40)))),(33,(41,((28,38),(29,30))))))),(45,(((52,(46,((56,(((44,66),(53,54)),(55,(62,((48,49),(50,51)))))),(58,(60,(47,59)))))),(57,61)),(64,(63,65))))))))))),(18,19)))))))))),(15,23)))))),(8,12)));

TREE tnt_94 = (1,((10,(6,(16,(20,((4,(9,(21,(11,(17,(2,(7,(3,((13,((5,14),(22,(68,(69,((31,(((24,43),(35,(25,36))),((((42,(26,27)),(70,(32,67))),(34,(37,(39,40)))),(33,(41,((28,38),(29,30))))))),(45,(((52,(46,((56,(((44,66),(53,54)),(55,(62,((48,49),(50,51)))))),(58,(60,(47,59)))))),(57,61)),(64,(63,65)))))))))),(18,19)))))))))),(15,23)))))),(8,12)));

TREE tnt_95 = (1,((10,(6,(16,(20,((4,(9,(21,(11,(17,(2,(7,(3,((13,(5,(22,(14,(68,(69,((31,(((24,43),(35,(25,36))),((((42,(26,27)),(70,(32,67))),(34,(37,(39,40)))),(33,(41,((28,38),(29,30))))))),(45,(((52,(46,((56,(((44,66),(53,54)),(55,(62,((48,49),(50,51)))))),(58,(60,(47,59)))))),(57,61)),(64,(63,65))))))))))),(18,19)))))))))),(15,23)))))),(8,12)));

TREE tnt_96 = (1,((10,(6,(16,(20,((4,(9,(21,(11,(17,(2,(7,(3,((13,(5,((14,22),(68,(69,((31,(((24,43),(35,(25,36))),((((42,(26,27)),(70,(32,67))),(34,(37,(39,40)))),(33,(41,((28,38),(29,30))))))),(45,(((52,(46,((56,(((44,66),(53,54)),(55,(62,((48,49),(50,51)))))),(58,(60,(47,59)))))),(57,61)),(64,(63,65)))))))))),(18,19)))))))))),(15,23)))))),(8,12)));

TREE tnt_97 = (1,((10,(6,(16,(20,((4,(9,(21,(11,(17,(2,(7,(3,((13,(5,(22,((14,68),(69,((31,(((24,43),(35,(25,36))),((((42,(26,27)),(70,(32,67))),(34,(37,(39,40)))),(33,(41,((28,38),(29,30))))))),(45,(((52,(46,((56,(((44,66),(53,54)),(55,(62,((48,49),(50,51)))))),(58,(60,(47,59)))))),(57,61)),(64,(63,65)))))))))),(18,19)))))))))),(15,23)))))),(8,12)));

TREE tnt_98 = (1,((10,(6,(16,(20,((4,(9,(21,(11,(17,(2,(7,(3,((13,(5,(22,(68,(14,(69,((31,(((24,43),(35,(25,36))),((((42,(26,27)),(70,(32,67))),(34,(37,(39,40)))),(33,(41,((28,38),(29,30))))))),(45,(((52,(46,((56,(((44,66),(53,54)),(55,(62,((48,49),(50,51)))))),(58,(60,(47,59)))))),(57,61)),(64,(63,65))))))))))),(18,19)))))))))),(15,23)))))),(8,12)));

TREE tnt_99 = (1,((10,(6,(16,(20,((4,(9,(21,(11,(17,(2,(7,(3,((13,(5,(22,(68,((14,69),((31,((43,(24,(35,(25,36)))),((((42,(26,27)),(70,(32,67))),(34,(37,(39,40)))),(33,(41,((28,38),(29,30))))))),(45,(((52,(46,((56,(((44,66),(53,54)),(55,(62,((48,49),(50,51)))))),(58,(60,(47,59)))))),(57,61)),(64,(63,65)))))))))),(18,19)))))))))),(15,23)))))),(8,12)));

TREE tnt_100 = (1,((10,(6,(16,(20,((4,(9,(21,(11,(17,(2,(7,(3,((14,(13,(5,(22,(68,(69,((31,(((24,43),(35,(25,36))),((((42,(26,27)),(70,(32,67))),(34,(37,(39,40)))),(33,(41,((28,38),(29,30))))))),(45,(((52,((46,(56,(((44,66),(53,54)),(55,(62,((48,49),(50,51))))))),(58,(60,(47,59))))),(57,61)),(64,(63,65))))))))))),(18,19)))))))))),(15,23)))))),(8,12)));

TREE tnt_101 = (1,((10,(6,(16,(20,((4,(9,(21,(11,(17,(2,(7,(3,((13,(5,(22,(68,(14,(69,((31,(((24,43),(35,(25,36))),((((42,(26,27)),(70,(32,67))),(34,(37,(39,40)))),(33,(41,((28,38),(29,30))))))),(45,(((52,((46,(56,(((44,66),(53,54)),(55,(62,((48,49),(50,51))))))),(58,(60,(47,59))))),(57,61)),(64,(63,65))))))))))),(18,19)))))))))),(15,23)))))),(8,12)));

TREE tnt_102 = (1,((10,(6,(16,(20,((4,(9,(21,(11,(17,(2,(7,(3,((13,(5,(22,(68,(14,(69,((31,(((24,43),(35,(25,36))),((((42,(26,27)),(70,(32,67))),(34,(37,(39,40)))),(33,(41,((28,38),(29,30))))))),(45,(((52,(46,((56,(((44,66),(53,54)),(55,(62,((48,49),(50,51)))))),(60,(58,(47,59)))))),(57,61)),(64,(63,65))))))))))),(18,19)))))))))),(15,23)))))),(8,12)));

TREE tnt_103 = (1,((10,(6,(16,(20,((4,(9,(21,(11,(17,(2,(7,(3,((13,(5,(22,((14,68),(69,((31,(((24,43),(35,(25,36))),((((42,(26,27)),(70,(32,67))),(34,(37,(39,40)))),(33,(41,((28,38),(29,30))))))),(45,(((52,((46,(56,(((44,66),(53,54)),(55,(62,((48,49),(50,51))))))),(58,(60,(47,59))))),(57,61)),(64,(63,65)))))))))),(18,19)))))))))),(15,23)))))),(8,12)));

TREE tnt_104 = (1,((10,(6,(16,(20,((4,(21,(9,(11,(17,(2,(7,(3,((13,(5,(22,(68,(14,(69,((31,(((24,43),(35,(25,36))),((((42,(26,27)),(70,(32,67))),(34,(37,(39,40)))),(33,(41,((28,38),(29,30))))))),(45,(((52,((46,(56,(((44,66),(53,54)),(55,(62,((48,49),(50,51))))))),(58,(60,(47,59))))),(57,61)),(64,(63,65))))))))))),(18,19)))))))))),(15,23)))))),(8,12)));

TREE tnt_105 = (1,((10,(6,(16,(20,((4,(9,(21,(11,(17,(2,(7,(13,(3,((5,(22,(68,(14,(69,((31,(((24,43),(35,(25,36))),((((42,(26,27)),(70,(32,67))),(34,(37,(39,40)))),(33,(41,((28,38),(29,30))))))),(45,(((52,((46,(56,(((44,66),(53,54)),(55,(62,((48,49),(50,51))))))),(58,(60,(47,59))))),(57,61)),(64,(63,65)))))))))),(18,19))))))))))),(15,23)))))),(8,12)));

TREE tnt_106 = (1,((10,(6,(16,(20,((4,(9,(21,(11,(17,(2,(7,(3,((13,(14,(5,(22,(68,(69,((31,(((24,43),(35,(25,36))),((((42,(26,27)),(70,(32,67))),(34,(37,(39,40)))),(33,(41,((28,38),(29,30))))))),(45,(((52,((46,(56,(((44,66),(53,54)),(55,(62,((48,49),(50,51))))))),(58,(60,(47,59))))),(57,61)),(64,(63,65))))))))))),(18,19)))))))))),(15,23)))))),(8,12)));

TREE tnt_107 = (1,((10,(6,(16,(20,((4,(9,(21,(11,(17,(2,(7,(3,((13,(5,(14,(22,(68,(69,((31,(((24,43),(35,(25,36))),((((42,(26,27)),(70,(32,67))),(34,(37,(39,40)))),(33,(41,((28,38),(29,30))))))),(45,(((52,((46,(56,(((44,66),(53,54)),(55,(62,((48,49),(50,51))))))),(58,(60,(47,59))))),(57,61)),(64,(63,65))))))))))),(18,19)))))))))),(15,23)))))),(8,12)));

TREE tnt_108 = (1,((10,(6,(16,(20,((4,(9,(21,(11,(17,(2,(7,(3,((13,((5,14),(22,(68,(69,((31,(((24,43),(35,(25,36))),((((42,(26,27)),(70,(32,67))),(34,(37,(39,40)))),(33,(41,((28,38),(29,30))))))),(45,(((52,((46,(56,(((44,66),(53,54)),(55,(62,((48,49),(50,51))))))),(58,(60,(47,59))))),(57,61)),(64,(63,65)))))))))),(18,19)))))))))),(15,23)))))),(8,12)));

TREE tnt_109 = (1,((10,(6,(16,(20,((4,(9,(21,(11,(17,(2,(7,(3,((13,(5,((14,22),(68,(69,((31,(((24,43),(35,(25,36))),((((42,(26,27)),(70,(32,67))),(34,(37,(39,40)))),(33,(41,((28,38),(29,30))))))),(45,(((52,((46,(56,(((44,66),(53,54)),(55,(62,((48,49),(50,51))))))),(58,(60,(47,59))))),(57,61)),(64,(63,65)))))))))),(18,19)))))))))),(15,23)))))),(8,12)));

TREE tnt_110 = (1,((10,(6,(16,(20,((4,(9,(21,(11,(17,(2,(7,(3,((13,(5,(22,(14,(68,(69,((31,(((24,43),(35,(25,36))),((((42,(26,27)),(70,(32,67))),(34,(37,(39,40)))),(33,(41,((28,38),(29,30))))))),(45,(((52,((46,(56,(((44,66),(53,54)),(55,(62,((48,49),(50,51))))))),(58,(60,(47,59))))),(57,61)),(64,(63,65))))))))))),(18,19)))))))))),(15,23)))))),(8,12)));

TREE tnt_111 = (1,((10,(6,(16,(20,((4,(9,(21,(11,(17,(2,(7,(3,(13,((5,(22,(68,(69,(14,((31,((43,(24,(35,(25,36)))),((((42,(26,27)),(70,(32,67))),(34,(37,(39,40)))),(33,(41,((28,38),(29,30))))))),(45,(((52,(46,((56,(((44,66),(53,54)),(55,(62,((48,49),(50,51)))))),(58,(60,(47,59)))))),(57,61)),(64,(63,65)))))))))),(18,19))))))))))),(15,23)))))),(8,12)));

TREE tnt_112 = (1,((10,(6,(16,(20,((4,(9,(21,(11,(17,(2,(7,(3,(13,((5,(14,(22,(68,(69,((31,((43,(24,(35,(25,36)))),((((42,(26,27)),(70,(32,67))),(34,(37,(39,40)))),(33,(41,((28,38),(29,30))))))),(45,(((52,(46,((56,(((44,66),(53,54)),(55,(62,((48,49),(50,51)))))),(58,(60,(47,59)))))),(57,61)),(64,(63,65)))))))))),(18,19))))))))))),(15,23)))))),(8,12)));

TREE tnt_113 = (1,((10,(6,(16,(20,((4,(9,(21,(11,(17,(2,(7,(3,(13,((5,(22,(14,(68,(69,((31,((43,(24,(35,(25,36)))),((((42,(26,27)),(70,(32,67))),(34,(37,(39,40)))),(33,(41,((28,38),(29,30))))))),(45,(((52,(46,((56,(((44,66),(53,54)),(55,(62,((48,49),(50,51)))))),(58,(60,(47,59)))))),(57,61)),(64,(63,65)))))))))),(18,19))))))))))),(15,23)))))),(8,12)));

TREE tnt_114 = (1,((10,(6,(16,(20,((4,(9,(21,(11,(17,(2,(7,(3,(13,((5,((14,22),(68,(69,((31,((43,(24,(35,(25,36)))),((((42,(26,27)),(70,(32,67))),(34,(37,(39,40)))),(33,(41,((28,38),(29,30))))))),(45,(((52,(46,((56,(((44,66),(53,54)),(55,(62,((48,49),(50,51)))))),(58,(60,(47,59)))))),(57,61)),(64,(63,65))))))))),(18,19))))))))))),(15,23)))))),(8,12)));

TREE tnt_115 = (1,((10,(6,(16,(20,((4,(9,(21,(11,(17,(2,(7,(3,(13,((5,(22,(68,(14,(69,((31,((43,(24,(35,(25,36)))),((((42,(26,27)),(70,(32,67))),(34,(37,(39,40)))),(33,(41,((28,38),(29,30))))))),(45,(((52,(46,((56,(((44,66),(53,54)),(55,(62,((48,49),(50,51)))))),(58,(60,(47,59)))))),(57,61)),(64,(63,65)))))))))),(18,19))))))))))),(15,23)))))),(8,12)));

TREE tnt_116 = (1,((10,(6,(16,(20,((4,(9,(21,(11,(17,(2,(7,(3,(13,((5,(22,((14,68),(69,((31,((43,(24,(35,(25,36)))),((((42,(26,27)),(70,(32,67))),(34,(37,(39,40)))),(33,(41,((28,38),(29,30))))))),(45,(((52,(46,((56,(((44,66),(53,54)),(55,(62,((48,49),(50,51)))))),(58,(60,(47,59)))))),(57,61)),(64,(63,65))))))))),(18,19))))))))))),(15,23)))))),(8,12)));

TREE tnt_117 = (1,((10,(6,(16,(20,((4,(9,(21,(11,(17,(2,(7,(3,(13,((5,(22,(68,(69,(14,((31,((43,(24,(35,(25,36)))),((((42,(26,27)),(70,(32,67))),(34,(37,(39,40)))),(33,(41,((28,38),(29,30))))))),(45,(((52,((46,(56,(((44,66),(53,54)),(55,(62,((48,49),(50,51))))))),(58,(60,(47,59))))),(57,61)),(64,(63,65)))))))))),(18,19))))))))))),(15,23)))))),(8,12)));

TREE tnt_118 = (1,((10,(6,(16,(20,((4,(9,(21,(11,(17,(2,(7,(3,((13,(5,(14,(22,(68,(69,((31,((43,(24,(35,(25,36)))),((((42,(26,27)),(70,(32,67))),(34,(37,(39,40)))),(33,(41,((28,38),(29,30))))))),(45,(((52,(46,((56,(((44,66),(53,54)),(55,(62,((48,49),(50,51)))))),(58,(60,(47,59)))))),(57,61)),(64,(63,65))))))))))),(18,19)))))))))),(15,23)))))),(8,12)));

TREE tnt_119 = (1,((10,(6,(16,(20,((4,(9,(21,(11,(17,(2,(7,(3,(13,((5,(14,(22,(68,(69,((31,((43,(24,(35,(25,36)))),((((42,(26,27)),(70,(32,67))),(34,(37,(39,40)))),(33,(41,((28,38),(29,30))))))),(45,(((52,((46,(56,(((44,66),(53,54)),(55,(62,((48,49),(50,51))))))),(58,(60,(47,59))))),(57,61)),(64,(63,65)))))))))),(18,19))))))))))),(15,23)))))),(8,12)));

TREE tnt_120 = (1,((10,(6,(16,(20,((4,(9,(21,(11,(17,(2,(7,(3,(13,((5,(14,(22,(68,(69,((31,((43,(24,(35,(25,36)))),((((42,(26,27)),(70,(32,67))),(34,(37,(39,40)))),(33,(41,((28,38),(29,30))))))),(45,(((52,(46,((56,(((44,66),(53,54)),(55,(62,((48,49),(50,51)))))),(60,(58,(47,59)))))),(57,61)),(64,(63,65)))))))))),(18,19))))))))))),(15,23)))))),(8,12)));

TREE tnt_121 = (1,((10,(6,(16,(20,((21,(4,(9,(11,(17,(2,(7,(3,((13,(5,((14,22),(68,(69,((31,((43,(24,(35,(25,36)))),((((42,(26,27)),(70,(32,67))),(34,(37,(39,40)))),(33,(41,((28,38),(29,30))))))),(45,(((52,(46,((56,(((44,66),(53,54)),(55,(62,((48,49),(50,51)))))),(58,(60,(47,59)))))),(57,61)),(63,(64,65)))))))))),(18,19)))))))))),(15,23)))))),(8,12)));

TREE tnt_122 = (1,((10,(6,(16,(20,((21,(4,(9,(11,(17,(2,(7,(3,((13,(5,((14,22),(68,(69,((31,(((24,43),(35,(25,36))),((((42,(26,27)),(70,(32,67))),(34,(37,(39,40)))),(33,(41,((28,38),(29,30))))))),(45,(((52,(46,((56,(((44,66),(53,54)),(55,(62,((48,49),(50,51)))))),(58,(60,(47,59)))))),(57,61)),(63,(64,65)))))))))),(18,19)))))))))),(15,23)))))),(8,12)));

TREE tnt_123 = (1,((10,(6,(16,(20,((21,(4,(9,(11,(17,(2,(7,(3,((13,(5,((14,22),(68,(69,((31,((43,(24,(35,(25,36)))),((((42,(26,27)),(70,(32,67))),(34,(37,(39,40)))),(33,(41,((28,38),(29,30))))))),(45,(((52,(46,((56,(((44,66),(53,54)),(55,(62,((48,49),(50,51)))))),(60,(58,(47,59)))))),(57,61)),(63,(64,65)))))))))),(18,19)))))))))),(15,23)))))),(8,12)));

TREE tnt_124 = (1,((10,(6,(16,(20,((21,(4,(9,(11,(17,(2,(7,(3,((13,(5,(14,(22,(68,(69,((31,((43,(24,(35,(25,36)))),((((42,(26,27)),(70,(32,67))),(34,(37,(39,40)))),(33,(41,((28,38),(29,30))))))),(45,(((52,(46,((56,(((44,66),(53,54)),(55,(62,((48,49),(50,51)))))),(58,(60,(47,59)))))),(57,61)),(63,(64,65))))))))))),(18,19)))))))))),(15,23)))))),(8,12)));

TREE tnt_125 = (1,((10,(6,(16,(20,((21,(4,(9,(11,(17,(2,(7,(3,((13,((5,14),(22,(68,(69,((31,((43,(24,(35,(25,36)))),((((42,(26,27)),(70,(32,67))),(34,(37,(39,40)))),(33,(41,((28,38),(29,30))))))),(45,(((52,(46,((56,(((44,66),(53,54)),(55,(62,((48,49),(50,51)))))),(58,(60,(47,59)))))),(57,61)),(63,(64,65)))))))))),(18,19)))))))))),(15,23)))))),(8,12)));

TREE tnt_126 = (1,((10,(6,(16,(20,((21,(4,(9,(11,(17,(2,(7,(3,((13,(5,(22,((14,68),(69,((31,((43,(24,(35,(25,36)))),((((42,(26,27)),(70,(32,67))),(34,(37,(39,40)))),(33,(41,((28,38),(29,30))))))),(45,(((52,(46,((56,(((44,66),(53,54)),(55,(62,((48,49),(50,51)))))),(58,(60,(47,59)))))),(57,61)),(63,(64,65)))))))))),(18,19)))))))))),(15,23)))))),(8,12)));

TREE tnt_127 = (1,((10,(6,(16,(20,((21,(4,(9,(11,(17,(2,(7,(3,((13,(5,(22,(68,(14,(69,((31,((43,(24,(35,(25,36)))),((((42,(26,27)),(70,(32,67))),(34,(37,(39,40)))),(33,(41,((28,38),(29,30))))))),(45,(((52,(46,((56,(((44,66),(53,54)),(55,(62,((48,49),(50,51)))))),(58,(60,(47,59)))))),(57,61)),(63,(64,65))))))))))),(18,19)))))))))),(15,23)))))),(8,12)));

TREE tnt_128 = (1,((10,(6,(16,(20,((4,(9,(21,(11,(17,(2,(7,(3,((13,(5,((14,22),(68,(69,((31,((43,(24,(35,(25,36)))),((((42,(26,27)),(70,(32,67))),(34,(37,(39,40)))),(33,(41,((28,38),(29,30))))))),(45,(((52,(46,((56,(((44,66),(53,54)),(55,(62,((48,49),(50,51)))))),(58,(60,(47,59)))))),(57,61)),(63,(64,65)))))))))),(18,19)))))))))),(15,23)))))),(8,12)));

TREE tnt_129 = (1,((10,(6,(16,(20,((21,(4,(9,(11,(17,(2,(7,(3,((13,(5,(22,(14,(68,(69,((31,((43,(24,(35,(25,36)))),((((42,(26,27)),(70,(32,67))),(34,(37,(39,40)))),(33,(41,((28,38),(29,30))))))),(45,(((52,(46,((56,(((44,66),(53,54)),(55,(62,((48,49),(50,51)))))),(58,(60,(47,59)))))),(57,61)),(63,(64,65))))))))))),(18,19)))))))))),(15,23)))))),(8,12)));

TREE tnt_130 = (1,((10,(6,(16,(20,((21,(4,(9,(11,(17,(2,(7,(3,((13,(14,(5,(22,(68,(69,((31,(((24,43),(35,(25,36))),((((42,(26,27)),(70,(32,67))),(34,(37,(39,40)))),(33,(41,((28,38),(29,30))))))),(45,(((52,(46,((56,(((44,66),(53,54)),(55,(62,((48,49),(50,51)))))),(58,(60,(47,59)))))),(57,61)),(63,(64,65))))))))))),(18,19)))))))))),(15,23)))))),(8,12)));

TREE tnt_131 = (1,((10,(6,(16,(20,((21,(4,(9,(11,(17,(2,(7,(3,((13,(14,(5,(22,(68,(69,((31,(((24,43),(35,(25,36))),((((42,(26,27)),(70,(32,67))),(34,(37,(39,40)))),(33,(41,((28,38),(29,30))))))),(45,(((52,(46,((56,(((44,66),(53,54)),(55,(62,((48,49),(50,51)))))),(60,(58,(47,59)))))),(57,61)),(64,(63,65))))))))))),(18,19)))))))))),(15,23)))))),(8,12)));

TREE tnt_132 = (1,((10,(6,(16,(20,((21,(4,(9,(11,(17,(2,(7,(3,((13,(5,(22,(14,(68,(69,((31,(((24,43),(35,(25,36))),((((42,(26,27)),(70,(32,67))),(34,(37,(39,40)))),(33,(41,((28,38),(29,30))))))),(45,(((52,(46,((56,(((44,66),(53,54)),(55,(62,((48,49),(50,51)))))),(60,(58,(47,59)))))),(57,61)),(64,(63,65))))))))))),(18,19)))))))))),(15,23)))))),(8,12)));

TREE tnt_133 = (1,((10,(6,(16,(20,((21,(4,(9,(11,(17,(2,(7,(3,((13,(5,((14,22),(68,(69,((31,(((24,43),(35,(25,36))),((((42,(26,27)),(70,(32,67))),(34,(37,(39,40)))),(33,(41,((28,38),(29,30))))))),(45,(((52,(46,((56,(((44,66),(53,54)),(55,(62,((48,49),(50,51)))))),(60,(58,(47,59)))))),(57,61)),(64,(63,65)))))))))),(18,19)))))))))),(15,23)))))),(8,12)));

TREE tnt_134 = (1,((10,(6,(16,(20,((21,(4,(9,(11,(17,(2,(7,(3,((13,(5,(22,((14,68),(69,((31,(((24,43),(35,(25,36))),((((42,(26,27)),(70,(32,67))),(34,(37,(39,40)))),(33,(41,((28,38),(29,30))))))),(45,(((52,(46,((56,(((44,66),(53,54)),(55,(62,((48,49),(50,51)))))),(60,(58,(47,59)))))),(57,61)),(64,(63,65)))))))))),(18,19)))))))))),(15,23)))))),(8,12)));

TREE tnt_135 = (1,((10,(6,(16,(20,((4,(9,(21,(11,(17,(2,(7,(3,((13,(14,(5,(22,(68,(69,((31,(((24,43),(35,(25,36))),((((42,(26,27)),(70,(32,67))),(34,(37,(39,40)))),(33,(41,((28,38),(29,30))))))),(45,(((52,(46,((56,(((44,66),(53,54)),(55,(62,((48,49),(50,51)))))),(60,(58,(47,59)))))),(57,61)),(64,(63,65))))))))))),(18,19)))))))))),(15,23)))))),(8,12)));

TREE tnt_136 = (1,((10,(6,(16,(20,((21,(4,(9,(11,(17,(2,(7,(3,((13,(14,(5,(22,(68,(69,((31,((43,(24,(35,(25,36)))),((((42,(26,27)),(70,(32,67))),(34,(37,(39,40)))),(33,(41,((28,38),(29,30))))))),(45,(((52,(46,((56,(((44,66),(53,54)),(55,(62,((48,49),(50,51)))))),(60,(58,(47,59)))))),(57,61)),(64,(63,65))))))))))),(18,19)))))))))),(15,23)))))),(8,12)));

TREE tnt_137 = (1,((10,(6,(16,(20,((21,(4,(9,(11,(17,(2,(7,(3,((13,(5,(14,(22,(68,(69,((31,(((24,43),(35,(25,36))),((((42,(26,27)),(70,(32,67))),(34,(37,(39,40)))),(33,(41,((28,38),(29,30))))))),(45,(((52,(46,((56,(((44,66),(53,54)),(55,(62,((48,49),(50,51)))))),(60,(58,(47,59)))))),(57,61)),(64,(63,65))))))))))),(18,19)))))))))),(15,23)))))),(8,12)));

TREE tnt_138 = (1,((10,(6,(16,(20,((21,(4,(9,(11,(17,(2,(7,(3,((13,((5,14),(22,(68,(69,((31,(((24,43),(35,(25,36))),((((42,(26,27)),(70,(32,67))),(34,(37,(39,40)))),(33,(41,((28,38),(29,30))))))),(45,(((52,(46,((56,(((44,66),(53,54)),(55,(62,((48,49),(50,51)))))),(60,(58,(47,59)))))),(57,61)),(64,(63,65)))))))))),(18,19)))))))))),(15,23)))))),(8,12)));

TREE tnt_139 = (1,((10,(6,(16,(20,((4,(9,(21,(11,(17,(2,(7,(3,((13,(5,(22,(14,(68,(69,((31,(((24,43),(35,(25,36))),((((42,(26,27)),(70,(32,67))),(34,(37,(39,40)))),(33,(41,((28,38),(29,30))))))),(45,(((52,(46,((56,(((44,66),(53,54)),(55,(62,((48,49),(50,51)))))),(60,(58,(47,59)))))),(57,61)),(64,(63,65))))))))))),(18,19)))))))))),(15,23)))))),(8,12)));

TREE tnt_140 = (1,((10,(6,(16,(20,((21,(4,(9,(11,(17,(2,(7,(3,((13,(5,(22,(14,(68,(69,((31,((43,(24,(35,(25,36)))),((((42,(26,27)),(70,(32,67))),(34,(37,(39,40)))),(33,(41,((28,38),(29,30))))))),(45,(((52,(46,((56,(((44,66),(53,54)),(55,(62,((48,49),(50,51)))))),(60,(58,(47,59)))))),(57,61)),(64,(63,65))))))))))),(18,19)))))))))),(15,23)))))),(8,12)));

TREE tnt_141 = (1,((10,(6,(16,(20,((4,(9,(21,(11,(17,(2,(7,(3,(13,((14,(5,(22,(68,(69,((31,((43,(24,(35,(25,36)))),((((42,(26,27)),(70,(32,67))),(34,(37,(39,40)))),(33,(41,((28,38),(29,30))))))),(45,(((52,(46,((56,(((44,66),(53,54)),(55,(62,((48,49),(50,51)))))),(60,(58,(47,59)))))),(57,61)),(63,(64,65)))))))))),(18,19))))))))))),(15,23)))))),(8,12)));

TREE tnt_142 = (1,((10,(6,(16,(20,((4,(9,(21,(11,(17,(2,(7,(3,((13,(14,(5,(22,(68,(69,((31,((43,(24,(35,(25,36)))),((((42,(26,27)),(70,(32,67))),(34,(37,(39,40)))),(33,(41,((28,38),(29,30))))))),(45,(((52,(46,((56,(((44,66),(53,54)),(55,(62,((48,49),(50,51)))))),(60,(58,(47,59)))))),(57,61)),(63,(64,65))))))))))),(18,19)))))))))),(15,23)))))),(8,12)));

TREE tnt_143 = (1,((10,(6,(16,(20,((4,(9,(21,(11,(17,(2,(7,(3,(((5,(22,(68,(69,((31,((43,(24,(35,(25,36)))),((((42,(26,27)),(70,(32,67))),(34,(37,(39,40)))),(33,(41,((28,38),(29,30))))))),(45,(((52,(46,((56,(((44,66),(53,54)),(55,(62,((48,49),(50,51)))))),(60,(58,(47,59)))))),(57,61)),(63,(64,65))))))))),(13,14)),(18,19)))))))))),(15,23)))))),(8,12)));

TREE tnt_144 = (1,((10,(6,(16,(20,((4,(9,(21,(11,(17,(2,(7,(3,(13,((5,(22,(14,(68,(69,((31,((43,(24,(35,(25,36)))),((((42,(26,27)),(70,(32,67))),(34,(37,(39,40)))),(33,(41,((28,38),(29,30))))))),(45,(((52,(46,((56,(((44,66),(53,54)),(55,(62,((48,49),(50,51)))))),(60,(58,(47,59)))))),(57,61)),(63,(64,65)))))))))),(18,19))))))))))),(15,23)))))),(8,12)));

TREE tnt_145 = (1,((10,(6,(16,(20,((4,(9,(21,(11,(17,(2,(7,(3,(13,((5,((14,22),(68,(69,((31,((43,(24,(35,(25,36)))),((((42,(26,27)),(70,(32,67))),(34,(37,(39,40)))),(33,(41,((28,38),(29,30))))))),(45,(((52,(46,((56,(((44,66),(53,54)),(55,(62,((48,49),(50,51)))))),(60,(58,(47,59)))))),(57,61)),(63,(64,65))))))))),(18,19))))))))))),(15,23)))))),(8,12)));

TREE tnt_146 = (1,((10,(6,(16,(20,((4,(9,(21,(11,(17,(2,(7,(3,(13,((5,(22,(68,(14,(69,((31,((43,(24,(35,(25,36)))),((((42,(26,27)),(70,(32,67))),(34,(37,(39,40)))),(33,(41,((28,38),(29,30))))))),(45,(((52,(46,((56,(((44,66),(53,54)),(55,(62,((48,49),(50,51)))))),(60,(58,(47,59)))))),(57,61)),(63,(64,65)))))))))),(18,19))))))))))),(15,23)))))),(8,12)));

TREE tnt_147 = (1,((10,(6,(16,(20,((4,(9,(21,(11,(17,(2,(7,(3,(13,((5,(22,((14,68),(69,((31,((43,(24,(35,(25,36)))),((((42,(26,27)),(70,(32,67))),(34,(37,(39,40)))),(33,(41,((28,38),(29,30))))))),(45,(((52,(46,((56,(((44,66),(53,54)),(55,(62,((48,49),(50,51)))))),(60,(58,(47,59)))))),(57,61)),(63,(64,65))))))))),(18,19))))))))))),(15,23)))))),(8,12)));

TREE tnt_148 = (1,((10,(6,(16,(20,((4,(9,(21,(11,(17,(2,(7,(3,(13,((14,(5,(22,(68,(69,((31,((43,(24,(35,(25,36)))),((((42,(26,27)),(70,(32,67))),(34,(37,(39,40)))),(33,(41,((28,38),(29,30))))))),(45,(((52,((46,(56,(((44,66),(53,54)),(55,(62,((48,49),(50,51))))))),(58,(60,(47,59))))),(57,61)),(63,(64,65)))))))))),(18,19))))))))))),(15,23)))))),(8,12)));

TREE tnt_149 = (1,((10,(6,(16,(20,((4,(9,(21,(11,(17,(2,(7,(3,((13,(5,(22,(14,(68,(69,((31,((43,(24,(35,(25,36)))),((((42,(26,27)),(70,(32,67))),(34,(37,(39,40)))),(33,(41,((28,38),(29,30))))))),(45,(((52,(46,((56,(((44,66),(53,54)),(55,(62,((48,49),(50,51)))))),(60,(58,(47,59)))))),(57,61)),(63,(64,65))))))))))),(18,19)))))))))),(15,23)))))),(8,12)));

TREE tnt_150 = (1,((10,(6,(16,(20,((4,(9,(21,(11,(17,(2,(7,(3,((13,(5,((14,22),(68,(69,((31,((43,(24,(35,(25,36)))),((((42,(26,27)),(70,(32,67))),(34,(37,(39,40)))),(33,(41,((28,38),(29,30))))))),(45,(((52,(46,((56,(((44,66),(53,54)),(55,(62,((48,49),(50,51)))))),(60,(58,(47,59)))))),(57,61)),(63,(64,65)))))))))),(18,19)))))))))),(15,23)))))),(8,12)));

TREE tnt_151 = (1,((10,(6,(16,(20,((21,(4,(9,(11,(17,(2,(7,(3,(13,((5,(22,(68,(14,(69,((31,((43,(24,(35,(25,36)))),((((42,(26,27)),(70,(32,67))),(34,(37,(39,40)))),(33,(41,((28,38),(29,30))))))),(45,(((52,((46,(56,(((44,66),(53,54)),(55,(62,((48,49),(50,51))))))),(58,(60,(47,59))))),(57,61)),(64,(63,65)))))))))),(18,19))))))))))),(15,23)))))),(8,12)));

TREE tnt_152 = (1,((10,(6,(16,(20,((21,(4,(9,(11,(17,(2,(7,(3,(13,((5,(22,((14,68),(69,((31,((43,(24,(35,(25,36)))),((((42,(26,27)),(70,(32,67))),(34,(37,(39,40)))),(33,(41,((28,38),(29,30))))))),(45,(((52,((46,(56,(((44,66),(53,54)),(55,(62,((48,49),(50,51))))))),(58,(60,(47,59))))),(57,61)),(64,(63,65))))))))),(18,19))))))))))),(15,23)))))),(8,12)));

TREE tnt_153 = (1,((10,(6,(16,(20,((4,(9,(21,(11,(17,(2,(7,(3,(13,((5,(22,(68,(14,(69,((31,((43,(24,(35,(25,36)))),((((42,(26,27)),(70,(32,67))),(34,(37,(39,40)))),(33,(41,((28,38),(29,30))))))),(45,(((52,((46,(56,(((44,66),(53,54)),(55,(62,((48,49),(50,51))))))),(58,(60,(47,59))))),(57,61)),(64,(63,65)))))))))),(18,19))))))))))),(15,23)))))),(8,12)));

TREE tnt_154 = (1,((10,(6,(16,(20,((4,(9,(21,(11,(17,(2,(7,(3,(13,((5,(22,((14,68),(69,((31,((43,(24,(35,(25,36)))),((((42,(26,27)),(70,(32,67))),(34,(37,(39,40)))),(33,(41,((28,38),(29,30))))))),(45,(((52,((46,(56,(((44,66),(53,54)),(55,(62,((48,49),(50,51))))))),(58,(60,(47,59))))),(57,61)),(64,(63,65))))))))),(18,19))))))))))),(15,23)))))),(8,12)));

TREE tnt_155 = (1,((10,(6,(16,(20,((4,(9,(21,(11,(17,(2,(7,(3,((13,(5,(22,(68,(14,(69,((31,((43,(24,(35,(25,36)))),((((42,(26,27)),(70,(32,67))),(34,(37,(39,40)))),(33,(41,((28,38),(29,30))))))),(45,(((52,((46,(56,(((44,66),(53,54)),(55,(62,((48,49),(50,51))))))),(58,(60,(47,59))))),(57,61)),(64,(63,65))))))))))),(18,19)))))))))),(15,23)))))),(8,12)));

TREE tnt_156 = (1,((10,(6,(16,(20,((4,(9,(21,(11,(17,(2,(7,(3,(13,((5,(22,(14,(68,(69,((31,((43,(24,(35,(25,36)))),((((42,(26,27)),(70,(32,67))),(34,(37,(39,40)))),(33,(41,((28,38),(29,30))))))),(45,(((52,((46,(56,(((44,66),(53,54)),(55,(62,((48,49),(50,51))))))),(58,(60,(47,59))))),(57,61)),(64,(63,65)))))))))),(18,19))))))))))),(15,23)))))),(8,12)));

TREE tnt_157 = (1,((10,(6,(16,(20,((4,(9,(21,(11,(17,(2,(7,(3,(13,((5,((14,22),(68,(69,((31,((43,(24,(35,(25,36)))),((((42,(26,27)),(70,(32,67))),(34,(37,(39,40)))),(33,(41,((28,38),(29,30))))))),(45,(((52,((46,(56,(((44,66),(53,54)),(55,(62,((48,49),(50,51))))))),(58,(60,(47,59))))),(57,61)),(64,(63,65))))))))),(18,19))))))))))),(15,23)))))),(8,12)));

TREE tnt_158 = (1,((10,(6,(16,(20,((4,(9,(21,(11,(17,(2,(7,(3,((13,(5,(22,((14,68),(69,((31,((43,(24,(35,(25,36)))),((((42,(26,27)),(70,(32,67))),(34,(37,(39,40)))),(33,(41,((28,38),(29,30))))))),(45,(((52,((46,(56,(((44,66),(53,54)),(55,(62,((48,49),(50,51))))))),(58,(60,(47,59))))),(57,61)),(64,(63,65)))))))))),(18,19)))))))))),(15,23)))))),(8,12)));

TREE tnt_159 = (1,((10,(6,(16,(20,((4,(9,(21,(11,(17,(2,(7,(3,(13,((5,(22,((14,68),(69,((31,(((24,43),(35,(25,36))),((((42,(26,27)),(70,(32,67))),(34,(37,(39,40)))),(33,(41,((28,38),(29,30))))))),(45,(((52,((46,(56,(((44,66),(53,54)),(55,(62,((48,49),(50,51))))))),(58,(60,(47,59))))),(57,61)),(64,(63,65))))))))),(18,19))))))))))),(15,23)))))),(8,12)));

TREE tnt_160 = (1,((10,(6,(16,(20,((4,(9,(21,(11,(17,(2,(7,(3,((14,(13,(5,(22,(68,(69,((31,((43,(24,(35,(25,36)))),((((42,(26,27)),(70,(32,67))),(34,(37,(39,40)))),(33,(41,((28,38),(29,30))))))),(45,(((52,((46,(56,(((44,66),(53,54)),(55,(62,((48,49),(50,51))))))),(58,(60,(47,59))))),(57,61)),(64,(63,65))))))))))),(18,19)))))))))),(15,23)))))),(8,12)));

TREE tnt_161 = (1,((10,(6,(16,(20,((21,(4,(9,(11,(17,(2,(7,(3,((13,(5,(22,(14,(68,(69,((31,(((24,43),(35,(25,36))),((((42,(26,27)),(70,(32,67))),(34,(37,(39,40)))),(33,(41,((28,38),(29,30))))))),(45,(((52,(46,((56,(((44,66),(53,54)),(55,(62,((48,49),(50,51)))))),(58,(60,(47,59)))))),(57,61)),(64,(63,65))))))))))),(18,19)))))))))),(15,23)))))),(8,12)));

TREE tnt_162 = (1,((10,(6,(16,(20,((21,(4,(9,(11,(17,(2,(7,(3,((13,(5,(14,(22,(68,(69,((31,(((24,43),(35,(25,36))),((((42,(26,27)),(70,(32,67))),(34,(37,(39,40)))),(33,(41,((28,38),(29,30))))))),(45,(((52,(46,((56,(((44,66),(53,54)),(55,(62,((48,49),(50,51)))))),(58,(60,(47,59)))))),(57,61)),(64,(63,65))))))))))),(18,19)))))))))),(15,23)))))),(8,12)));

TREE tnt_163 = (1,((10,(6,(16,(20,((21,(4,(9,(11,(17,(2,(7,(3,((13,((5,14),(22,(68,(69,((31,(((24,43),(35,(25,36))),((((42,(26,27)),(70,(32,67))),(34,(37,(39,40)))),(33,(41,((28,38),(29,30))))))),(45,(((52,(46,((56,(((44,66),(53,54)),(55,(62,((48,49),(50,51)))))),(58,(60,(47,59)))))),(57,61)),(64,(63,65)))))))))),(18,19)))))))))),(15,23)))))),(8,12)));

TREE tnt_164 = (1,((10,(6,(16,(20,((21,(4,(9,(11,(17,(2,(7,(3,((13,(5,(22,(68,(14,(69,((31,(((24,43),(35,(25,36))),((((42,(26,27)),(70,(32,67))),(34,(37,(39,40)))),(33,(41,((28,38),(29,30))))))),(45,(((52,(46,((56,(((44,66),(53,54)),(55,(62,((48,49),(50,51)))))),(58,(60,(47,59)))))),(57,61)),(64,(63,65))))))))))),(18,19)))))))))),(15,23)))))),(8,12)));

TREE tnt_165 = (1,((10,(6,(16,(20,((21,(4,(9,(11,(17,(2,(7,(3,((13,(5,(22,((14,68),(69,((31,(((24,43),(35,(25,36))),((((42,(26,27)),(70,(32,67))),(34,(37,(39,40)))),(33,(41,((28,38),(29,30))))))),(45,(((52,(46,((56,(((44,66),(53,54)),(55,(62,((48,49),(50,51)))))),(58,(60,(47,59)))))),(57,61)),(64,(63,65)))))))))),(18,19)))))))))),(15,23)))))),(8,12)));

TREE tnt_166 = (1,((10,(6,(16,(20,((21,(4,(9,(11,(17,(2,(7,(3,((13,(5,(22,((14,68),(69,((31,(((24,43),(35,(25,36))),((((42,(26,27)),(70,(32,67))),(34,(37,(39,40)))),(33,(41,((28,38),(29,30))))))),(45,(((52,((46,(56,(((44,66),(53,54)),(55,(62,((48,49),(50,51))))))),(58,(60,(47,59))))),(57,61)),(64,(63,65)))))))))),(18,19)))))))))),(15,23)))))),(8,12)));

TREE tnt_167 = (1,((10,(6,(16,(20,((4,(9,(21,(11,(17,(2,(7,(3,((13,(5,(22,(68,(14,(69,((31,((43,(24,(35,(25,36)))),((((42,(26,27)),(70,(32,67))),(34,(37,(39,40)))),(33,(41,((28,38),(29,30))))))),(45,(((52,(46,((56,(((44,66),(53,54)),(55,(62,((48,49),(50,51)))))),(58,(60,(47,59)))))),(57,61)),(64,(63,65))))))))))),(18,19)))))))))),(15,23)))))),(8,12)));

TREE tnt_168 = (1,((10,(6,(16,(20,((4,(9,(21,(11,(17,(2,(7,(3,((14,(13,(5,(22,(68,(69,((31,((43,(24,(35,(25,36)))),((((42,(26,27)),(70,(32,67))),(34,(37,(39,40)))),(33,(41,((28,38),(29,30))))))),(45,(((52,(46,((56,(((44,66),(53,54)),(55,(62,((48,49),(50,51)))))),(58,(60,(47,59)))))),(57,61)),(64,(63,65))))))))))),(18,19)))))))))),(15,23)))))),(8,12)));

TREE tnt_169 = (1,((10,(6,(16,(20,((4,(9,(21,(11,(17,(2,(7,(3,((13,(14,(5,(22,(68,(69,((31,((43,(24,(35,(25,36)))),((((42,(26,27)),(70,(32,67))),(34,(37,(39,40)))),(33,(41,((28,38),(29,30))))))),(45,(((52,(46,((56,(((44,66),(53,54)),(55,(62,((48,49),(50,51)))))),(58,(60,(47,59)))))),(57,61)),(64,(63,65))))))))))),(18,19)))))))))),(15,23)))))),(8,12)));

TREE tnt_170 = (1,((10,(6,(16,(20,((4,(9,(21,(11,(17,(2,(7,(3,((13,((5,14),(22,(68,(69,((31,((43,(24,(35,(25,36)))),((((42,(26,27)),(70,(32,67))),(34,(37,(39,40)))),(33,(41,((28,38),(29,30))))))),(45,(((52,(46,((56,(((44,66),(53,54)),(55,(62,((48,49),(50,51)))))),(58,(60,(47,59)))))),(57,61)),(64,(63,65)))))))))),(18,19)))))))))),(15,23)))))),(8,12)));

TREE tnt_171 = (1,((10,(6,(16,(20,((4,(9,(21,(11,(17,(2,(7,(3,((13,(5,(22,(14,(68,(69,((31,((43,(24,(35,(25,36)))),((((42,(26,27)),(70,(32,67))),(34,(37,(39,40)))),(33,(41,((28,38),(29,30))))))),(45,(((52,(46,((56,(((44,66),(53,54)),(55,(62,((48,49),(50,51)))))),(58,(60,(47,59)))))),(57,61)),(64,(63,65))))))))))),(18,19)))))))))),(15,23)))))),(8,12)));

TREE tnt_172 = (1,((10,(6,(16,(20,((4,(9,(21,(11,(17,(2,(7,(3,((13,(5,(22,(68,(14,(69,((31,((43,(24,(35,(25,36)))),((((42,(26,27)),(70,(32,67))),(34,(37,(39,40)))),(33,(41,((28,38),(29,30))))))),(45,(((52,(46,((56,(((44,66),(53,54)),(55,(62,((48,49),(50,51)))))),(60,(58,(47,59)))))),(57,61)),(64,(63,65))))))))))),(18,19)))))))))),(15,23)))))),(8,12)));

TREE tnt_173 = (1,((10,(6,(16,(20,((4,(9,(21,(11,(17,(2,(7,(3,((13,(5,(22,((14,68),(69,((31,((43,(24,(35,(25,36)))),((((42,(26,27)),(70,(32,67))),(34,(37,(39,40)))),(33,(41,((28,38),(29,30))))))),(45,(((52,(46,((56,(((44,66),(53,54)),(55,(62,((48,49),(50,51)))))),(58,(60,(47,59)))))),(57,61)),(64,(63,65)))))))))),(18,19)))))))))),(15,23)))))),(8,12)));

TREE tnt_174 = (1,((10,(6,(16,(20,((4,(9,(21,(11,(17,(2,(7,(3,((13,(14,(5,(22,(68,(69,((31,((43,(24,(35,(25,36)))),((((42,(26,27)),(70,(32,67))),(34,(37,(39,40)))),(33,(41,((28,38),(29,30))))))),(45,(((52,((46,(56,(((44,66),(53,54)),(55,(62,((48,49),(50,51))))))),(58,(60,(47,59))))),(57,61)),(64,(63,65))))))))))),(18,19)))))))))),(15,23)))))),(8,12)));

TREE tnt_175 = (1,((10,(6,(16,(20,((4,(9,(21,(11,(17,(2,(7,(3,((13,((5,14),(22,(68,(69,((31,((43,(24,(35,(25,36)))),((((42,(26,27)),(70,(32,67))),(34,(37,(39,40)))),(33,(41,((28,38),(29,30))))))),(45,(((52,((46,(56,(((44,66),(53,54)),(55,(62,((48,49),(50,51))))))),(58,(60,(47,59))))),(57,61)),(64,(63,65)))))))))),(18,19)))))))))),(15,23)))))),(8,12)));

TREE tnt_176 = (1,((10,(6,(16,(20,((4,(9,(21,(11,(17,(2,(7,(3,((13,((5,14),(22,(68,(69,((31,((43,(24,(35,(25,36)))),((((42,(26,27)),(70,(32,67))),(34,(37,(39,40)))),(33,(41,((28,38),(29,30))))))),(45,(((52,(46,((56,(((44,66),(53,54)),(55,(62,((48,49),(50,51)))))),(60,(58,(47,59)))))),(57,61)),(64,(63,65)))))))))),(18,19)))))))))),(15,23)))))),(8,12)));

TREE tnt_177 = (1,((10,(6,(16,(20,((4,(9,(21,(11,(17,(2,(7,(3,((13,(5,(22,(14,(68,(69,((31,((43,(24,(35,(25,36)))),((((42,(26,27)),(70,(32,67))),(34,(37,(39,40)))),(33,(41,((28,38),(29,30))))))),(45,(((52,((46,(56,(((44,66),(53,54)),(55,(62,((48,49),(50,51))))))),(58,(60,(47,59))))),(57,61)),(64,(63,65))))))))))),(18,19)))))))))),(15,23)))))),(8,12)));

TREE tnt_178 = (1,((10,(6,(16,(20,((4,(9,(21,(11,(17,(2,(7,(3,((13,(5,((14,22),(68,(69,((31,((43,(24,(35,(25,36)))),((((42,(26,27)),(70,(32,67))),(34,(37,(39,40)))),(33,(41,((28,38),(29,30))))))),(45,(((52,((46,(56,(((44,66),(53,54)),(55,(62,((48,49),(50,51))))))),(58,(60,(47,59))))),(57,61)),(64,(63,65)))))))))),(18,19)))))))))),(15,23)))))),(8,12)));

TREE tnt_179 = (1,((10,(6,(16,(20,((4,(9,(21,(11,(17,(2,(7,(3,((13,(5,(14,(22,(68,(69,((31,((43,(24,(35,(25,36)))),((((42,(26,27)),(70,(32,67))),(34,(37,(39,40)))),(33,(41,((28,38),(29,30))))))),(45,(((52,((46,(56,(((44,66),(53,54)),(55,(62,((48,49),(50,51))))))),(58,(60,(47,59))))),(57,61)),(64,(63,65))))))))))),(18,19)))))))))),(15,23)))))),(8,12)));

TREE tnt_180 = (1,((10,(6,(16,(20,((4,(9,(21,(11,(17,(2,(7,(3,((13,(5,(22,(68,((14,69),((31,((43,(24,(35,(25,36)))),((((42,(26,27)),(70,(32,67))),(34,(37,(39,40)))),(33,(41,((28,38),(29,30))))))),(45,(((52,((46,(56,(((44,66),(53,54)),(55,(62,((48,49),(50,51))))))),(58,(60,(47,59))))),(57,61)),(64,(63,65)))))))))),(18,19)))))))))),(15,23)))))),(8,12)));

TREE tnt_181 = (1,((10,(6,(16,(20,((4,(9,(21,(11,(17,(2,(7,(3,((13,(5,(14,(22,(68,(69,((31,((43,(24,(35,(25,36)))),((((42,(26,27)),(70,(32,67))),(34,(37,(39,40)))),(33,(41,((28,38),(29,30))))))),(45,(((52,(46,((56,(((44,66),(53,54)),(55,(62,((48,49),(50,51)))))),(60,(58,(47,59)))))),(57,61)),(64,(63,65))))))))))),(18,19)))))))))),(15,23)))))),(8,12)));

TREE tnt_182 = (1,((10,(6,(16,(20,((4,(9,(21,(11,(17,(2,(7,(3,((13,(5,(22,(68,((14,69),((31,((43,(24,(35,(25,36)))),((((42,(26,27)),(70,(32,67))),(34,(37,(39,40)))),(33,(41,((28,38),(29,30))))))),(45,(((52,(46,((56,(((44,66),(53,54)),(55,(62,((48,49),(50,51)))))),(60,(58,(47,59)))))),(57,61)),(64,(63,65)))))))))),(18,19)))))))))),(15,23)))))),(8,12)));

TREE tnt_183 = (1,((10,(6,(16,(20,((4,(9,(21,(11,(17,(2,(7,(3,((13,(5,(22,((14,68),(69,((31,((43,(24,(35,(25,36)))),((((42,(26,27)),(70,(32,67))),(34,(37,(39,40)))),(33,(41,((28,38),(29,30))))))),(45,(((52,(46,((56,(((44,66),(53,54)),(55,(62,((48,49),(50,51)))))),(60,(58,(47,59)))))),(57,61)),(64,(63,65)))))))))),(18,19)))))))))),(15,23)))))),(8,12)));

TREE tnt_184 = (1,((10,(6,(16,(20,((4,(9,(21,(11,(17,(2,(7,(3,((13,(5,(14,(22,(68,(69,((31,(((24,43),(35,(25,36))),((((42,(26,27)),(70,(32,67))),(34,(37,(39,40)))),(33,(41,((28,38),(29,30))))))),(45,(((52,(46,((56,(((44,66),(53,54)),(55,(62,((48,49),(50,51)))))),(60,(58,(47,59)))))),(57,61)),(64,(63,65))))))))))),(18,19)))))))))),(15,23)))))),(8,12)));

TREE tnt_185 = (1,((10,(6,(16,(20,((4,(21,(9,(11,(17,(2,(7,(3,((13,(5,(22,((14,68),(69,((31,((43,(24,(35,(25,36)))),((((42,(26,27)),(70,(32,67))),(34,(37,(39,40)))),(33,(41,((28,38),(29,30))))))),(45,(((52,(46,((56,(((44,66),(53,54)),(55,(62,((48,49),(50,51)))))),(60,(58,(47,59)))))),(57,61)),(64,(63,65)))))))))),(18,19)))))))))),(15,23)))))),(8,12)));

TREE tnt_186 = (1,((10,(6,(16,(20,((4,(9,(21,(11,(17,(2,(7,(3,((13,(5,(22,((14,68),(69,((31,(((24,43),(35,(25,36))),((((42,(26,27)),(70,(32,67))),(34,(37,(39,40)))),(33,(41,((28,38),(29,30))))))),(45,(((52,(46,((56,(((44,66),(53,54)),(55,(62,((48,49),(50,51)))))),(60,(58,(47,59)))))),(57,61)),(64,(63,65)))))))))),(18,19)))))))))),(15,23)))))),(8,12)));

TREE tnt_187 = (1,((10,(6,(16,(20,((4,(9,(21,(11,(17,(2,(7,(3,((13,((5,14),(22,(68,(69,((31,(((24,43),(35,(25,36))),((((42,(26,27)),(70,(32,67))),(34,(37,(39,40)))),(33,(41,((28,38),(29,30))))))),(45,(((52,(46,((56,(((44,66),(53,54)),(55,(62,((48,49),(50,51)))))),(60,(58,(47,59)))))),(57,61)),(64,(63,65)))))))))),(18,19)))))))))),(15,23)))))),(8,12)));

TREE tnt_188 = (1,((10,(6,(16,(20,((4,(9,(21,(11,(17,(2,(7,(3,((14,(13,(5,(22,(68,(69,((31,(((24,43),(35,(25,36))),((((42,(26,27)),(70,(32,67))),(34,(37,(39,40)))),(33,(41,((28,38),(29,30))))))),(45,(((52,(46,((56,(((44,66),(53,54)),(55,(62,((48,49),(50,51)))))),(60,(58,(47,59)))))),(57,61)),(64,(63,65))))))))))),(18,19)))))))))),(15,23)))))),(8,12)));

TREE tnt_189 = (1,((10,(6,(16,(20,((4,(9,(21,(11,(17,(2,(7,(3,((13,(5,((14,22),(68,(69,((31,(((24,43),(35,(25,36))),((((42,(26,27)),(70,(32,67))),(34,(37,(39,40)))),(33,(41,((28,38),(29,30))))))),(45,(((52,(46,((56,(((44,66),(53,54)),(55,(62,((48,49),(50,51)))))),(60,(58,(47,59)))))),(57,61)),(64,(63,65)))))))))),(18,19)))))))))),(15,23)))))),(8,12)));

TREE tnt_190 = (1,((10,(6,(16,(20,((4,9,21,(11,(17,(2,(7,(3,5,13,14,22,68,69,(18,19),((31,((24,43,(35,(25,36))),((((42,(26,27)),(70,(32,67))),(34,(37,(39,40)))),(33,(41,((28,38),(29,30))))))),(45,(((52,(46,(56,(((44,66),(53,54)),(55,(62,((48,49),(50,51)))))),(58,60,(47,59)))),(57,61)),(63,64,65)))))))))),(15,23)))))),(8,12)));

TREE tnt_191 = (1,((10,(6,(16,(20,((4,(9,(21,(11,(17,(2,(7,(3,((13,(5,(14,22,68,(69,((31,(((24,43),(35,(25,36))),((((42,(26,27)),(70,(32,67))),(34,(37,(39,40)))),(33,(41,((28,38),(29,30))))))),(45,(((52,(46,((56,(((44,66),(53,54)),(55,(62,((48,49),(50,51)))))),(58,(60,(47,59)))))),(57,61)),(63,64,65)))))))),(18,19)))))))))),(15,23)))))),(8,12)));

END;

BEGIN ASSUMPTIONS;

TYPESET * UNTITLED = unord: 1 - 350;

END;

BEGIN MESQUITECHARMODELS;

ProbModelSet * UNTITLED = 'Mk1 (est.)': 1 - 350;

END;

Begin MESQUITE;

MESQUITESCRIPTVERSION 2;

TITLE AUTO;

tell ProjectCoordinator;

timeSaved 1551344951003;

getEmployee #mesquite.minimal.ManageTaxa.ManageTaxa;

tell It;

setID 0 470337572729042143;

tell It;

setDefaultOrder 0 1 2 3 4 5 6 7 8 9 10 11 12 13 14 15 16 17 18 19 20 21 22 23 24 25 26 27 28 29 30 31 32 33 34 35 36 37 38 39 40 41 42 43 44 45 46 47 48 49 50 51 52 53 54 55 56 57 58 59 60 61 62 63 64 65 66 67 69 68;

attachments ;

endTell;

endTell;

getEmployee #mesquite.charMatrices.ManageCharacters.ManageCharacters;

tell It;

setID 0 2195790664221188916;

mqVersion 320;

checksumv 0 3 2724627649 null getNumChars 350 numChars 350 getNumTaxa 70 numTaxa 70 short true bits 2305843009213694079 states 127 sumSquaresStatesOnly 94625.0 sumSquares -6.041308684139878E20 longCompressibleToShort false usingShortMatrix true NumFiles 1 NumMatrices 1;

mqVersion;

endTell;

getWindow;

tell It;

suppress;

setResourcesState false false 221;

setPopoutState 300;

setExplanationSize 0;

setAnnotationSize 0;

setFontIncAnnot 0;

setFontIncExp 0;

setSize 1920 952;

setLocation -9 0;

setFont SanSerif;

setFontSize 10;

getToolPalette;

tell It;

endTell;

desuppress;

endTell;

getEmployee #mesquite.trees.BasicTreeWindowCoord.BasicTreeWindowCoord;

tell It;

makeTreeWindow #470337572729042143 #mesquite.trees.BasicTreeWindowMaker.BasicTreeWindowMaker;

tell It;

suppressEPCResponse;

setTreeSource #mesquite.trees.StoredTrees.StoredTrees;

tell It;

setTreeBlock 1;

setTreeBlockID 0168df071ab91;

toggleUseWeights off;

endTell;

setAssignedID 985.1549930020616.4565963579461245254;

getTreeWindow;

tell It;

setExplanationSize 30;

setAnnotationSize 20;

setFontIncAnnot 0;

setFontIncExp 0;

setSize 1699 880;

setLocation -9 0;

setFont SanSerif;

setFontSize 10;

getToolPalette;

tell It;

endTell;

getTreeDrawCoordinator #mesquite.trees.BasicTreeDrawCoordinator.BasicTreeDrawCoordinator;

tell It;

suppress;

setTreeDrawer #mesquite.trees.SquareLineTree.SquareLineTree;

tell It;

setNodeLocs #mesquite.trees.NodeLocsStandard.NodeLocsStandard;

tell It;

branchLengthsToggle off;

toggleScale on;

toggleBroadScale off;

toggleCenter on;

toggleEven on;

setFixedTaxonDistance 0;

endTell;

setEdgeWidth 4;

showEdgeLines on;

orientUp;

endTell;

setBackground White;

setBranchColor Black;

showNodeNumbers off;

showBranchColors on;

labelBranchLengths off;

centerBrLenLabels on;

showBrLensUnspecified on;

showBrLenLabelsOnTerminals on;

setBrLenLabelColor 0 0 255;

setNumBrLenDecimals 6;

desuppress;

getEmployee #mesquite.trees.BasicDrawTaxonNames.BasicDrawTaxonNames;

tell It;

setColor Black;

toggleColorPartition off;

toggleColorAssigned on;

toggleShadePartition off;

toggleShowFootnotes on;

toggleNodeLabels on;

toggleCenterNodeNames off;

toggleShowNames on;

namesAngle ?;

endTell;

endTell;

setTreeNumber 190;

setTree '(1,((8,12),(10,(6,(16,(20,((15,23),(4,9,21,(11,(17,(2,(7,(3,5,13,14,22,68,69,(18,19),((31,((24,43,(35,(25,36))),((33,(41,((28,38),(29,30)))),((34,(37,(39,40))),((42,(26,27)),(70,(32,67))))))),(45,((63,64,65),((57,61),(52,(46,(58,60,(47,59)),(56,(((44,66),(53,54)),(55,(62,((48,49),(50,51)))))))))))))))))))))))));';

setDrawingSizeMode 0;

toggleLegendFloat on;

scale 0;

toggleTextOnTree off;

togglePrintName off;

showWindow;

newAssistant #mesquite.ancstates.TraceCharacterHistory.TraceCharacterHistory;

tell It;

suspend ;

setDisplayMode #mesquite.ancstates.ShadeStatesOnTree.ShadeStatesOnTree;

tell It;

toggleLabels off;

togglePredictions off;

toggleGray off;

endTell;

setHistorySource #mesquite.ancstates.RecAncestralStates.RecAncestralStates;

tell It;

getCharacterSource #mesquite.charMatrices.CharSrcCoordObed.CharSrcCoordObed;

tell It;

setCharacterSource #mesquite.charMatrices.StoredCharacters.StoredCharacters;

tell It;

setDataSet #2195790664221188916;

endTell;

endTell;

setMethod #mesquite.parsimony.ParsAncestralStates.ParsAncestralStates;

tell It;

setModelSource #mesquite.parsimony.CurrentParsModels.CurrentParsModels;

toggleMPRsMode off;

endTell;

toggleShowSelectedOnly off;

endTell;

setCharacter 230;

setMapping 1;

toggleShowLegend on;

setColorMode 0;

toggleWeights on;

setInitialOffsetX 483;

setInitialOffsetY -216;

setLegendWidth 142;

setLegendHeight 164;

resume ;

endTell;

endTell;

desuppressEPCResponse;

getEmployee #mesquite.trees.ColorBranches.ColorBranches;

tell It;

setColor Red;

removeColor off;

endTell;

getEmployee #mesquite.ornamental.BranchNotes.BranchNotes;

tell It;

setAlwaysOn off;

endTell;

getEmployee #mesquite.ornamental.ColorTreeByPartition.ColorTreeByPartition;

tell It;

colorByPartition off;

endTell;

getEmployee #mesquite.ornamental.DrawTreeAssocDoubles.DrawTreeAssocDoubles;

tell It;

setOn on;

setDigits 4;

writeAsPercentage off;

toggleCentred off;

toggleHorizontal on;

toggleWhiteEdges on;

toggleShowOnTerminals on;

setFontSize 10;

setOffset 0 0;

endTell;

getEmployee #mesquite.ornamental.DrawTreeAssocStrings.DrawTreeAssocStrings;

tell It;

setOn on;

toggleCentred on;

toggleHorizontal on;

setFontSize 10;

setOffset 0 0;

toggleShowOnTerminals on;

endTell;

getEmployee #mesquite.trees.TreeInfoValues.TreeInfoValues;

tell It;

panelOpen false;

endTell;

endTell;

endTell;

getEmployee #mesquite.charMatrices.BasicDataWindowCoord.BasicDataWindowCoord;

tell It;

showDataWindow #2195790664221188916 #mesquite.charMatrices.BasicDataWindowMaker.BasicDataWindowMaker;

tell It;

getWindow;

tell It;

setExplanationSize 30;

setAnnotationSize 20;

setFontIncAnnot 0;

setFontIncExp 0;

setSize 1699 880;

setLocation -9 0;

setFont SanSerif;

setFontSize 10;

getToolPalette;

tell It;

setTool mesquite.charMatrices.BasicDataWindowMaker.BasicDataWindow.ibeam;

endTell;

setActive;

setTool mesquite.charMatrices.BasicDataWindowMaker.BasicDataWindow.ibeam;

colorCells #mesquite.charMatrices.NoColor.NoColor;

colorRowNames #mesquite.charMatrices.TaxonGroupColor.TaxonGroupColor;

colorColumnNames #mesquite.charMatrices.CharGroupColor.CharGroupColor;

colorText #mesquite.charMatrices.NoColor.NoColor;

setBackground White;

toggleShowNames on;

toggleShowTaxonNames on;

toggleTight off;

toggleThinRows off;

toggleShowChanges on;

toggleSeparateLines off;

toggleShowStates on;

toggleAutoWCharNames on;

toggleAutoTaxonNames off;

toggleShowDefaultCharNames off;

toggleConstrainCW on;

toggleBirdsEye off;

toggleShowPaleGrid off;

toggleShowPaleCellColors off;

toggleShowPaleExcluded off;

togglePaleInapplicable on;

toggleShowBoldCellText off;

toggleAllowAutosize on;

toggleColorsPanel off;

toggleDiagonal on;

setDiagonalHeight 80;

toggleLinkedScrolling on;

toggleScrollLinkedTables off;

endTell;

showWindow;

getWindow;

tell It;

forceAutosize;

endTell;

getEmployee #mesquite.charMatrices.AlterData.AlterData;

tell It;

toggleBySubmenus off;

endTell;

getEmployee #mesquite.charMatrices.ColorByState.ColorByState;

tell It;

setStateLimit 9;

toggleUniformMaximum on;

endTell;

getEmployee #mesquite.charMatrices.ColorCells.ColorCells;

tell It;

setColor Red;

removeColor off;

endTell;

getEmployee #mesquite.categ.StateNamesStrip.StateNamesStrip;

tell It;

showStrip off;

endTell;

getEmployee #mesquite.charMatrices.AnnotPanel.AnnotPanel;

tell It;

togglePanel off;

endTell;

getEmployee #mesquite.charMatrices.CharReferenceStrip.CharReferenceStrip;

tell It;

showStrip off;

endTell;

getEmployee #mesquite.charMatrices.QuickKeySelector.QuickKeySelector;

tell It;

autotabOff;

endTell;

getEmployee #mesquite.charMatrices.SelSummaryStrip.SelSummaryStrip;

tell It;

showStrip off;

endTell;

getEmployee #mesquite.categ.SmallStateNamesEditor.SmallStateNamesEditor;

tell It;

panelOpen true;

endTell;

endTell;

endTell;

endTell;

end;

## Supplementary Data S3. Occurrence ages for branch length calculations

|  | FAD | LAD |
| --- | --- | --- |
| *Acristavus gagslarsoni* | 80.6 | 79.8 |
| *Adynomosaurus arcanus* | 70.1 | 68 |
| *Amurosaurus riabinini* | 70.1 | 68 |
| *Aralosaurus tuberiferus* | 86.3 | 81 |
| *Arenysaurus ardevoli* | 67.7 | 66 |
| *Bactrosaurus johnsoni* | 76.3 | 69.91 |
| *Blasisaurus canudoi* | 67.7 | 66 |
| *Brachylophosaurus canadensis* | 78.5 | 78.2 |
| *Canardia garonnensis* | 69.91 | 66 |
| *Charonosaurus jiayinensis* | 69.91 | 66 |
| *Claosaurus agilis* | 87.86 | 86.26 |
| *Corythosaurus casuarius* | 77.03 | 76.39 |
| *Corythosaurus intermedius* | 77.03 | 76.39 |
| *Edmontosaurus annectens* | 67.1 | 66 |
| *Edmontosaurus regalis* | 72.5 | 71 |
| *Eolambia caroljonesa* | 95 | 93.9 |
| *Eotrachodon orientalis* | 84 | 83 |
| *Equijubus normani* | 113 | 100.5 |
| *Gilmoreosaurus mongliensis* | 76.3 | 69.91 |
| *Gryposaurus latidens* | 81 | 80.6 |
| *Gryposaurus monumentensis* | 75.6 | 75.5 |
| *Gryposaurus notabilis* | 76.3 | 76.1 |
| *Hadrosaurus foulkii* | 83.6 | 72.1 |
| *Kamuysaurus japonicus* | 72.1 | 69.9 |
| *Hypacrosaurus altispinus* | 71 | 68.4 |
| *Hypacrosaurus stebingeri* | 76.1 | 76 |
| *Iguanodon bernissartensis* | 127.2 | 110 |
| *Jaxartosaurus aralensis* | 86.3 | 85.56 |
| *Jintasaurus meniscus* | 113 | 100.5 |
| *Jinzhousaurus yangi* | 119 | 113 |
| *Kerberosaurus manakini* | 72.1 | 67.96 |
| *Kritosaurus navajovius* | 76.2 | 73.5 |
| *Laiyangosaurus youngi* | 73.5 | 73.5 |
| *Lambeosaurus lambei* | 76.6 | 76.3 |
| *Lambeosaurus magnicristatus* | 76.2 | 76.1 |
| *Levnesovia transoxiana* | 92.9 | 90.6 |
| *Lophorhothon atopus* | 83 | 82 |
| *Magnapaulia laticauda* | 73.6 | 73 |
| *Maiasaura peeblesorum* | 77.2 | 76.5 |
| *Mantellisaurus atherfieldensis* | 129.4 | 127.2 |
| *Nanningosaurus dashiensis* | 100.5 | 66 |
| *Nanyangosaurus zhugeii* | 91.85 | 76.38 |
| *Nipponosaurus sahalinensis* | 85 | 81 |
| *Olorotitan arharensi* | 72.1 | 69.9 |
| *Ouranosaurus nigeriensis* | 129.4 | 113 |
| *Pararhabdodon isonense* | 72.1 | 69.9 |
| *Parasaurolophus cyrtocristatus* | 75.4 | 75.3 |
| *Parasaurolophus tubicen* | 73.6 | 73.5 |
| *Parasaurolophus walkeri* | 76.7 | 76.6 |
| *Plesiohadros djadokhtaensis* | 75 | 71 |
| *Probactrosaurus gobiensis* | 129.4 | 100.5 |
| *Probrachylophosaurus bergei* | 78.5 | 78.2 |
| *Prosaurolophus maximus* | 75.7 | 74.1 |
| *Rhinorex condrupus* | 75.88 | 75.15 |
| *Sahaliyania elunchunorum* | 69.91 | 66 |
| *Saurolophus angustirostris* | 76.38 | 66 |
| *Saurolophus osborni* | 76.3 | 69.9 |
| *Secernosaurus koerneri* | 76.38 | 69.9 |
| *Shantungosaurus giganteus* | 81 | 72.1 |
| *Sirindhorna khoratensis* | 125 | 113 |
| *Tanius sinensis* | 73 | 69.23 |
| *Telmatosaurus transsylvanicus* | 72.1 | 69.9 |
| *Tethyshadros insularis* | 76.3 | 69.91 |
| *Tsintaosaurus spinorhinus* | 84.4 | 81 |
| *Velafrons coahuilensis* | 76.3 | 72.1 |
| *Wulagasaurus dongi* | 69.91 | 66 |
| *Xuwulong yueluni* | 125 | 100.5 |
| *Yunganglong datongensis* | 100.5 | 93.9 |
| *Zhanghenglong yangchengensis* | 84.94 | 84.52 |
| *Zuoyunlong huangi* | 100.5 | 93.9 |

## Supplementary Data S4. Tree file for DEC analysis (additive)

((((((((Edmontosaurus_annectens:6.4,Edmontosaurus_regalis:1):9.5,Shantungosaurus_giganteus:1):4.4,((Kerberosaurus_manakini:13.3,Laiyangosaurus_youngi:1):1,Kamuysaurus_japonicus:13.3):1):1,(((Saurolophus_angustirostris:1,Saurolophus_osborni:1.08):1,Prosaurolophus_maximus:1.68):7.62,Lophorhothon_atopus:1):2.4):1,((((Gryposaurus_latidens:1,Rhinorex_condrupus:6.12):1,(Gryposaurus_monumentensis:1.7,Gryposaurus_notabilis:1):5.7):1,Secernosaurus_koerneri:5.62):1,Kritosaurus_navajovius:5.8):4.4):1,(((Brachylophosaurus_canadensis:1,Probrachylophosaurus_bergei:1):1,Maiasaura_peeblesorum:2.3):3.1,(Acristavus_gagslarsoni:1,Wulagasaurus_dongi:11.69):1):4.8):1,Hadrosaurus_foulkii:1.8):2.9,Lambeosaurinae:1);

## Supplementary Data S5. Tree file for DEC analysis (zero-branch lengths additive)

((((((((Edmontosaurus_annectens:5.4,Edmontosaurus_regalis:1):8.5,Shantungosaurus_giganteus:1):3.4,((Kerberosaurus_manakini:12.3,Laiyangosaurus_youngi:1):1,Kamuysaurus_japonicus:12.3):1):1,(((Saurolophus_angustirostris:1,Saurolophus_osborni:0.08):1,Prosaurolophus_maximus:0.68):6.62,Lophorhothon_atopus:1):1.4):1,((((Gryposaurus_latidens:1,Rhinorex_condrupus:5.12):1,(Gryposaurus_monumentensis:0.7,Gryposaurus_notabilis:1):4.7):1,Secernosaurus_koerneri:4.62):1,Kritosaurus_navajovius:4.8):3.4):1,(((Brachylophosaurus_canadensis:1,Probrachylophosaurus_bergei:1):1,Maiasaura_peeblesorum:1.3):2.1,(Acristavus_gagslarsoni:1,Wulagasaurus_dongi:10.69):1):3.8):1,Hadrosaurus_foulkii:0.8):1.9,Lambeosaurinae:1);

## Supplementary Data S6. Tree file for DEC analysis (minimum branch lengths)

((((((((Edmontosaurus_annectens:5.5,Edmontosaurus_regalis:0.1):8.5,Shantungosaurus_giganteus:0.1):3.6,((Kerberosaurus_manakini:12.4,Laiyangosaurus_youngi:0.1):0.1,Kamuysaurus_japonicus:12.5):0.1):0.1,(((Saurolophus_angustirostris:0.1,Saurolophus_osborni:0.18):0.1,Prosaurolophus_maximus:0.88):6.52,Lophorhothon_atopus:0.1):1.7):0.1,((((Gryposaurus_latidens:0.1,Rhinorex_condrupus:5.22):0.1,(Gryposaurus_monumentensis:0.8,Gryposaurus_notabilis:0.1):4.8):0.1,Secernosaurus_koerneri:4.92):0.1,Kritosaurus_navajovius:5.2):3.5):0.1,(((Brachylophosaurus_canadensis:0.1,Probrachylophosaurus_bergei:0.1):0.1,Maiasaura_peeblesorum:1.5):2.1,(Acristavus_gagslarsoni:0.1,Wulagasaurus_dongi:10.79):0.1):4.2):0.1,Hadrosaurus_foulkii:1.5):1.3,Lambeosaurinae:0.1);

Supplementary Data S7. Tree file for DEC analysis (equal)

((((((((Edmontosaurus_annectens:9.65,Edmontosaurus_regalis:4.25):4.916666667,Shantungosaurus_giganteus:0.6666666667):0.6666666667,((Kerberosaurus_manakini:3.9,Laiyangosaurus_youngi:2.5):2.5,Kamuysaurus_japonicus:6.4):3.833333333):0.9066666667,(((Saurolophus_angustirostris:2.206666667,Saurolophus_osborni:2.286666667):2.206666667,Prosaurolophus_maximus:5.093333333):2.326666667,Lophorhothon_atopus:0.12):0.12):0.12,((((Gryposaurus_latidens:0.4,Rhinorex_condrupus:5.52):0.4,(Gryposaurus_monumentensis:3.45,Gryposaurus_notabilis:2.75):2.75):0.4,Secernosaurus_koerneri:5.82):0.4,Kritosaurus_navajovius:6.4):0.76):0.12,(((Brachylophosaurus_canadensis:0.7,Probrachylophosaurus_bergei:0.7):0.7,Maiasaura_peeblesorum:2.7):2.62,(Acristavus_gagslarsoni:0.96,Wulagasaurus_dongi:11.65):0.96):0.96):1.52,Hadrosaurus_foulkii:1.4):1.4,Ouranosaurus_nigeriensis:0.1);

Supplementary Data S8. Tree file for DEC analysis (basic)

((((((((Edmontosaurus_annectens:5.4,Edmontosaurus_regalis:0):8.5,Shantungosaurus_giganteus:0):0,((Kerberosaurus_manakini:1.4,Laiyangosaurus_youngi:0):0,Kamuysaurus_japonicus:1.4):7.5):2,(((Saurolophus_angustirostris:0,Saurolophus_osborni:0.08):0,Prosaurolophus_maximus:0.68):6.62,Lophorhothon_atopus:0):0):0,((((Gryposaurus_latidens:0,Rhinorex_condrupus:5.12):0,(Gryposaurus_monumentensis:0.7,Gryposaurus_notabilis:0):4.7):0,Secernosaurus_koerneri:4.62):0,Kritosaurus_navajovius:4.8):2):0,(((Brachylophosaurus_canadensis:0,Probrachylophosaurus_bergei:0):0,Maiasaura_peeblesorum:1.3):2.1,(Acristavus_gagslarsoni:0,Wulagasaurus_dongi:10.69):0):2.4):0.6,Hadrosaurus_foulkii:0):2.7,Ouranosaurus_nigeriensis:0);

## Supplementary Data S9. Distribution file for DEC analysis. A represents Asia, B represents North America, C represents South America, and D represents Europe.

| 1 | *Edmontosaurus annectens* | B |
| --- | --- | --- |
| 2 | *Edmontosaurus regalis* | B |
| 3 | *Shantungosaurus giganteus* | A |
| 4 | *Kerberosaurus manakini* | A |
| 5 | *Laiyangosaurus youngi* | A |
| 6 | *Kamuysaurus japonicus* | A |
| 7 | *Saurolophus angustirostris* | A |
| 8 | *Saurolophus osborni* | B |
| 9 | *Prosaurolophus maximus* | B |
| 10 | *Lophorhothon atopus* | B |
| 11 | *Gryposaurus latidens* | B |
| 12 | *Rhinorex condrupus* | B |
| 13 | *Gryposaurus monumentensis* | B |
| 14 | *Gryposaurus notabilis* | B |
| 15 | *Secernosaurus koerneri* | C |
| 16 | *Kritosaurus navajovius* | B |
| 17 | *Brachylophosaurus canadensis* | B |
| 18 | *Probrachylophosaurus bergei* | B |
| 19 | *Maiasaura peeblesorum* | B |
| 20 | *Acristavus gagslarsoni* | B |
| 21 | *Wulagasaurus dongi* | A |
| 22 | *Hadrosaurus foulkii* | B |
| 23 | Lambeosaurinae | ABD |

## Supplementary Data S10. Probability matrix for DEC analysis

|  | Asia | North America | South America | Europe |
| --- | --- | --- | --- | --- |
| Asia | - | 1 | 0 | 1 |
| North America | 1 | - | 1 | 1 |
| South America | 0 | 1 | - | 0 |
| Europe | 1 | 1 | 0 | - |

## Supplementary Data S11. Tree file for ASR analysis (additive)

((((((((((((((((((((((((((((Corythosaurus_casuarius:0.1,Corythosaurus_intermedius:0.1):0.1,(Hypacrosaurus_altispinus:5.2,Hypacrosaurus_stebingeri:0.1):1.03):0.1,Velafrons_coahuilensis:0.83):0.1,Magnapaulia_laticauda:3.53):0.1,((Amurosaurus_riabinini:0.1,Sahaliyania_elunchunorum:0.29):6.6,(Lambeosaurus_lambei:0.1,Lambeosaurus_magnicristatus:0.5):0.1):0.53):0.1,Olorotitan_arharensi:5.03):0.1,((Parasaurolophus_walkeri:0.1,Parasaurolophus_cyrtocristatus:1.4):0,(Charonosaurus_jiayinensis:3.79,Parasaurolophus_tubicen:0.1):3.2):0.43):0,Arenysaurus_ardevoli:9.43):9.37,Jaxartosaurus_aralensis:0.1):0.1,(Pararhabdodon_isonense:12.4,Tsintaosaurus_spinorhinus:0.1):2):0.1,((Canardia_garonnensis:15.19,Nipponosaurus_sahalinensis:0.1):0,Blasisaurus_canudoi:17.4):1.4):0.1,Aralosaurus_tuberiferus:0.1):0.1,(((((((Edmontosaurus_annectens:5.5,Edmontosaurus_regalis:0.1):8.6,Shantungosaurus_giganteus:0.1):3.5,((Kerberosaurus_manakini:12.4,Laiyangosaurus_youngi:0.1):0.1,Kamuysaurus_japonicus:12.4):0.1):0.1,(((Saurolophus_angustirostris:0.1,Saurolophus_osborni:0.18):0.1,Prosaurolophus_maximus:0.78):6.72,Lophorhothon_atopus:0.1):1.5):0.1,((((Gryposaurus_latidens:0.1,Rhinorex_condrupus:5.22):0.1,(Gryposaurus_monumentensis:0.8,Gryposaurus_notabilis:0.1):4.8):0.1,Secernosaurus_koerneri:4.72):0.1,Kritosaurus_navajovius:4.9):3.5):0.1,((((Brachylophosaurus_canadensis:0.1,Probrachylophosaurus_bergei:0.1):0.1,Maiasaura_peeblesorum:1.4):2.2,Wulagasaurus_dongi:10.79):0,Acristavus_gagslarsoni:0.1):3.9):0.1,Hadrosaurus_foulkii:0.9):2):14.3,Plesiohadros_djadokhtaensis:25.6):0,((((Telmatosaurus_transsylvanicus:4.3,Tethyshadros_insularis:0.1):24.3,Adynomosaurus_arcanus:30.5):0,Eotrachodon_orientalis:16.6):0,((Nanningosaurus_dashiensis:0.1,Nanyangosaurus_zhugeii:8.75):0,(Zhanghenglong_yangchengensis:15.66,Claosaurus_agilis:12.74):0):0):0):0.1,Gilmoreosaurus_mongliensis:24.3):0.1,Bactrosaurus_johnsoni:24.3):0.1,Tanius_sinensis:27.6):0.1,Levnesovia_transoxiana:7.7):12.6,Jintasaurus_meniscus:0.1):0,(Yunganglong_datongensis:12.6,Eolambia_caroljonesa:18.1):0):16.5,(Probactrosaurus_gobiensis:0.1,Zuoyunlong_huangi:29):0.1):0.1,Xuwulong_yueluni:4.5):0.1,Sirindhorna_khoratensis:4.5):0.1,Equijubus_normani:16.5):0.1,Jinzhousaurus_yangi:10.5):0.1,(Iguanodon_bernissartensis:2.3,Mantellisaurus_atherfieldensis:0.1):0.1):0.1,Ouranosaurus_nigeriensis:0.1);

## Supplementary Data S12. Tree file for ASR analysis (zero-branch lengths additive)

(((((((((((((((((((((((((((((Corythosaurus_casuarius:0.1,Corythosaurus_intermedius:0.1):0.1,(Hypacrosaurus_altispinus:5.1,Hypacrosaurus_stebingeri:0.1):0.93):0.1,Velafrons_coahuilensis:0.73):0.1,Magnapaulia_laticauda:3.43):0.1,((Amurosaurus_riabinini:0.1,Sahaliyania_elunchunorum:0.19):6.5,(Lambeosaurus_lambei:0.1,Lambeosaurus_magnicristatus:0.4):0.1):0.43):0.1,Olorotitan_arharensi:4.93):0.1,((((Charonosaurus_jiayinensis:3.69,Parasaurolophus_tubicen:0.1):3.1,Parasaurolophus_cyrtocristatus:1.3):0,Parasaurolophus_walkeri:0.1):0.33,Arenysaurus_ardevoli:9.33):0):9.27,Jaxartosaurus_aralensis:0.1):0.1,(Pararhabdodon_isonense:12.3,Tsintaosaurus_spinorhinus:0.1):1.9):0.1,((Canardia_garonnensis:15.09,Blasisaurus_canudoi:17.3):0,Nipponosaurus_sahalinensis:0.1):1.3):0.1,Aralosaurus_tuberiferus:0.1):0.1,(((((((Edmontosaurus_annectens:5.4,Edmontosaurus_regalis:0.1):8.5,Shantungosaurus_giganteus:0.1):3.4,((Kerberosaurus_manakini:12.3,Laiyangosaurus_youngi:0.1):0.1,Kamuysaurus_japonicus:12.3):0.1):0.1,(((Saurolophus_angustirostris:0.1,Saurolophus_osborni:0.08):0.1,Prosaurolophus_maximus:0.68):6.62,Lophorhothon_atopus:0.1):1.4):0.1,((((Gryposaurus_latidens:0.1,Rhinorex_condrupus:5.12):0.1,(Gryposaurus_monumentensis:0.7,Gryposaurus_notabilis:0.1):4.7):0.1,Secernosaurus_koerneri:4.62):0.1,Kritosaurus_navajovius:4.8):3.4):0.1,((((Brachylophosaurus_canadensis:0.1,Probrachylophosaurus_bergei:0.1):0.1,Maiasaura_peeblesorum:1.3):2.1,Acristavus_gagslarsoni:0.1):0,Wulagasaurus_dongi:10.69):3.8):0.1,Hadrosaurus_foulkii:0.8):1.9):14.2,(Nanningosaurus_dashiensis:0.1,Eotrachodon_orientalis:16.5):0):0,(Telmatosaurus_transsylvanicus:4.2,Tethyshadros_insularis:0.1):24.2):0,((Nanyangosaurus_zhugeii:8.65,Adynomosaurus_arcanus:30.4):0,Plesiohadros_djadokhtaensis:25.5):0):0,(Zhanghenglong_yangchengensis:15.56,Claosaurus_agilis:12.64):0):0.1,Gilmoreosaurus_mongliensis:24.2):0.1,Bactrosaurus_johnsoni:24.2):0.1,Tanius_sinensis:27.5):0.1,Levnesovia_transoxiana:7.6):12.5,(Jintasaurus_meniscus:0.1,Eolambia_caroljonesa:18):0):0,Yunganglong_datongensis:12.5):16.4,(Probactrosaurus_gobiensis:0.1,Zuoyunlong_huangi:28.9):0.1):0.1,Xuwulong_yueluni:4.4):0.1,Sirindhorna_khoratensis:4.4):0.1,Equijubus_normani:16.4):0.1,Jinzhousaurus_yangi:10.4):0.1,(Iguanodon_bernissartensis:2.2,Mantellisaurus_atherfieldensis:0.1):0.1):0.1,Ouranosaurus_nigeriensis:0.1);

## Supplementary Data S13. Tree file for ASR analysis (minimum branch lengths)

(((((((((((((((((((((((((((Corythosaurus_casuarius:0.1,Corythosaurus_intermedius:0.1):0.1,(Hypacrosaurus_altispinus:5.2,Hypacrosaurus_stebingeri:0.1):1.03):0.1,Velafrons_coahuilensis:1.03):0.1,Magnapaulia_laticauda:3.83):0.1,((Amurosaurus_riabinini:0.1,Sahaliyania_elunchunorum:0.29):6.6,(Lambeosaurus_lambei:0.1,Lambeosaurus_magnicristatus:0.5):0.1):0.73):0.1,Olorotitan_arharensi:5.53):0.1,((((Charonosaurus_jiayinensis:3.79,Parasaurolophus_tubicen:0.1):3.1,Parasaurolophus_walkeri:0.1):0,Parasaurolophus_cyrtocristatus:1.4):0.93,Arenysaurus_ardevoli:10.03):0):8.67,Jaxartosaurus_aralensis:0.1):0.1,(Pararhabdodon_isonense:12.4,Tsintaosaurus_spinorhinus:0.1):2):0.1,((Canardia_garonnensis:15.19,Nipponosaurus_sahalinensis:0.1):0,Blasisaurus_canudoi:17.4):1.5):0.1,Aralosaurus_tuberiferus:0.4):0.1,(((((((Edmontosaurus_annectens:5.5,Edmontosaurus_regalis:0.1):8.5,Shantungosaurus_giganteus:0.1):3.6,((Kerberosaurus_manakini:12.4,Laiyangosaurus_youngi:0.1):0.1,Kamuysaurus_japonicus:12.5):0.1):0.1,(((Saurolophus_angustirostris:0.1,Saurolophus_osborni:0.18):0.1,Prosaurolophus_maximus:0.88):6.52,Lophorhothon_atopus:0.1):1.7):0.1,((((Gryposaurus_latidens:0.1,Rhinorex_condrupus:5.22):0.1,(Gryposaurus_monumentensis:0.8,Gryposaurus_notabilis:0.1):4.8):0.1,Secernosaurus_koerneri:4.92):0.1,Kritosaurus_navajovius:5.2):3.5):0.1,(((Brachylophosaurus_canadensis:0.1,Probrachylophosaurus_bergei:0.1):0.1,Maiasaura_peeblesorum:1.5):2,(Wulagasaurus_dongi:10.79,Acristavus_gagslarsoni:0.1):0):4.3):0.1,Hadrosaurus_foulkii:1.5):1.7):13.8,Claosaurus_agilis:12.74):0,((((((Telmatosaurus_transsylvanicus:4.3,Tethyshadros_insularis:0.1):24.2,Nanyangosaurus_zhugeii:8.75):0,Zhanghenglong_yangchengensis:15.66):0,Eotrachodon_orientalis:16.6):0,Nanningosaurus_dashiensis:0.1):0,(Plesiohadros_djadokhtaensis:25.6,Adynomosaurus_arcanus:30.5):0):0):0.1,Gilmoreosaurus_mongliensis:24.4):0.1,Bactrosaurus_johnsoni:24.5):0.1,Tanius_sinensis:27.9):0.1,Levnesovia_transoxiana:8.1):12.1,Eolambia_caroljonesa:18.1):0,(Jintasaurus_meniscus:0.1,Yunganglong_datongensis:12.6):0):16.5,(Probactrosaurus_gobiensis:0.1,Zuoyunlong_huangi:29):0.1):0.1,Xuwulong_yueluni:4.7):0.1,Sirindhorna_khoratensis:4.8):0.1,Equijubus_normani:16.9):0.1,Jinzhousaurus_yangi:11):0.1,(Iguanodon_bernissartensis:2.3,Mantellisaurus_atherfieldensis:0.1):0.6):0.1,Ouranosaurus_nigeriensis:0.8);

Supplementary Data S14. Tree file for ASR analysis (basic)

((((((((((((((((((((((((((((Corythosaurus_casuarius:0,Corythosaurus_intermedius:0):0,(Hypacrosaurus_altispinus:5.1,Hypacrosaurus_stebingeri:0):0.93):0,Velafrons_coahuilensis:0.73):0,Magnapaulia_laticauda:3.43):0,((Amurosaurus_riabinini:0,Sahaliyania_elunchunorum:0.19):6.5,(Lambeosaurus_lambei:0,Lambeosaurus_magnicristatus:0.4):0):0.43):0,Olorotitan_arharensi:4.93):0,Arenysaurus_ardevoli:9.33):0,((Parasaurolophus_walkeri:0,Parasaurolophus_cyrtocristatus:1.3):0,(Charonosaurus_jiayinensis:3.69,Parasaurolophus_tubicen:0):3.1):0.33):9.27,Jaxartosaurus_aralensis:0):0,(Pararhabdodon_isonense:12.3,Tsintaosaurus_spinorhinus:0):1.9):0,((Blasisaurus_canudoi:17.3,Canardia_garonnensis:15.09):0,Nipponosaurus_sahalinensis:0):1.3):0,Aralosaurus_tuberiferus:0):0,(((((((Edmontosaurus_annectens:5.4,Edmontosaurus_regalis:0):8.5,Shantungosaurus_giganteus:0):0,((Kerberosaurus_manakini:1.4,Laiyangosaurus_youngi:0):0,Kamuysaurus_japonicus:1.4):7.5):2,(((Saurolophus_angustirostris:0,Saurolophus_osborni:0.08):0,Prosaurolophus_maximus:0.68):6.62,Lophorhothon_atopus:0):0):0,((((Gryposaurus_latidens:0,Rhinorex_condrupus:5.12):0,(Gryposaurus_monumentensis:0.7,Gryposaurus_notabilis:0):4.7):0,Secernosaurus_koerneri:4.62):0,Kritosaurus_navajovius:4.8):2):0,((((Brachylophosaurus_canadensis:0,Probrachylophosaurus_bergei:0):0,Maiasaura_peeblesorum:1.3):2.1,Wulagasaurus_dongi:10.69):0,Acristavus_gagslarsoni:0):2.4):0.6,Hadrosaurus_foulkii:0):2.7):14.2,(Telmatosaurus_transsylvanicus:4.2,Tethyshadros_insularis:0):24.2):0,(((((Nanyangosaurus_zhugeii:8.65,Zhanghenglong_yangchengensis:15.56):0,Plesiohadros_djadokhtaensis:25.5):0,Claosaurus_agilis:12.64):0,(Adynomosaurus_arcanus:30.4,Eotrachodon_orientalis:16.5):0):0,Nanningosaurus_dashiensis:0):0):0,Gilmoreosaurus_mongliensis:24.2):0,Bactrosaurus_johnsoni:24.2):0,Tanius_sinensis:27.5):0,Levnesovia_transoxiana:7.6):12.5,Jintasaurus_meniscus:0):0,(Yunganglong_datongensis:12.5,Eolambia_caroljonesa:18):0):16.4,(Probactrosaurus_gobiensis:0,Zuoyunlong_huangi:28.9):0):0,Xuwulong_yueluni:4.4):0,Sirindhorna_khoratensis:4.4):0,Equijubus_normani:16.4):0,Jinzhousaurus_yangi:10.4):0,(Iguanodon_bernissartensis:2.2,Mantellisaurus_atherfieldensis:0):0):0,Ouranosaurus_nigeriensis:0);

Supplementary Data S15. Tree file for ASR analysis (equal)

((((((((((((((((((((((((((Corythosaurus_casuarius:1.15875,Corythosaurus_intermedius:1.15875):1.15875,(Hypacrosaurus_altispinus:6.72375,Hypacrosaurus_stebingeri:1.62375):1.62375):1.15875,Velafrons_coahuilensis:4.20625):1.15875,Magnapaulia_laticauda:8.065):1.15875,((Amurosaurus_riabinini:3.25,Sahaliyania_elunchunorum:3.44):7.399166667,(Lambeosaurus_lambei:2.074583333,Lambeosaurus_magnicristatus:2.474583333):2.074583333):2.074583333):1.15875,Olorotitan_arharensi:11.8825):1.15875,(((Parasaurolophus_cyrtocristatus:5.520625,Parasaurolophus_walkeri:4.220625):0,(Charonosaurus_jiayinensis:5.24,Parasaurolophus_tubicen:1.55):5.770625):4.220625,Arenysaurus_ardevoli:17.44125):0):3.525416667,Jaxartosaurus_aralensis:2.366666667):2.366666667,(Pararhabdodon_isonense:15.61666667,Tsintaosaurus_spinorhinus:3.316666667):3.316666667):2.366666667,((Nipponosaurus_sahalinensis:4.2,Blasisaurus_canudoi:21.5):0,Canardia_garonnensis:19.29):4.2):2.366666667,Aralosaurus_tuberiferus:9.466666667):2.366666667,(((((((Edmontosaurus_annectens:9.65,Edmontosaurus_regalis:4.25):4.916666667,Shantungosaurus_giganteus:0.6666666667):0.6666666667,((Kerberosaurus_manakini:3.9,Laiyangosaurus_youngi:2.5):2.5,Kamuysaurus_japonicus:6.4):3.833333333):0.9066666667,(((Saurolophus_angustirostris:2.206666667,Saurolophus_osborni:2.286666667):2.206666667,Prosaurolophus_maximus:5.093333333):2.326666667,Lophorhothon_atopus:0.12):0.12):0.12,((((Gryposaurus_latidens:0.4,Rhinorex_condrupus:5.52):0.4,(Gryposaurus_monumentensis:3.45,Gryposaurus_notabilis:2.75):2.75):0.4,Secernosaurus_koerneri:5.82):0.4,Kritosaurus_navajovius:6.4):0.76):0.12,(((Brachylophosaurus_canadensis:0.7,Probrachylophosaurus_bergei:0.7):0.7,Maiasaura_peeblesorum:2.7):2.14,(Wulagasaurus_dongi:12.13,Acristavus_gagslarsoni:1.44):0):1.44):7.386666667,Hadrosaurus_foulkii:7.266666667):7.266666667):4.45,((Nanyangosaurus_zhugeii:10.73333333,Plesiohadros_djadokhtaensis:27.58333333):0,Claosaurus_agilis:14.72333333):0):0,((((Telmatosaurus_transsylvanicus:16.3,Tethyshadros_insularis:12.1):14.18333333,Zhanghenglong_yangchengensis:17.64333333):0,(Eotrachodon_orientalis:18.58333333,Nanningosaurus_dashiensis:2.083333333):0):0,Adynomosaurus_arcanus:32.48333333):0):2.083333333,Gilmoreosaurus_mongliensis:28.36666667):2.083333333,Bactrosaurus_johnsoni:30.45):2.083333333,Tanius_sinensis:35.83333333):2.083333333,Levnesovia_transoxiana:18.01666667):10.28333333,((Yunganglong_datongensis:20.7,Jintasaurus_meniscus:8.2):0,Eolambia_caroljonesa:26.2):0):8.225,(Probactrosaurus_gobiensis:0.0125,Zuoyunlong_huangi:28.9125):0.0125):0.0125,Xuwulong_yueluni:4.4375):0.0125,Sirindhorna_khoratensis:4.45):0.0125,Equijubus_normani:16.4625):0.0125,Jinzhousaurus_yangi:10.475):0.0125,(Iguanodon_bernissartensis:2.24375,Mantellisaurus_atherfieldensis:0.04375):0.04375):0.0125,Ouranosaurus_nigeriensis:0.1);

## Supplementary Data S16. Habitat environments used in ASR analysis: M, marginal; I, inland.

|  | Env |
| --- | --- |
| *Acristavus gagslarsoni* | I |
| *Adynomosaurus arcanus* | I&M |
| *Amurosaurus riabinini* | I |
| *Aralosaurus tuberiferus* | M |
| *Arenysaurus ardevoli* | M |
| *Bactrosaurus johnsoni* | I |
| *Blasisaurus canudoi* | M |
| *Brachylophosaurus canadensis* | I&M |
| *Canardia garonnensis* | M |
| *Charonosaurus jiayinensis* | I |
| *Claosaurus agilis* | M |
| *Corythosaurus casuarius* | I |
| *Corythosaurus intermedius* | I |
| *Edmontosaurus annectens* | I&M |
| *Edmontosaurus regalis* | I |
| *Eolambia caroljonesa* | I |
| *Eotrachodon orientalis* | M |
| *Equijubus normani* | I |
| *Gilmoreosaurus mongliensis* | I |
| *Gryposaurus latidens* | I |
| *Gryposaurus monumentensis* | I |
| *Gryposaurus notabilis* | I |
| *Hadrosaurus foulkii* | M |
| *Kamuysaurus japonicus* | M |
| *Hypacrosaurus altispinus* | I&M |
| *Hypacrosaurus stebingeri* | I |
| *Iguanodon bernissartensis* | I |
| *Jaxartosaurus aralensis* | ? |
| *Jintasaurus meniscus* | I |
| *Jinzhousaurus yangi* | I |
| *Kerberosaurus manakini* | I |
| *Kritosaurus navajovius* | I&M |
| *Laiyangosaurus youngi* | I |
| *Lambeosaurus lambei* | I&M |
| *Lambeosaurus magnicristatus* | I&M |
| *Levnesovia transoxiana* | I |
| *Lophorhothon atopus* | M |
| *Magnapaulia laticauda* | ? |
| *Maiasaura peeblesorum* | I |
| *Mantellisaurus atherfieldensis* | I |
| *Nanningosaurus dashiensis* | ? |
| *Nanyangosaurus zhugeii* | I |
| *Nipponosaurus sahalinensis* | M |
| *Olorotitan arharensi* | I |
| *Ouranosaurus nigeriensis* | I |
| *Pararhabdodon isonense* | I |
| *Parasaurolophus cyrtocristatus* | I&M |
| *Parasaurolophus tubicen* | I |
| *Parasaurolophus walkeri* | I |
| *Plesiohadros djadokhtaensis* | I |
| *Probactrosaurus gobiensis* | I |
| *Probrachylophosaurus bergei* | I |
| *Prosaurolophus maximus* | I&M |
| *Rhinorex condrupus* | I&M |
| *Sahaliyania elunchunorum* | I |
| *Saurolophus angustirostris* | I |
| *Saurolophus osborni* | I&M |
| *Secernosaurus koerneri* | I |
| *Shantungosaurus giganteus* | I |
| *Sirindhorna khoratensis* | I&M |
| *Tanius sinensis* | I |
| *Telmatosaurus transsylvanicus* | I |
| *Tethyshadros insularis* | M |
| *Tsintaosaurus spinorhinus* | I |
| *Velafrons coahuilensis* | M |
| *Wulagasaurus dongi* | I |
| *Xuwulong yueluni* | I |
| *Yunganglong datongensis* | ? |
| *Zhanghenglong yangchengensis* | I |
| *Zuoyunlong huangi* | ? |

# Supplementary Figures


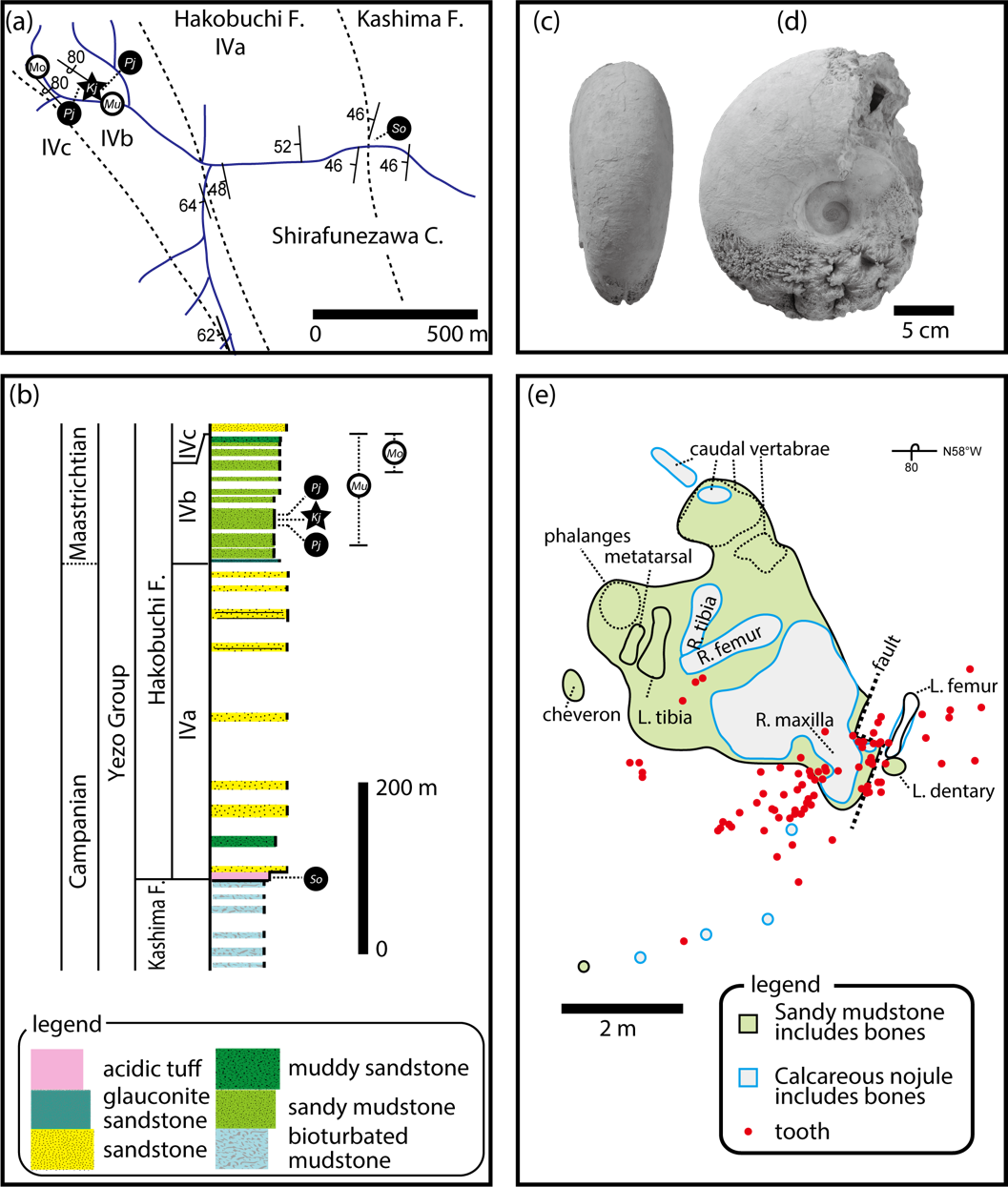


Supplementary Figure S1. Simplified geological map showing Shirafunezawa Creek. Black star with *Kj* represents the locality of *Kamuysaurus japonicus* gen. et sp. nov. (HMG-1219) (a). White circle with *Mu* represents the locality of *Mesodermochelys undulates* (sea turtle) holotype (HMG-5) in a float. White circle with Mo shows the locality of a mosasaurine specimen (HMG-10). In situ occurrences of index species of ammonoids and inoceramid bivalves are shown as the following abbreviations. *So*: *Sphenoceramus orientalis* (upper lower Campanian inoceramid). *Pj*: *Pachydiscus* (*Neodesmoceras*) *japonicus* (lowest Maastrichtian ammonoid). Stratigraphic section of Shirafunezawa Creek and detail occurrences of molluscan and vertebrate fossils (b). Lowest Maastrichtian index ammonoid, *Pachydiscus* (*Neodesmoceras*) *japonicus* (HMG-1992) from 3 m above the horizon of *Kamuysaurus japonicus* gen. et sp. nov. (c and d). A schematic quarry map showing bones and teeth distribution of *Kamuysaurus japonicus* gen. et sp. nov. in down side view of the horizontal plane (e).


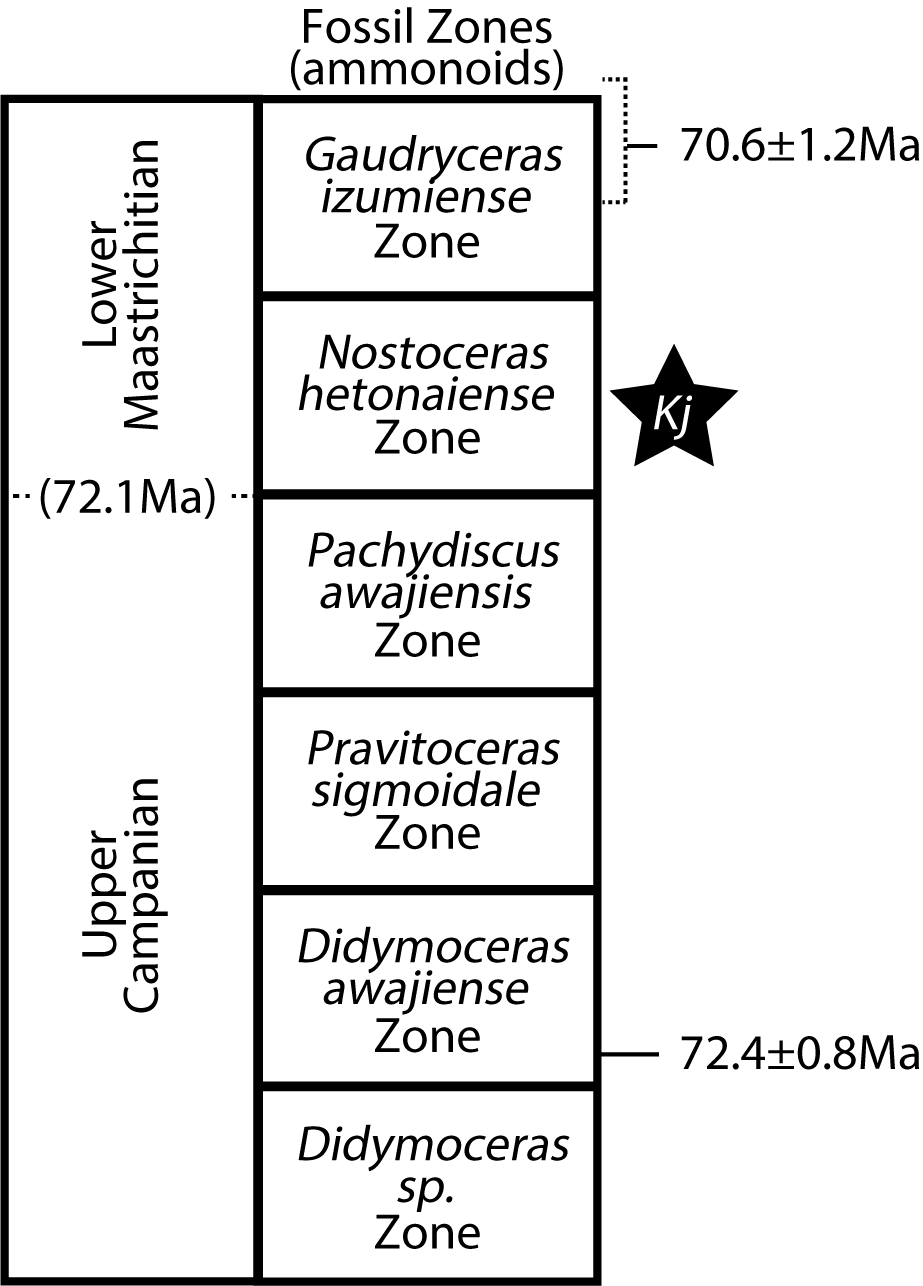


Supplementary Figure S2. Upper Campanian to lower Maastrichtian fossil zones in Japan. Index ammonoid zones based on southwest Japan^1^ and Hokkaido (lower Maastrichtian^2^). Two U–Pb ages were obtained from the Wakayama and Soya Hill areas^3,4^.


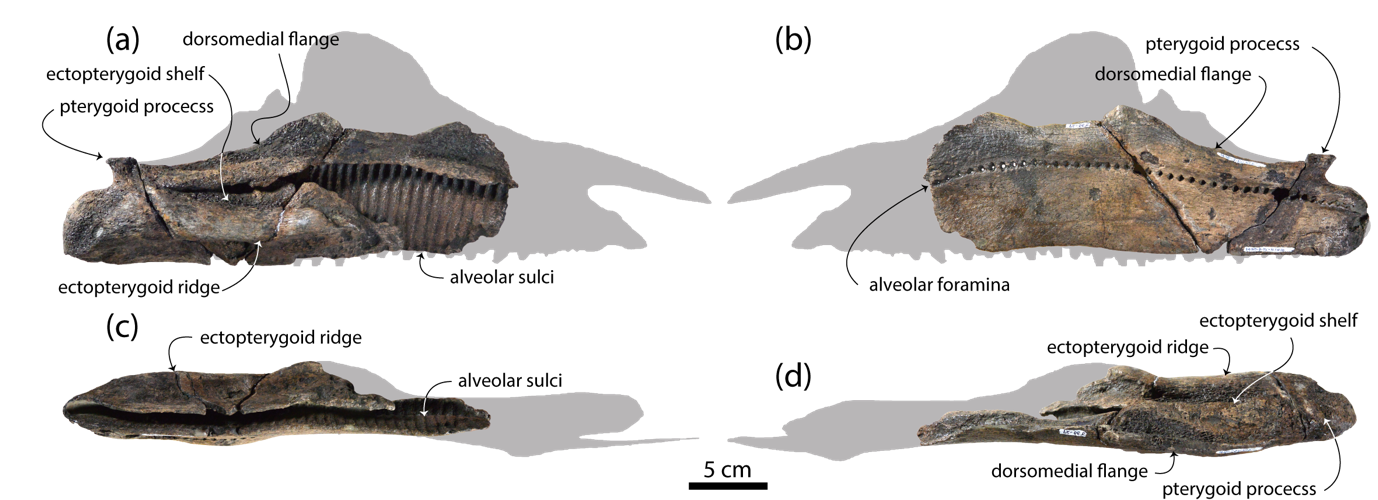


Supplementary Figure S3. Right maxilla in lateral (a), medial (b), ventral (c), and dorsal (d) views.


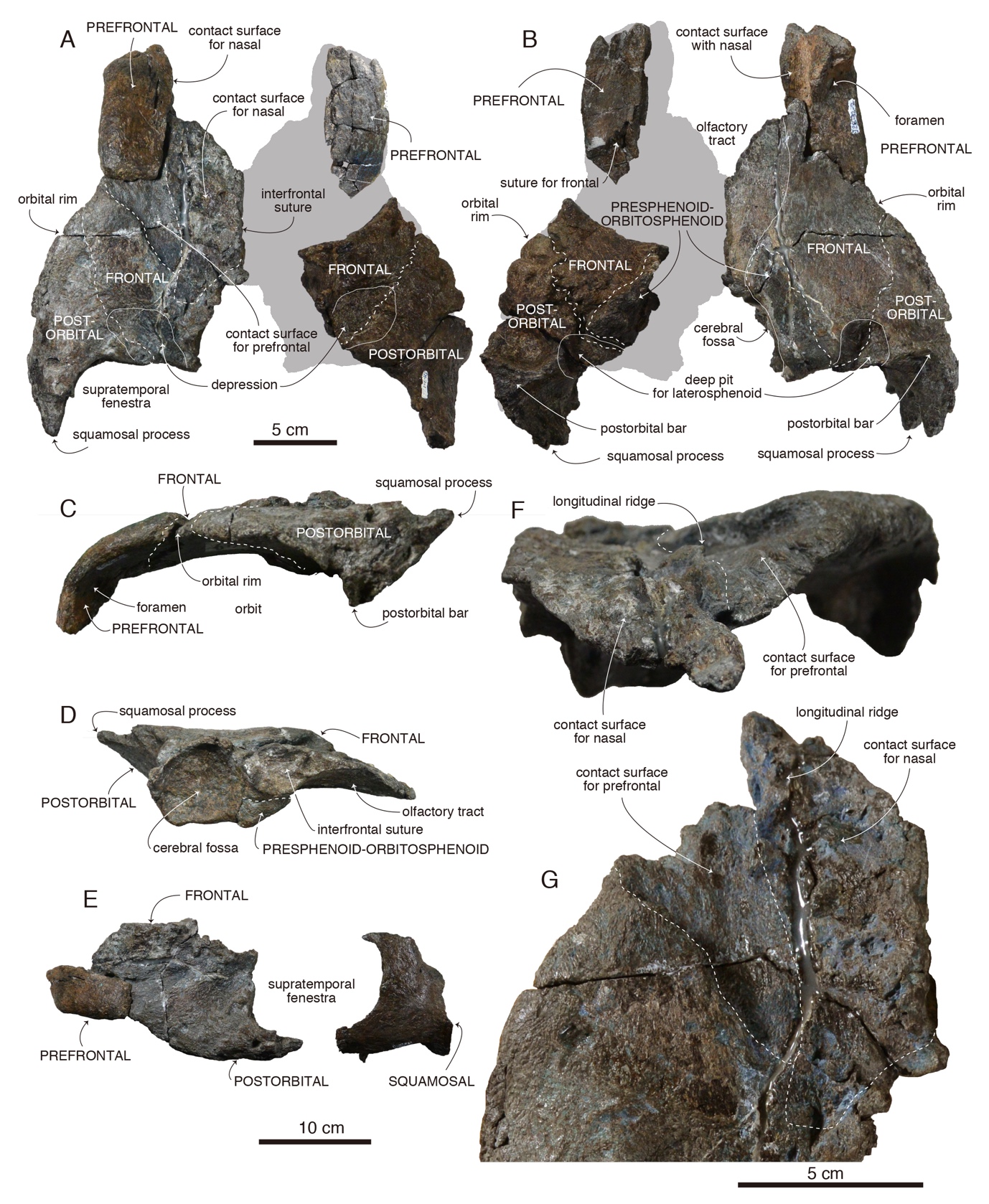


Supplementary Figure S4. Both prefrontals, frontals, postorbitals, and preshenoid-orbitosphenoids in dorsal (a) and ventral (b) views. Left prefrontal, frontal, and postorbital in lateral view (c). Left frontal, postorbital, and presphenoid-orbitosphenoid in medial view (d). Left prefrontal, frontal, postorbital, and squamosal in dorsal view, showing the shape of the supratemporal fenestra (e). Close-up photos of the frontal, showing contact surfaces for the nasal and prefrontal, in anterior (f) and dorsal view (g). Scale bar below (a) is for (a) to (d), scale bar below (e) is for (e), and scale bar below (g) is for (f) and (g).


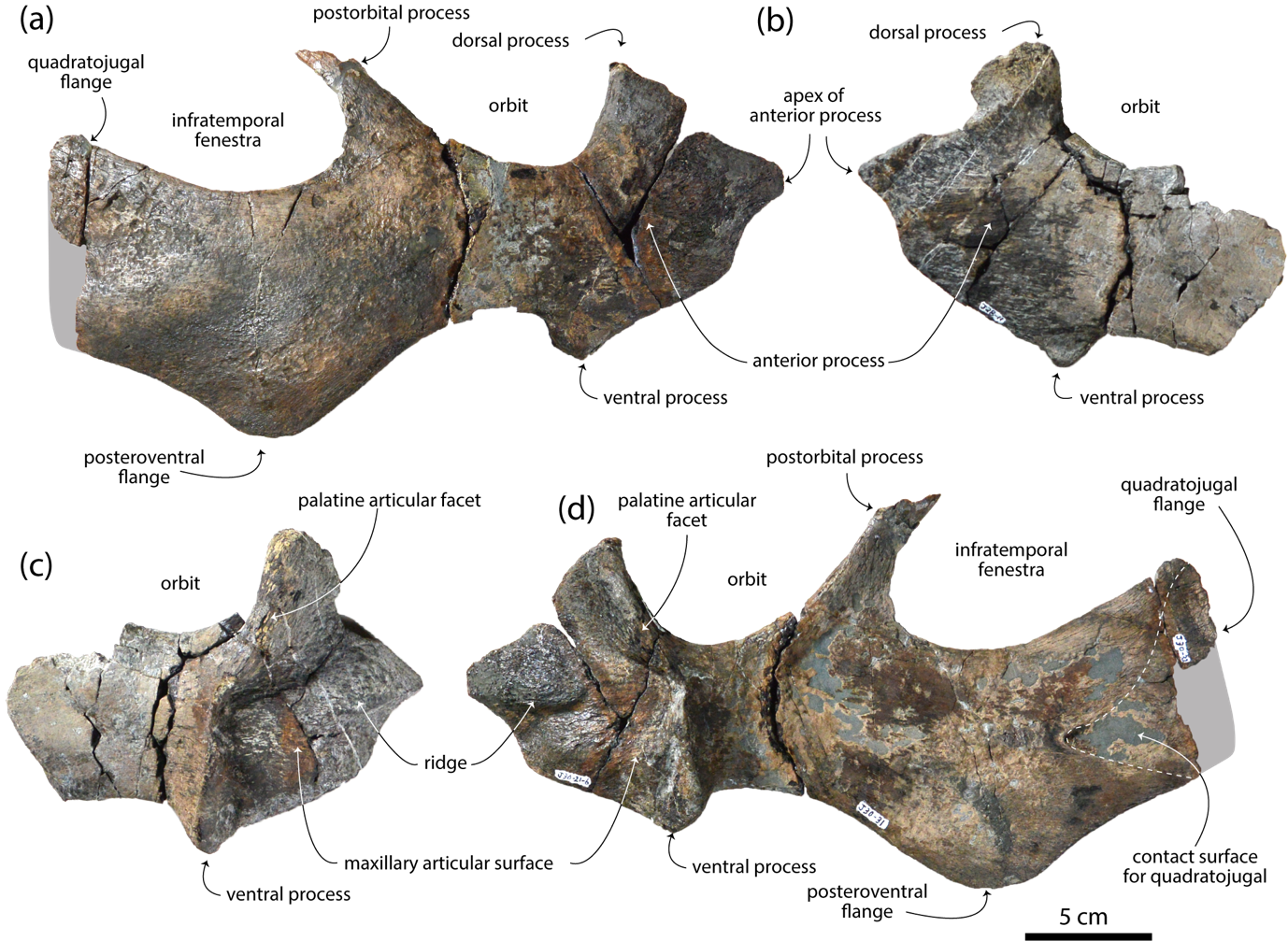


Supplementary Figure S5. Left jugal in lateral (a) and medial (d) views and right jugal in lateral (b) and medial (c) views.


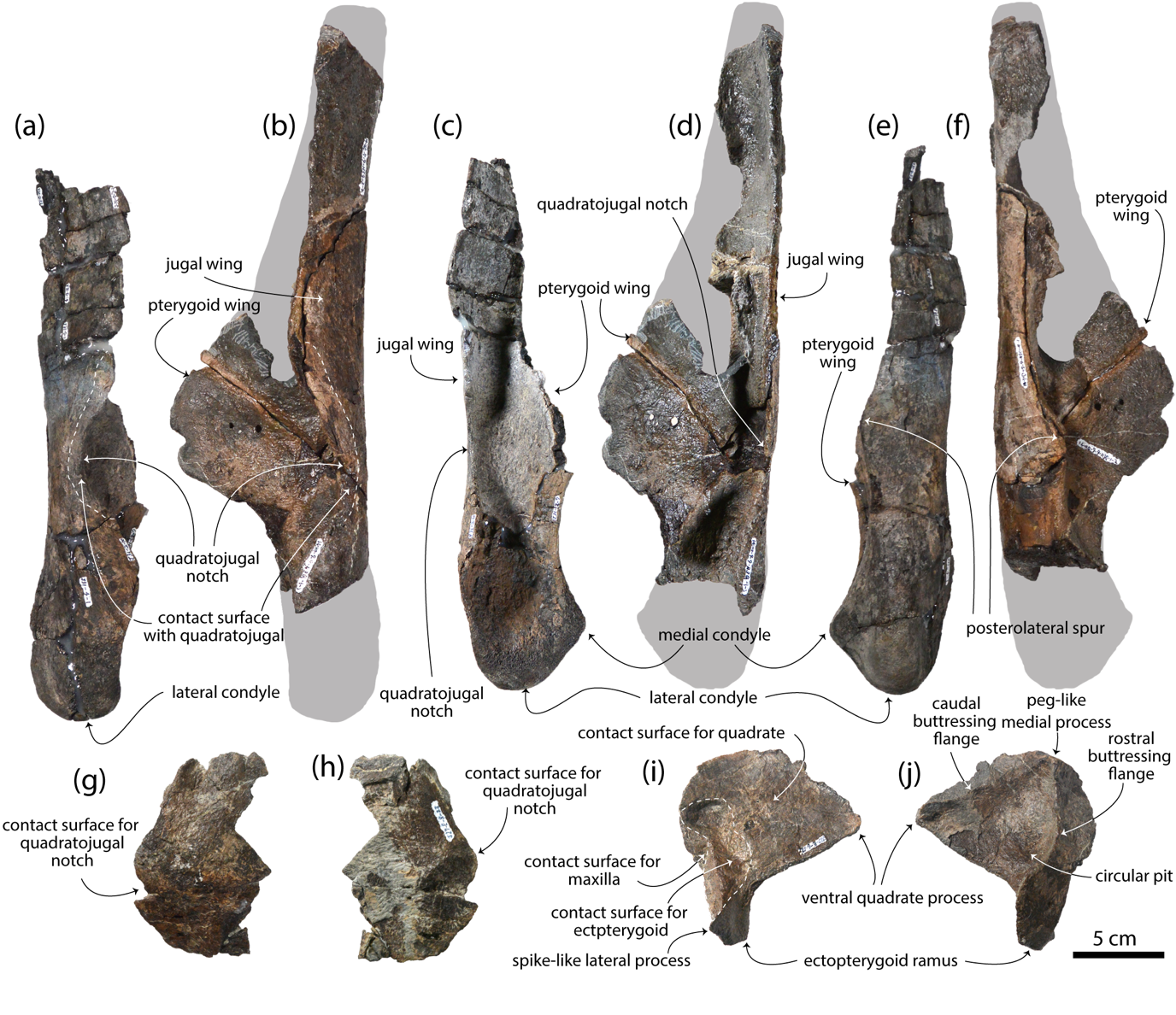


Supplementary Figure S6. Quadrates in lateral (a, right; b, left), anterior (c, right; d, left), and posterior (e, right; f, left) views. Right quadratojugal in lateral (g) and medial (h) views. Left pterygoid in lateral (i) and medial (j) views.


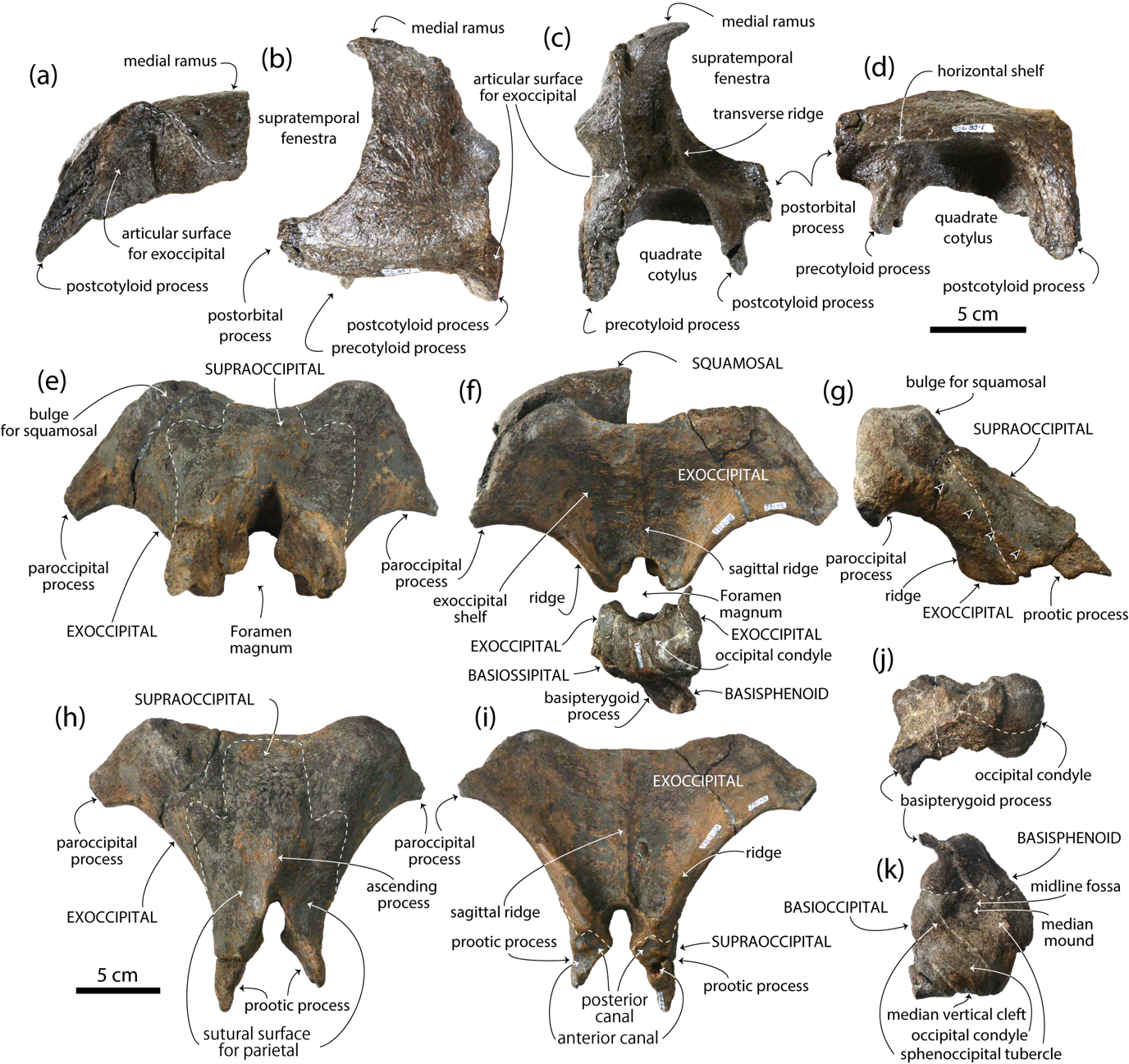


Supplementary Figure S7. Left squamosal in posterior (a), dorsal (b), ventral (c), and lateral (d) views. Exoccipital and supraoccipital in anterior (e), right lateral (g), anterodorsal (h), and posteroventral (i) views. Squamosal, exoccipital, and basioccipital in posterior view (f). Basioccipital in left lateral (j) and ventral (k) views. The arrowheads in (g) represents the posteroventral margin for prootic contact surface.


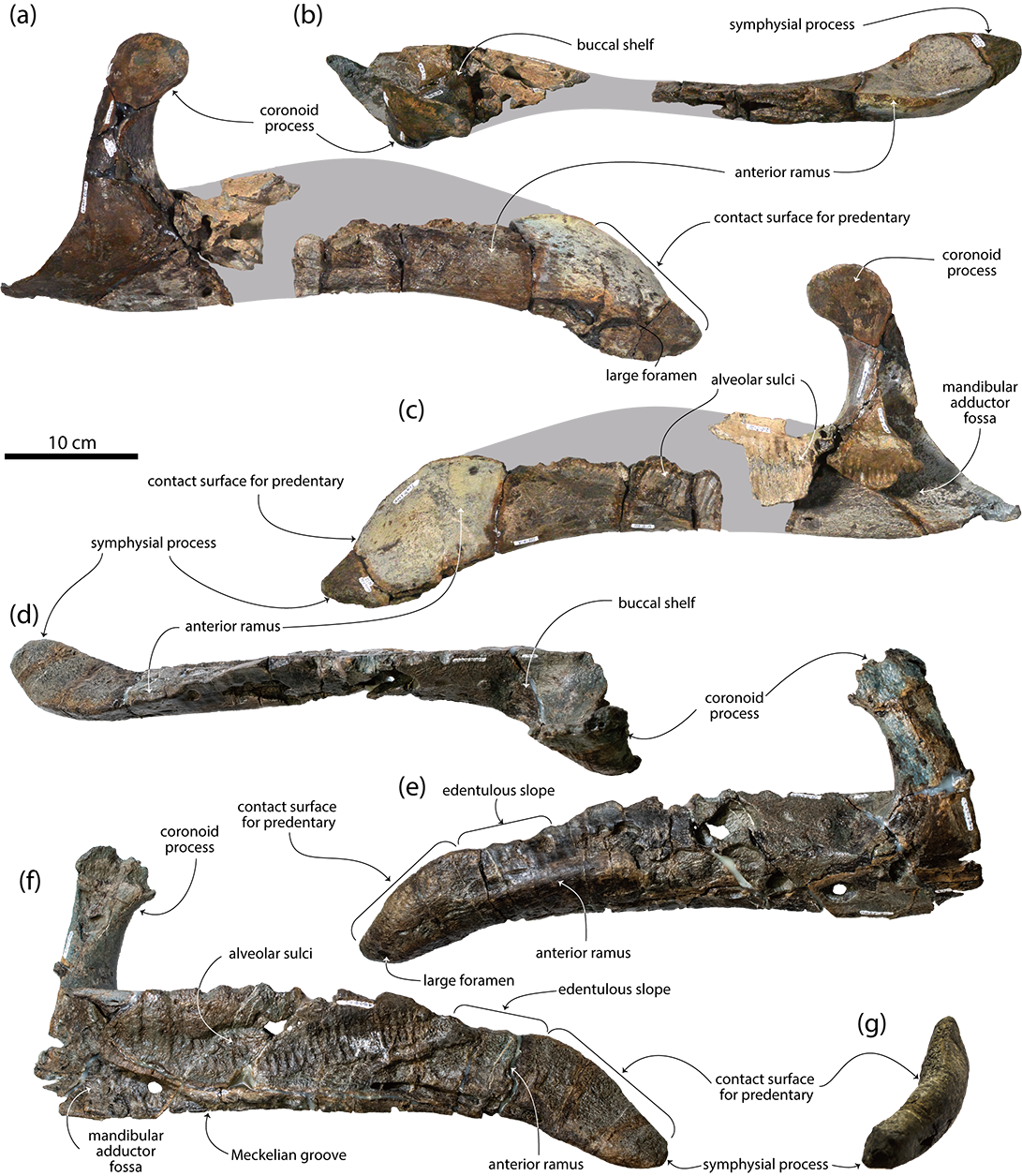


Supplementary Figure S8. Right dentary in lateral (a), dorsal (b), and medial (c) views. Left dentary in dorsal (d), lateral (e), medial (f), and anterior (g) views.


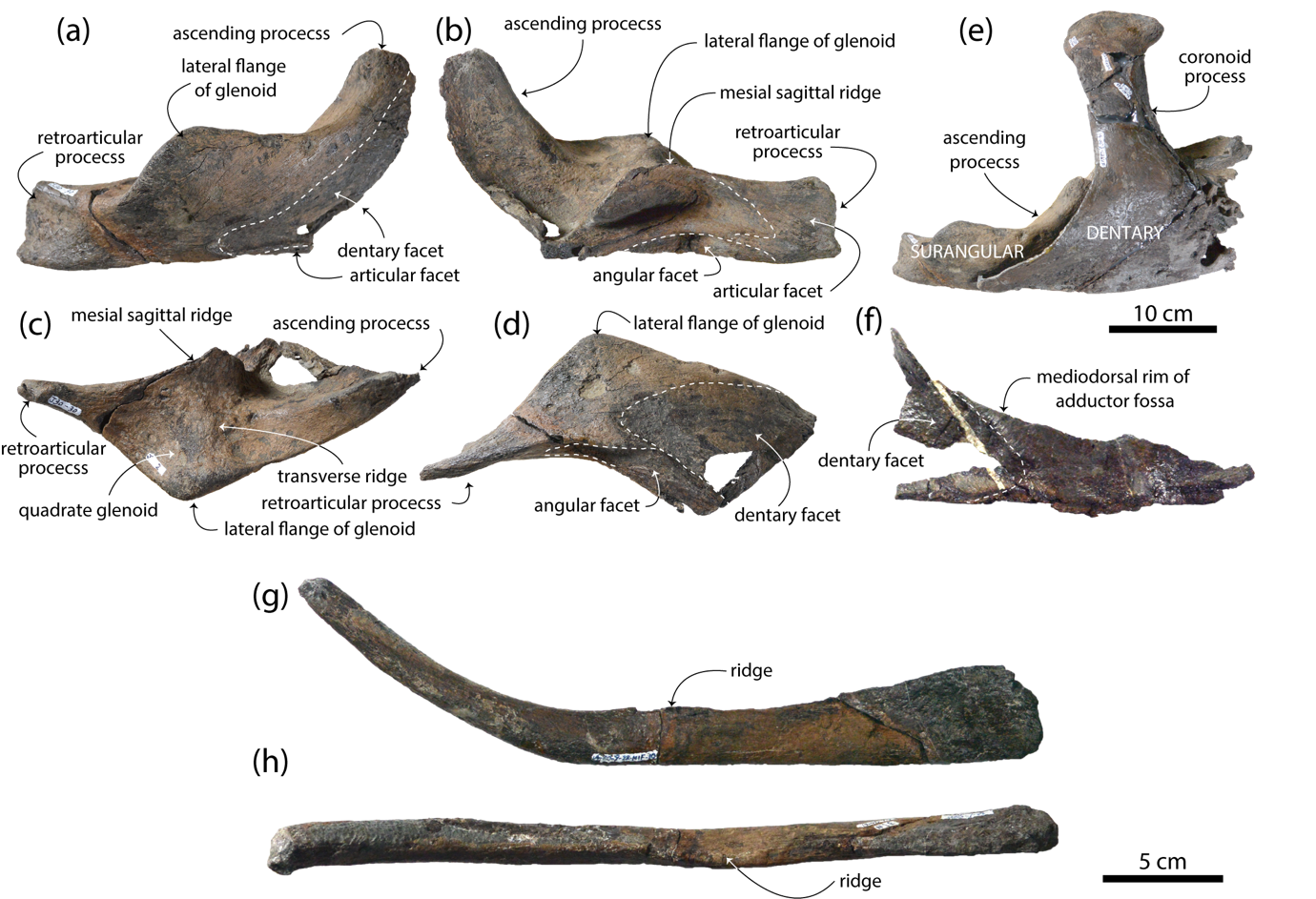


Supplementary Figure S9. Right surangular in lateral (a), dorsal (b), ventral (c), and medial (d) views. Posterior portion of the right dentary and surangular (e). Left splenial in lateral view (f). Right ceratobranchial in right lateral (g) and dorsal (h) views.


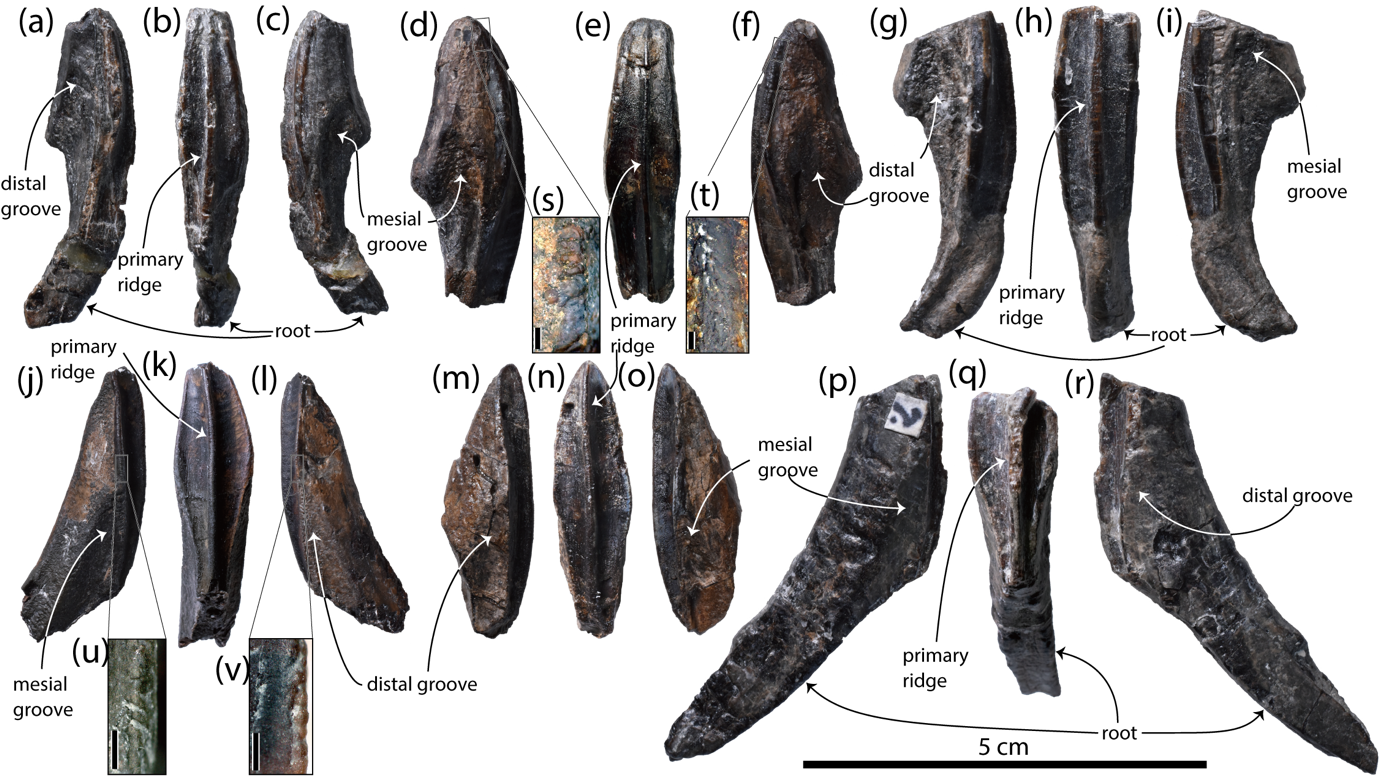


Supplementary Figure S10. Maxillary teeth (a-i) in and dentary teeth (j-r) in labial (b, e, and h), lingual (m, l, and q), mesial (c, d, i, j, o, and p), and distal (a, f, g, n, k, and r) views. Marginal denticles of dentary teeth (s and t). Scale for (s) to (v) = 0.5 mm.


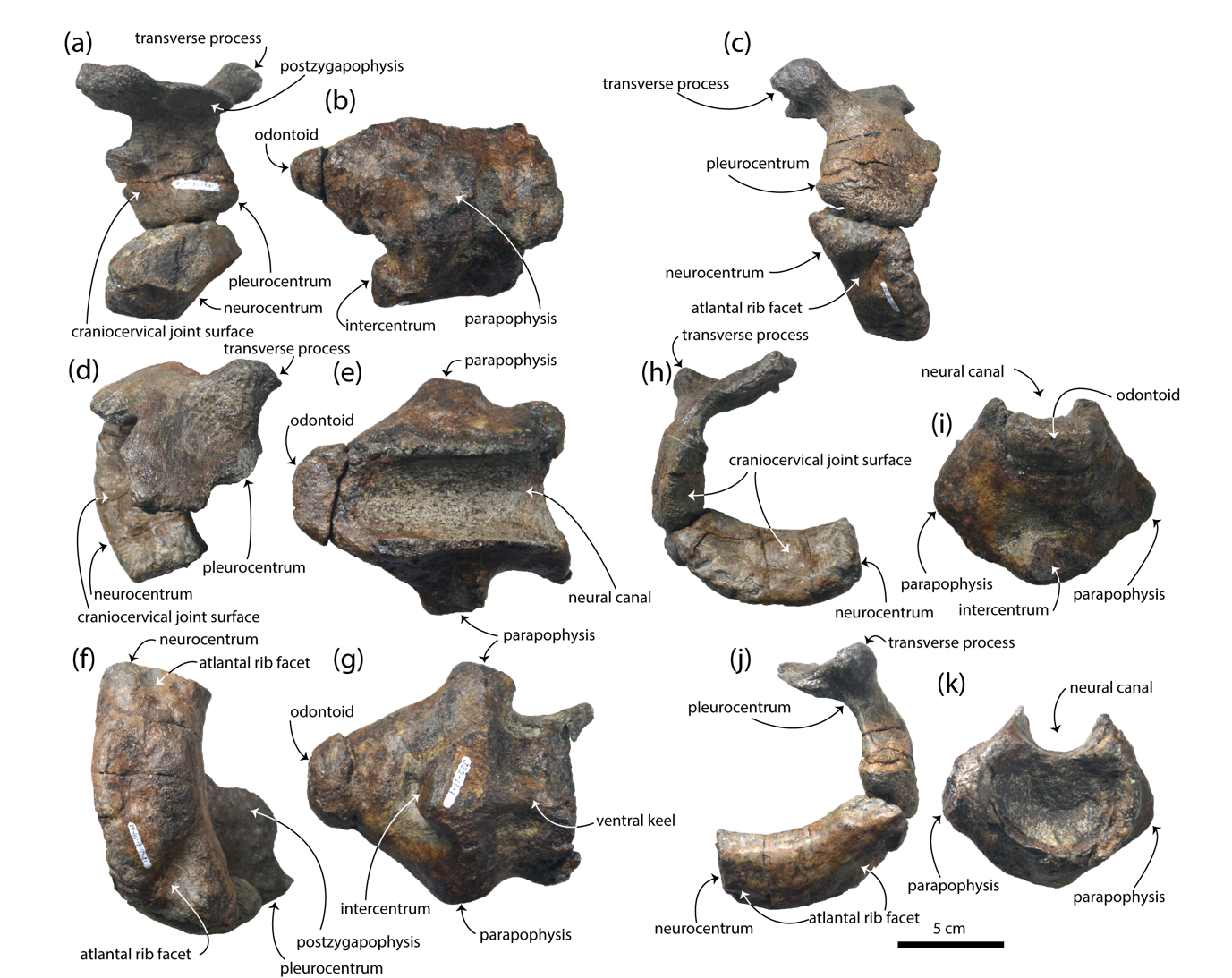


Supplementary Figure S11. Atlas in left lateral (a), right lateral (c), dorsal (d), ventral (f), anterior (h), and posterior (j) views. Axis in left lateral (b), dorsal (e), ventral (g), anterior (i), and posterior (k) views.


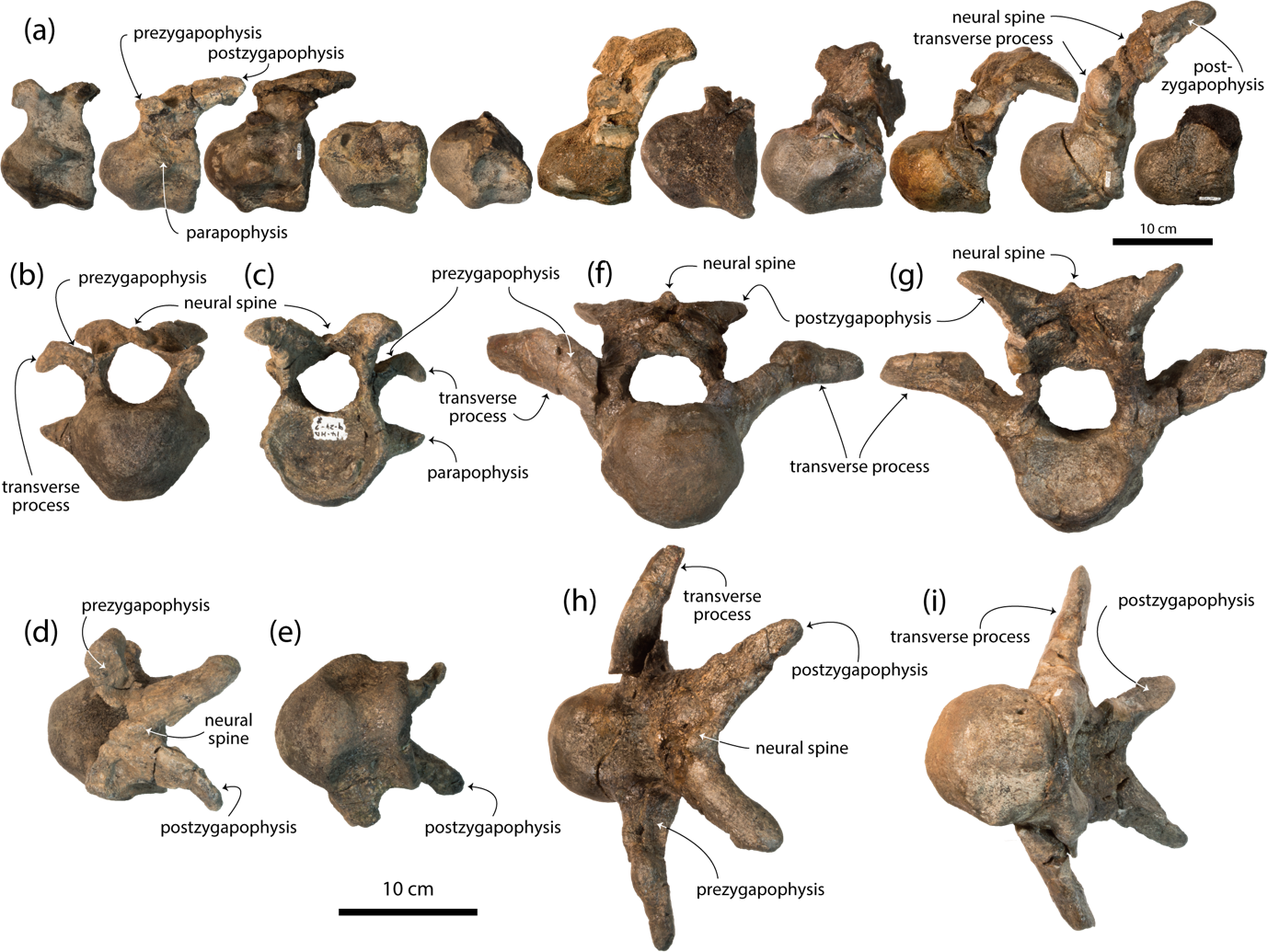


Supplementary Figure S12. Cervical vertebrae (third to thirteenth cervicals) in lateral view (a). Fourth cervical in anterior (b), posterior (c), dorsal (d), and ventral (e) views. Twelfth cervical in anterior (f), posterior (g), dorsal (h), and ventral (i) views.


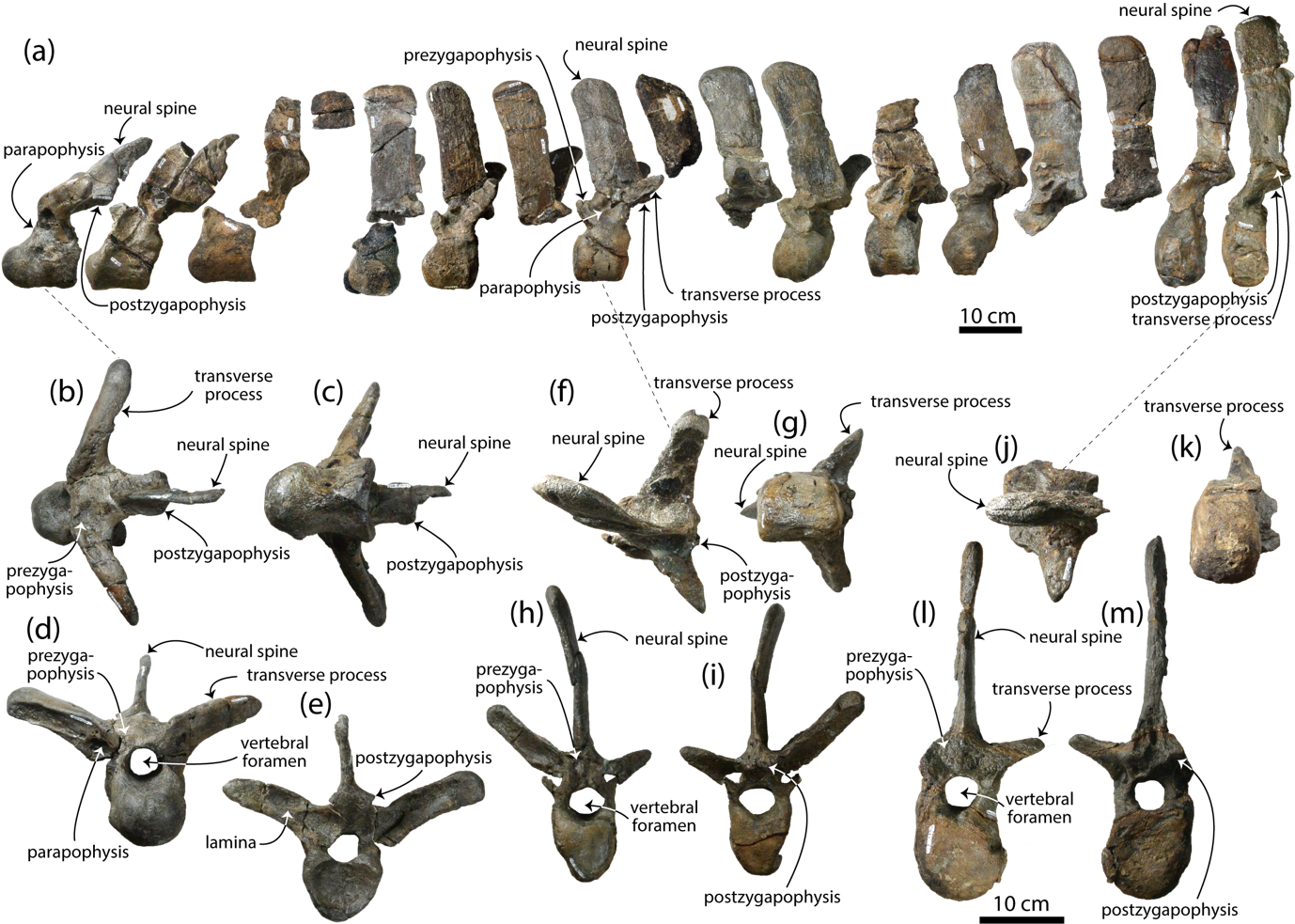


Supplementary Figure S13. Doral vertebrae in lateral view. First dorsal vertebra in dorsal (b), ventral (c), anterior (d), and posterior (e) views. Eighth dorsal vertebra in dorsal (f), ventral (g), anterior (h), and posterior (i) views. Seventeenth dorsal vertebra in dorsal (j), ventral (k), anterior (l), and posterior (m) views.


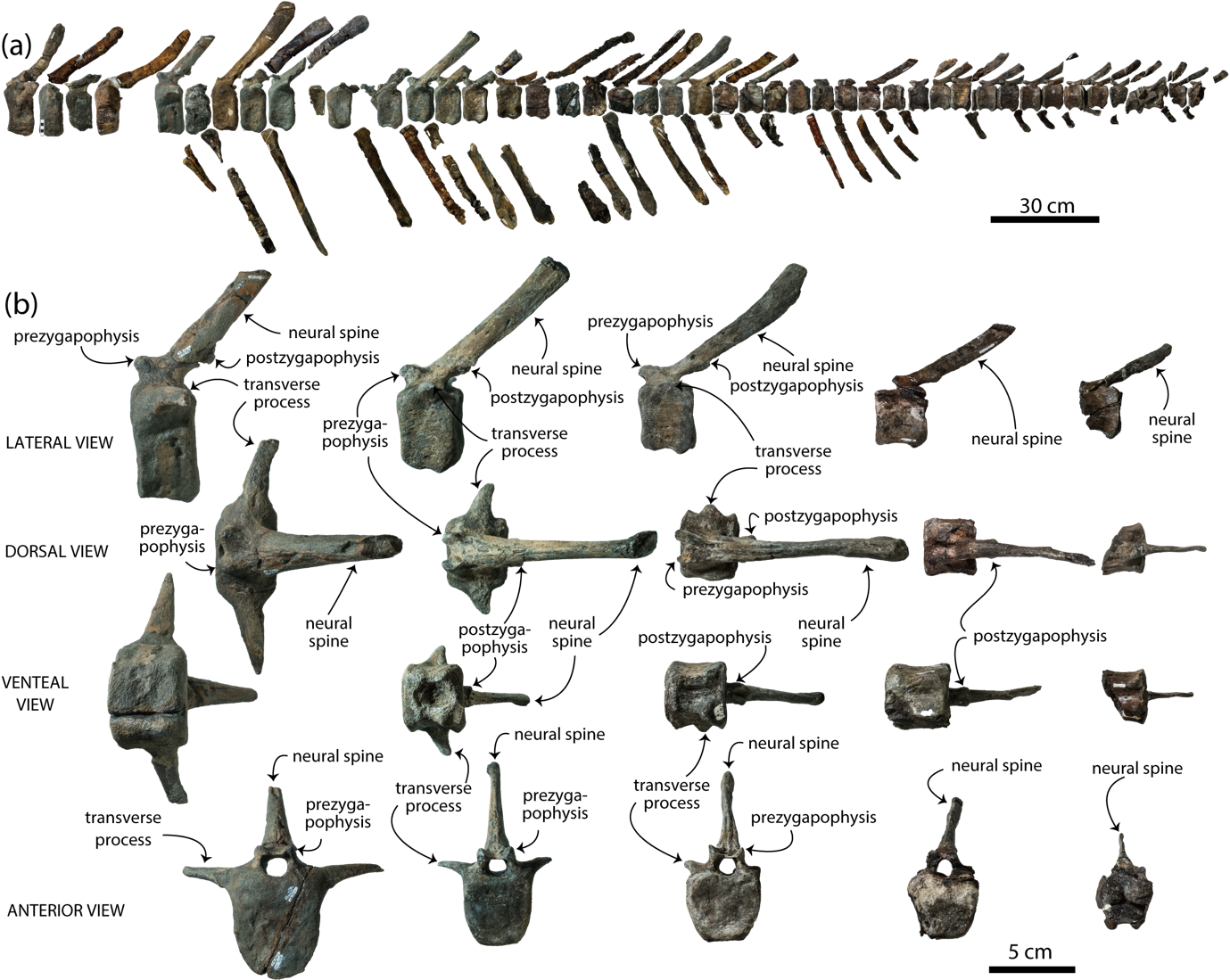


Supplementary Figure S14. All preserved caudal vertebrae and chevrons in left lateral view (a). Five caudal vertebrae in lateral, dorsal, ventral, and anterior views (b).


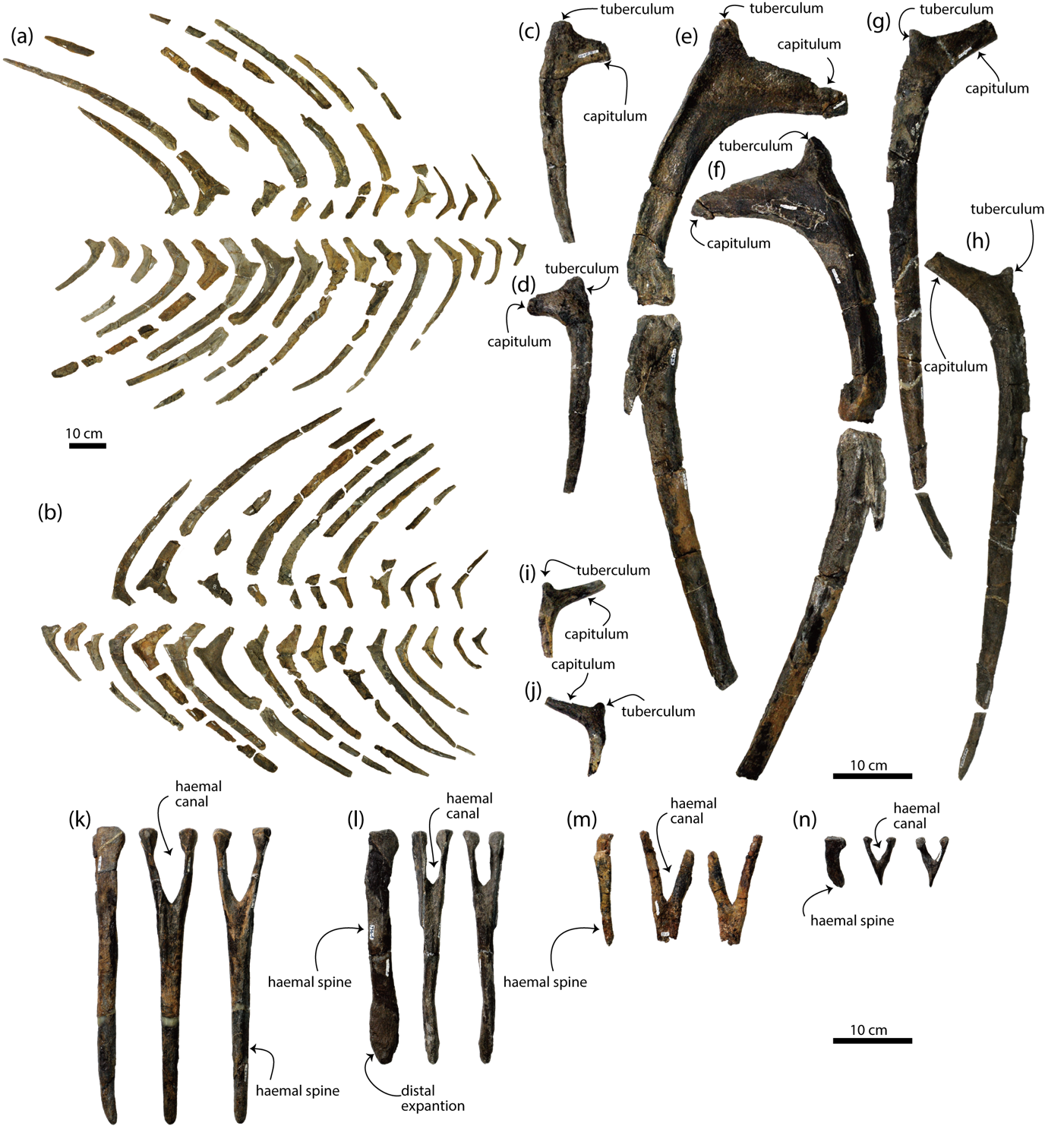


Supplementary Figure S15. Last cervical and fifteen dorsal ribs organized in order in anterior (a) and posterior (b) views. Last cervical rib (c and d), sixth rib (e and f), eleventh rib (g and h), and fifteenth rib (i and j) in anterior (c, e, g, and i) and posterior (d, f, h, and j) views. Fifth (k), eighth (l), nineteenth (m), and thirty-seventh (n) haemal arches in lateral, anterior, and posterior views.


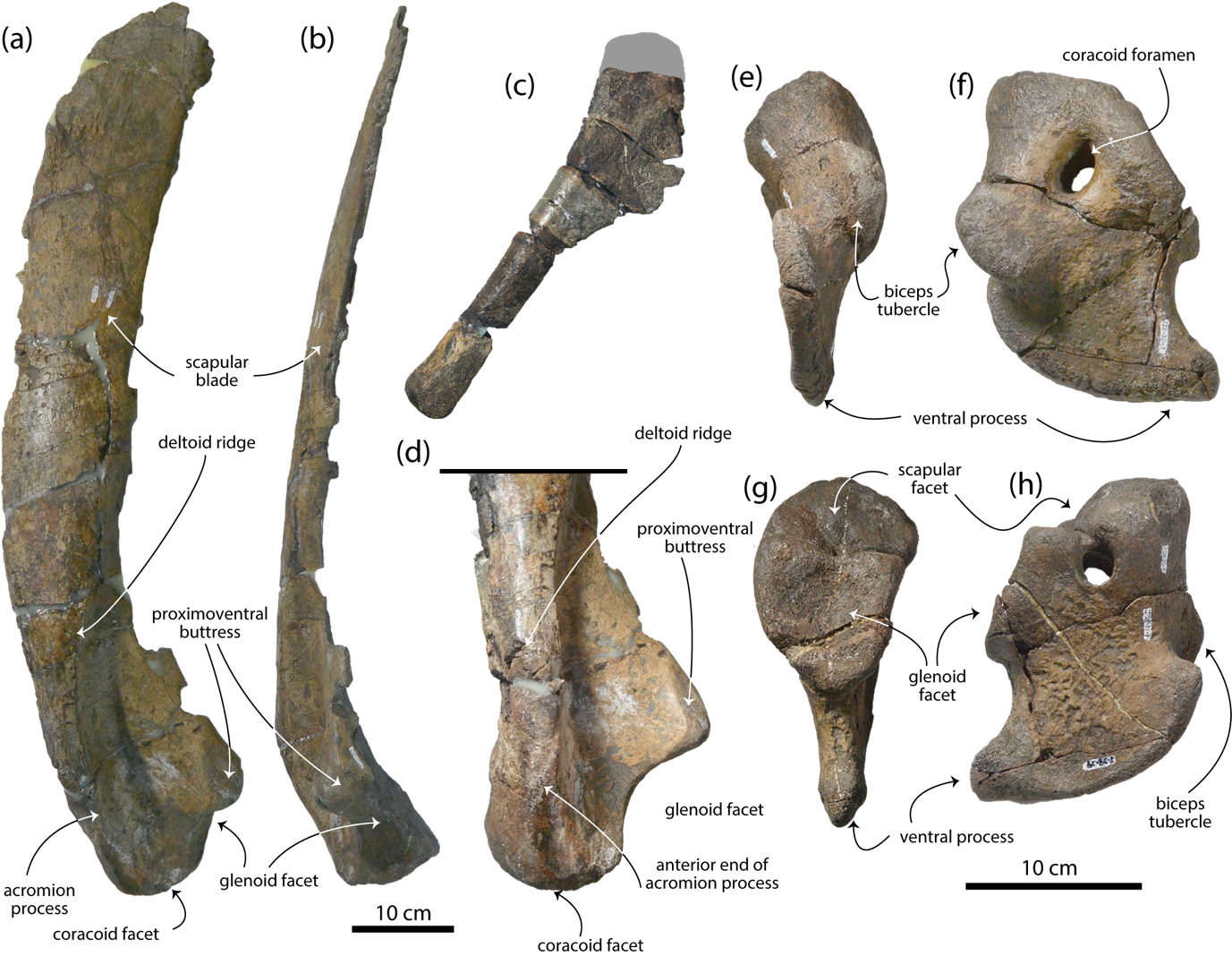


Supplementary Figure S16. Left scapula in lateral (a) and posterior (b) views. Proximal end of the left scapula on lateral view (d). Right sternum in ventral view (c). Left coracoid in anterior (e), lateral (f), posterior (g), and medial (h) views. Scale below (b) is for (a) to (d), and scale below (g) is for (e) to (h).


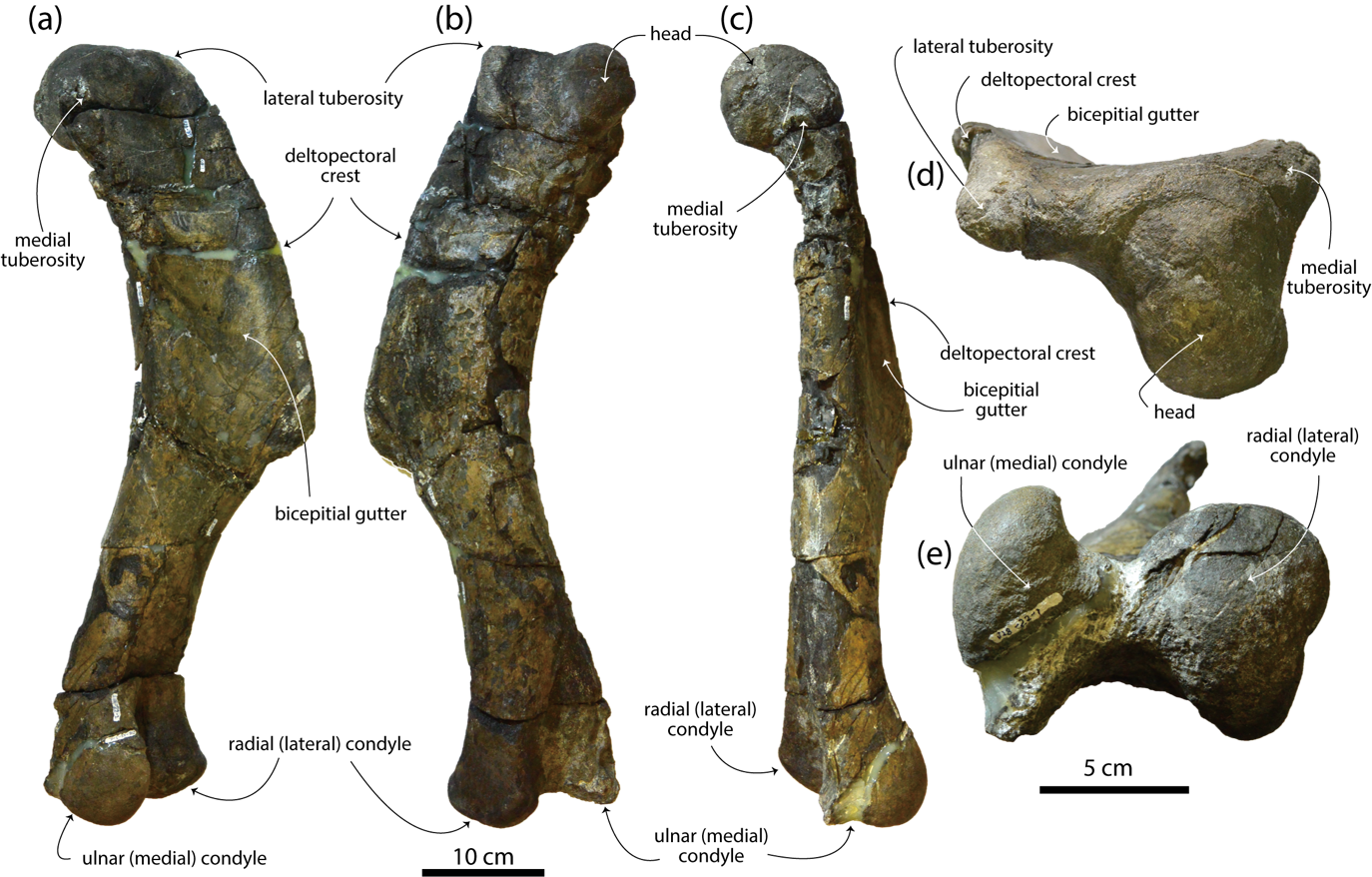


Supplementary Figure S17. Left humerus in anterior (a), posterior (b), medial (c), proximal (d), and distal (e) views.


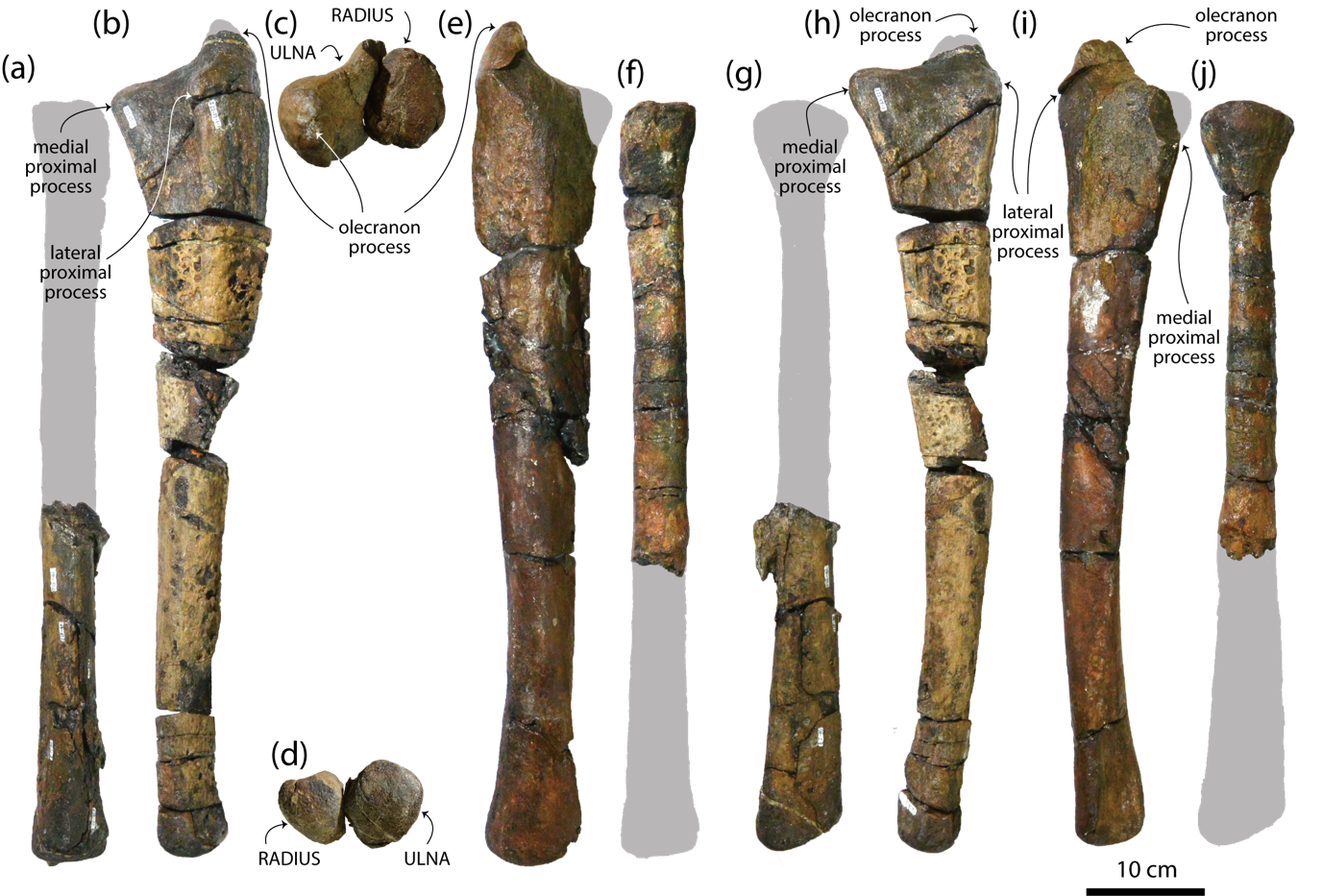


Supplementary Figure S18. Left ulna and radius in medial (a and b), distal (d), and anterior (g and h) views. Right ulna and radius in medial (e and f), proximal (c), and anterior (i and j) views.


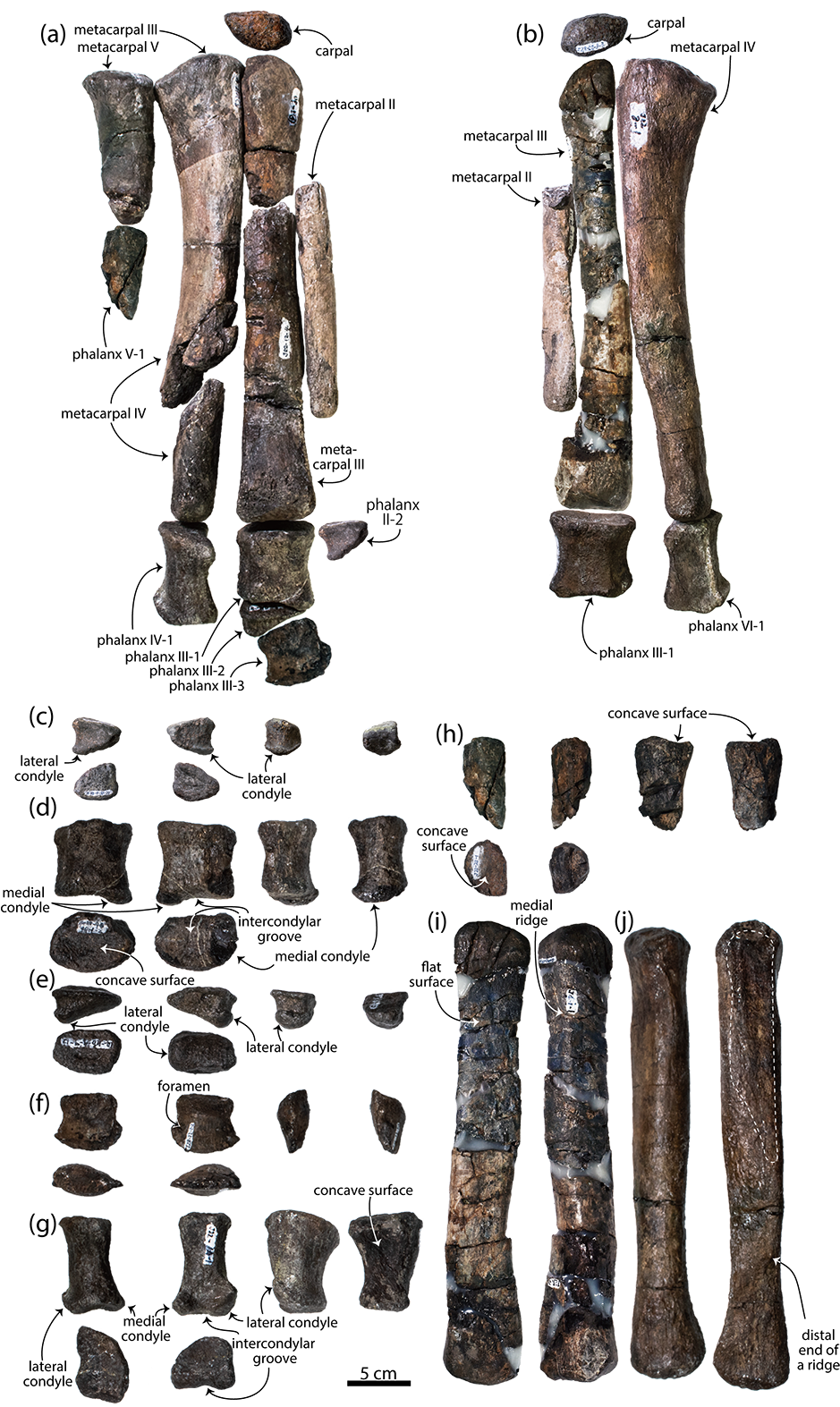


Supplementary Figure S19. Right (a) and left (b) manus in dorsal view. Manual phalanges II-2 (c), III-1 (d), III-2 (e), III-3 (f), and IV-1 (g), and V-1(h) in dorsal, ventral, lateral, medial, proximal and distal views. Left metacarpals III (h) and IV (i) in lateral and medial views.


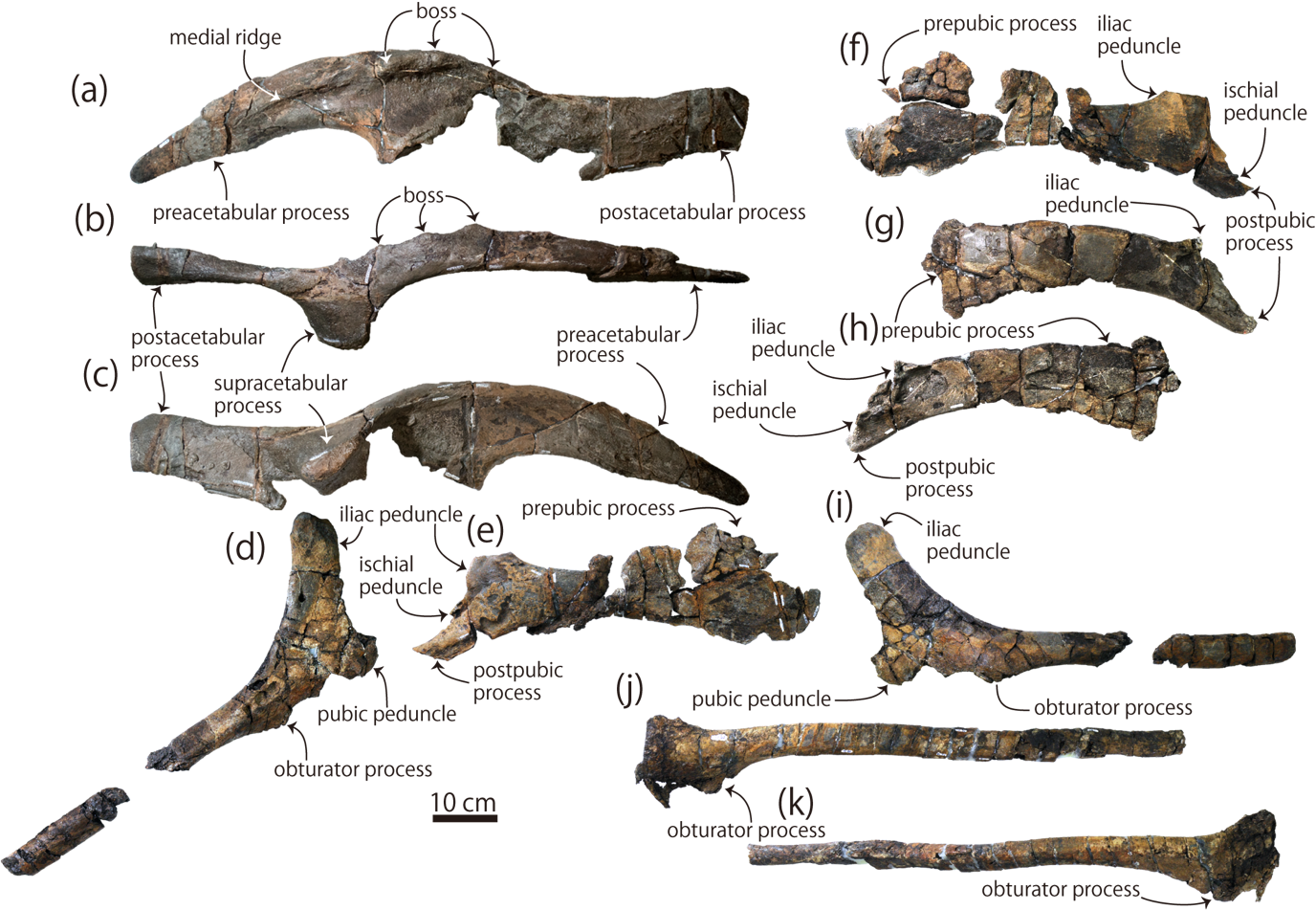


Supplementary Figure S20. Left ilium in medial (a), dorsal (b), and lateral (c) views. Right pubis (e and f), left pubis (g and h), right ischium (d and i), and left ischium (j and k) in lateral (e, e, g, and j) and medial (f, h, i, and k) views.


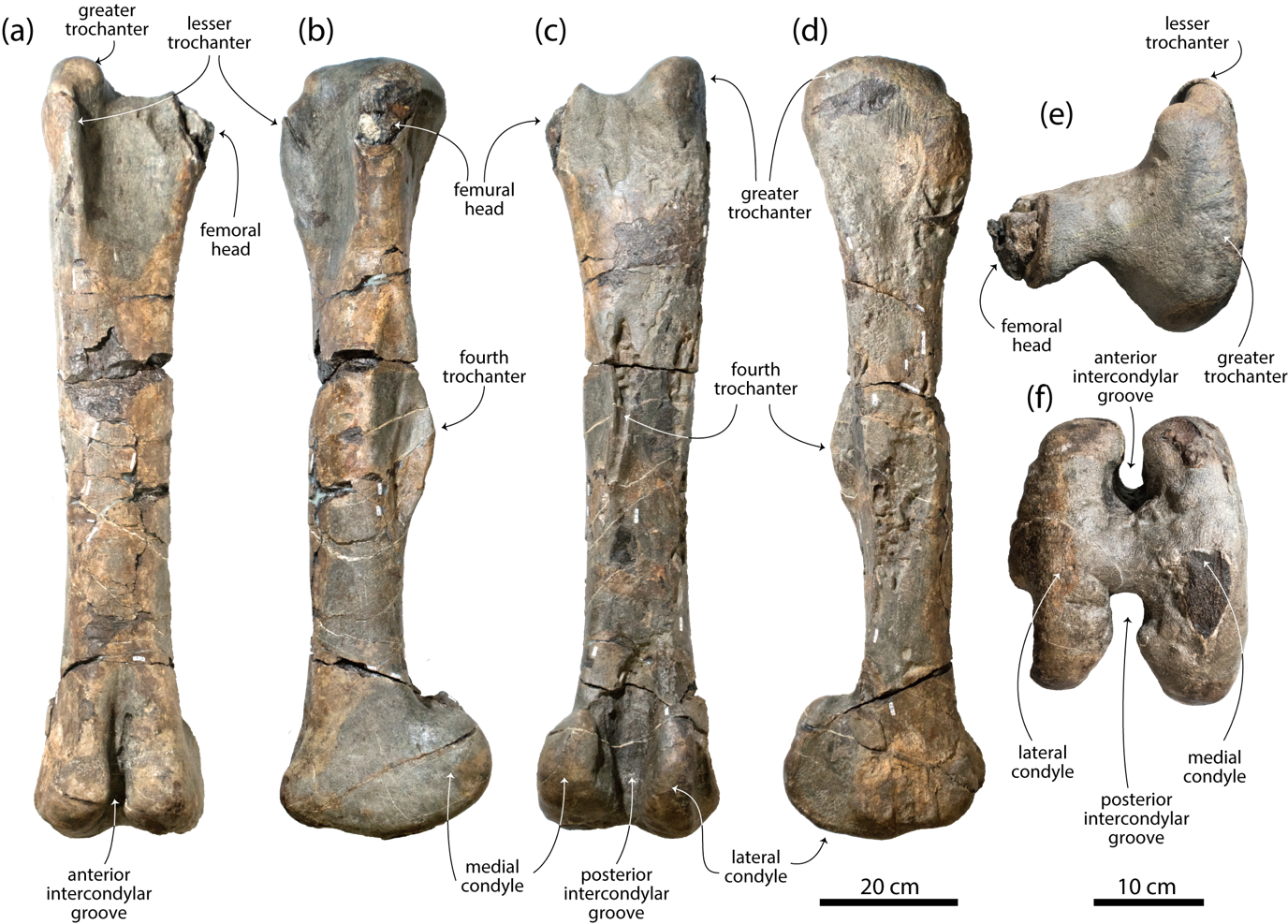


Supplementary Figure S21. Right femur in anterior (a), medial (b), posterior (c), lateral (d), proximal (e), and distal (f) views.


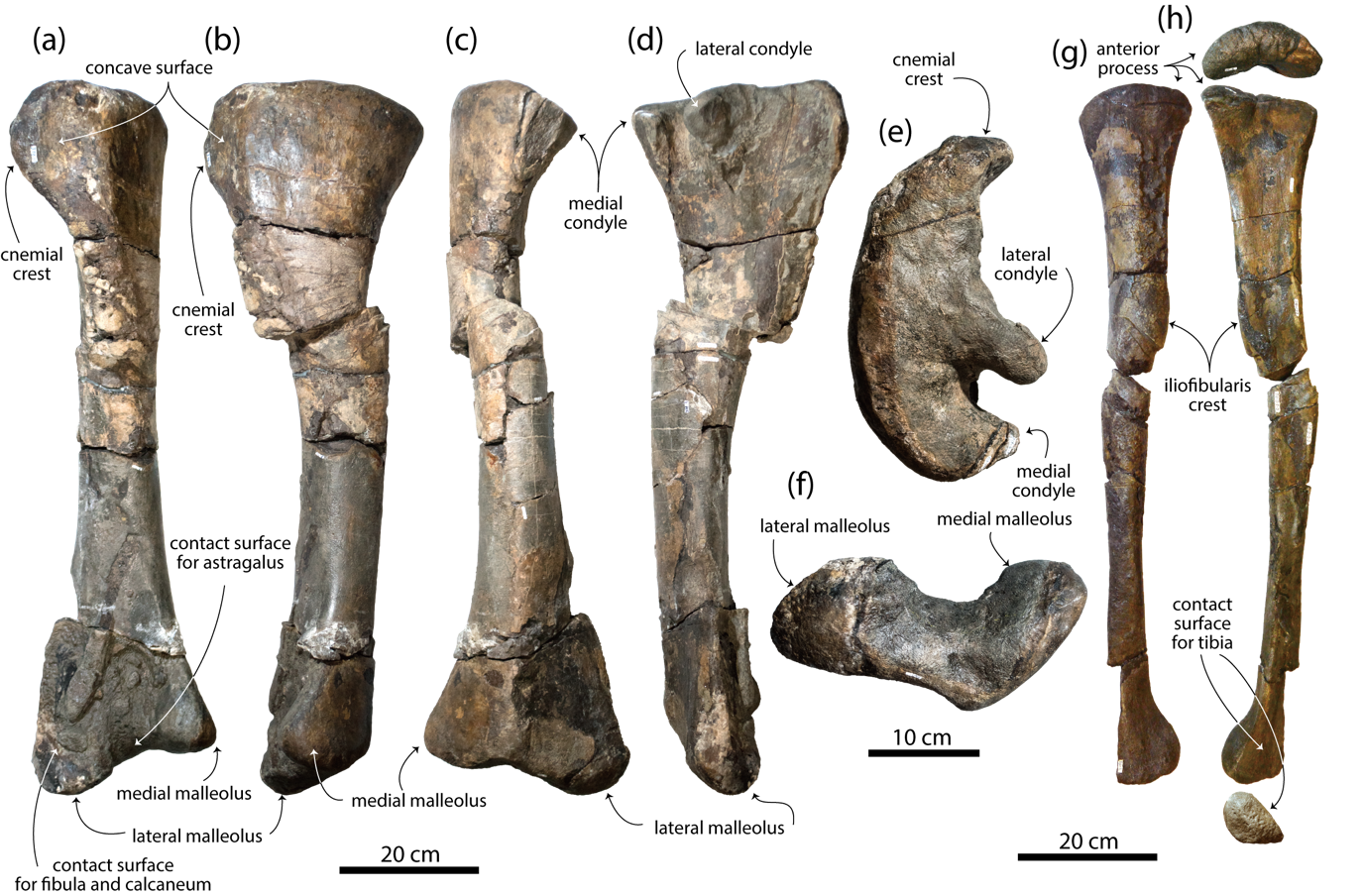


Supplementary Figure S22. Right tibia in anterior (a), medial (b), posterior (c), lateral (d), proximal (e), and distal (f) views. Right fibula in lateral (g), proximal and medial (h) views.


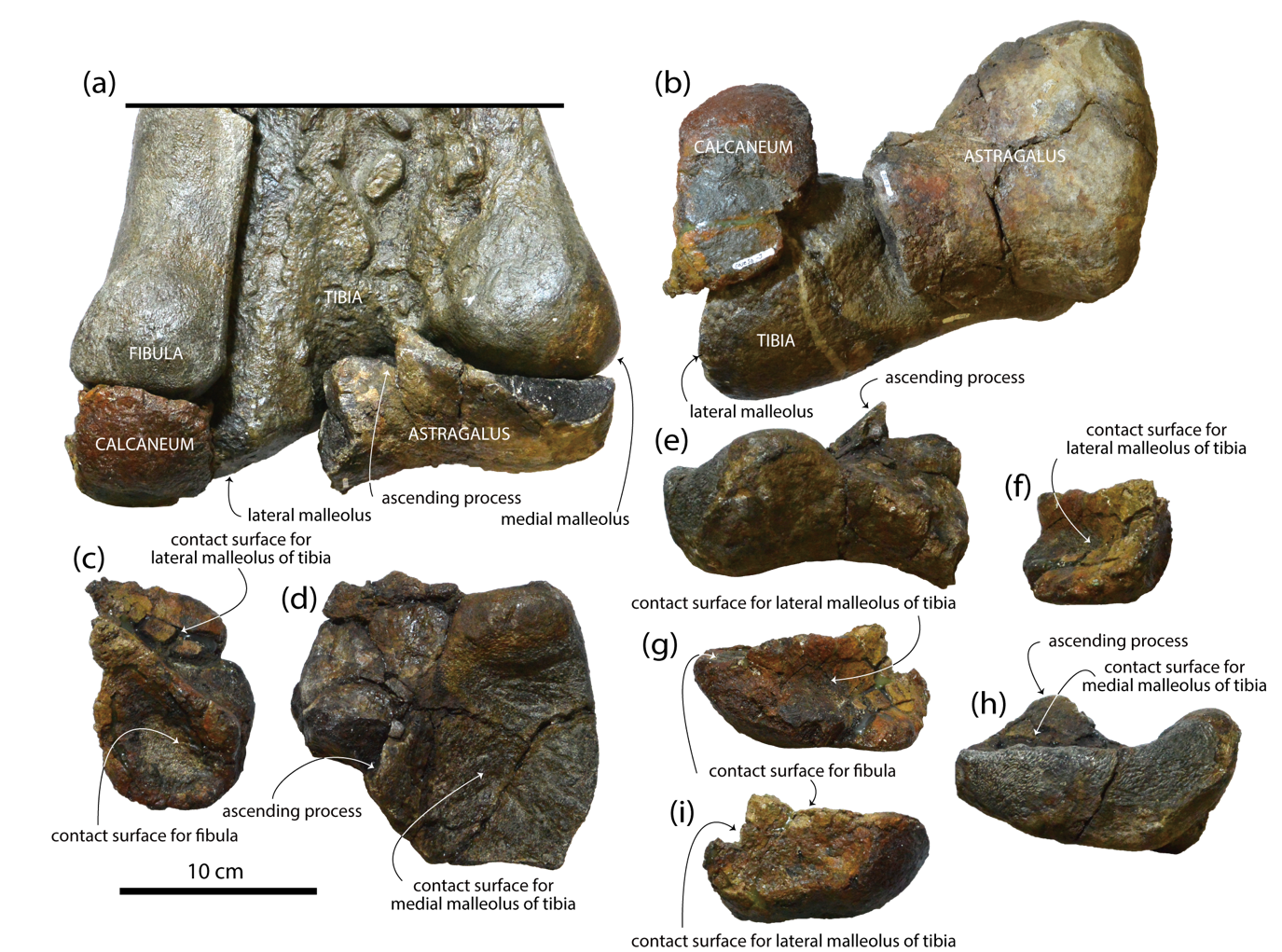


Supplementary Figure S23. Right tibia, fibula, astragalus, and calcaneum in articulation in anterior view (a) and distal (b) views. Right astragalus and calcaneum in proximal (c and d), posterior (e and f), medial (g and h), and lateral (i) views.


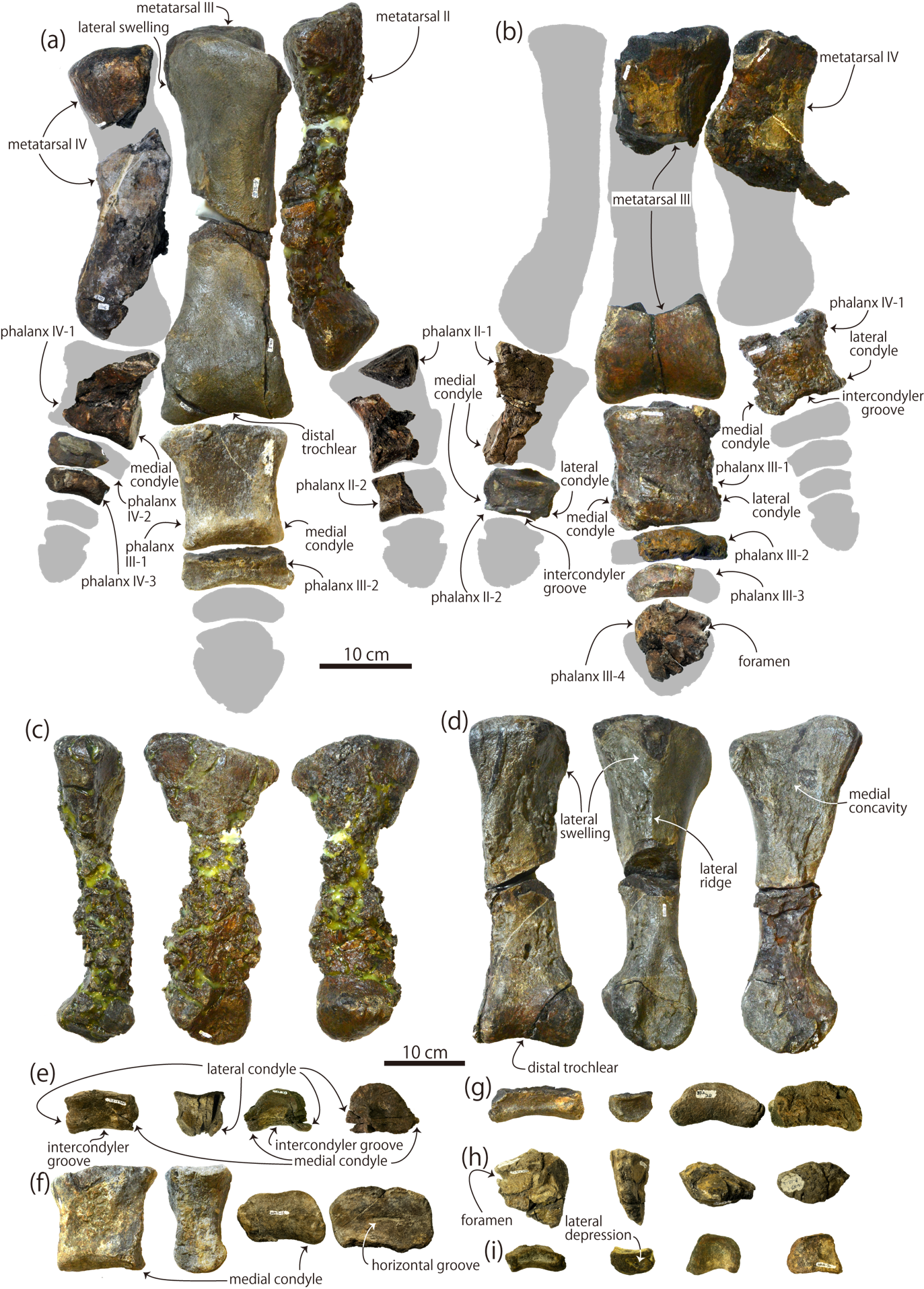


Supplementary Figure S24. Right (a) and left (b) pes in dorsal view. Right metatarsals II (c) and III (d) in dorsal, lateral, and medial views. Pedal phalanges II-2 (e), III-1 (f), III-2 (g), III-4 (h), and IV-3 (i) in ventral, lateral, distal, and proximal views.


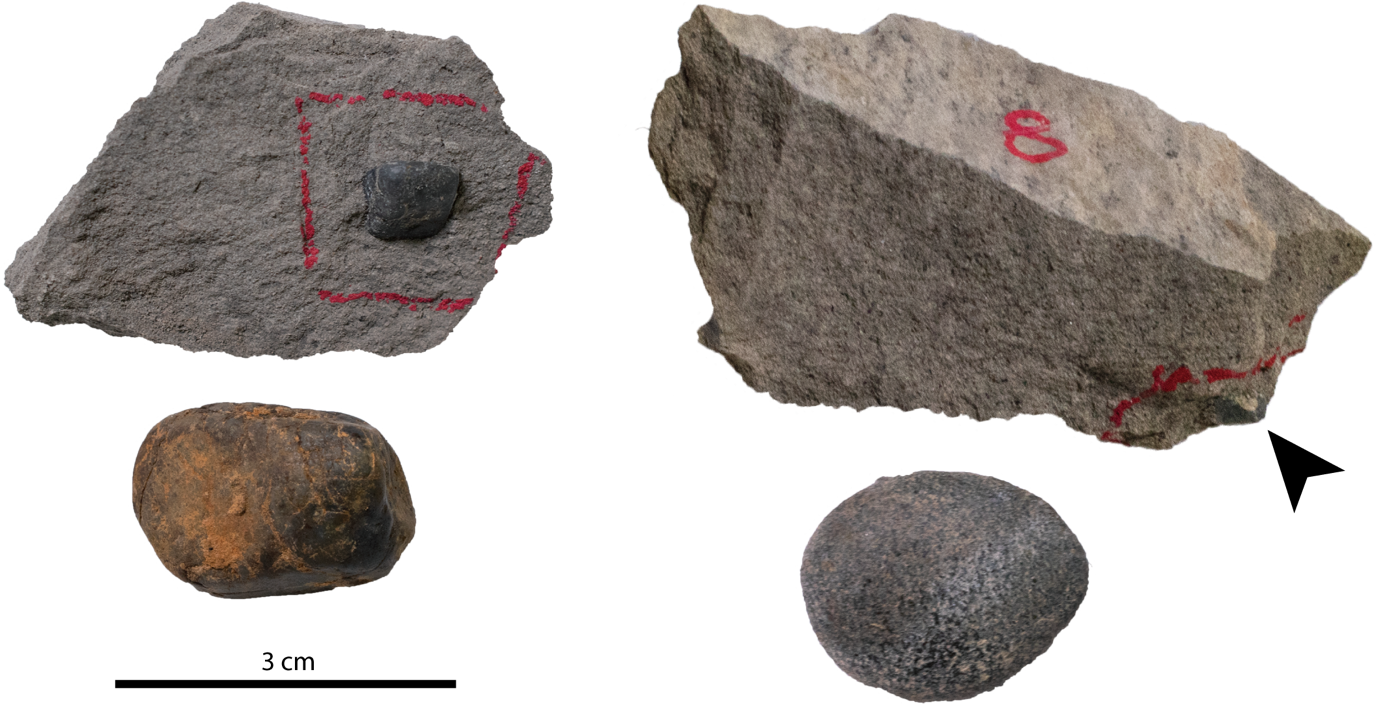


Supplementary Figure S25. Potential gastroliths collected together with *Kamuysaurus japonicus*. Arrowhead points a small pebble embed in the matrix.


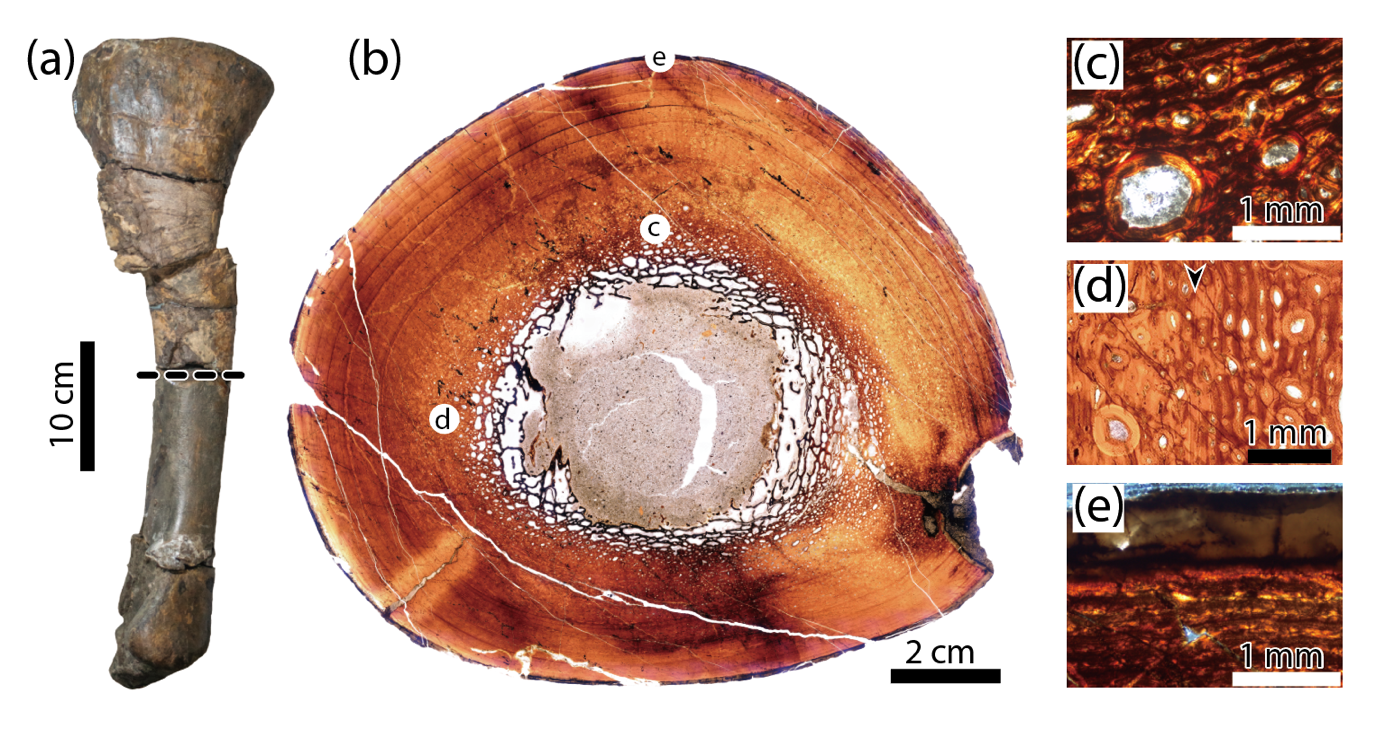


Supplementary Figure S26. (a) Medial view of the right tibia. Dashed line indicates minimum diaphysis where thin section was made. (b) Whole thin section image under plane polarized light; C-E: zoomed in images of areas indicated in 1B. (c) Cross polarized image of inner cortex. (d) Plane polarized image of inner cortex, and the arrow indicates the point where vascular density and space changes. (e) Cross polarized image of the outer cortex.


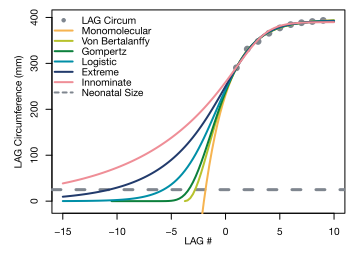


Supplementary Figure S27. Age estimations by fitting growth models to the LAG incremental pattern. LAG circumferences are listed in Supplementary Table S5.


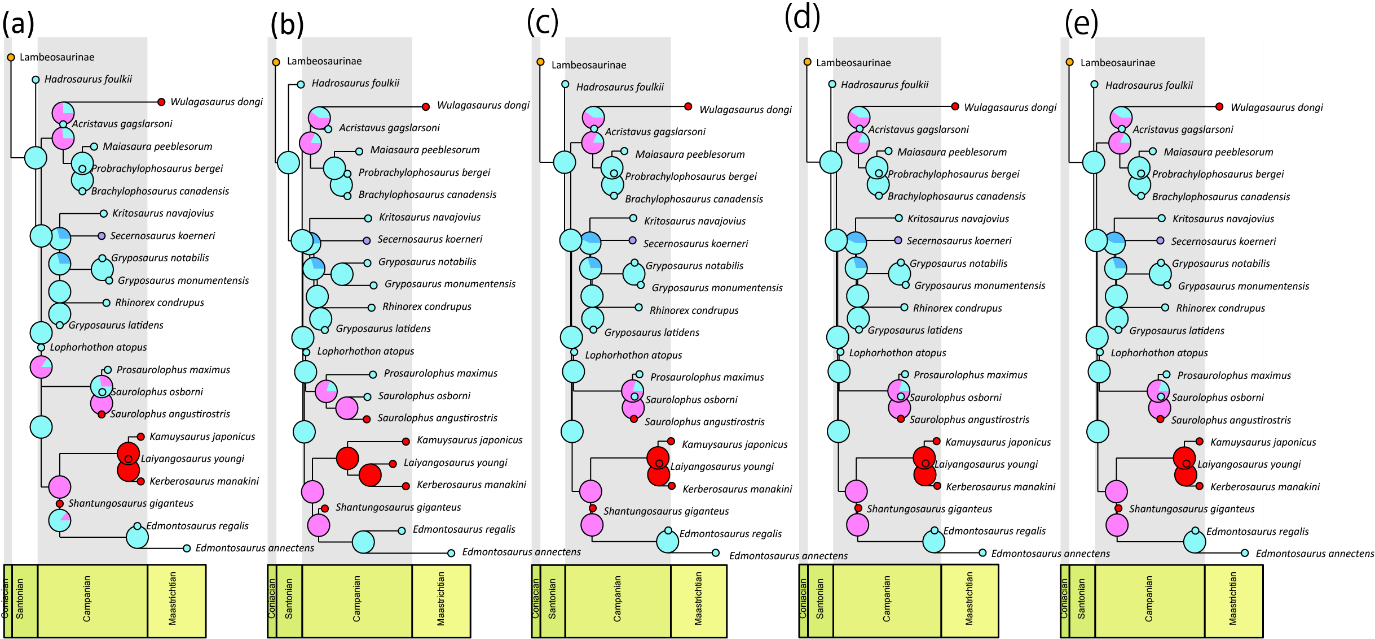


Supplementary Figure S28. Results of DEC analyses based on five different time-calibration methods. (a) basic; (b) equal; (c) additive, (d) zero-branch length additive; (e) minimum branch length. Red: Asia; blue: North America; purple: South America; pink: Asia + North America; dark blue: North America + South America; orange: Asia + North America + Europe.

**References for Supplementary Figures**

1 Morozumi, Y. Late Cretaceous (Campanian and Maastrichtian) ammonites from Awaji Island, Southwest Japan. *Bull. Osaka Mus. Nat. Hist.* **39**, 1-58 (1985).

2 Shigeta, Y., Tanabe, K. & Izukura, M. *Gaudryceras izumiense* Matsumoto and Morozumi, a Maastrichtian ammonoid from Hokkaido and Alaska and its biostratigraphic implications. *Paleontol. Res.* **14**, 202-211, doi:10.2517/1342-8144-14.3.202 (2010).

3 Shigeta, Y., Tsutsumi, Y. & Misaki, A. U–Pb age of the *Didymoceras awajiense* Zone (upper Campanian, Cretaceous) in the Aridagawa area, Wakayama, southwestern Japan. *Bull. Natl. Mus. Nat. Sci. Ser. C, Geol. & Paleontol.* **43**, 11-18 (2017).

4 Shigeta, Y., Izukura, M. & Tsutsumi, Y. An early Maastrichtian (latest Cretaceous) ammonoid fauna from the Soya Hill area, Hokkaido, northern Japan. *Bull. Hobetsu Mus.* **32**, 7-41 (2017).
